# Thiyl radical reversible-deactivation polymerization *via* degenerative transfer with vinyl sulfides

**DOI:** 10.1039/d5sc06492a

**Published:** 2025-10-31

**Authors:** Huajuan Hu, Ping Yi, Derong Cao, Hanchu Huang

**Affiliations:** a School of Chemistry and Chemical Engineering, State Key Laboratory of Luminescent Materials and Devices, South China University of Technology Guangzhou 510640 China; b School of Materials Science and Engineering, Key Laboratory for Polymeric Composite and Functional Materials of Ministry of Education, Sun Yat-Sen University Guangzhou 510006 China huanghch9@mail.sysu.edu.cn

## Abstract

Precise control over thiyl radical polymerizations remains a significant challenge in polymer chemistry, particularly within the framework of traditional reversible-deactivation radical polymerization (RDRP) techniques. In this work, we introduce a novel thiyl radical reversible-deactivation polymerization (SRDP) strategy that employs vinyl sulfides as degenerative transfer agents to reversibly deactivate the propagating thiyl radicals, thus enabling direct and efficient control over the thiyl radical polymerizations to afford polymers with tunable molecular weights and low dispersities. The controlled nature of this polymerization was further confirmed by first-order kinetics, a linear relationship between molecular weight and conversion, and efficient chain extension. In addition, density functional theory calculations offered valuable insights into the reversible-deactivation ability of vinyl sulfides. The versatility of the SRDP method was demonstrated through its compatibility with a wide range of thiyl radical polymerizations and its successful application in synthesizing structurally diverse copolymers. This study represents a new reversible-deactivation pattern for thiyl radical polymerizations and would lead to a powerful platform for the precise synthesis of complex functional materials.

## Abstract 2

This study presents a novel vinyl sulfide-based radical dynamic chemistry that effectively controls various thiyl radical polymerizations, resulting in sulfur-containing polymers with well-defined architectures.

## Graphical abstract

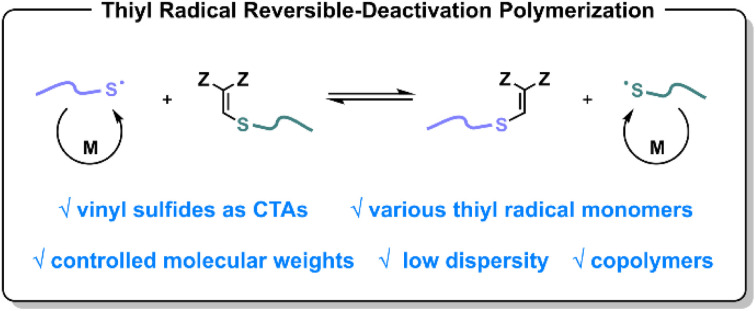

## Introduction

**Fig. 1:**
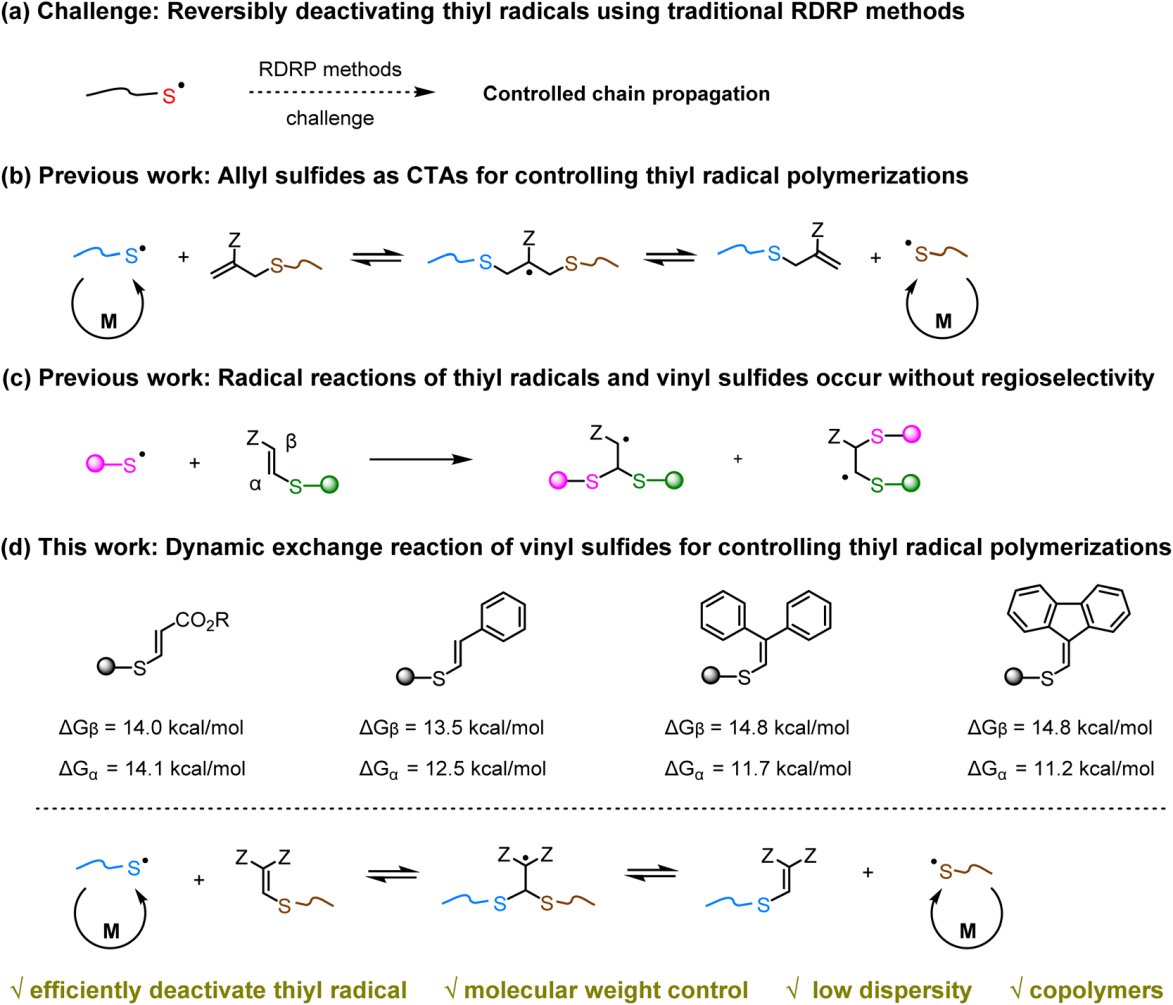
Design of new thiyl radical reversible-deactivation polymerization strategy.

The emergence of new polymerization methods continues to inspire innovation in polymer chemistry, enabling the creation of functional polymers with unique structures and desirable properties.^1–4^ Among these advancements, thiyl radical polymerization, a chain-growth polymerization propagated *via* thiyl radicals, has garnered significant attention recently due to its unique ability to synthesize polymers with extended main-chain structures.^5–16^ However, due to the lack of reversible-deactivation radical polymerization (RDRP) methods, these polymerizations have suffered from poor control over molecular weight and high dispersity, thereby limiting their ability to produce polymers with well-defined architectures (Fig. 1a). To date, strategies developed to address these issues have primarily focused on copolymerizing with vinyl monomers^17–20^ or desulfurization^21–25^ to generate a stabilized carbon radical capable of being controlled by reversible addition-fragmentation chain transfer (RAFT) agents. Although these approaches have shown promise, they still face limitations regarding efficiency, atom economy, and general applicability. To overcome these challenges, we recently proposed the first thiyl radical reversible-deactivation polymerization (SRDP) strategy, wherein allyl sulfides serve as chain transfer agents (CTAs) to reversibly deactivate propagating thiyl radicals (Fig. 1b).^26^ This strategy enables precise control over the thiyl radical polymerization process, resulting in polymers with controlled molecular weights and narrow dispersities. Despite this advancement, thiyl radical reversible-deactivation polymerization is still in its infancy, and there is a pressing need to expand the RDRP toolbox for better control of various thiyl radical polymerizations. Therefore, in this work, we aim to introduce a new strategy for thiyl radical reversible-deactivation polymerization and broaden the range of thiyl radical polymerizations that can be effectively controlled through this technique.

To achieve this goal, it is essential to identify suitable chemistries capable of reversibly deactivating the thiyl radicals. Vinyl sulfides are highly versatile compounds with widespread applications in organic chemistry and polymer science.^27–30^ In polymer science, they are frequently used as reversible covalent linkages to synthesize linear recyclable polymers and dynamic covalent cross-linked networks.^31–36^ Due to their structural similarity to allyl sulfides, vinyl sulfides hold great promise as CTAs for controlling thiyl radical polymerizations. However, existing studies on reversible exchange reactions of vinyl sulfides have primarily focused on the anionic mechanism, whereas investigations into the radical mechanism remain elusive. This challenge arises because thiyl radicals can add to both the α- and β-positions of vinyl sulfides, thereby complicating their use as handles for the reversible exchange of thiyl radicals (Fig. 1c).^37,38^ To better understand this issue, we performed density functional theory (DFT) calculations (Fig. 1d). The results showed that for carbonyl-substituted vinyl sulfides, the energy barriers for α- and β-addition are comparable. Interestingly, when replacing the carbonyl group with a phenyl moiety and further tuning the substituents, we observed an increase in the energy barrier difference between α-addition and β-addition, thereby favoring thiyl radical addition at the α-position. Motivated by these findings, we wondered if we could achieve a reversible radical exchange reaction between thiyl radicals and vinyl sulfides by optimizing their structures. If successful, it would not only provide new CTAs for controlling thiyl radical polymerizations but also expand the applications of the dynamic chemistry of vinyl sulfides in polymer synthesis.

Herein, we present a novel radical dynamic chemistry based on vinyl sulfides and demonstrate its successful application in controlling the thiyl radical polymerizations. As depicted in Fig. 1d, the propagating thiyl radical adds to the double bond of a vinyl sulfide-capped polymer, resulting in an intermediate radical that subsequently undergoes β-elimination. This process generates a dormant polymer and a new thiyl radical, establishing a dynamic equilibrium between active and dormant states, thereby allowing for precise control over thiyl radical polymerizations to produce polymers with predictable molecular weights, low dispersities, and tailored architectures. DFT calculations further support the proposed mechanism, highlighting the critical role of vinyl sulfides in controlling the polymerization process. The versatility of the SRDP method is demonstrated by its effective applications in controlling the polymerizations of various thiyl radical monomers and synthesizing structurally diverse copolymers, thus establishing SRDP as a robust platform for creating well-defined macromolecules with a variety of main-chain functionalities.

## Results and discussion

To evaluate the feasibility of our strategy, we chose the radical ring-opening polymerization (ROP) of cyclic allylic sulfides^5–9^ and the radical transfer polymerization of thiocyanate alkenes^10,11^ as the model polymerizations. A key reason for this choice is that these polymerizations represent typical thiyl radical polymerizations and hold great potential for synthesizing macromolecules with complex main-chain functionalities. Accordingly, macrocyclic monomer M1 was first synthesized as a model compound to optimize the reaction conditions (see Tables S1–S5 for details). The free radical polymerization of M1 was carried out using AIBN as the initiator in DMF at 70 °C under a nitrogen atmosphere, which successfully afforded the polymer P1 with a number-average molecular weight (*M*_n,SEC_) of 27.2 kDa and a dispersity (*Đ*) of 1.77 (Table 1, entry 1). Encouraged by this result, we then focused on achieving control over the polymerization by utilizing vinyl sulfides as CTAs. First, a carbonyl-substituted vinyl sulfide (CTA1), commonly used in dynamic covalent chemistry *via* anionic mechanism, was tested, but it showed limited control over the polymerization (Table 1, entry 2). Building on our DFT calculation findings mentioned above, various phenyl substituents were introduced at the vinyl group of the CTAs (CTA2–CTA5). Indeed, a significant improvement in the controllability of polymerization was observed, with fluorenyl-substituted CTA (CTA4) proving to be the most effective, yielding P1 with *M*_n,SEC_ of 9.2 kDa and *Đ* of 1.38 (Table 1, entries 3–6). Further investigation of different R groups in the CTAs (CTA6–CTA9) revealed that CTA7, with the 3-ethoxy-3-oxopropyl R-group, was the optimal choice for the polymerization, as indicated by the close agreement between the theoretical molecular weight (*M*_n,theo_) and the NMR molecular weight (*M*_n,NMR_) (Table 1, entries 7–10). Notably, although CTA9 afforded polymers with relatively low dispersity, it also showed poor conversion and a mismatch between *M*_n,theo_ and *M*_n,NMR_, likely due to the limited re-initiation ability of the phenylthiyl radicals derived from CTA9. Next, using CTA7 as the optimal CTA, we explored the impact of solvents and found that the polymerization performed efficiently in DMF and dimethyl sulfoxide (DMSO) but was less efficient in tetrahydrofuran (THF), 1,4-dioxane, and toluene (Table S2). Reducing the amount of AIBN to 0.1 equivalent (referenced to CTA) resulted in sluggish polymerization. In contrast, increasing the amount of AIBN to 0.8 equivalent improved the polymerization rate while preserving good control, representing the best condition tested (Table S4).^39^ Under the optimized reaction conditions, different molecular weights of P1 were targeted by adjusting the monomer/CTA ratio from 10 : 1 to 100 : 1. As expected, the *M*_n_ increased with the monomer/CTA ratio, while the corresponding *Đ* remained at a relatively low value, supporting the controlled nature of the polymerization (Table 1, entries 11–14).

**Table 1:** Evaluation of polymerization conditions

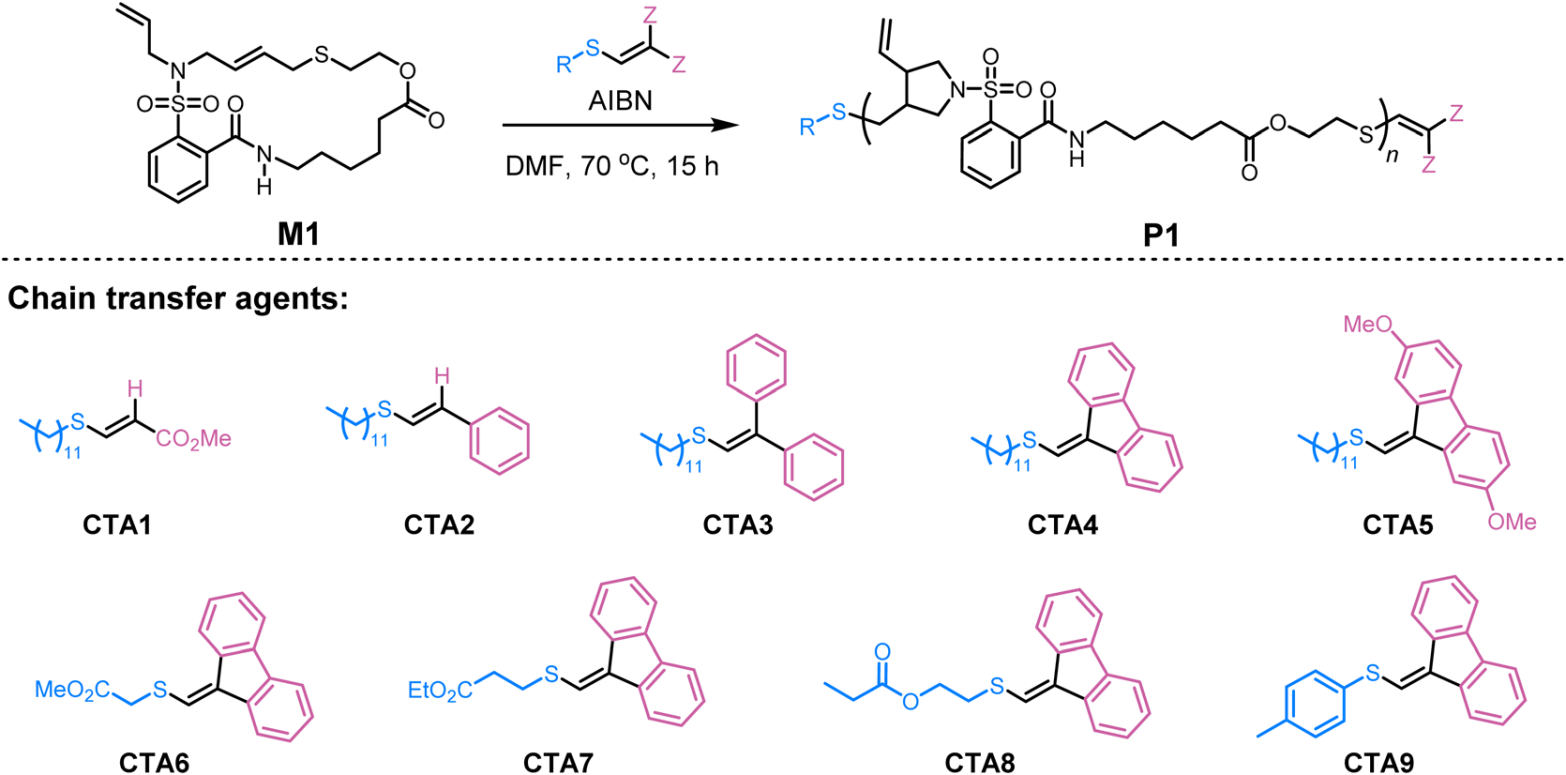							
Entry^a^	[M]_0_/[CTA]_0_/[AIBN]_0_	CTA	Conversion^b^	*M*_n,theo_ c (kDa)	*M*_n,NMR_ d (kDa)	*M*_n,SEC_ e (kDa)	*Đ* e
1	25/0/1	—	78%	—	—	27.2	1.77
2	25/1/0.5	CTA1	47%	5.8	—	25.4	1.69
3	25/1/0.5	CTA2	48%	6.0	22.5	13.2	1.55
4	25/1/0.5	CTA3	56%	6.9	12.7	11.4	1.45
5	25/1/0.5	CTA4	54%	6.7	7.5	9.2	1.38
6	25/1/0.5	CTA5	50%	6.3	10.9	9.1	1.36
7	25/1/0.5	CTA6	59%	7.2	7.8	9.3	1.36
8	25/1/0.5	CTA7	65%	7.9	8.3	9.7	1.38
9	25/1/0.5	CTA8	53%	6.5	7.5	8.9	1.39
10	25/1/0.5	CTA9	25%	3.2	4.6	5.4	1.28
11	10/1/0.8	CTA7	71%	3.6	4.3	5.6	1.22
12	25/1/0.8	CTA7	75%	9.1	9.0	10.7	1.38
13	50/1/0.8	CTA7	67%	15.9	14.0	17.2	1.47
14	100/1/0.8	CTA7	51%	24.1	25.0	21.1	1.55

a Experimental conditions: [M]_0_ = 0.1 M in entries 1–10 and [M]_0_ = 0.2 M in entries 11–14, conducted at 70 °C under a nitrogen atmosphere unless otherwise specified.

b Determined by ^1^H nuclear magnetic resonance (NMR) analysis of the crude reaction mixture.

c Calculated following the equation: *M*_n,theo_ = [M]_0_/[CTA]_0_ × MW^M^ × conversion + MW^CTA^, where MW^M^ and MW^CTA^ correspond to the molar mass of the monomer and CTA, respectively.

d Determined by ^1^H NMR analysis of the isolated polymers.

e Molecular weight and dispersity were determined by size-exclusion chromatography (SEC) in THF at 40 °C using polystyrene standards.

The generality of the SRDP strategy was further explored using M2, a macrocyclic monomer with a larger 25-membered ring. The corresponding polymers P2 with *M*_n,SEC_ ranging from 6.3 to 19.9 kDa were obtained at the monomer/CTA ratios ranging from 10 : 1 to 50 : 1, indicating that the ring size of the monomer had a negligible influence on the polymerization reactivity (Table 2, entries 1–3). This strategy also proved effective for the monomer M3, which has a different reaction mechanism and reactivity, yielding polymers P3 with *M*_n,SEC_ varying from 5.9 to 14.5 kDa and corresponding *Đ* values ranging from 1.32 to 1.58, confirming a good generality of the SRDP approach (Table 2, entries 4–5).

**Table 2:** Controlled radical ROP of cyclic allylic sulfides

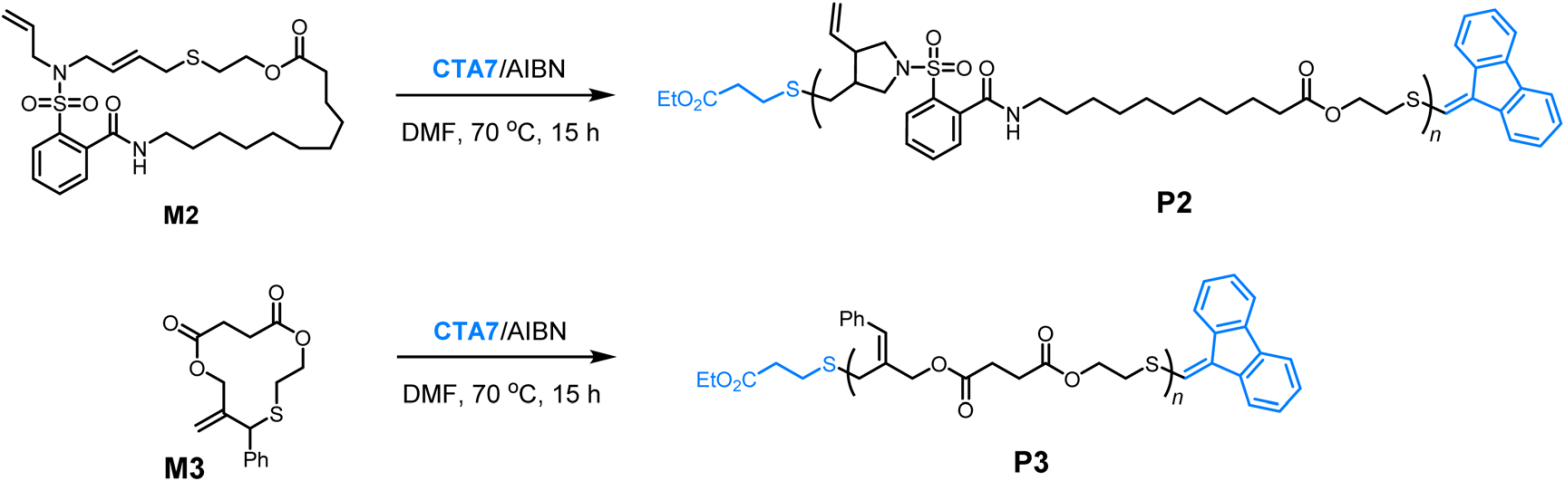							
Entry^a^	Monomer	[M]_0_/[CTA]_0_/[AIBN]_0_	Conversion^b^	*M*_n,theo_ c (kDa)	*M*_n,NMR_ d (kDa)	*M*_n,SEC_ e (kDa)	*Đ* e
1	M2	10/1/0.8	66%	3.9	4.5	6.3	1.32
2	M2	25/1/0.8	61%	8.5	9.5	13.1	1.39
3	M2	50/1/0.8	56%	15.3	14.4	19.9	1.51
4	M3	25/1/0.5	67%	5.4	5.9	5.9	1.32
5	M3	50/1/0.5	65%	10.3	10.9	9.4	1.48
6	M3	100/1/0.5	63%	19.6	21.9	14.5	1.58

a Experimental conditions: [M]_0_ = 0.2 M at 70 °C under a nitrogen atmosphere unless otherwise specified.

b Determined by ^1^H NMR analysis of the crude reaction mixture.

c Calculated following the equation: *M*_n,theo_ = [M]_0_/[CTA]_0_ × MW^M^ × conversion + MW^CTA^, where MW^M^ and MW^CTA^ correspond to the molar mass of the monomer and CTA, respectively.

d Determined by ^1^H NMR analysis of the isolated polymers.

e Molecular weight and dispersity were determined by SEC in THF at 40 °C using polystyrene standards.

**Fig. 2:**
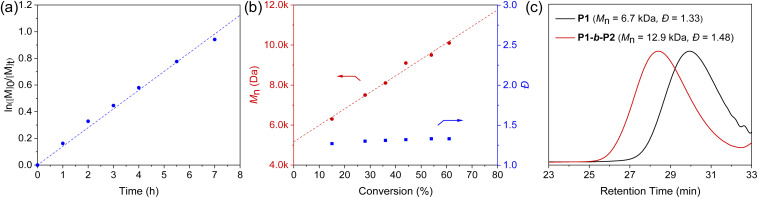
(a) Plot of ln([M]_0_/[M]_*t*_) *versus* reaction time for the polymerization of M1 at [M]_0_/[CTA]_0_ = 25/1. (b) Plots of *M*_n_ (red) and *Đ* (blue) *versus* monomer conversion for the polymerization of M1 under identical conditions. (c) SEC analysis for the diblock copolymerization.

To further validate the controlled nature of the polymerization, we monitored the kinetics by sampling the reaction at different time intervals. The polymerization demonstrated first-order kinetic behavior, with a linear increase in *M*_n_ as a function of monomer conversion while maintaining low dispersities (Fig. 2a and b). In addition, a macromolecular CTA P1 (*M*_n,SEC_ = 6.7 kDa, *Đ* = 1.33), which was synthesized *via* the homopolymerization of M1, could be successfully chain-extended using monomer M2, yielding the diblock copolymer P1-*b*-P2 with *M*_n,SEC_ of 12.9 kDa and *Đ* of 1.48 (Fig. 2c). These findings strongly support a controlled chain-growth mechanism to the polymerization.

**Fig. 3:**
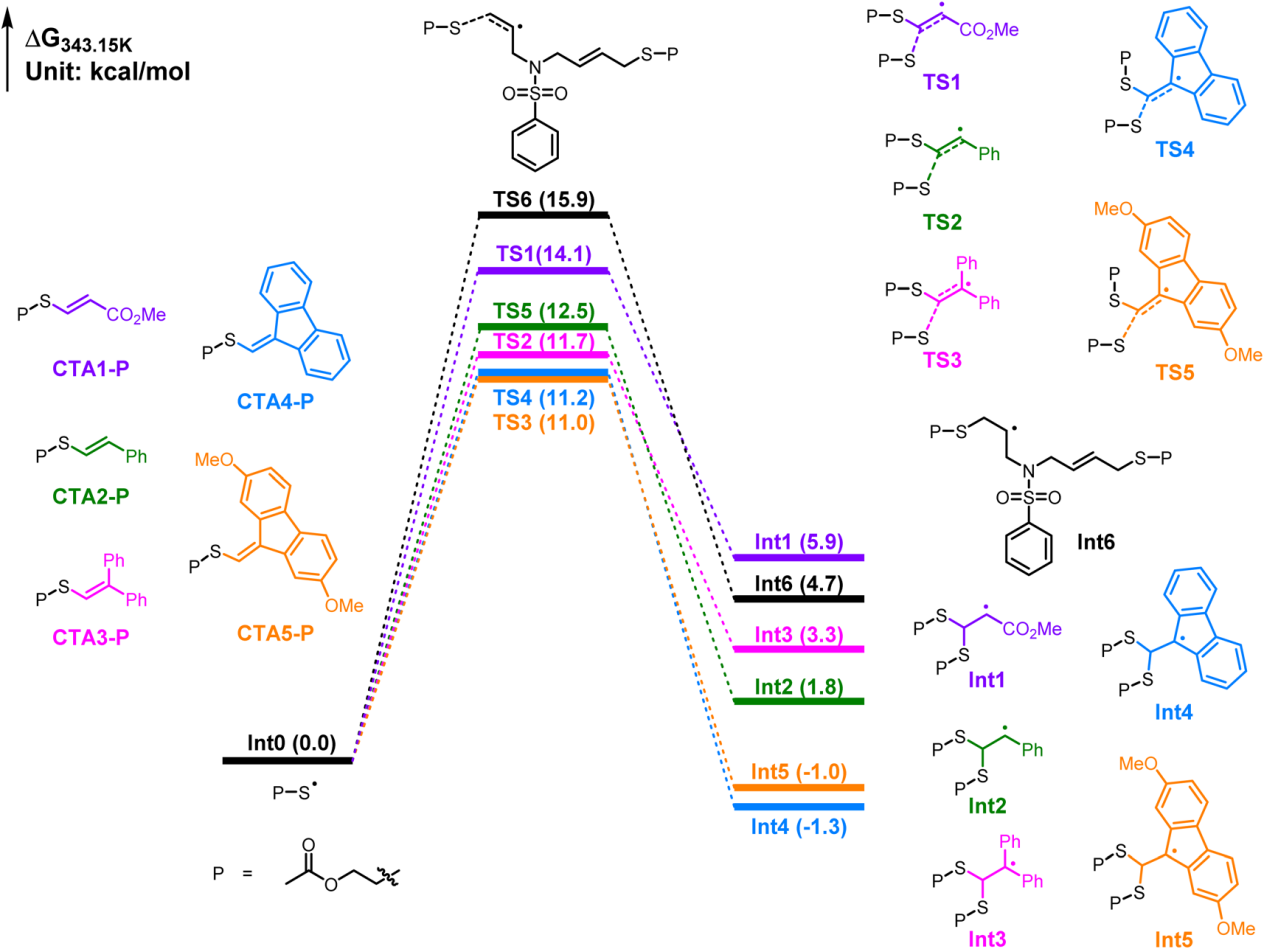
Calculated free energy profiles for the chain propagation process, with relative free energies reported in kcal mol^−1^.

To gain more insight into the control ability of vinyl sulfides, we performed DFT calculations at the PWPB95-D3(BJ)/def2-TZVPP/SMD(DMF)//M06-2X/def2-SVP/SMD(DMF) level of theory (Fig. 3).^40–46^ For clarity, the 2-acetoxyethane-1-thiyl radical and CTA-P were used as models for the propagating thiyl radical and the dormant CTA-capped polymer, respectively. The calculation results for the main equilibrium revealed that the energy barrier for the addition of the thiyl radical to the monomer was higher than that for its addition to each CTA-P, indicating that the latter is kinetically more favorable (TS6*vs.*TS1–TS5). Among the various CTAs, the fluorenyl-substituted CTA-Ps (CTA4-P and CTA5-P) exhibited lower addition energy barriers, allowing the thiyl radical addition more efficiently than the other CTA-Ps. Furthermore, the equilibrium constant (*K*) calculations (Table S9) showed that CTA4-P has a relatively higher *K* value than CTA5-P, thereby enabling a rapid equilibrium between the propagating radicals and dormant polymers, making it more effective in regulating polymerization.

Additionally, calculations for the pre-equilibrium indicated that the RS groups had minimal effects on the addition energy barrier of the original CTAs. However, the stability of the RS˙ leaving groups had a significant influence on the β-elimination energy barrier (Fig. S28). A more stable RS˙ leaving group corresponded to a lower energy barrier for β-elimination, but it resulted in a higher energy barrier for reinitiation involving the RS˙ leaving group. Consequently, the (3-ethoxy-3-oxopropyl)thio group exhibited better performance than other RS groups, likely due to a balance between these two opposing effects.^47–49^ Overall, the calculated results agreed with the experimental observations, providing valuable insights and guidelines for the further design of new SRDP agents.

**Fig. 4:**
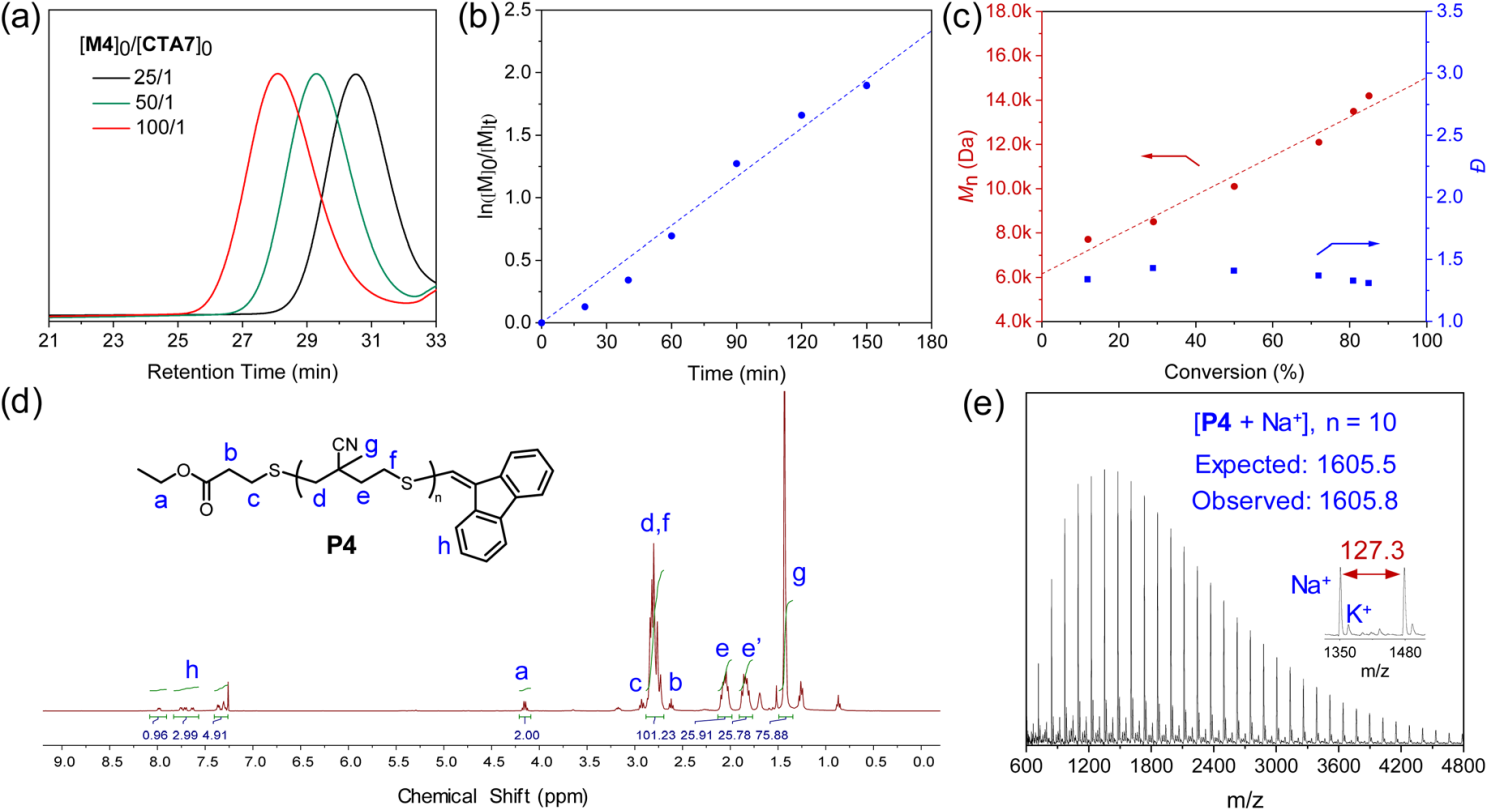
(a) SEC traces of polymers obtained from the polymerization of M4 at varying [M]_0_/[CTA]_0_ ratios (Table 3, entries 1–3). (b) Plot of ln([M]_0_/[M]_*t*_) *versus* reaction time for the polymerization of M4 at [M]_0_/[CTA]_0_ = 100/1. (c) Plots of *M*_n_ (red) and *Đ* (blue) *versus* monomer conversion for the polymerization of M4 under the same conditions. (d) ^1^H NMR (CDCl_3_, 25 °C) analysis of P4 (Table 3, entry 1). (e) MALDI-TOF analysis of P4 (Scheme S10).

With the SRDP successfully established, we then turn our attention to the radical transfer polymerization of thiocyanate alkenes, a unique thiyl radical polymerization documented by the Li and Sato groups.^10,11^ The polymerization of monomer M4 was first tested, which successfully yielded polymers P4 with *M*_n,SEC_ varying from 5.0 to 16.7 kDa and corresponding *Đ* values ranging from 1.24 to 1.33 (Table 3, entries 1–3 and Fig. 4a). The apparent chain transfer coefficient for the M4/CTA7 combination was measured to be 1.52 (Fig. S35).^50^ The controlled chain-growth nature of the polymerization was confirmed by a first-order kinetic behavior and a linear relationship between molecular weight and monomer conversion (Fig. 4b and c). Structural analysis of P4 by NMR spectroscopy and matrix-assisted laser desorption/ionization time-of-flight (MALDI-TOF) mass spectrometry revealed nearly quantitative preservation of the chain-end groups, further validating the excellent efficiency of vinyl sulfides in regulating the thiyl radical propagation process (Fig. 4d and e). Notably, in the MALDI-TOF spectra, the peaks corresponding to polymers initiated by AIBN are observed in only small quantities (Fig. S40). This outcome is likely because the carbon-centered radicals derived from AIBN have a relatively low initiating ability towards the less reactive vinyl groups, especially when compared to the thiyl radicals generated from the CTAs. Moreover, vinyl sulfides demonstrate relatively higher stability than allyl sulfides under AIBN conditions,^51^ making them a more suitable choice for preparing polymers that require nearly quantitative chain ends. Encouraged by these results, we then extended the SRDP strategy to the radical cascade polymerization of monomer M5, which involves a combination of radical closing polymerization and radical transfer polymerization. Like M4, the polymerization of M5 also produced polymers with controlled molecular weights and narrowed dispersities (Table 3, entries 4–6). Collectively, these findings underscore the potential of the SRDP strategy to polymerize a broad range of thiyl radical monomers, holding significant promise for applications in the synthesis of functional polymers with diverse architectures.

**Table 3:** Controlled radical transfer polymerization of thiocyanate alkenes

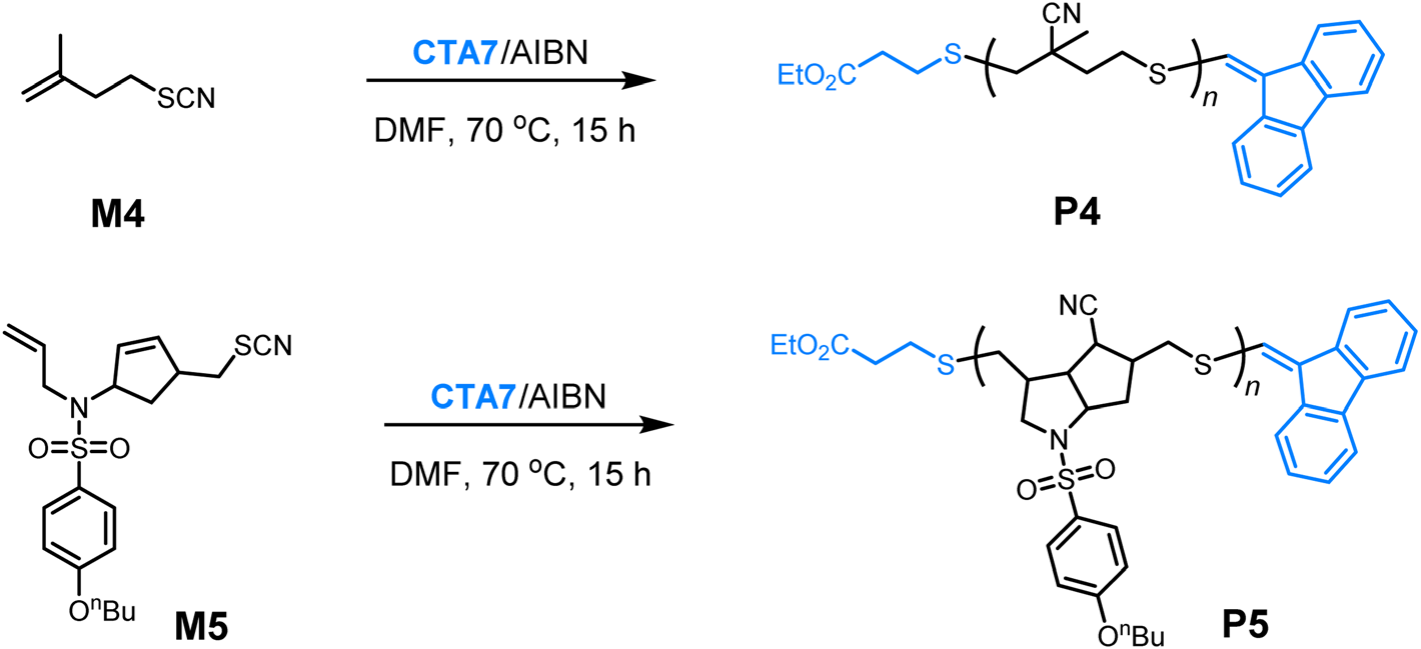							
Entry^a^	Monomer	[M]_0_/[CTA]_0_/[AIBN]_0_	Conversion^b^	*M*_n,theo_ c (kDa)	*M*_n,NMR_ d (kDa)	*M*_n,SEC_ e (kDa)	*Đ* e
1	M4	25/1/0.5	99%	3.5	3.6	5.0	1.24
2	M4	50/1/0.5	98%	6.5	7.4	9.4	1.26
3	M4	100/1/0.5	>99%	13.0	16.7	16.7	1.33
4	M5	25/1/0.8	57%	6.1	8.1	5.2	1.20
5	M5	50/1/0.8	52%	10.9	13.8	6.5	1.26
6	M5	100/1/0.8	49%	20.2	24.2	7.9	1.30

a Experimental conditions: [M4]_0_ = 0.8 M in entries 1–3 and [M5]_0_ = 0.2 M in entries 4–6, conducted at 70 °C under a nitrogen atmosphere unless otherwise specified.

b Determined by ^1^H NMR analysis of the crude reaction mixture.

c Calculated following the equation: *M*_n,theo_ = [M]_0_/[CTA]_0_ × MW^M^ × conversion + MW^CTA^, where MW^M^ and MW^CTA^ correspond to the molar mass of the monomer and CTA, respectively.

d Determined by ^1^H NMR analysis of the isolated polymers.

e Molecular weight and dispersity were determined by SEC in THF at 40 °C using polystyrene standards.

**Fig. 5:**
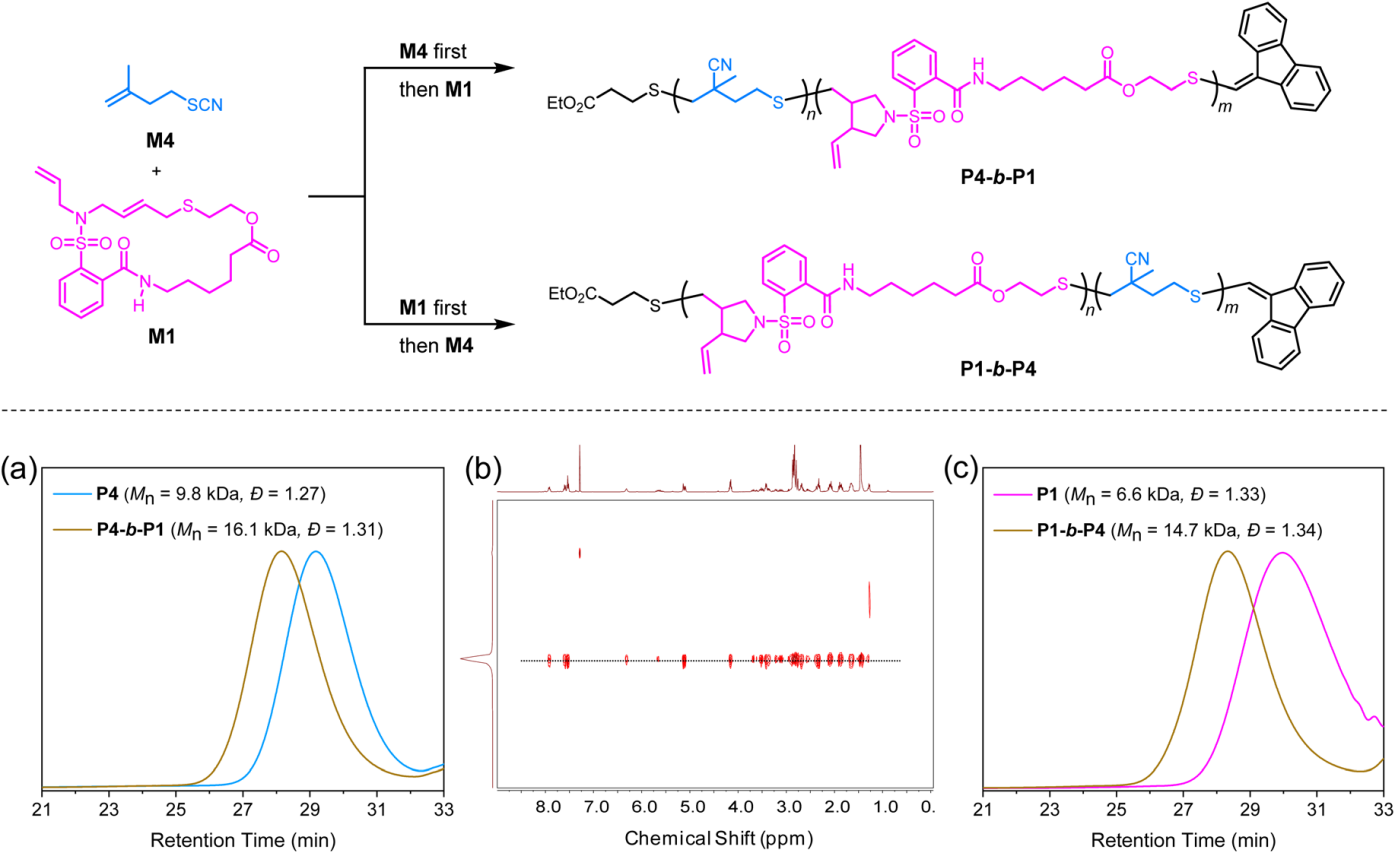
Synthesis and characterization of diblock copolymers derived from M1 and M4. (a) SEC traces for the preparation of diblock copolymer P4-*b*-P1. (b) DOSY NMR (CDCl_3_, 25 °C) spectrum of P4-*b*-P1. (c) SEC traces for the preparation of diblock copolymer P1-*b*-P4.

To further demonstrate the versatility of SRDP, we then explored the possibility of copolymerizing cyclic allylic sulfides with thiocyanate alkenes to synthesize diblock copolymers (Fig. 5). To this end, the polymerization of monomer M4 was first carried out, resulting in the formation of the first block with *M*_n,SEC_ of 9.8 kDa and *Đ* of 1.27. Subsequently, monomer M1 was used for chain extension, yielding the diblock copolymer P4-*b*-P1 with *M*_n,SEC_ of 16.1 kDa and *Đ* of 1.31 (Fig. 5a). The successful synthesis of the diblock copolymer was further validated by diffusion-ordered spectroscopy (DOSY), which showed a single diffusion peak in the spectrum (Fig. 5b). Additionally, we could use P1 as the first block and still achieve a successful block copolymerization with M4, resulting in the formation of the diblock copolymer, P1-*b*-P4, with *M*_n,SEC_ of 14.7 kDa and *Đ* of 1.34 (Fig. 5c). These results demonstrate the high fidelity of the resultant polymers and highlight the flexibility of the SRDP strategy in achieving successful block copolymerizations with different monomer combinations.

**Fig. 6:**
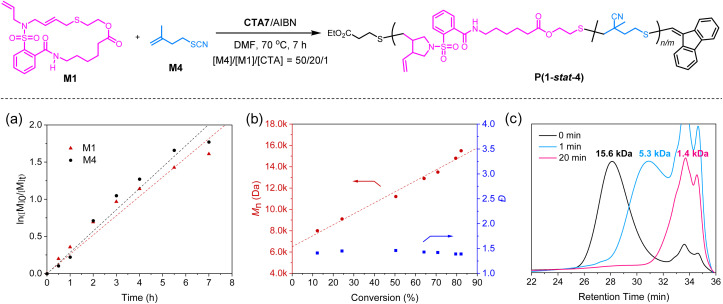
Synthesis and characterization of statistical copolymer P(1-*stat*-4). (a) Plot of ln([M]_0_/[M]_*t*_) *versus* reaction time. (b) Plots of *M*_n_ (red) and *Đ* (blue) *versus* monomer conversion. (c) SEC traces for the degradation of copolymer P(1-*stat*-4).

After successfully synthesizing the diblock copolymers, we shifted our focus to the statistical copolymerization of monomers M1 and M4. First, copolymerization was carried out using a feed ratio of [M4] : [M1] : [CTA7] = 50 : 20 : 1, resulting in the formation of the statistical copolymer P(1-*stat*-4) with *M*_n,SEC_ of 15.6 kDa and *Đ* of 1.39. The successful formation of the statistical copolymer was further confirmed by a single diffusion peak in the DOSY spectrum (Fig. S47). The kinetic analysis of the statistical copolymerization using ^1^H NMR indicated that both M1 and M4 exhibited first-order kinetics during the copolymerization process (Fig. 6a). Their observed rate constants were calculated for M1 and M4 to be *k*_M1_ = 0.23 h^−1^ and *k*_M4_ = 0.28 h^−1^, respectively. Moreover, the molecular weight of P(1-*stat*-4) increased linearly with the total conversion of M1 and M4, while maintaining low *Đ* throughout the polymerization process, indicative of a well-controlled copolymerization (Fig. 6b). Notably, this is the first example of controlled statistical copolymerization involving two thiyl radical monomers that exhibit different reaction mechanisms. Given the presence of ester linkages in the polymer backbone, we hypothesized that these copolymers could be susceptible to degradation under alkaline conditions. To test this hypothesis, we investigated the degradation reactivity of the copolymers by treating them with sodium methoxide. SEC analysis showed a dramatic reduction in the molecular weight of P(1-*stat*-4) within just 1 minute, with the degradation reaction reaching completion in approximately 20 minutes (Fig. 6c). These findings further underscore the promise of the SRDP method for creating functional, degradable materials with tailored properties.

## Conclusions

In summary, we have established a novel SRDP strategy by employing vinyl sulfides as effective chain transfer agents capable of reversibly deactivating the propagating thiyl radicals. This approach enables precise control over thiyl radical polymerizations, yielding polymers with tunable molecular weights, relatively low dispersities, and well-defined structures. Mechanistic investigations provided valuable insight into the reversible deactivation behavior of vinyl sulfides, further validating their role in achieving controlled polymerization. Furthermore, the versatility of the SRDP method has been demonstrated through its successful application in controlling a wide range of thiyl radical polymerizations, including the radical ring-opening polymerization of cyclic allylic sulfides and the radical transfer polymerization of thiocyanate alkenes, thereby allowing for the construction of diverse polymers with tailored main-chain functionalities. Notably, this research also marks the first reversible exchange reaction of vinyl sulfides through a radical mechanism. Altogether, our work not only offers a powerful platform for advancing controlled thiyl radical polymerizations but also opens new avenues for the application of the dynamic chemistry of vinyl sulfides in polymer synthesis. Further investigations into these potential applications are currently ongoing in our laboratory.

## Author contributions

H. Hu and P. Yi contributed equally to this work by conducting the experiments and characterizations. D. Cao provided technical support. H. Huang conceived the idea, supervised the project, and wrote the manuscript. All authors have given approval to the final version of the manuscript.

## Conflicts of interest

There are no conflicts to declare.

## Supplementary Material

SC-016-D5SC06492A-s001

## Data Availability

The data supporting this article have been included as part of the supplementary information (SI). Supplementary information: synthetic experiments, optimization tables, computational details, characterization data, and spectra. See DOI: https://doi.org/10.1039/d5sc06492a.
