# Supplementary material for: Thiyl radical reversible-deactivation polymerization via degenerative transfer with vinyl sulfides
Source: Chem Sci. 2025 Oct 31;16(48):23253–61. doi: 10.1039/d5sc06492a (PMC12588356; doi:10.1039/d5sc06492a)
Supplement: SC-016-D5SC06492A-s001 [file SC-016-D5SC06492A-s001.pdf]

Supplementary Information (SI) for Chemical Science.
This journal is © The Royal Society of Chemistry 2025

Supporting Information

Thiyl Radical Reversible-Deactivation Polymerization via Degenerative Transfer with Vinyl Sulfides

Huajuan Hu,[†ab] Ping Yi,[†ab] Derong Cao [a] and Hanchu Huang*[b]

[a] School of Chemistry and Chemical Engineering, State Key Laboratory of Luminescent Materials and Devices, South China University of Technology, Guangzhou 510640, China

[b] School of Materials Science and Engineering, Key Laboratory for Polymeric Composite and Functional Materials of Ministry of Education, Sun Yat-Sen University, Guangzhou 510006, China

[†] These authors contributed equally to this work.

***Corresponding Email**: huanghch9@mail.sysu.edu.cn

Table of Contents

General Information S5

Experimental Procedures S6

Scheme S1. Synthesis of chain transfer agents. S6

Scheme S2. Synthesis of monomers. S11

Scheme S3. Polymerization of different monomers. S15

Scheme S4. Radical reaction of polymers **P1** with different thiols. S18

Figure S1. ^{1}H NMR ($CDCl_3$, 25°C) spectra of polymers resulted from the reaction of **P1** and thiols. S18

Figure S2. ^{1}H NMR ($CDCl_3$, 25°C) determination of monomer conversion for the polymerization of monomer **M1** (Table S1, entry 8). S19

Figure S3. ^{1}H NMR ($CDCl_3$, 25°C) determination of molecular weight of **P1** (Table S1, entry 8). S20

Table S1. Effect of different CTAs on the polymerization of monomer **M1**. S21

Figure S4. SEC traces for the polymerization of monomer **M1** with different CTAs (Table S1, entries 1–10). S22

Figure S5. ^{1}H NMR ($CDCl_3$, 25°C) spectra of the resulting polymers in table S1. .... S22

Table S2. Effect of different solvents on the polymerization of monomer **M1**. S23

Figure S6. SEC traces for the polymerization of monomer **M1** in different solvents (Table S2, entries 1–5). S23

Table S3. Effect of different concentrations on the polymerization of monomer **M1**. S24

Figure S7. SEC traces for the polymerization of monomer **M1** at different concentrations (Table S3, entries 1–4). .. S24

Table S4. Effect of different amounts of AIBN on the polymerization of monomer **M1**. S25

Figure S8. SEC traces for the polymerization of monomer **M1** at different amounts of AIBN (Table S4, entries 1–5). S25

Table S5. Polymerization of monomer **M1** at different monomer/CTA ratios. S26

Figure S9. SEC traces for the polymerization of monomer **M1** at different monomer/CTA ratios (Table S5, entries 1–4). S26

Figure S10. ^{1}H NMR ($CDCl_3$, 25°C) determination of monomer conversion for the polymerization of monomer **M2** (Table S6, entry 2). S27

Figure S11. ^{1}H NMR ($CDCl_3$, 25°C) determination of molecular weight of **P2** (Table S6, entry 2). S28

Table S6. Polymerization of monomer **M2** at different monomer/CTA ratios. S29

Figure S12. SEC traces for the polymerization of monomer **M2** at different monomer/CTA ratios (Table S6, entries 1–3). S29

Figure S13. ^{1}H NMR ($CDCl_3$, 25°C) determination of monomer conversion for the polymerization of monomer **M3** (Table S7, entry 2). S30

Figure S14. ^{1}H NMR ($CDCl_3$, 25°C) determination of molecular weight of **P3** (Table S7, entry 1). S31

Table S7. Polymerization of monomer **M3** at different monomer/CTA ratios. S32

Figure S15. SEC traces for the polymerization of monomer **M3** at different monomer/CTA ratios (Table S7, entries 1–3).......... S32

Scheme S5. Kinetic study for the SRDP of **M1**.......... S33

Figure S16. (a) Plot of ln([M]$_0$/[M]$_t$) versus reaction time for the SRDP of **M1**. (b) Plots of M_n and $Đ$ versus monomer conversion for the SRDP of **M1**. S33

Figure S17. SEC traces for the kinetic study of the SRDP of **M1**. S33

Figure S18. ^{1}H NMR ($CDCl_3$, 25°C) traces for the kinetic study of the SRDP of **M1**. S34

Figure S19. MALDI-TOF analysis of **P1**.......... S35

Scheme S6. Synthesis of diblock copolymer **P1-*b*-P2**.......... S36

Figure S20. SEC traces for the synthesis of diblock copolymer **P1-*b*-P2**. S36

Figure S21. ^{1}H NMR ($CDCl_3$, 25°C) analysis of the diblock copolymer **P1-*b*-P2**. S37

DFT Calculations.......... S38

Figure S22. Proposed mechanism of the thiyl radical reversible-deactivation process.......... S38

Figure S23. Proposed mechanism for the chain-growth process of the polymerization of **M1**/**M2**. S39

Figure S24. Proposed mechanism for the chain-growth process of the polymerization of **M3**. S39

Figure S25. Proposed mechanism for the chain-growth process of the polymerization of **M4**. S40

Figure S26. Proposed mechanism for the chain-growth process of the polymerization of **M5**. S40

Scheme S7. Calculation of rate and equilibrium constants.......... S41

Table S8. Computed results for different CTA-Ps at the main equilibrium.......... S42

Figure S27. Calculated free energy profiles for different CTA-Ps at the main equilibrium.......... S43

Table S9. Calculated addition (k_{add}), elimination (k_{elim}) and equilibrium (K) constants for different CTA-Ps at the main equilibrium. S43

Table S10. Computed results for different original CTAs at the pre-equilibrium and reinitiation.......... S44

Figure S28. Calculated free energy profiles for different original CTAs at the pre-equilibrium. S45

Figure S29. ^{1}H NMR ($CDCl_3$, 25°C) determination of monomer conversion for the polymerization of monomer **M4** (Table S11, entry 2). S46

Figure S30. ^{1}H NMR ($CDCl_3$, 25°C) determination of molecular weight of **P4** (Table S11, entry 1). S47

Table S11. Polymerization of monomer **M4** at different monomer/CTA ratios. S48

Figure S31. SEC traces for the polymerization of monomer **M4** at different monomer/CTA ratios (Table S11, entries 1–3).......... S48

Figure S32. ^{1}H NMR ($CDCl_3$, 25°C) determination of monomer conversion for the polymerization of monomer **M5** (Table S12, entry 1). S49

Figure S33. ^{1}H NMR ($CDCl_3$, 25°C) determination of molecular weight of **P5** (Table S12, entry 1). S50

Table S12. Polymerization of monomer **M5** at different monomer/CTA ratios. S51

Figure S34. SEC traces for the polymerization of monomer **M5** at different monomer/CTA ratios (Table S12, entries 1–3).. S51

Scheme S8. Procedure for calculating the chain transfer coefficient of the **M4**/**CTA7** combination.......................... S52

Figure S35. Calculated chain transfer coefficient of the **M4**/**CTA7** combination. S52

Figure S36. ^{1}H NMR ($CDCl_3$, 25°C) traces for the calculated chain transfer coefficient of the **M4**/**CTA7** combination.S52

Scheme S9. Kinetic study for the SRDP of **M4**.. S53

Figure S37. (a) Plot of $\ln([M]_0/[M]_t)$ versus reaction time for the SRDP of **M4**. (b) Plots of M_n and $Đ$ versus monomer conversion for the SRDP of **M4**. S53

Figure S38. SEC traces for the kinetic study of the SRDP of **M4**. S53

Figure S39. ^{1}H NMR ($CDCl_3$, 25°C) traces for the kinetic study of the SRDP of **M4**... S54

Scheme S10. Polymer synthesis for MALDI-TOF analysis. S55

Figure S40. MALDI-TOF analysis of polymer **P4**. S55

Scheme S11. Synthesis of diblock copolymer **P4-*b*-P1**. S56

Figure S41. SEC traces for the synthesis of diblock copolymer **P4-*b*-P1**. S56

Figure S42. DOSY ($CDCl_3$, 25°C) analysis of diblock copolymer **P4-*b*-P1**. S57

Scheme S12. Synthesis of diblock copolymer **P1-*b*-P4**. S58

Figure S43. SEC traces for the synthesis of diblock copolymer **P1-*b*-P4** S58

Scheme S13. Kinetic study for the statistical copolymerization of **M1** and **M4**. S59

Figure S44. (a) Plot of $\ln([M]_0/[M]_t)$ versus reaction time for the statistical copolymerization of **M1** and **M4**. (b) Plots of M_n and $Đ$ versus monomer conversion for the statistical copolymerization of **M1** and **M4**....................................... S59

Figure S45. SEC traces for the kinetic study of the statistical copolymerization of **M1** and **M4**. S60

Figure S46. ^{1}H NMR ($CDCl_3$, 25°C) traces for the kinetic study of the statistical copolymerization of **M1** and **M4**...... S60

Figure S47. DOSY ($CDCl_3$, 25°C) analysis of the statistical copolymers **P(1-*stat*-4)** ($M_{n,SEC}$ = 15.6 kDa, $Đ$ =1.39). S61

Scheme S14. Degradation of the statistical copolymer **P(1-*stat*-4)** with sodium methoxide...................................... S62

Figure S48. SEC traces for degradation of statistical copolymer **P(1-*stat*-4)**.. S62

References S63

NMR Spectra S64

Cartesian Coordinates of All Optimized Structures.. S89

General Information

Materials. Unless otherwise noted, the organic solvents such as dimethylformamide (DMF), dimethyl sulfoxide (DMSO), tetrahydrofuran (THF), toluene, and dioxane were of analytical purity grade and used as received. Azobis(isobutyronitrile) (AIBN) was recrystallized from methanol before use. Other commercially available reagents were used without further purification. Thin layer chromatography (TLC) was performed using Huanghai TLC silica gel plates (SHGF254) and visualized using UV light.

Instruments. ^{1}H and ^{13}C NMR spectra were recorded on a Bruker AV400 FT-NMR spectrometer with $CDCl_3$ as solvents at room temperature, and the chemical shifts were given in ppm. The residual solvent signals were used as references ($CDCl_3$: δ_H = 7.26 ppm, δ_C = 77.16 ppm). Size exclusion chromatography (SEC) measurements were performed on Shimadzu high-performance SEC system HLC-8320SEC with an LC-20AD pump at 40 °C and a 0.6 mL/min flow rate. HPLC grade tetrahydrofuran (THF) was used as the eluent. Polystyrene standards (Shodex, SM-105) were used to determine the molecular weight and molecular weight distribution of polymers. The polymers were dissolved in the THF solution and filtered through a 0.45 μm PTFE filter before being injected into the SEC system. High-resolution mass spectrometry was performed on the Orbitrap Fusion Lumos system. Matrix-assisted laser desorption/ionization time-of-flight (MALDI-TOF) mass spectrometry was recorded on the Bruker UltrafleXtreme mass spectrometer. Fourier-transform infrared (FTIR) analysis was performed at Thermo Fisher Scientific NICOLET iS10.

Computational Study. Unless otherwise noted, all quantum chemical calculations were carried out with the Gaussian 16 computer program.[1] The geometry optimizations and frequency calculations were completed at the M06-2X/def2-SVP level with the solvation model based on density (SMD) in DMF.[2] All computed frequencies are real except the transition state structures, which have one imaginary frequency. The connectivity between each transition state and its two neighboring stationary points was confirmed by intrinsic reaction coordinate (IRC) calculations. Single-point energies were calculated at the PWPB95-D3(BJ)/def2-TZVPP level with the SMD in DMF,[3] and the calculations were carried out with the ORCA 6.0.1 computer program.[4] Thermal corrections were implemented with Shermo software[5] using a scale factor of 0.9762[6] and Grimme's quasi-harmonic oscillator approximation[7] at the solution standard state (T = 343.15 K, c = 1 mol/L).

Experimental Procedures

Scheme S1. Synthesis of chain transfer agents.

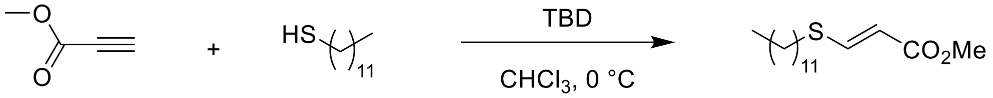

CTA1

A mixture of 1-dodecanethiol (4.8 mL, 20 mmol) and 1,5,7-triazabicyclo[4.4.0]dec-5-ene (TBD) (13.9 mg, 0.1 mmol) in $CHCl_3$ (2 mL), was added dropwise to a mixture of methyl propiolate (0.84 g, 10 mmol) in $CHCl_3$ (4 mL) while cooling in an ice bath. The mixture was stirred at 0 °C. After the reaction was complete, the solution was warmed to room temperature. The reaction mixture was washed with saturated $NaHCO_3$ solution (3×20 mL) and brine (20 mL). The organic phase was dried over Na_2SO_4 and concentrated *in vacuo*. The crude product was purified by column chromatography to afford **CTA1** as a yellowish solid (2.72 g, 95% yield). ^{1}H NMR (400 MHz, $CDCl_3$): δ 7.08 (d, *J* = 10.2 Hz, 1H), 5.90–5.67 (m, 1H), 3.76–3.69 (m, 3H), 2.82–2.68 (m, 2H), 1.71–1.60 (m, 2H), 1.44–1.34 (m, 2H), 1.34–1.17 (m, 16H), 0.87 (t, *J* = 6.8 Hz, 3H). ^{13}C NMR (100 MHz, $CDCl_3$): δ 167.2, 150.8, 147.4, 113.3, 112.6, 51.5, 51.3, 36.2, 32.2, 32.0, 30.4, 29.7, 29.7, 29.6, 29.6, 29.4, 29.3, 29.2, 28.9, 28.7, 28.6, 22.8, 14.2. IR (KBr, thin film): 2925, 2854, 1707, 1580, 1434, 1215, 1168, 800 cm^{-1}. MS (m/z): $[M+H]^+$ calc'd for $C_{16}H_{31}O_2S$, 287.2, found 287.0.

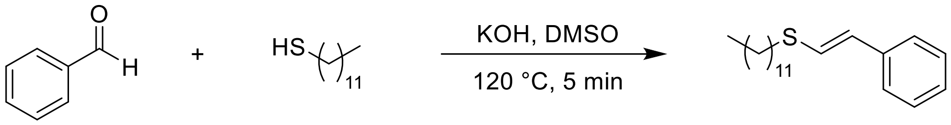

CTA2

Benzaldehyde (1.06 g, 10 mmol), KOH (2.24 g, 40 mmol),1-dodecanethiol (4.8 mL, 20 mmol), and DMSO (50 mL) were added to a 100 mL round-bottomed flask with a magnetic stir bar. The mixture was stirred at 120 °C under nitrogen for 5 minutes. After the reaction was complete, the solution was cooled to room temperature. The reaction mixture was diluted with water (100 mL) and then extracted with ethyl acetate (3×50 mL). The combined organic extracts were washed with brine (3×50 mL), dried over Na_2SO_4, and concentrated *in vacuo*. The crude product was purified by column chromatography to afford **CTA2** as a colorless oil (1.87 g, 61% field). ^{1}H NMR (400 MHz, $CDCl_3$): δ 7.39–7.26 (m, 3H), 7.24–7.15 (m, 1H), 6.74 (d, *J* = 15.6 Hz, 1H), 6.47 (d, *J* = 15.6 Hz, 1H), 2.85–2.68 (m, 2H), 1.76–1.56 (m, 2H), 1.49–1.8 (m, 2H), 1.38–1.19 (m, 16H), 0.90 (t, *J* = 6.8 Hz, 3H). ^{13}C NMR (100 MHz, $CDCl_3$): δ 137.2, 128.6, 126.7, 126.7, 125.4, 125.4, 32.7, 31.9, 29.7, 29.6, 29.6, 29.5, 29.5, 29.3, 29.2, 28.8, 22.7, 14.1. IR (KBr, thin film): 2924, 2853, 1598, 1465, 1446, 936, 736, 691 cm^{-1}. MS (m/z): $[M+H]^+$ calc'd for $C_{20}H_{33}S$, 305.2, found 305.0.

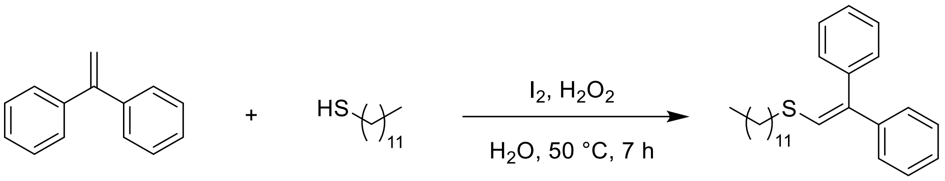

CTA3

A 50 mL reaction flask was charged with ethene-1,1-diyldibenzene (1.80 g, 10 mmol), iodine (0.51 g, 2 mmol), 1-dodecanethiol (2.4 mL, 10 mmol), water (6.0 mL) and hydrogen peroxide (510 μL, 5 mmol). The resulting suspension

was stirred at 50 °C for 7 hours. After the reaction was complete, the solution was quenched by saturated $Na_2S_2O_3$ (15 mL) and then extracted with ethyl acetate (3×50 mL). The combined organic extracts were washed with brine (3×50 mL), dried over Na_2SO_4, and concentrated *in vacuo*. The crude product was purified by column chromatography to afford **CTA3** as a yellowish oil (2.09 g, 55% field). ^{1}H NMR (400 MHz, $CDCl_3$): δ 7.44–7.37 (m, 2H), 7.37–7.27 (m, 5H), 7.27–7.20 (m, 3H), 6.62 (s, 1H), 2.78 (t, *J* = 7.4 Hz, 2H), 1.75–1.66 (m, 2H), 1.48–1.38 (m, 2H), 1.38–1.21 (m, 16H), 0.92 (t, *J* = 6.8 Hz, 3H). ^{13}C NMR (100 MHz, $CDCl_3$): δ 142.1, 139.7, 138.4, 129.8, 128.4, 128.3, 127.5, 127.1, 126.9, 126.5, 35.0, 32.0, 30.5, 29.8, 29.8, 29.7, 29.6, 29.5, 29.3, 28.8, 22.8, 14.3. IR (KBr, thin film): 2925, 2853, 1494, 1465, 1442, 770, 753, 697 cm^{-1}. MS (m/z): $[M+H]^+$ calc'd for $C_{26}H_{37}S$, 381.2, found 381.1.

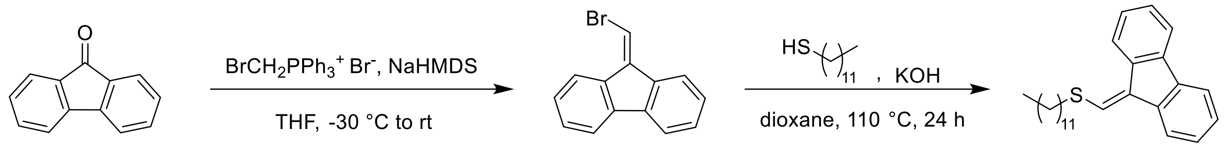

S1 **CTA4**

A dry 100 mL double round-bottomed flask equipped with a magnetic stirrer was charged with bromomethyltriphenyl phosphine bromide (4.54 g, 10.4 mmol) and anhydrous THF (30 mL). After cooling the solution to -30 °C in ice and salt bath, sodium bis(trimethylsilyl)amide (5.4 mL, 10.8 mmol) was added slowly under nitrogen for 40 minutes, a solution of 9H-fluoren-9-one (1.62 g, 9 mmol) in THF (10 mL) was slowly added and warmed to room temperature, the reaction was carried out at room temperature overnight. After the reaction was complete, the solution was quenched by H_2O (50 mL) and then extracted with ethyl acetate (3×50 mL). The combined organic extracts were washed with brine (3×50 mL), dried over Na_2SO_4, and concentrated *in vacuo*. The crude product was purified by column chromatography to afford **S1** as a yellowish solid (1.09 g, 47% yield, CAS: 4612-64-0). ^{1}H NMR (400 MHz, $CDCl_3$): δ 8.63–8.55 (m, 1H), 7.74–7.70 (m, 1H), 7.70–7.65 (m, 1H), 7.59–7.53 (m, 1H), 7.49–7.42 (m, 1H), 7.42–7.33 (m, 3H), 7.31–7.25 (m, 1H). ^{13}C NMR (100 MHz, $CDCl_3$): δ 141.5, 139.2, 138.9, 138.5, 136.7, 129.6, 128.8, 127.4, 127.3, 125.8, 120.3, 120.0, 119.9, 105.9.

A dry 50 mL round-bottomed flask equipped with a magnetic stirrer was charged with **S1** (1.03 g, 4 mmol), 1,4-dioxane (4 mL), KOH (0.45 g, 8 mmol) and 1-dodecanethiol (1.0 mL, 4 mmol). The resulting suspension was refluxed at 120 °C in oil bath for 24 hours. After the reaction was complete, the solution was cooled to room temperature, the reaction was quenched by H_2O (30 mL) and then extracted with ethyl acetate (3×50 mL). The combined organic extracts were washed with brine (3×50 mL), dried over Na_2SO_4, and concentrated *in vacuo*. The crude product was purified by column chromatography to afford **CTA4** as a white solid (0.70 g, 46% yield). ^{1}H NMR (400 MHz, $CDCl_3$): δ 8.12–8.04 (m, 1H), 7.83–7.71 (m, 2H), 7.66–7.59 (m, 1H), 7.42–7.35 (m, 3H), 7.35–7.27 (m, 2H), 3.02 (t, *J* = 7.4 Hz, 2H), 1.88–1.76 (m, 2H), 1.54–1.5 (m, 2H), 1.41–1.21 (m, 16H), 0.91 (t, *J* = 6.8 Hz, 3H). ^{13}C NMR (100 MHz, $CDCl_3$): δ 140.0, 138.5, 137.9, 137.2, 131.6, 128.4, 127.3, 127.0, 126.8, 126.8, 125.3, 119.8, 119.7, 119.1, 36.5, 32.1, 30.6, 29.8, 29.8, 29.7, 29.6, 29.5, 29.3, 28.7, 22.8, 14.3. IR (KBr, thin film): 2924, 2853, 1586, 1448, 844, 777, 738, 726 cm^{-1}. MS (m/z): $[M+H]^+$ calc'd for $C_{26}H_{35}S$, 379.2, found 379.0.

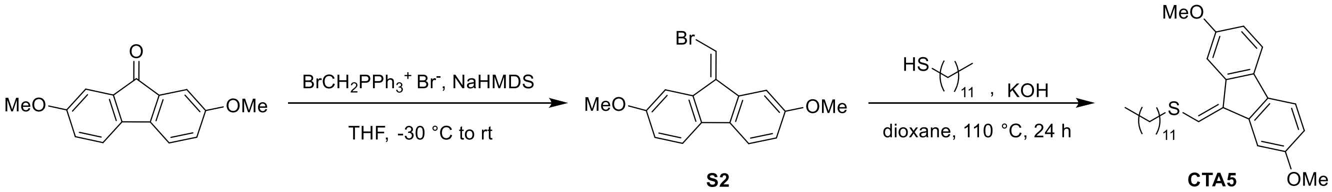

A dry 100 mL double round-bottomed flask equipped with a magnetic stirrer was charged with bromomethyltriphenyl phosphine bromide (4.54 g, 10.4 mmol) and anhydrous THF (30 mL). After cooling the solution to -30 °C in ice and salt bath, sodium bis(trimethylsilyl)amide (5.4 mL, 10.8 mmol) was added slowly under nitrogen for 40 minutes, a solution of 2,7-dimethoxy-9H-fluoren-9-one (2.16 g, 9 mmol) in THF (10 mL) was slowly added and warmed to room temperature, the reaction was carried out at room temperature overnight. After the reaction was complete, the solution was quenched by H_2O (50 mL) and then extracted with ethyl acetate (3×50 mL). The combined organic extracts were washed with brine (3×50 mL), dried over Na_2SO_4, and concentrated *in vacuo*. The crude product was purified by column chromatography to afford **S2** as a yellow solid (1.63 g, 57% yield). ^{1}H NMR (400 MHz, $CDCl_3$): δ 8.11 (d, *J* = 2.4 Hz, 1H), 7.46 (d, *J* = 8.3 Hz, 1H), 7.43 (d, *J* = 8.3 Hz, 1H), 7.31 (s, 1H), 7.03 (d, *J* = 2.3 Hz, 1H), 6.94 (dd, *J* = 8.3, 2.4 Hz, 1H), 6.87 (dd, *J* = 8.3, 2.3 Hz, 1H), 3.88 (s, 3H), 3.85 (s, 3H). ^{13}C NMR (100 MHz, $CDCl_3$): δ 158.9, 158.6, 139.8, 139.2, 138.0, 134.8, 132.3, 119.7, 115.1, 114.6, 111.8, 106.1, 106.0, 55.8, 55.7. IR (KBr, thin film): 2954, 2924, 2851, 1604, 1579, 1463, 1432, 1296, 1219, 1184, 1155, 1115, 1044, 1000, 804, 787, 717 cm^{-1}. MS (m/z): $[M]^{\cdot+}$ calc'd for $C_{16}H_{13}BrO_2$, 316.0, found 316.0.

A dry 50 mL round-bottomed flask equipped with a magnetic stirrer was charged with **S2** (1.27 g, 4 mmol), 1,4-dioxane (4 mL), KOH (0.45 g, 8 mmol) and 1-dodecanethiol (1.0 mL, 4 mmol). The resulting suspension was refluxed at 120 °C in oil bath for 24 hours. After the reaction was complete, the solution was cooled to room temperature, the reaction was quenched by H_2O (30 mL) and then extracted with ethyl acetate (3×50 mL). The combined organic extracts were washed with brine (3×50 mL), dried over Na_2SO_4, and concentrated *in vacuo*. The crude product was purified by column chromatography to afforded **CTA5** as a yellowish solid (0.89 g, 51% yield). ^{1}H NMR (400 MHz, $CDCl_3$): δ 7.60 (d, *J* = 2.3 Hz, 1H), 7.54 (d, *J* = 8.3 Hz, 1H), 7.50 (d, *J* = 8.2 Hz, 1H), 7.31 (s, 1H), 7.10 (d, *J* = 2.2 Hz, 1H), 6.89 (dd, *J* = 8.3, 2.4 Hz, 1H), 6.84 (dd, *J* = 8.3, 2.3 Hz, 1H), 3.91 (s, 3H), 3.88 (s, 3H), 3.00 (t, *J* = 7.4 Hz, 2H), 1.86–1.75 (m, 2H), 1.53–1.43 (m, 2H), 1.38–1.25 (m, 16H), 0.91 (t, *J* = 6.7 Hz, 3H). ^{13}C NMR (100 MHz, $CDCl_3$): δ 158.7, 158.6, 139.9, 138.6, 133.4, 131.6, 131.5, 128.4, 119.7, 119.5, 113.1, 112.8, 111.2, 104.9, 55.7, 36.5, 32.0, 30.6, 29.8, 29.8, 29.7, 29.6, 29.5, 29.3, 28.7, 22.8, 14.3. IR (KBr, thin film): 2925, 2853,1608, 1585, 1466, 1434, 1305, 1271, 1240, 1221, 1159, 1120, 1046, 838, 805, 768 cm^{-1}. MS (m/z): $[M+H]^+$ calc'd for $C_{28}H_{39}O_2S$, 439.3, found 439.1.

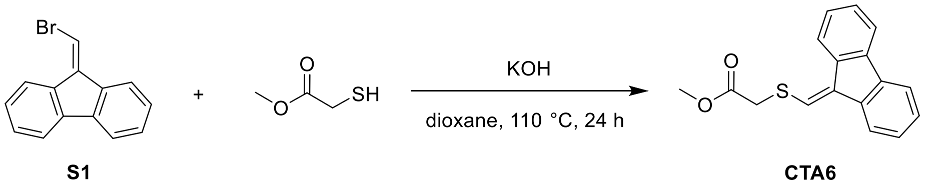

A dry 50 mL round-bottomed flask equipped with a magnetic stirrer was charged with **S1** (1.03 g, 4 mmol), 1,4-dioxane (4 mL), KOH (0.45 g, 8 mmol) and methyl thioglycolate (360 µL, 4 mmol). The resulting suspension was refluxed at 120 °C in oil bath for 24 hours. After the reaction was complete, the solution was cooled to room temperature, the reaction was quenched by H_2O (30 mL) and then extracted with ethyl acetate (3×50 mL). The combined organic extracts

were washed with brine (3×50 mL), dried over Na_2SO_4, and concentrated *in vacuo*. The crude product was purified by column chromatography to afford **CTA6** as a yellowish oil (0.46 g, 41% yield). ^1H NMR (400 MHz, $CDCl_3$): δ 8.05–7.96 (m, 1H), 7.78–7.73 (m, 1H), 7.73–7.69 (m, 1H), 7.65–7.60 (m, 1H), 7.42–7.26 (m, 5H), 3.80 (s, 3H), 3.70 (s, 2H). ^{13}C NMR (100 MHz, $CDCl_3$): δ 169.6, 140.4, 138.3, 138.2, 136.8, 133.1, 127.8, 127.4, 127.1, 127.0, 125.5, 124.8, 119.9, 119.8, 119.6, 53.0, 36.6. IR (KBr, thin film): 3059, 3008, 2951, 1732, 1611, 1588, 1477, 1350, 1434, 1407, 1337, 1284, 1220, 1196, 1162, 1006, 935, 841, 820, 772, 726, 640, 618 cm^{-1}. MS (m/z): $[M+H]^+$ calc'd for $C_{17}H_{15}O_2S$, 283.1, found 282.9.

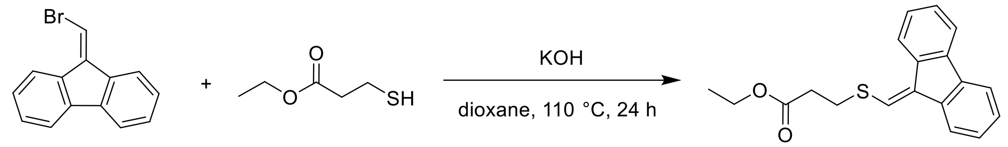

S1 **CTA7**

A dry 50 mL round-bottomed flask equipped with a magnetic stirrer was charged with **S1** (1.03 g, 4 mmol), 1,4-dioxane (4 mL), KOH (0.45 g, 8 mmol) and ethyl 3-sulfanylpropanoate (510 µL, 4 mmol). The resulting suspension was refluxed at 120 °C in oil bath for 24 hours. After the reaction was complete, the solution was cooled to room temperature, the reaction was quenched by H_2O (30 mL) and then extracted with ethyl acetate (3×50 mL). The combined organic extracts were washed with brine (3×50 mL), dried over Na_2SO_4, and concentrated *in vacuo*. The crude product was purified by column chromatography to afford **CTA7** as a yellowish solid (0.55 g, 44% yield). ^1H NMR (400 MHz, $CDCl_3$): δ 8.09–8.01 (m, 1H), 7.81–7.71 (m, 2H), 7.66–7.60 (m, 1H), 7.43–7.27 (m, 5H), 4.20 (q, *J* = 7.1 Hz, 2H), 3.28 (t, *J* = 7.2 Hz, 2H), 2.82 (t, *J* = 7.2 Hz, 2H), 1.28 (t, *J* = 7.1 Hz, 3H). ^{13}C NMR (100 MHz, $CDCl_3$): δ 171.4, 140.1, 138.3, 138.0, 136.9, 132.4, 127.5, 127.0, 127.0, 126.9, 126.8, 125.3, 119.7, 119.2, 61.0, 35.6, 31.1, 14.2. IR (KBr, thin film): 2980, 2922, 2851, 1731, 1610, 1586, 1448, 1373, 1348, 1288, 1265, 1244, 1219, 1182, 1155, 1033, 921, 841, 772, 728, 703, 618 cm^{-1}. MS (m/z): $[M+H]^+$ calc'd for $C_{19}H_{19}O_2S$, 311.1, found 310.8.

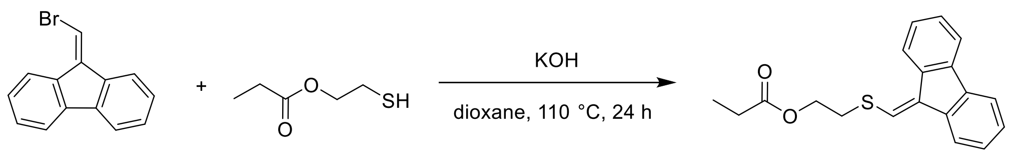

S1 **CTA8**

A dry 50 mL round-bottomed flask equipped with a magnetic stirrer was charged with **S1** (1.03 g, 4 mmol), 1,4-dioxane (4 mL), KOH (0.45 g, 8 mmol) and 2-mercaptoethyl propionate (0.48 mg, 4 mmol). The resulting suspension was refluxed at 120 °C in oil bath for 24 hours. After the reaction was complete, the solution was cooled to room temperature, the reaction was quenched by H_2O (30 mL) and then extracted with ethyl acetate (3×50 mL). The combined organic extracts were washed with brine (3×50 mL), dried over Na_2SO_4, and concentrated *in vacuo*. The crude product was purified by column chromatography to afford **CTA8** as a yellowish solid (0.43 g, 35% yield). ^1H NMR (400 MHz, $CDCl_3$): δ 8.08–8.02 (m, 1H), 7.81–7.75 (m, 1H), 7.75–7.71 (m, 1H), 7.64–7.60 (m, 1H), 7.43–7.37 (m, 2H), 7.37–7.27 (m, 3H), 4.41 (t, *J* = 6.6 Hz, 2H), 3.23 (t, *J* = 6.7 Hz, 2H), 2.38 (q, *J* = 7.5 Hz, 2H), 1.16 (t, *J* = 7.5 Hz, 3H). ^{13}C NMR (100 MHz, $CDCl_3$): δ 174.2, 140.1, 138.2, 138.0, 136.9, 132.5, 127.6, 127.1, 127.0, 126.9, 126.7, 125.3, 119.8, 119.2, 63.5, 34.5, 27.5, 9.1. IR (KBr, thin film): 2980, 2940, 1736, 1610, 1587, 1448, 1381, 1348, 1291, 1269, 1176, 1083, 1014, 922, 842, 770, 741, 728, 637, 618 cm^{-1}. MS (m/z): [M+H]+ calc'd for $C_{19}H_{19}O_2S$, 311.1, found 310.7.

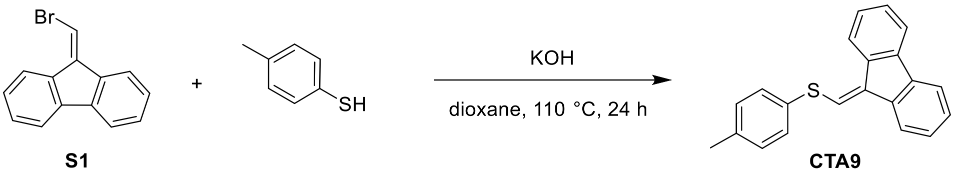

A dry 50 mL round-bottomed flask equipped with a magnetic stirrer was charged with **S1** (1.03 g, 4 mmol), 1,4-dioxane (4 mL), KOH (0.45 g, 8 mmol) and *p*-thiocresol (0.50 mg, 4 mmol). The resulting suspension was refluxed at 120 °C in oil bath for 24 hours. After the reaction was complete, the solution was cooled to room temperature, the reaction was quenched by H_2O (30 mL) and then extracted with ethyl acetate (3×50 mL). The combined organic extracts were washed with brine (3×50 mL), dried over Na_2SO_4, and concentrated *in vacuo*. The crude product was purified by column chromatography to afford **CTA9** as a yellowish solid (0.72 g, 60% yield). ^{1}H NMR (400 MHz, $CDCl_3$): δ 8.13–8.06 (m, 1H), 7.74–7.68 (m, 1H), 7.65 (d, *J* = 7.4 Hz, 1H), 7.49 (d, *J* = 7.5 Hz, 1H), 7.47–7.38 (m, 3H), 7.38–7.29 (m, 2H), 7.27–7.21 (m, 1H), 7.21–7.11 (m, 3H), 2.31 (s, 3H). ^{13}C NMR (100 MHz, $CDCl_3$): δ 140.2, 138.5, 138.4, 138.3, 136.9, 132.3, 131.9, 131.2, 130.3, 127.7, 127.5, 127.2, 127.1, 126.9, 125.3, 119.8, 119.8, 119.5, 21.3. IR (KBr, thin film): 3058, 2921, 1611, 1585, 1490, 1447, 1441, 1349, 1218, 1093, 1017, 924, 838, 807, 768, 740, 726, 647, 618, 490 cm^{-1}. MS (m/z): $[M+H]^+$ calc'd for $C_{21}H_{17}S$, 301.1, found 300.9.

Scheme S2. Synthesis of monomers.

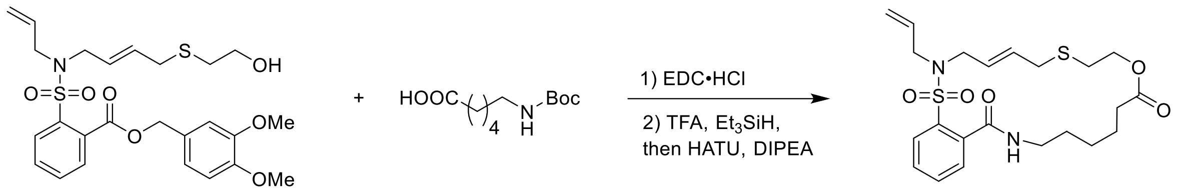

S3 M1

In a 250 mL flask, **S3** (5.21 g, 10 mmol, prepared according to the procedure reported by our group[8]) and Boc-6-aminocaproic acid (3.47 g, 15 mmol) were dissolved in dry DCM (50 mL). To this solution were added *N*-ethyl-*N'*-(3-dimethylaminopropyl)carbodiimide hydrochloride (EDC•HCl, 1.5 equiv.) and 4-dimethylaminopyridine (DMAP, 0.05 equiv.). After stirring at room temperature for 14 h, the reaction mixture was diluted with DCM (100 mL), washed with 1 M HCl (2×50 mL) and brine (50 mL), dried by Na_2SO_4, filtered, and concentrated *in vacuo* to afford a yellow oil, which was then directly subjected to the subsequent reaction. The yellow oil (1.0 equiv.) was dissolved in DCM (50 mL). To this solution, triethylsilane (10 equiv.) was added via a syringe, followed by the addition of trifluoroacetic acid (TFA, 13.0 equiv.). After stirring at room temperature for 2 h, the reaction mixture was diluted with toluene (50 mL) and concentrated *in vacuo* to give the intermediated amino acid, which was directly dissolved in DMF (1500 mL). 1-[Bis(dimethylamino)methylene]-1*H*-1,2,3-triazolo[4,5-*b*]pyridinium 3-oxide hexafluorophosphate (HATU, 2.0 equiv.) was then added to the reaction mixture at room temperature. The mixture was stirred for 1 h, and diisopropylethylamine (DIPEA, 5.0 equiv.) was slowly added. After stirring for an additional 36 h at room temperature, the reaction mixture was concentrated *in vacuo* and diluted with ethyl acetate (150 mL). The organic layer was washed with 1 M HCl (3×50 mL), 0.5 M NaOH (2×50 mL), and brine (50 mL) and dried over Na_2SO_4. The filtrate was concentrated and purified by column chromatography to afford monomer **M1** as a white solid (2.20 g, 47 % yield). ^{1}H NMR (400 MHz, $CDCl_3$): δ 7.93–7.88 (m, 1H), 7.60–7.54 (m, 1H), 7.54–7.46 (m, 2H), 6.21 (t, *J* = 5.8 Hz, 1H), 5.70–5.48 (m, 3H), 5.18–5.10 (m, 2H), 4.16 (t, *J* = 7.2 Hz, 2H), 3.88 (d, *J* = 4.5 Hz, 2H), 3.81 (d, *J* = 6.3 Hz, 2H), 3.43 (q, *J* = 6.4 Hz, 2H), 3.12 (d, *J* = 5.5 Hz, 2H), 2.66 (t, *J* = 7.2 Hz, 2H), 2.37 (t, *J* = 6.7 Hz, 2H), 1.78–1.59 (m, 4H), 1.53–1.44 (m, 2H). ^{13}C NMR (100 MHz, $CDCl_3$): δ 173.5, 168.1, 137.5, 136.6, 132.7, 132.6, 129.9, 129.7, 129.7, 129.2, 128.8, 119.3, 64.0, 49.6, 47.7, 39.9, 33.9, 33.4, 28.6, 28.1, 25.8, 24.2.

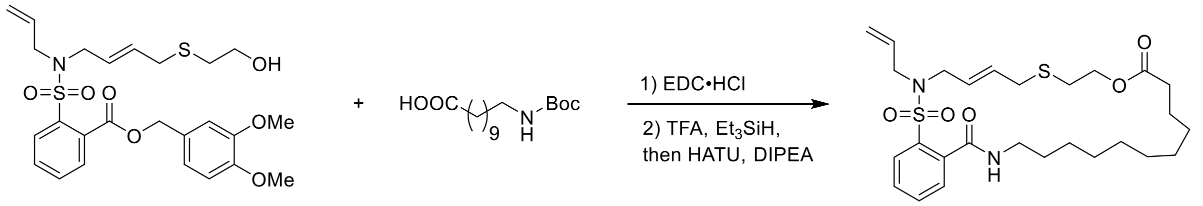

S3 M2

In a 250 mL flask, **S3** (2.61 g, 5 mmol) and Boc-11-aminoundecanoic acid (2.26 g, 7.5 mmol) were dissolved in dry DCM (50 mL). To this solution were added *N*-ethyl-*N'*-(3-dimethylaminopropyl)carbodiimide hydrochloride (EDC•HCl, 1.5 equiv.) and 4-dimethylaminopyridine (DMAP, 0.05 equiv.). After stirring at room temperature for 14 h, the reaction mixture was diluted with DCM (100 mL), washed with 1 M HCl (2×50 mL) and brine (50 mL), dried by Na_2SO_4, filtered, and concentrated *in vacuo* to afford a yellow oil, which was then directly subjected to the subsequent reaction. The

yellow oil (1.0 equiv.) was dissolved in DCM (50 mL). To this solution, triethylsilane (10 equiv.) was added via a syringe, followed by the addition of trifluoroacetic acid (TFA, 13.0 equiv.). After stirring at room temperature for 2 h, the reaction mixture was diluted with toluene (50 mL) and concentrated *in vacuo* to give the intermediated amino acid, which was directly dissolved in DMF (1500 mL). 1-[Bis(dimethylamino)methylene]-1*H*-1,2,3-triazolo[4,5-*b*]pyridinium 3-oxide hexafluorophosphate (HATU, 2.0 equiv.) was then added to the reaction mixture at room temperature. The mixture was stirred for 1 h, and diisopropylethylamine (DIPEA, 5.0 equiv.) was slowly added. After stirring for an additional 36 h at room temperature, the reaction mixture was concentrated *in vacuo* and diluted with ethyl acetate (150 mL). The organic layer was washed with 1 M HCl (3×50 mL), 0.5 M NaOH (2×50 mL), and brine (50 mL) and dried over Na_2SO_4. The filtrate was concentrated and purified by column chromatography to afford monomer **M2** as a white solid (1.17 g, 44 % yield). ^{1}H NMR (400 MHz, $CDCl_3$): δ 7.94–7.90 (m, 1H), 7.60–7.46 (m, 3H), 6.26 (t, *J* = 5.6 Hz, 1H), 5.67–5.49 (m, 3H), 5.17–5.09 (m, 2H), 4.18 (t, *J* = 6.9 Hz, 2H), 3.85–3.77 (m, 4H), 3.40 (q, *J* = 6.6 Hz, 2H), 3.18–3.10 (m, 2H), 2.65 (t, *J* = 6.9 Hz, 2H), 2.31 (t, *J* = 7.2 Hz, 2H), 1.68–1.56 (m, 4H), 1.46–1.36 (m, 2H), 1.36-1.25 (m, 10H). ^{13}C NMR (100 MHz, $CDCl_3$): δ 173.6, 167.9, 137.1, 136.6, 132.8, 132.6, 130.9, 130.0, 129.6, 129.4, 128.3, 119.4, 63.9, 49.1, 48.0, 40.3, 34.2, 33.6, 28.7, 28.6, 28.5, 28.5, 28.4, 28.3, 26.2, 24.8.

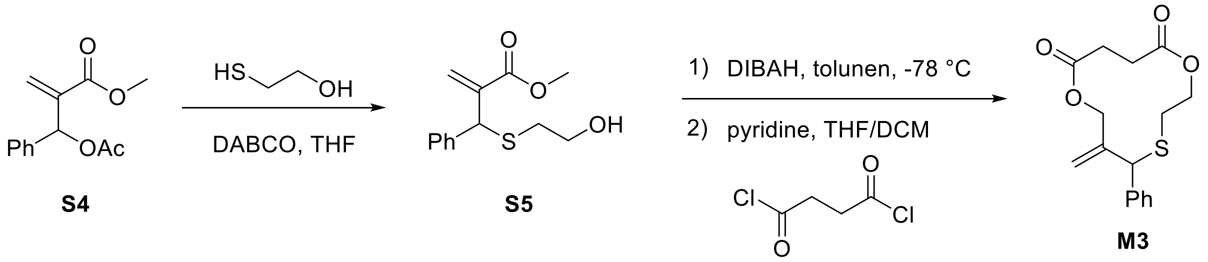

In a 250 mL flask, **S4** (7.03 g, 30 mmol, prepared according to the procedure reported by our group[9]) was dissolved in THF (30 mL), to which was slowly added 1,4-diazabicyclo[2.2.2]octane (DABCO) (4.04 g, 36 mmol) and a catalytic amount of H_2O (0.3 mL). After stirring at room temperature for 30 min, a 30 mL THF solution of 2-hydroxy-1-ethanethiol (2.1 mL, 13.2 mmol) was added. The reaction mixture continued to stir at room temperature for 30 min. Once the reaction was complete, it was quenched with H_2O (15 mL) and extracted with ethyl acetate (3×50 mL). The organic layer was washed with 1 M HCl (2×50 mL) and brine (30 mL), dried by Na_2SO_4, and concentrated *in vacuo*. The crude product was purified by column chromatography to afford **S5** as a colorless oil (4.54 g, 60% yield). ^{1}H NMR (400 MHz, $CDCl_3$): δ 7.41–7.35 (m, 2H), 7.32–7.26 (m, 2H), 7.25–7.19 (m, 1H), 6.41 (s, 1H), 5.92 (s, 1H), 5.11 (s, 1H), 3.75–3.60 (m, 5H), 2.91–2.80 (m, 1H), 2.66–2.52 (m, 2H). ^{13}C NMR (100 MHz, $CDCl_3$): δ 166.6, 140.4, 139.4, 128.5, 128.5, 127.5, 127.4, 60.6, 52.2, 48.6, 35.3. IR (KBr, thin film): 3434, 2952, 2923, 1720, 1626, 1492, 1452, 1438, 1387, 1316, 1293, 1253, 1201, 1133, 1047, 948, 845, 816, 751, 703, 652, 492, 446 cm^{-1}. MS (m/z): $[M+H]^+$ calc'd for $C_{13}H_{17}O_3S$, 253.1, found 252.8.

To a solution of **S5** (4.04 g,16 mmol) in dry DCM (30 mL) at -78 °C, the diisobutylaluminum hydride (1.5 M in toluene, 32.0 mL, 48 mmol) was added dropwise. After that, the reaction mixture was stirred at -78 °C for 6 hours until full consumption of the starting material. The reaction mixture was quenched carefully with HCl (1 M) and then extracted with DCM (3×30 mL). The organic layer was washed with brine (3×30 mL), dried by Na_2SO_4, filtered, and concentrated *in vacuo* to afford a colorless oil. This oil was dissolved in DCM (30 mL) and then transferred to a constant-pressure

drop funnel. Succinyl dichloride (1.7 mL, 16 mmol) was also dissolved in DCM (30 mL) and transferred to a separate constant-pressure drop funnel. The drop speeds of the two funnels were adjusted to keep them consistent, and the solutions were slowly added to a solution of pyridine (6.4 mL, 80 mmol) in DCM (200 mL). After the addition was complete, the solution was stirred for an additional 3 hours. The solvent was then removed under reduced pressure. The resulting brown pyridinium chloride salts were filtered. The supernatant was diluted with DCM (30 mL), washed twice with 1M HCl (2x50 mL), and then washed with brine (30 mL). The organic layer was dried over Na_2SO_4, concentrated, and purified by column chromatography, yielding **M3** as a yellowish oil (0.34 g, 7% yield). ^{1}H NMR (400 MHz, $CDCl_3$): δ 7.40–7.36 (m, 2H), 7.36–7.30 (m, 2H), 7.30–7.23 (m, 1H), 5.53 (s, 1H), 5.46 (s, 1H), 4.99 (s, 1H), 4.82 (d, *J* = 11.8 Hz, 1H), 4.73 (ddd, *J* = 11.7, 6.6, 2.1 Hz, 1H), 4.43 (d, *J* = 11.8 Hz, 1H), 4.28–4.18 (m, 1H), 2.91 (ddd, *J* = 15.8, 8.4, 2.2 Hz, 1H), 2.86–2.73 (m, 2H), 2.71–2.58 (m, 2H), 2.53 (ddd, *J* = 15.8, 6.7, 2.0 Hz, 1H). ^{13}C NMR (100 MHz, $CDCl_3$): δ 172.0, 171.0, 142.0, 138.9, 128.8, 128.7, 127.8, 119.3, 67.5, 66.6, 50.2, 30.7, 30.6, 30.6. IR (KBr, thin film): 3027, 2944, 1739, 1651, 1599, 1492, 1452, 1379, 1356, 1267, 1156, 1064, 1008, 929, 845, 750, 700, 539, 464 cm^{-1}. MS (m/z): [M-H]$^-$ calc'd for $C_{16}H_{17}O_4S$, 305.1, found 304.8.

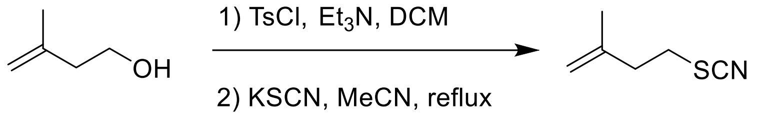

M4

In a 250 mL flask, 3-methylbut-3-en-1-ol (1.72 g,20 mmol) was dissolved in dry DCM (60 mL). To this solution, 4-methylbenzenesulfonyl chloride (4.58 g,24 mmol) and triethylamine (6.7 mL, 48 mmol) were added. After stirring at room temperature for 12 h, the reaction mixture was washed with 1 M HCl (2×50 mL) and brine (50 mL), dried by Na_2SO_4, filtered, and concentrated *in vacuo* to afford a yellowish oil. This oil was dissolved with MeCN (60 mL). KSCN (5.83 g, 60 mmol) was added to this solution and refluxed for 12 h. The reaction mixture was cooled to room temperature, and acetonitrile was removed under vacuum. The crude material was purified by column chromatography to afford **M4** as a yellowish oil (1.73 g, 68% yield, CAS: 3024523-04-1). ^{1}H NMR (400 MHz, $CDCl_3$): δ 4.88 (s, 1H), 4.80 (s, 1H), 3.07 (t, *J* = 7.5 Hz, 2H), 2.51 (t, *J* = 7.5 Hz, 2H), 1.75 (s, 3H). ^{13}C NMR (100 MHz, $CDCl_3$): δ 141.2, 113.5, 112.3, 37.7, 32.2, 22.0.

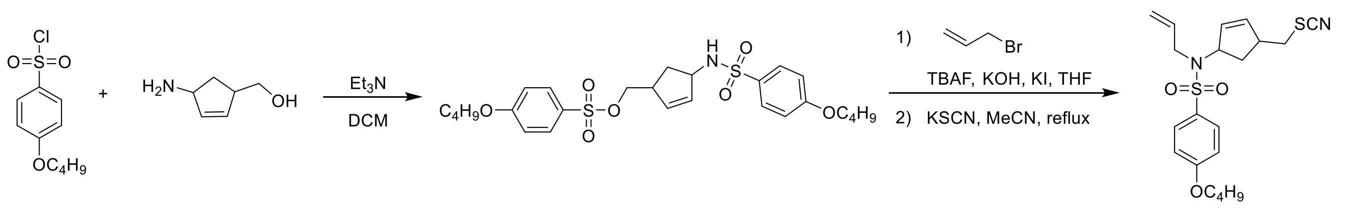

S6 **M5**

A 100 mL reaction flask was charged with 4-butoxybenzenesulfonyl chloride (3.11 g, 12.5 mmol), (1*S*,4*R*)-(4-aminocyclopent-2-enyl)methanol hydrochloride (0.75 g, 5 mmol), triethylamine (6.9 mL, 50 mmol) and DCM (30 mL). The reaction was stirred at room temperature overnight. After completion, DCM (30 mL) was added, and the organic layer was washed with brine (3×50 mL), dried over Na_2SO_4, and concentrated *in vacuo*. The crude product was purified

by column chromatography to afford **S6** as a white solid (1.61 g, 60% yield).^{1}H NMR (400 MHz, $CDCl_3$): δ 7.85–7.69 (m, 4H), 6.96 (dd, *J* = 13.0, 8.6 Hz, 4H), 5.64–5.57 (m, 1H), 5.55–5.46 (m, 1H), 4.85 (d, *J* = 9.4 Hz, 1H), 4.36–4.26 (m, 1H), 4.05–3.96 (m, 4H), 3.93–3.80 (m, 2H), 2.80 (t, *J* = 6.6 Hz, 1H), 2.31–2.16 (m, 1H), 1.86–1.68 (m, 4H), 1.57–1.40 (m, 4H), 1.22–1.14 (m, 1H), 0.96 (t, *J* = 7.4 Hz, 6H). ^{13}C NMR (100 MHz, $CDCl_3$): δ 163.6, 162.5, 133.4, 133.4, 132.2, 130.1, 129.2, 126.6, 115.0, 114.7, 71.9, 68.3, 68.2, 58.8, 43.8, 34.2, 31.1, 31.0, 19.2, 19.2, 13.9, 13.8. IR (KBr, thin film): 3286, 2959, 2874, 1596, 1578, 1497, 1467, 1416, 1357, 1336, 1305, 1260, 1189, 1168, 1096, 1007, 965, 906, 833, 750, 688, 587, 563 cm^{-1}. MS (m/z): $[M+H]^+$ calc'd for $C_{26}H_{36}NO_7S_2$, 538.2, found 538.0.

A 100 mL reaction flask was charged with **S6** (2.15 g, 4 mmol), 3-bromoprop-1-ene (0.85 g, 7.2 mmol), tetrabutylammonium fluoride (TBAF) (0.8 mL, 0.8 mmol), KI (66 mg, 0.4 mmol), KOH (0.47 g, 8.4 mmol) and DCM (20 mL). The reaction was stirred at room temperature overnight. After completion, the solution was quenched with saturated NH_4Cl solution and extracted with ethyl acetate (3×30 mL). The combined organic extracts were washed with brine (3×50 mL), dried over Na_2SO_4, filtered, and concentrated *in vacuo* to afford a yellowish oil. This oil was dissolved in MeCN (10 mL), and KSCN (1.17 g, 12 mmol) was added. The mixture was refluxed for 12 h. After cooling to room temperature, the acetonitrile was removed under vacuum. The crude product was then purified by column chromatography to afford **M5** as a yellowish oil (1.27 g, 78% yield). ^{1}H NMR (400 MHz, $CDCl_3$): δ 7.75–7.67 (m, 2H), 6.98–6.88 (m, 2H), 5.90–5.74 (m, 2H), 5.54–5.46 (m, 1H), 5.17 (dd, *J* = 17.2, 1.6 Hz, 1H), 5.10 (dd, *J* = 10.2, 1.5 Hz, 1H), 5.07–4.98 (m, 1H), 3.99 (t, *J* = 6.5 Hz, 2H), 3.76–3.60 (m, 2H), 3.04–2.87 (m, 3H), 2.51–2.37 (m, 1H), 1.81–1.71 (m, 2H), 1.53–1.42 (m, 2H), 1.41–1.32 (m, 1H), 0.96 (t, *J* = 7.4 Hz, 3H). ^{13}C NMR (100 MHz, $CDCl_3$): δ 162.5, 135.9, 134.4, 133.3, 131.6, 129.2, 117.1, 114.7, 112.2, 68.1, 63.9, 46.5, 44.2, 38.6, 34.0, 31.0, 19.2, 13.8. IR (KBr, thin film): 2959, 2873, 2154, 1595, 1577, 1497, 1468, 1415, 1378, 1336, 1303, 1258, 1154, 1113, 1093, 1053, 923, 835, 753, 685, 592, 568 cm^{-1}. MS (m/z): $[M+H]^+$ calc'd for $C_{20}H_{26}N_2O_3S_2$, 407.1, found 407.0.

.

Scheme S3. Polymerization of different monomers.

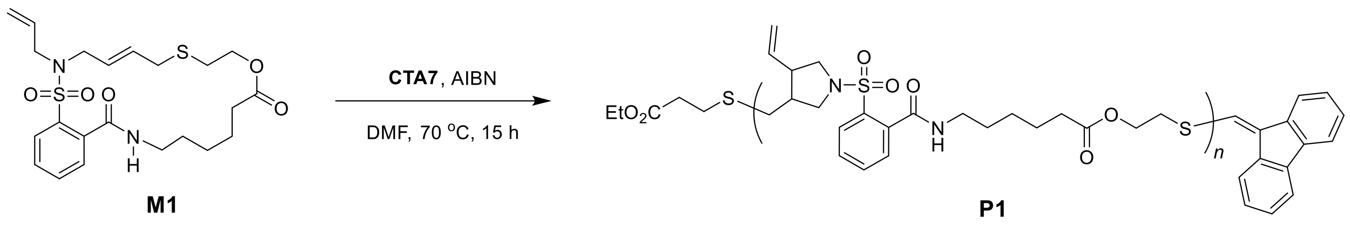

A 10 mL Schlenk vial equipped with a stir bar was charged with monomer **M1** (93.3 mg, 0.2 mmol), followed by the stock solution of **CTA7** (0.04 M, 200 µL, 0.008 mmol), AIBN (0.04 M, 160 µL, 0.0064 mmol), and DMF (640 µL). The vial was sealed. The solution was deoxygenated via three freeze-pump-thaw cycles, backfilled with nitrogen, and then heated at 70 °C for 15 h. After the reaction, the vial was cooled and opened to air to stop the polymerization. The reaction mixture was diluted with a minimum amount of DCM and precipitated in hexane. The obtained solid was re-dissolved with a minimum amount of DCM for further precipitation, yielding the polymer that was then characterized using SEC, NMR and FTIR.

$M_{n,SEC}$ = 9700, $Đ$ = 1.38

^{1}H NMR (400 MHz, $CDCl_3$): δ 7.96–7.85 (m, 1H), 7.64–7.45 (m, 3H), 6.30 (s, 1H), 5.73–5.48 (m, 1H), 5.17–5.01 (m, 2H), 4.21–4.07 (m, 2H), 3.74–3.57 (m, 1H), 3.56–3.29 (m, 4H), 3.24–2.83 (m, 2H), 2.76–2.60 (m, 2H), 2.58–2.06 (m, 5H), 1.69–1.56 (m, 4H), 1.41 (q, J = 8.3, 7.9 Hz, 2H).

^{13}C NMR (100 MHz, $CDCl_3$): δ 173.4, 168.2, 136.9, 136.3, 135.4, 134.2, 132.9, 129.8, 129.7, 129.5, 129.5, 118.3, 118.3, 63.3, 63.2, 52.2, 52.2, 51.6, 50.8, 48.6, 45.5, 44.4, 42.5, 40.3, 34.1, 33.7, 31.4, 31.2, 31.0, 29.0, 26.5, 24.6.

IR (KBr, thin film): 3363, 2923, 2853, 1732, 1659, 1537, 1469, 1378, 1343, 1260, 1162, 1076, 911, 733, 651, 602, 573 cm^{-1}.

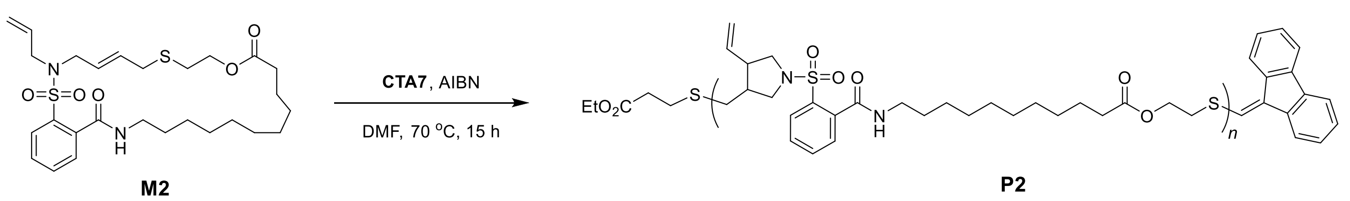

A 10 mL Schlenk vial equipped with a stir bar was charged with monomer **M2** (107.4 mg, 0.2 mmol), followed by the stock solution of **CTA7** (0.04 M, 200 µL, 0.008 mmol), AIBN (0.04 M, 160 µL, 0.0064 mmol), and DMF (640 µL). The vial was sealed. The solution was deoxygenated via three freeze-pump-thaw cycles, backfilled with nitrogen, and then heated at 70 °C for 15 h. After the reaction, the vial was cooled and opened to air to stop the polymerization. The reaction mixture was diluted with a minimum amount of DCM and precipitated in hexane. The obtained solid was re-dissolved with a minimum amount of DCM for further precipitation, yielding the polymer that was then characterized using SEC, NMR and FTIR.

$M_{n,SEC}$ = 13100, $Đ$ = 1.39

^{1}H NMR (400 MHz, $CDCl_3$): δ 7.96–7.85 (m, 1H), 7.62–7.56 (m, 1H), 7.56–7.48 (m, 2H), 6.36–6.14 (m, 1H), 5.72–5.50 (m, 1H), 5.17–5.02 (m, 2H), 4.19–4.07 (m, 2H), 3.73–3.30 (m, 5H), 3.23–2.84 (m, 2H), 2.77–2.61 (m, 2H), 2.60–2.44 (m, 1H), 2.44–2.08 (m, 4H), 1.64–1.51 (m, 4H), 1.38–1.17 (m, 12H).

^{13}C NMR (100 MHz, $CDCl_3$): δ 173.7, 168.1, 137.0, 136.3, 135.5, 135.4, 134.2, 132.9, 129.9, 129.7, 129.6, 129.5, 118.3, 118.2, 63.3, 63.2, 52.2, 51.6, 50.8, 48.6, 45.5, 44.4, 42.5, 40.7, 34.3, 33.7, 31.5, 31.1, 31.0, 29.6, 29.5, 29.4, 29.4, 29.3, 29.2, 27.1, 25.0.

IR (KBr, thin film): 3366, 2926, 2854, 1735, 1662, 1537, 1466, 1377, 1345, 1257, 1163, 1071, 921, 760, 729, 652, 603, 573 cm^{-1}.

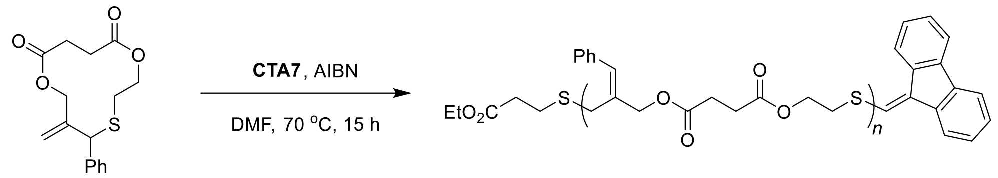

M3 **P3**

A 10 mL Schlenk vial equipped with a stir bar was charged with monomer **M3** (61.3 mg, 0.2 mmol), followed by the stock solution of **CTA7** (0.04 M, 200 µL, 0.008 mmol), AIBN (0.04 M, 100 µL, 0.004 mmol), and DMF (700 µL). The vial was sealed. The solution was deoxygenated via three freeze-pump-thaw cycles, backfilled with nitrogen, and then heated at 70 °C for 15 h. After the reaction, the vial was cooled and opened to air to stop the polymerization. The reaction mixture was diluted with a minimum amount of DCM and precipitated in hexane. The obtained solid was re-dissolved with a minimum amount of DCM for further precipitation, yielding the polymer that was then characterized using SEC, NMR and FTIR.

$M_{n,SEC}$ = 5900, $Đ$ = 1.32

^{1}H NMR (400 MHz, $CDCl_3$): δ 7.40–7.19 (m, 5H), 6.69 (s, 1H), 4.85 (s, 2H), 4.28–3.96 (m, 2H), 3.48–3,34 (m, 2H), 2.74–2.53 (m, 6H).

^{13}C NMR (100 MHz, $CDCl_3$): δ 172.0, 135.9, 135.8, 133.6, 132.4, 132.3, 132.0, 128.9, 128.8, 128.6, 128.6, 127.8, 127.7, 67.2, 63.5, 63.3, 61.2, 37.3, 30.6, 30.4, 29.2, 29.1, 29.1.

IR (KBr, thin film): 2956, 2925, 2852, 1732, 1492, 1447, 1410, 1379, 1349, 1151, 1073, 974, 910, 733, 700 cm^{-1}.

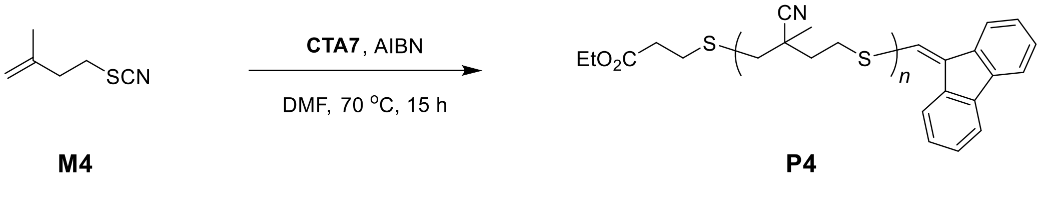

M4 **P4**

A 10 mL Schlenk vial equipped with a stir bar was charged with monomer **M4** (101.8 mg, 0.8 mmol), followed by the stock solution of **CTA7** (0.08 M, 400 µL, 0.032 mmol), AIBN (0.04 M, 400 µL, 0.016 mmol), and DMF (200 µL). The vial was sealed. The solution was deoxygenated via three freeze-pump-thaw cycles, backfilled with nitrogen, and then

heated at 70 °C for 15 h. After the reaction, the vial was cooled and opened to air to stop the polymerization. The reaction mixture was diluted with a minimum amount of DCM and precipitated in hexane. The obtained solid was re-dissolved with a minimum amount of DCM for further precipitation, yielding the polymer that was then characterized using SEC, NMR and FTIR.

$M_{n,SEC}$ = 5000, $Đ$ = 1.24

^{1}H NMR (400 MHz, $CDCl_3$): δ 2.89–2.67 (m, 4H), 2.12–1.99 (m, 1H), 1.91–1.76 (m, 1H), 1.43 (s, 3H).

^{13}C NMR (100 MHz, $CDCl_3$): δ 122.8, 41.7, 38.7, 38.4, 29.3, 23.9.

IR (KBr, thin film): 2924, 2854, 2234, 1731, 1460, 1379, 1344, 1311, 1242, 1208, 1154, 1082, 911, 843, 772, 731, 648 cm^{-1}.

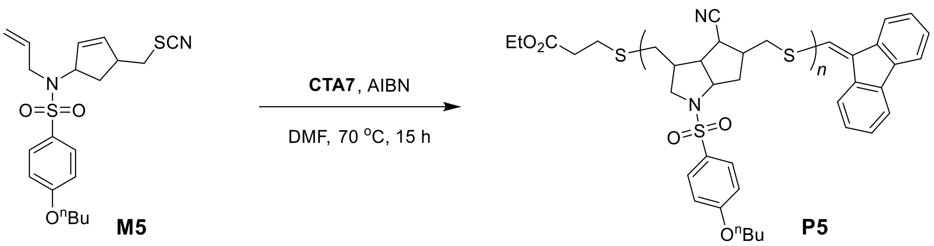

M5 P5

A 10 mL Schlenk vial equipped with a stir bar was charged with monomer **M5** (81.3 mg, 0.2 mmol), followed by the stock solution of **CTA7** (0.04 M, 200 µL, 0.008 mmol), AIBN (0.04 M, 160 µL, 0.0064 mmol), and DMF (640 µL). The vial was sealed. The solution was deoxygenated via three freeze-pump-thaw cycles, backfilled with nitrogen, and then heated at 70 °C for 15 h. After the reaction, the vial was cooled and opened to air to stop the polymerization. The reaction mixture was diluted with a minimum amount of DCM and precipitated in hexane. The obtained solid was re-dissolved with a minimum amount of DCM for further precipitation, yielding the polymer that was then characterized using SEC, NMR and FTIR.

$M_{n,SEC}$ = 5200, $Đ$ = 1.20

^{1}H NMR (400 MHz, $CDCl_3$): δ 7.80–7.65 (m, 2H), 7.06–6.90 (m, 2H), 4.23–4.10 (m, 1H), 4.10–3.93 (m, 2H), 3.89–3.51 (m, 2H), 3.39–3.08 (m, 1H), 2.95–2.08 (m, 8H), 1.90–1.70 (m, 3H), 1,57–1.43 (m, 2H), 0.98 (t, J = 7.4 Hz, 3H).

^{13}C NMR (100 MHz, $CDCl_3$): δ 163.1, 162.9, 130.0, 129.4, 115.0, 115.0, 68.4, 68.4, 63.7, 63.6, 50.6, 44.4, 44.2, 41.5, 39.9, 37.9, 33.9, 31.2, 29.5, 27.3, 19.3, 14.0.

IR (KBr, thin film): 2957, 2927, 2871, 1594, 1576, 1496, 1466, 1342, 1304, 1259, 1155, 1093, 1026, 911, 836, 734, 594, 571 cm^{-1}.

Scheme S4. Radical reaction of polymers **P1** with different thiols.

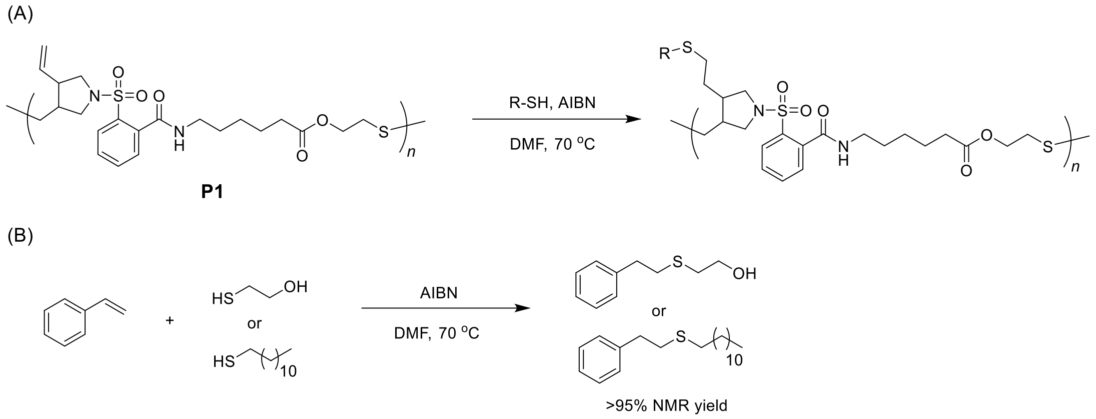

A 10 mL Schlenk vial was charged with thiol (0.15 mmol), followed by the addition of a solution of polymer (0.1 mmol repeating units) and AIBN (0.01 mmol) in DMF (1 mL). The vial was sealed, and the solution was deoxygenated via three freeze-pump-thaw cycles and then backfilled with nitrogen. After stirring at 70 °C for 15 h, the vial was cooled in an ice bath and opened to the air to stop the reaction. The reaction mixture was diluted with a minimum amount of DCM and precipitated in diethyl ether, yielding the polymer, which was then characterized using ^{1}H NMR (**Scheme S4-A**). Additionally, control experiments were also performed to confirm the generation of thiyl radicals under the same reaction conditions (**Scheme S4-B**). As shown in **Figure S1**, the pendant vinyl groups in the polymers still largely remained intact under thiyl radical conditions, indicating that they are far less susceptible to attack by thiyl radicals. This is likely because the pendant vinyl groups are sterically shielded by the surrounding polymer backbone, which prevents the propagating thiyl radicals from approaching these vinyl groups. This observation is consistent with the existing literature (see Ref. *Macromolecules*, 2022, **55**, 9411).

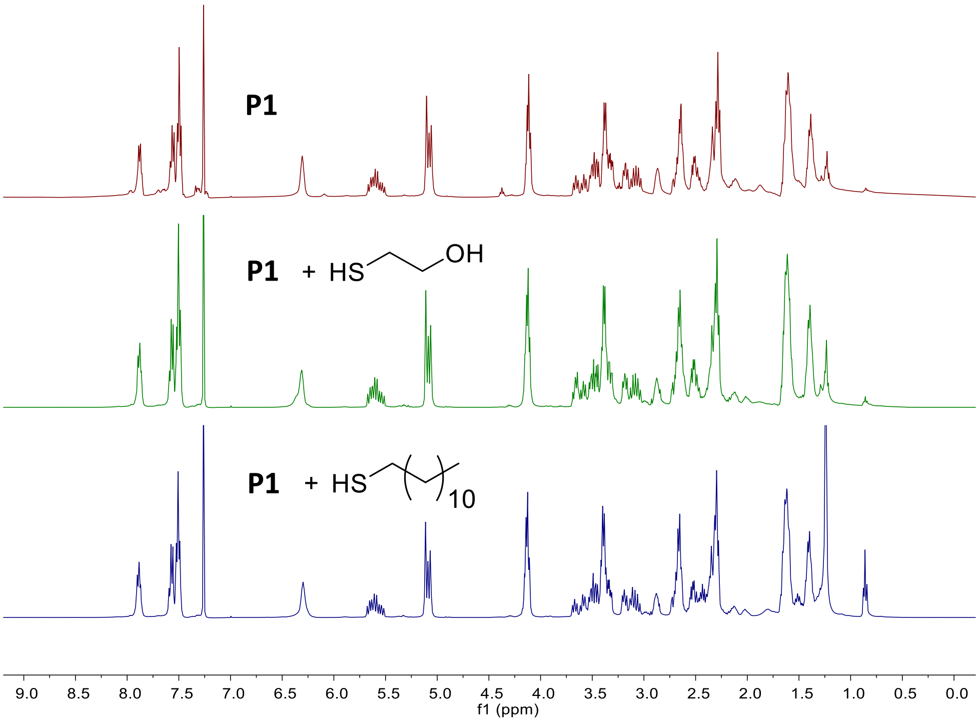

Figure S1. ^{1}H NMR ($CDCl_3$, 25°C) spectra of polymers resulted from the reaction of **P1** and thiols.

NMR analysis of the conversion of monomer M1

Monomer conversion was determined based on the assumption that the integral of peak a at δ = 4.16 ppm corresponds to methylene groups present in both monomers and polymers. In contrast, the integral of peak b at δ = 3.93 ppm corresponds to a methylene group found in unreacted monomers. By normalizing the integral of peak b to 2, the monomer conversion (α) was calculated using the following equation:

$$\alpha = (1 - \frac{I_b}{I_a}) \times 100\%$$

For example:

α = [1-(2÷5.69)] ×100% = 65%

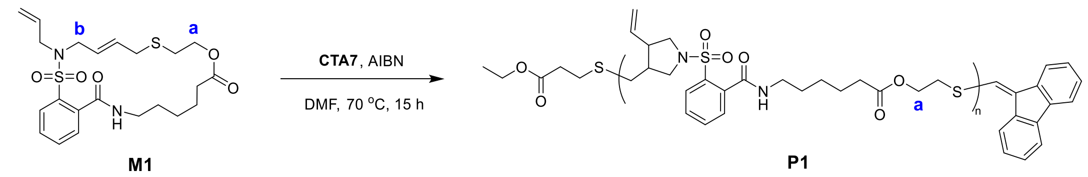

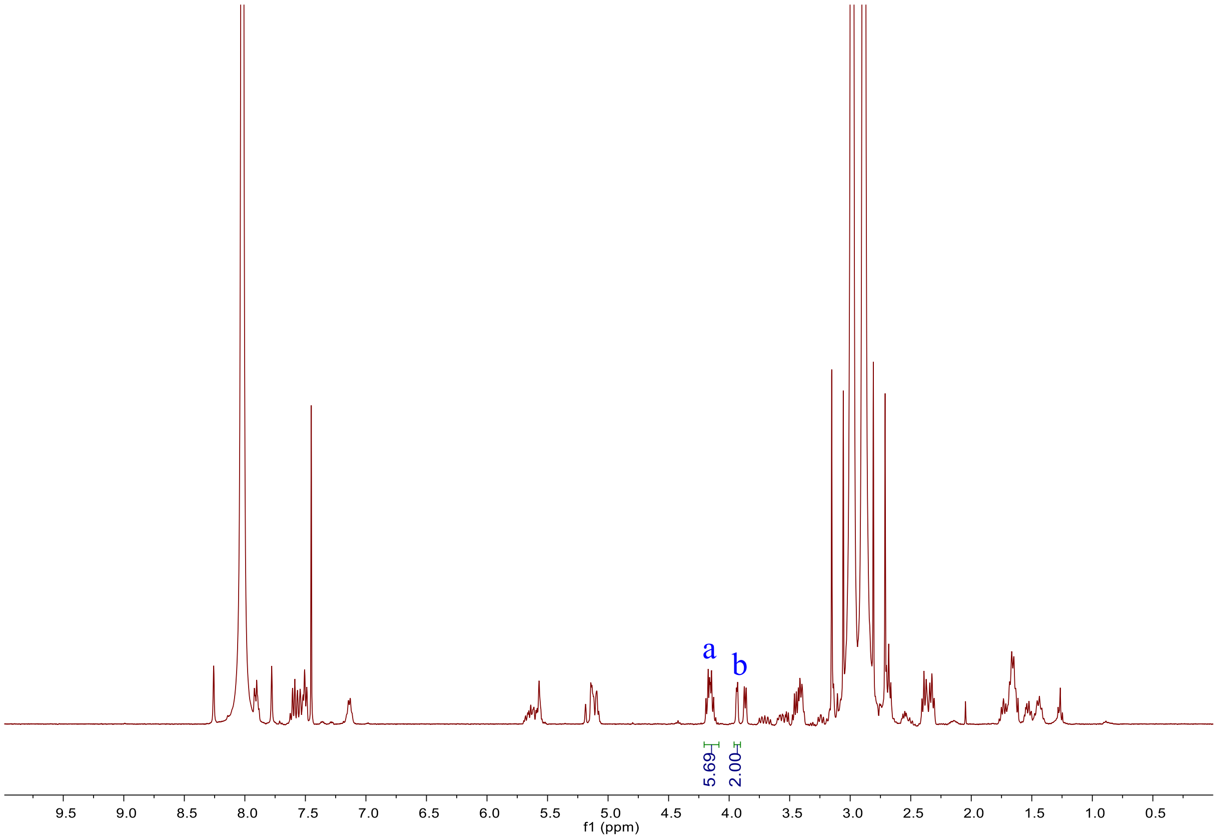

Figure S2. ^{1}H NMR ($CDCl_3$, 25°C) determination of monomer conversion for the polymerization of monomer **M1** (Table S1, entry 8).

NMR analysis of the molecular weight of P1

The determination of $M_{n,NMR}$ for the polymers was based on the assumption that the integral of the peak at δ = 7.34 ppm (Peaks a and b) corresponds to the alkenyl group and the phenyl group of the polymer chain-end, and the integral of the peak at δ = 7.93 ppm (Peak c) corresponds to the phenyl group of the polymer repeating unit. By normalizing the integral of Peaks a and b to 3, the $M_{n,NMR}$ was calculated based on the following equation:

$$M_{n,NMR} = \frac{3I_c}{I_{a,b}} MW^M + MW^{CTA}$$

For example:

$$M_{n,NMR} = 17.19 \times 466.61 + 310.41 = 8300$$

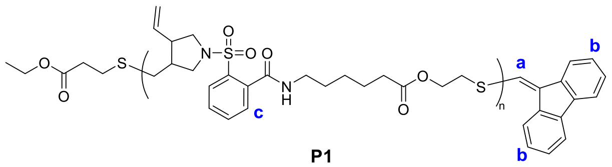

P1

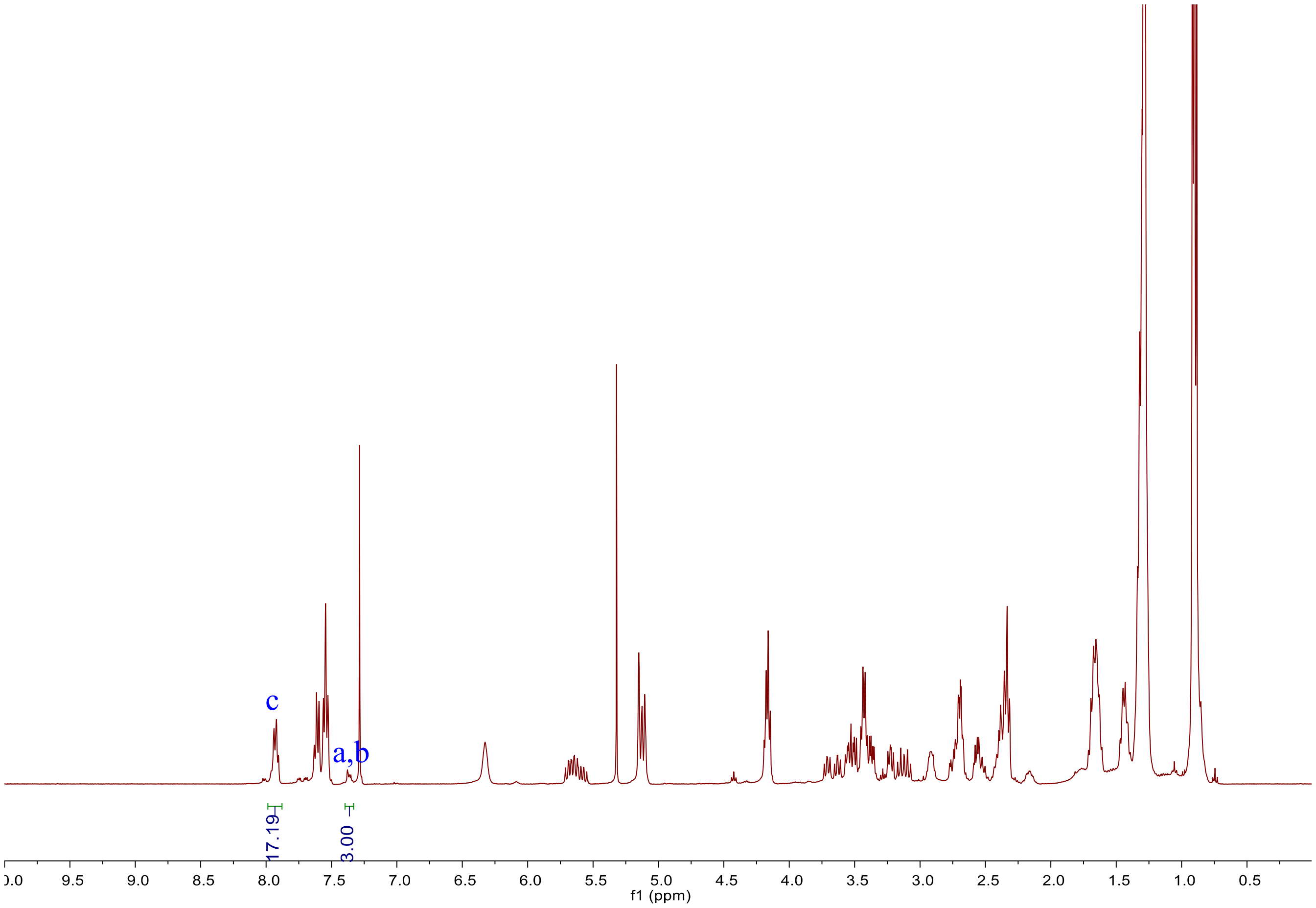

Figure S3. ^{1}H NMR ($CDCl_3$, 25°C) determination of molecular weight of **P1** (Table S1, entry 8).

Table S1. Effect of different CTAs on the polymerization of monomer **M1**.

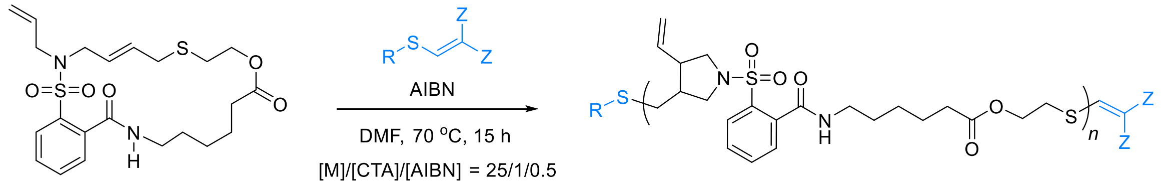

M1 **P1**

Chain transfer agents:

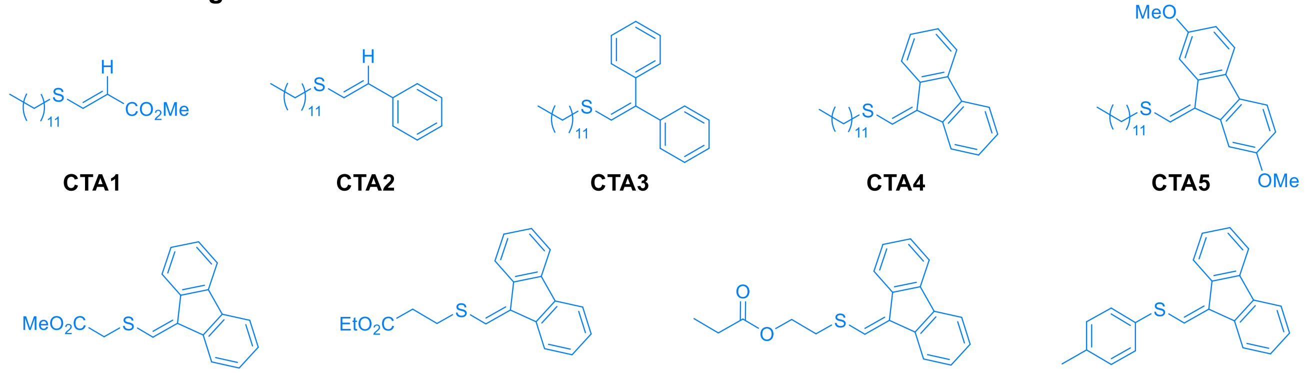

Entry[a]	SRAFT agent	Conversion[b]	$M_{n,theo}$[c]	$M_{n,NMR}$[d]	$M_{n,SEC}$[e]	$Đ$[e]
1[f]	/	78%	/	/	27200	1.77
2	**CTA1**	47%	5800	/	25400	1.69
3	**CTA2**	48%	6000	22500	13200	1.55
4	**CTA3**	56%	6900	12700	11400	1.45
5	**CTA4**	54%	6700	7500	9200	1.38
6	**CTA5**	50%	6300	10900	9100	1.36
7	**CTA6**	59%	7200	7800	9300	1.36
8	**CTA7**	65%	7900	8300	9700	1.38
9	**CTA8**	53%	6500	7500	8900	1.39
10	**CTA9**	25%	3200	4600	5400	1.28

[a] Experimental conditions: [**M**] = 0.1 M, reacted at 70 °C for 15 h under a nitrogen atmosphere, unless otherwise noted. [b] Monomer conversion was determined by ^1H NMR analysis of the crude reaction mixture. [c] Theoretical molecular weight was calculated using the following equation: $M_{n\ theo}$= $([M]_0/[CTA]_0) \times MW^M \times$ conversion + MW^{CTA}, where $[M]_0$, $[CTA]_0$, MW^M and MW^{CTA} correspond to initial monomer concentration, initial CTA concentration, molar mass of monomer unit, and molar mass of CTA, respectively. [d] Molecular weight was determined by ^1H NMR analysis of the isolated polymers. [e] Molecular weight and polydispersity index ($Đ$) were determined by SEC analysis in THF at 40 °C using polystyrene standards. [f] [M]/[AIBN] = 25/1.

Note: The overall conversion for the polymerization of these macrocyclic monomers was relatively low, which may be attributed to the increasing steric hindrance between the bulky macrocyclic monomers and the growing polymer chains, thereby reducing the effective collision frequency between monomers and propagating radicals. Similar inhibition phenomena have also been observed in previous studies using ROMP (see ref. *J. Am. Chem. Soc.*, 2015, **137**, 8038) and RAFT (see ref. *J. Am. Chem. Soc.*, 2019, **141**, 12493 and *Angew. Chem. Int. Ed.*, 2023, **62**, e202308524) methods with comparable monomers.

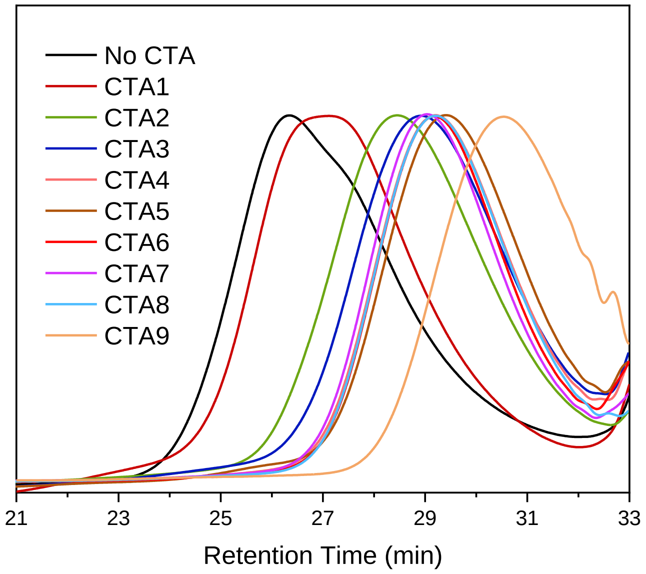

Figure S4. SEC traces for the polymerization of monomer **M1** with different CTAs (Table S1, entries 1–10).

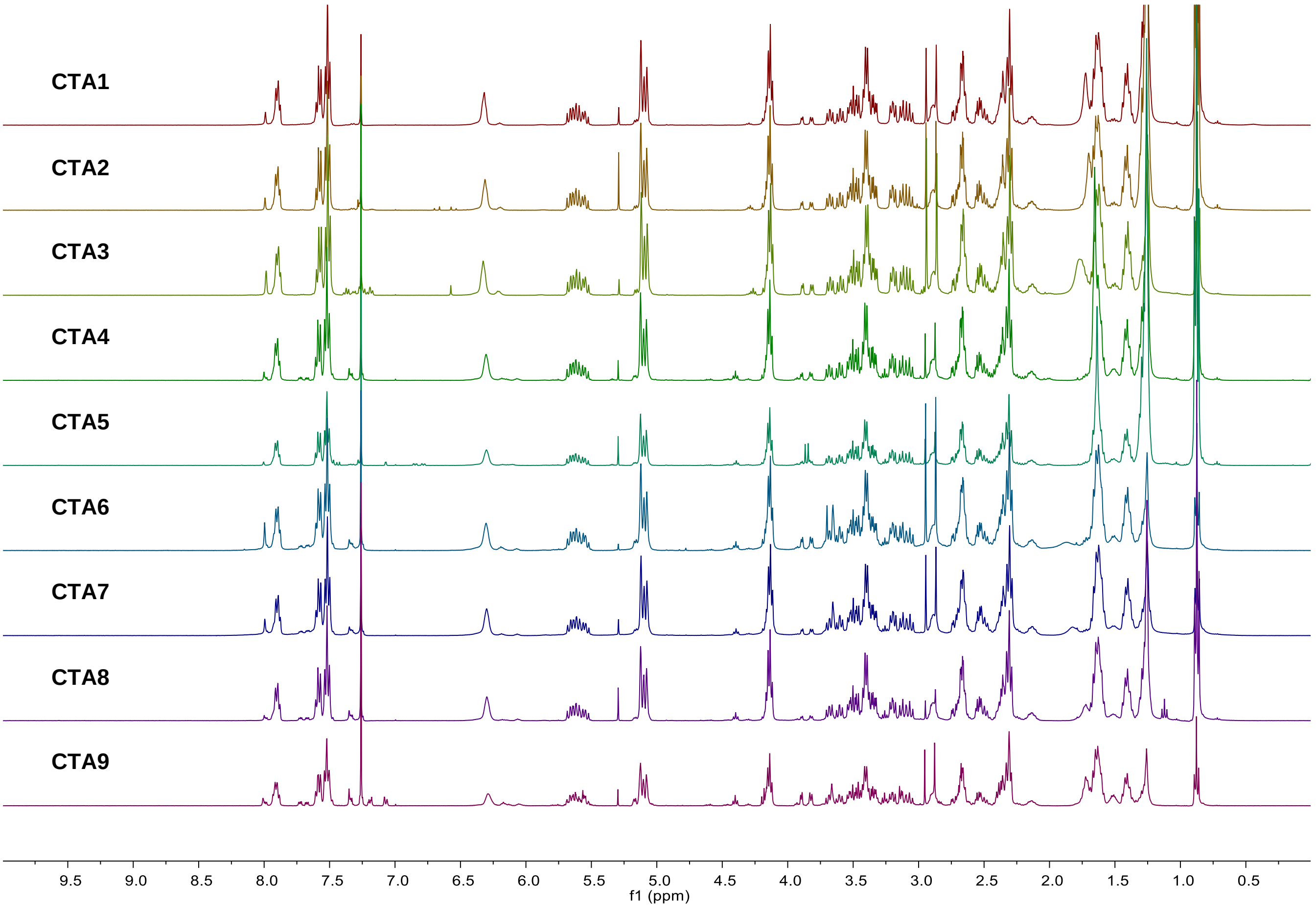

Figure S5. ^{1}H NMR ($CDCl_3$, 25°C) spectra of the resulting polymers in table S1.

Table S2. Effect of different solvents on the polymerization of monomer **M1**.

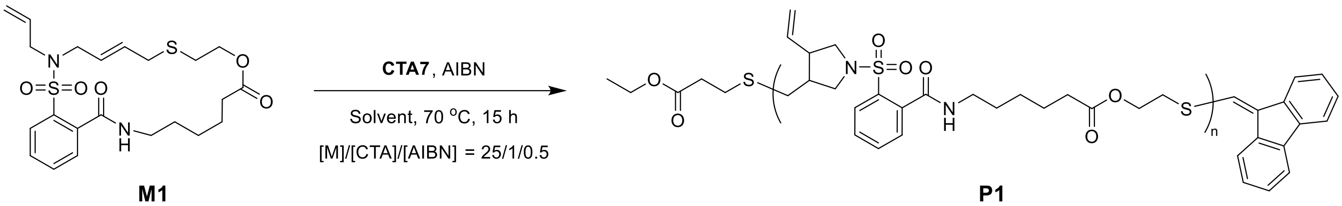

Entry[a]	Solvent	Conversion[b]	$M_{n,theo}$[c]	$M_{n,NMR}$[d]	$M_{n,SEC}$[e]	$Đ$[e]
1	DMF	65%	7900	8300	9700	1.38
2	DMSO	75%	9100	8400	9400	1.43
3	THF	45%	5600	6800	6800	1.31
4	1,4-Dioxane	15%	2100	6700	4600	1.21
5	Toluene	23%	3000	5400	6900	1.27

[a] Experimental conditions: [**M**] = 0.1 M, reacted at 70 °C for 15 h under a nitrogen atmosphere, unless otherwise noted. [b] Monomer conversion was determined by ^{1}H NMR analysis of the crude reaction mixture. [c] Theoretical molecular weight was calculated using the following equation: $M_{n\ theo}$= $([M]_0/[CTA]_0) \times MW^{M} \times$ conversion + MW^{CTA}, where $[M]_0$, $[CTA]_0$, MW^{M} and MW^{CTA} correspond to initial monomer concentration, initial CTA concentration, molar mass of monomer unit, and molar mass of CTA, respectively. [d] Molecular weight was determined by ^{1}H NMR analysis of the isolated polymers. [e] Molecular weight and polydispersity index ($Đ$) were determined by SEC analysis in THF at 40 °C using polystyrene standards.

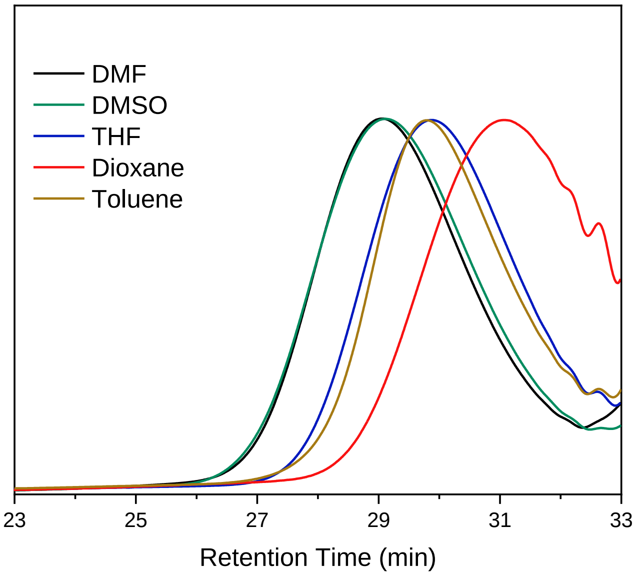

Figure S6. SEC traces for the polymerization of monomer **M1** in different solvents (Table S2, entries 1–5).

Table S3. Effect of different concentrations on the polymerization of monomer **M1**.

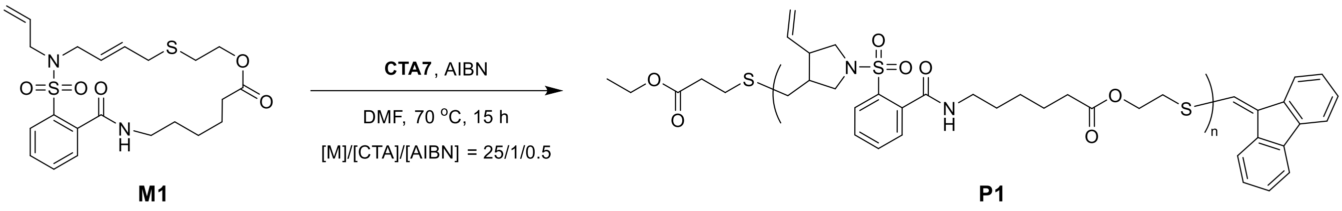

Entry[a]	Monomer Concentration	Conversion[b]	$M_{n,theo}$[c]	$M_{n,SEC}$[d]	$Đ$[d]
1	0.05 M	49%	6000	8500	1.38
2	0.1 M	65%	7900	9700	1.38
3	0.2 M	71%	8600	10500	1.40
4	0.4 M	75%	9100	10300	1.42

[a] Experimental conditions: carried out in DMF at 70 °C for 15 h under a nitrogen atmosphere, unless otherwise noted. [b] Monomer conversion was determined by ^1H NMR analysis of the crude reaction mixture. [c] Theoretical molecular weight was calculated using the following equation: $M_{n\ theo}$= $([M]_0/[CTA]_0) \times MW^M \times$ conversion + MW^{CTA}, where $[M]_0$, $[CTA]_0$, MW^M and MW^{CTA} correspond to initial monomer concentration, initial CTA concentration, molar mass of monomer unit, and molar mass of CTA, respectively. [d] Molecular weight and polydispersity index ($Đ$) were determined by SEC analysis in THF at 40 °C using polystyrene standards.

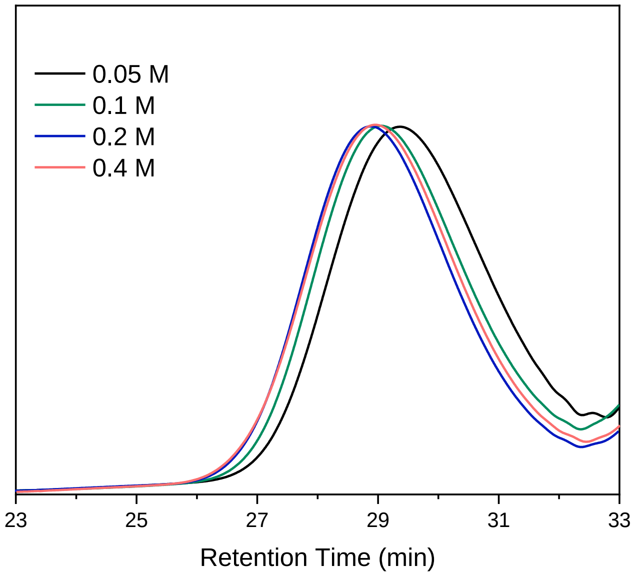

Figure S7. SEC traces for the polymerization of monomer **M1** at different concentrations (Table S3, entries 1–4).

Table S4. Effect of different amounts of AIBN on the polymerization of monomer **M1**.

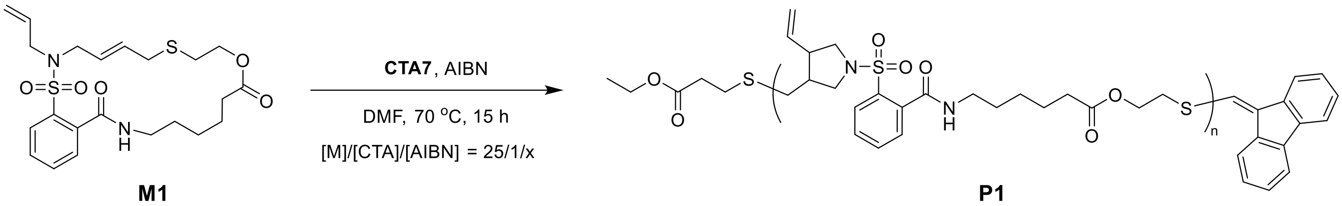

Entry[a]	[M]/[CTA]/[AIBN]	Conversion[b]	$M_{n,theo}$[c]	$M_{n,NMR}$[d]	$M_{n,SEC}$[e]	$Đ$[e]
1	25/1/1	73%	8800	9400	10700	1.37
2	25/1/0.8	75%	9100	9000	10700	1.38
3	25/1/0.5	71%	8600	9100	10500	1.40
4	25/1/0.3	67%	8200	8400	10100	1.40
5	25/1/0.1	34%	4300	5300	7400	1.36

[a] Experimental conditions: [**M**] = 0.2 M, reacted at 70 °C for 15 h under a nitrogen atmosphere, unless otherwise noted. [b] Monomer conversion was determined by ^{1}H NMR analysis of the crude reaction mixture. [c] Theoretical molecular weight was calculated using the following equation: $M_{n\ theo}$= $([M]_0/[CTA]_0) \times MW^M \times$ conversion + MW^{CTA}, where $[M]_0$, $[CTA]_0$, MW^M and MW^{CTA} correspond to initial monomer concentration, initial CTA concentration, molar mass of monomer unit, and molar mass of CTA, respectively. [d] Molecular weight was determined by ^{1}H NMR analysis of the isolated polymers. [e] Molecular weight and polydispersity index ($Đ$) were determined by SEC analysis in THF at 40 °C using polystyrene standards.

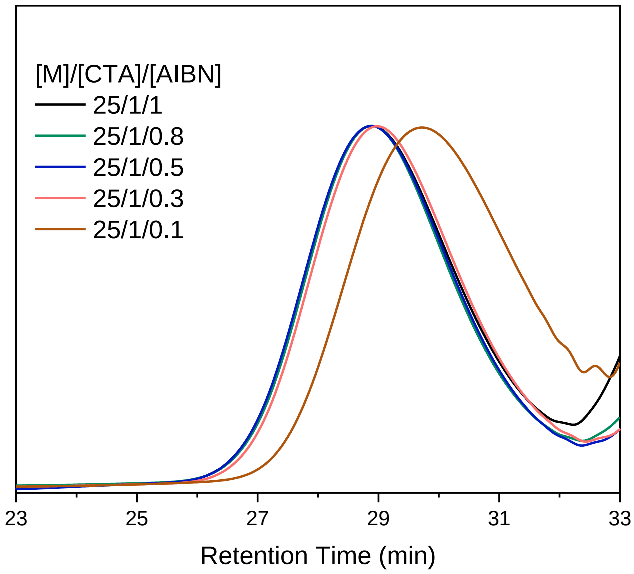

Figure S8. SEC traces for the polymerization of monomer **M1** at different amounts of AIBN (Table S4, entries 1–5).

Table S5. Polymerization of monomer **M1** at different monomer/CTA ratios.

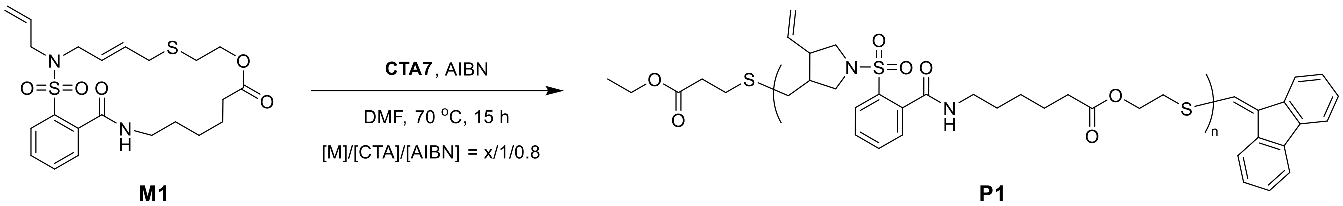

Entry[a]	[M]/[CTA]	Conversion[b]	$M_{n,theo}$[c]	$M_{n,NMR}$[d]	$M_{n,SEC}$[e]	$Đ$[e]
1	10/1	71%	3600	4300	5600	1.22
2	25/1	75%	9100	9000	10700	1.38
3	50/1	67%	15900	14000	17200	1.47
4	100/1	51%	24100	25000	21100	1.55

[a] Experimental conditions: [**M**] = 0.2 M, reacted at 70 °C for 15 h under a nitrogen atmosphere, unless otherwise noted. [b] Monomer conversion was determined by ^{1}H NMR analysis of the crude reaction mixture. [c] Theoretical molecular weight was calculated using the following equation: $M_{n\ theo}$= $([M]_0/[CTA]_0) \times MW^M \times$ conversion + MW^{CTA}, where $[M]_0$, $[CTA]_0$, MW^M and MW^{CTA} correspond to initial monomer concentration, initial CTA concentration, molar mass of monomer unit, and molar mass of CTA, respectively. [d] Molecular weight was determined by ^{1}H NMR analysis of the isolated polymers. [e] Molecular weight and polydispersity index ($Đ$) were determined by SEC analysis in THF at 40 °C using polystyrene standards.

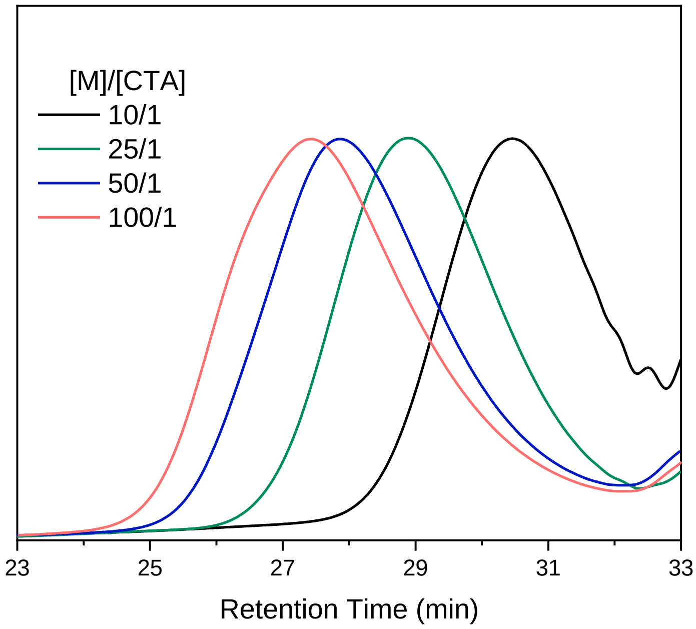

Figure S9. SEC traces for the polymerization of monomer **M1** at different monomer/CTA ratios (Table S5, entries 1–4).

NMR analysis of the conversion of monomer M2

Monomer conversion was determined based on the assumption that the integral of peak a at δ = 4.17 ppm corresponds to methylene groups present in both monomers and polymers. In contrast, the integral of peak b and c at δ = 3.87 ppm corresponds to a methylene group found in unreacted monomers. By normalizing the integral of peak b and c to 4, the monomer conversion (α) was calculated using the following equation:

$$\alpha = \left(1 - \frac{\frac{I_{b,c}}{2}}{I_a}\right) \times 100\%$$

For example:

$$\alpha = [1-(4 \div 2 \div 5.13)] \times 100\% = 61\%$$

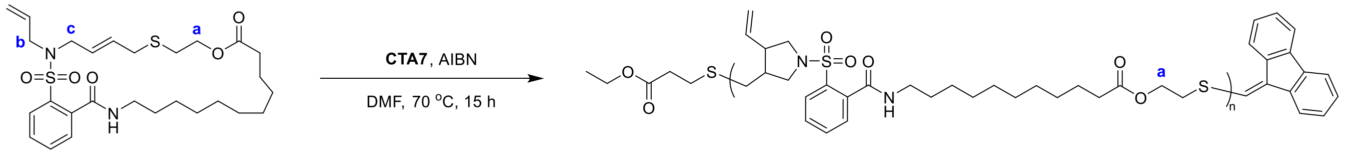

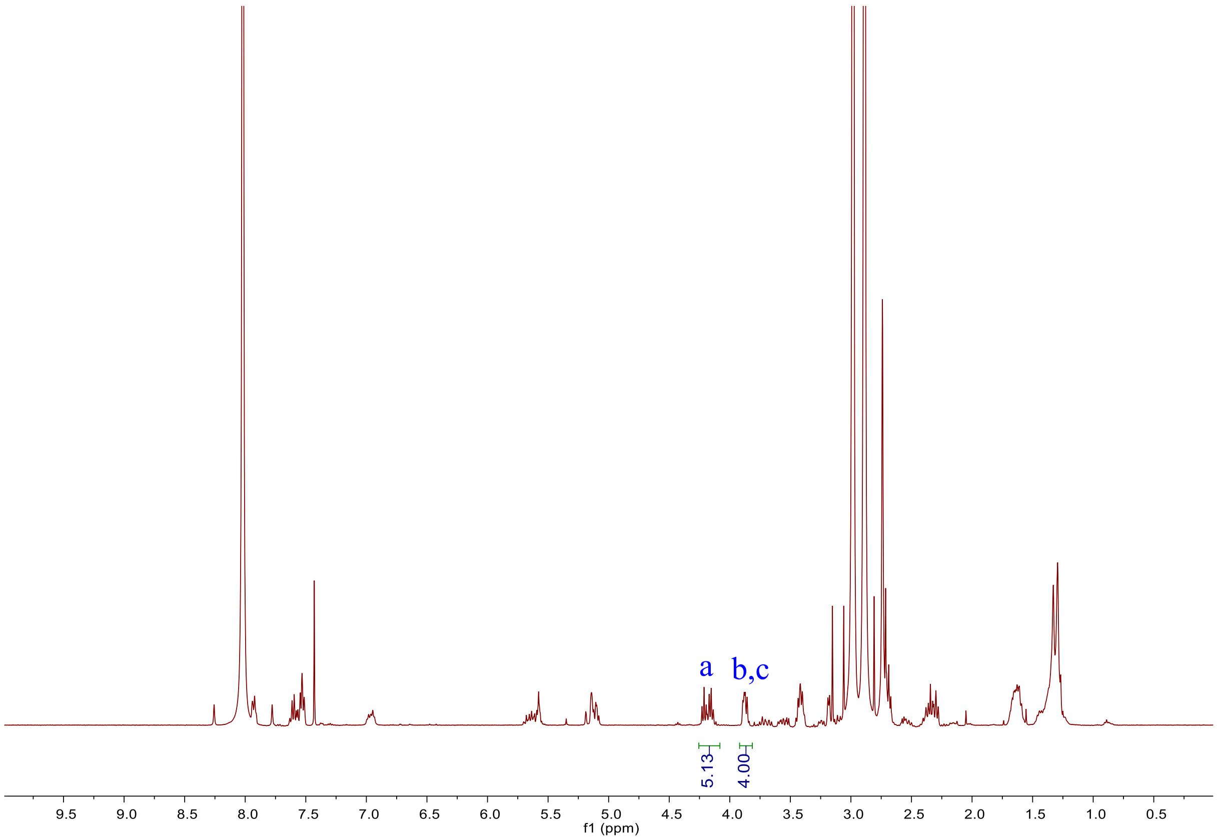

Figure S10. ^{1}H NMR ($CDCl_3$, 25°C) determination of monomer conversion for the polymerization of monomer **M2** (Table S6, entry 2).

NMR analysis of the molecular weight of P2

The determination of $M_{n,NMR}$ for the polymers was based on the assumption that the integral of the peak at δ = 7.34 ppm (Peaks a and b) corresponds to the alkenyl group and the phenyl group of the polymer chain-end, and the integral of the peak at δ = 7.93 ppm (Peak c) corresponds to the phenyl group of the polymer repeating unit. By normalizing the integral of Peaks a and b to 3, the $M_{n,NMR}$ was calculated based on the following equation:

$$M_{n,NMR} = \frac{3I_c}{I_{a,b}} MW^{M} + MW^{CTA}$$

For example:

$$M_{n,NMR} = 17.08 \times 536.75 + 310.41 = 9500$$

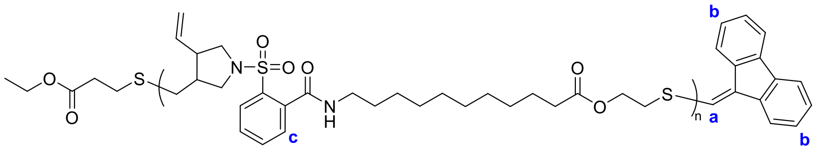

P2

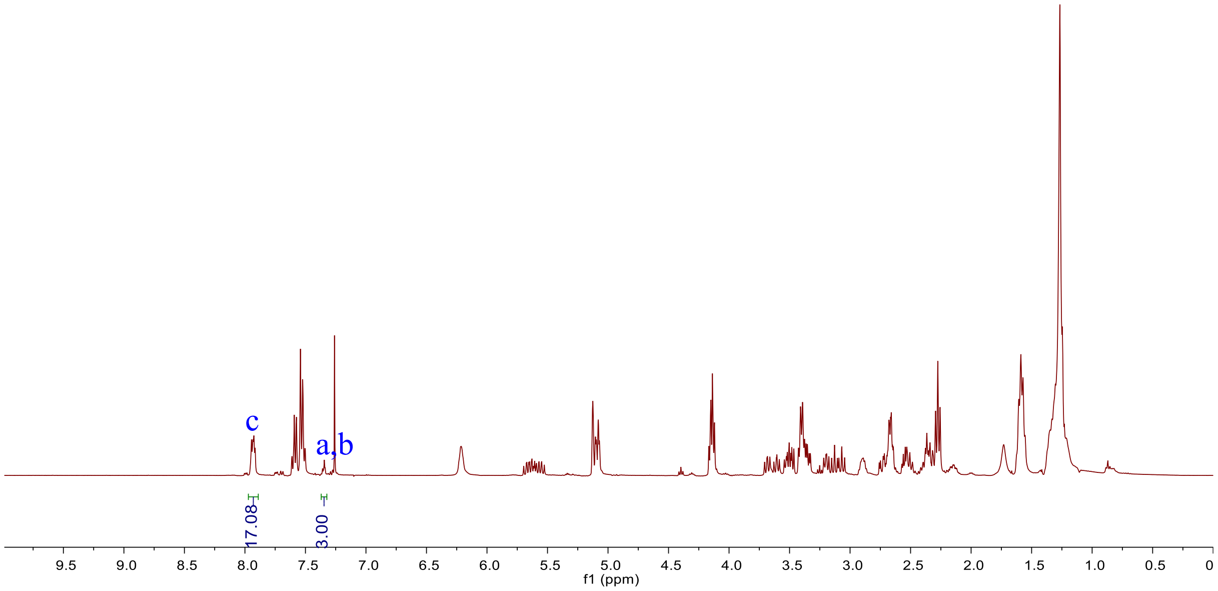

Figure S11. ^{1}H NMR ($CDCl_3$, 25°C) determination of molecular weight of **P2** (Table S6, entry 2).

Table S6. Polymerization of monomer **M2** at different monomer/CTA ratios.

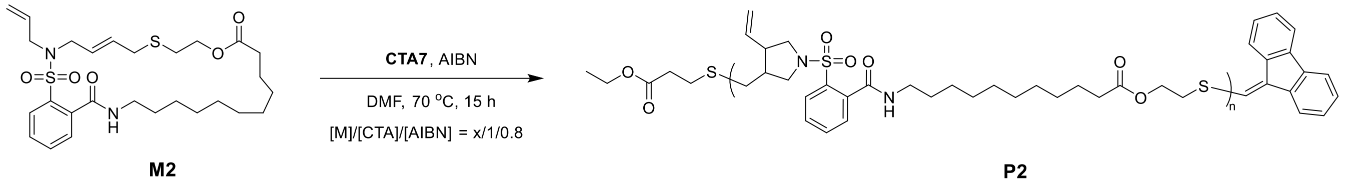

Entry[a]	[M]/[CTA]	Conversion[b]	$M_{n,theo}$[c]	$M_{n,NMR}$[d]	$M_{n,SEC}$[e]	$Đ$[e]
1	10/1	66%	3900	4500	6300	1.32
2	25/1	61%	8500	9500	13100	1.39
3	50/1	56%	15300	14400	19900	1.51

[a] Experimental conditions: [**M**] = 0.2 M, reacted at 70 °C for 15 h under a nitrogen atmosphere, unless otherwise noted. [b] Monomer conversion was determined by ^{1}H NMR analysis of the crude reaction mixture. [c] Theoretical molecular weight was calculated using the following equation: $M_{n\ theo}$= $([M]_0/[CTA]_0) \times MW^M \times$ conversion + MW^{CTA}, where $[M]_0$, $[CTA]_0$, MW^M and MW^{CTA} correspond to initial monomer concentration, initial CTA concentration, molar mass of monomer unit, and molar mass of CTA, respectively. [d] Molecular weight was determined by ^{1}H NMR analysis of the isolated polymers. [e] Molecular weight and polydispersity index ($Đ$) were determined by SEC analysis in THF at 40 °C using polystyrene standards.

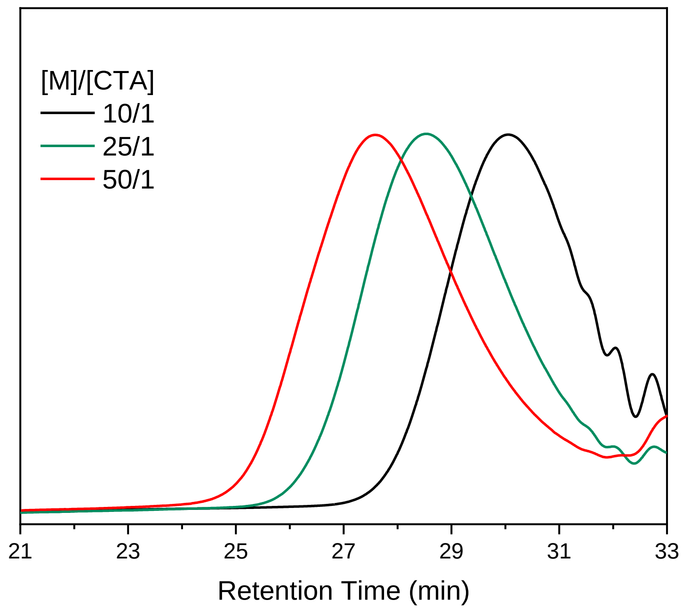

Figure S12. SEC traces for the polymerization of monomer **M2** at different monomer/CTA ratios (Table S6, entries 1–3).

NMR analysis of the conversion of monomer M3

Monomer conversion was determined based on the assumption that the integral of peak a at δ = 5.48 ppm corresponds to the alkenyl groups present in polymers. In contrast, the integral of peak b at δ = 6.72 ppm corresponds to the alkenyl group found in unreacted monomers. By normalizing the integral of peak b to 1, the monomer conversion (α) was calculated using the following equation:

$$\alpha = \left(\frac{I_a}{I_a + I_b}\right) \times 100\%$$

For example:

α = [1÷(1+1.85)] ×100% = 65%

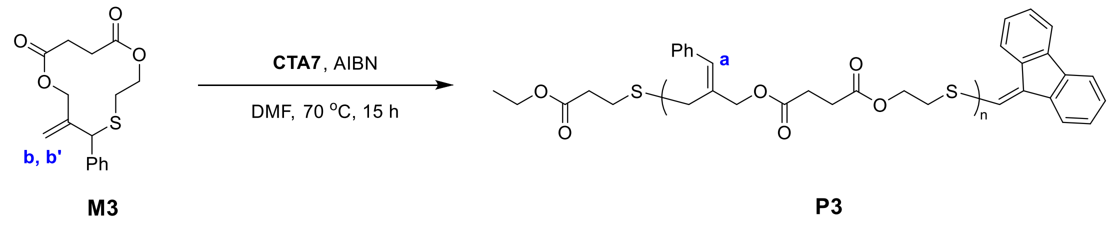

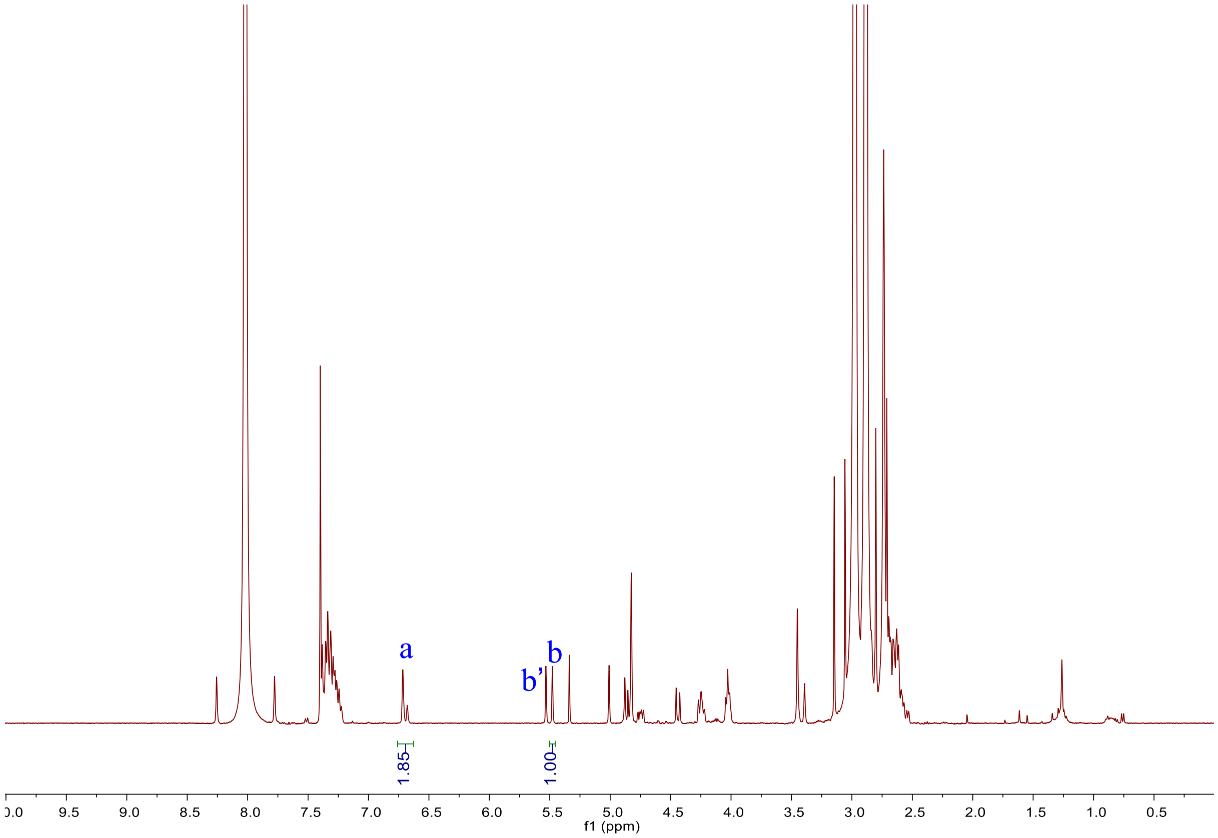

Figure S13. ^{1}H NMR ($CDCl_3$, 25°C) determination of monomer conversion for the polymerization of monomer **M3** (Table S7, entry 2).

NMR analysis of the molecular weight of P3

The determination of $M_{n,NMR}$ for the polymers was based on the assumption that the integral of the peak at δ = 7.60 ppm (Peak a) corresponds to the phenyl group of the polymer chain-end, and the integral of the peak at δ = 6.71 ppm (Peak b) corresponds to the alkenyl group of the polymer repeating unit. By normalizing the integral of Peak a to 1, the $M_{n,NMR}$ was calculated based on the following equation:

$$M_{n,NMR} = \frac{I_b}{I_a} MW^M + MW^{CTA}$$

For example:

$$M_{n,NMR} = 18.33 \times 306.38 + 310.41 = 5900$$

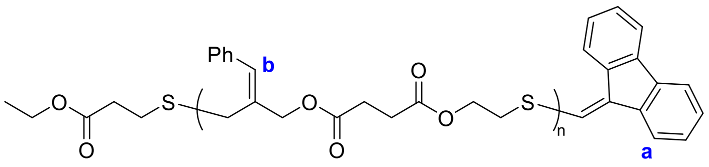

P3

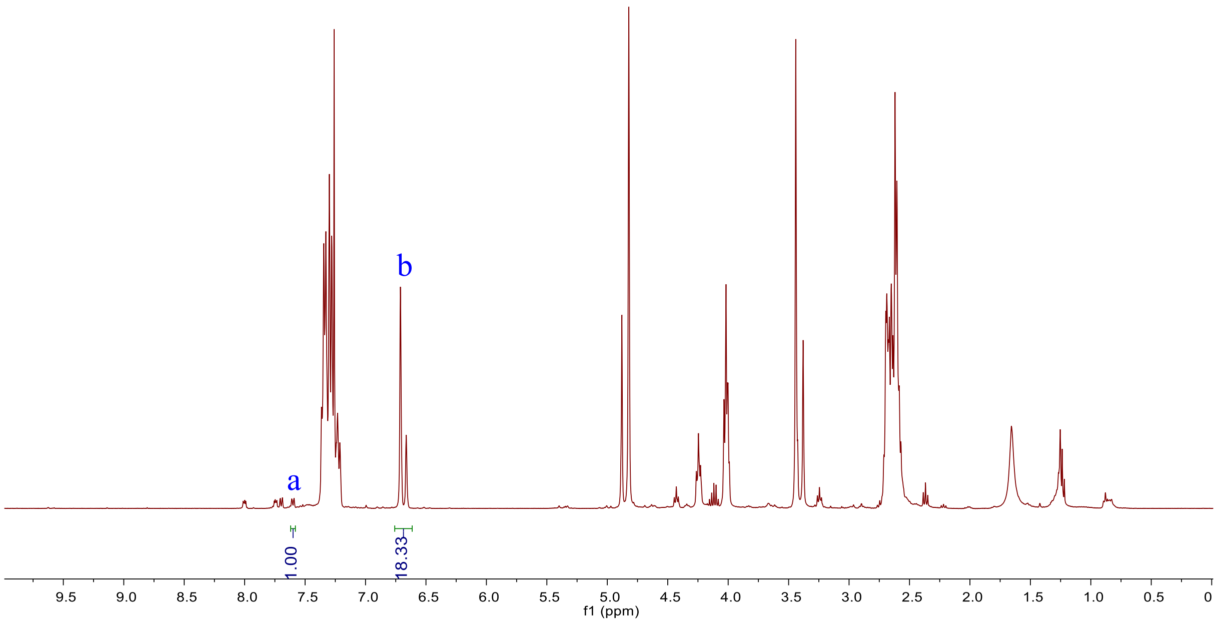

Figure S14. ^{1}H NMR ($CDCl_3$, 25°C) determination of molecular weight of **P3** (Table S7, entry 1).

Table S7. Polymerization of monomer **M3** at different monomer/CTA ratios.

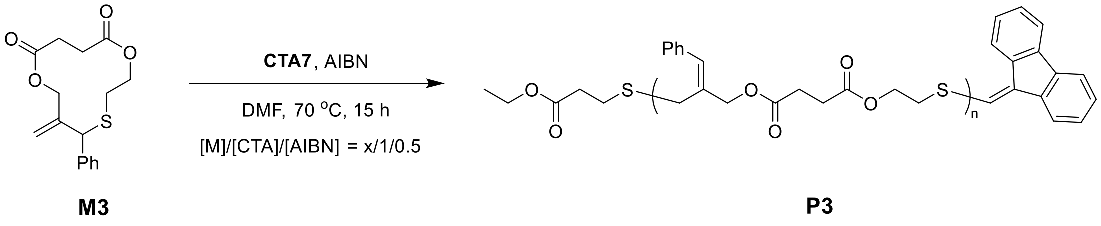

Entry[a]	[M]/[CTA]	Conversion[b]	$M_{n,theo}$[c]	$M_{n,NMR}$[d]	$M_{n,SEC}$[e]	$Đ$[e]
1	25/1	67%	5400	5900	5900	1.32
2	50/1	65%	10300	10900	9400	1.48
3	100/1	63%	19600	21900	14500	1.58

[a] Experimental conditions: [**M**] = 0.2 M, reacted at 70 °C for 15 h under a nitrogen atmosphere, unless otherwise noted. [b] Monomer conversion was determined by ^{1}H NMR analysis of the crude reaction mixture. [c] Theoretical molecular weight was calculated using the following equation: $M_{n\ theo}$= ([M]$_0$/[CTA]$_0$) × MW^M × conversion + MW^{CTA}, where [M]$_0$, [CTA]$_0$, MW^M and MW^{CTA} correspond to initial monomer concentration, initial CTA concentration, molar mass of monomer unit, and molar mass of CTA, respectively. [d] Molecular weight was determined by ^{1}H NMR analysis of the isolated polymers. [e] Molecular weight and polydispersity index ($Đ$) were determined by SEC analysis in THF at 40 °C using polystyrene standards.

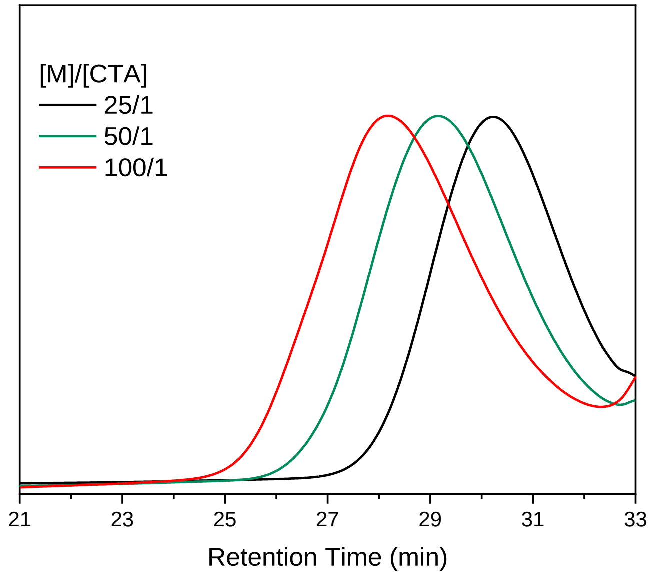

Figure S15. SEC traces for the polymerization of monomer **M3** at different monomer/CTA ratios (Table S7, entries 1–3).

Scheme S5. Kinetic study for the SRDP of **M1**.

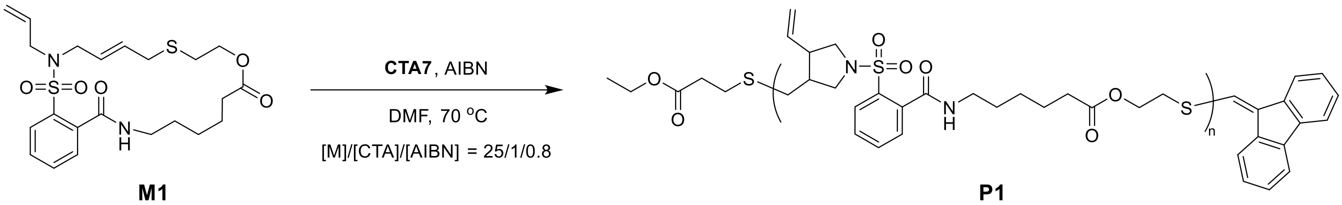

A 10 mL Schlenk vial was charged with monomer **M1** (93.3 mg, 0.2mmol), **CTA7** (0.04 M in DMF, 200 µL, 8.0 µmol), AIBN (0.04 M in DMF, 160 µL, 6.4 µmol) and DMF (640 µL). The vial was sealed, and the solution was deoxygenated by three freeze-pump-thaw cycles, followed by backfilling with nitrogen. The reaction mixture was then stirred at 70 °C, and aliquots were taken at various time intervals (t = 1, 2, 3, 4, 5.5, and 7 h). Monomer conversion was determined by ^{1}H NMR spectroscopy, while molecular weight and dispersity were analyzed by SEC.

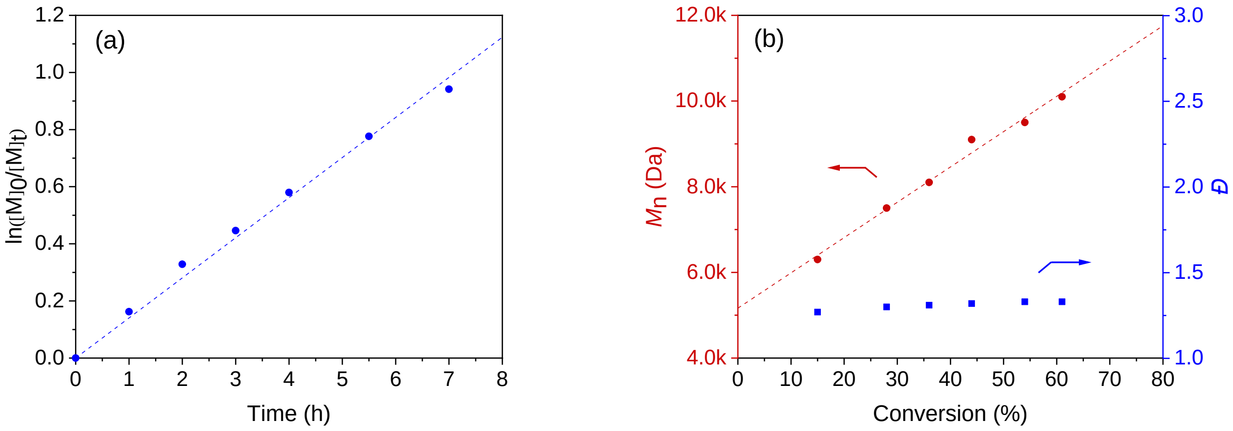

Figure S16. (a) Plot of ln([M]$_0$/[M]$_t$) versus reaction time for the SRDP of **M1**. (b) Plots of M_n and $Đ$ versus monomer conversion for the SRDP of **M1**.

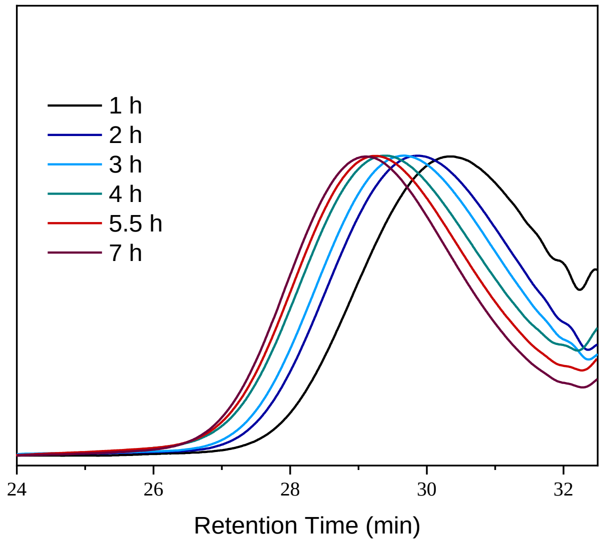

Figure S17. SEC traces for the kinetic study of the SRDP of **M1**.

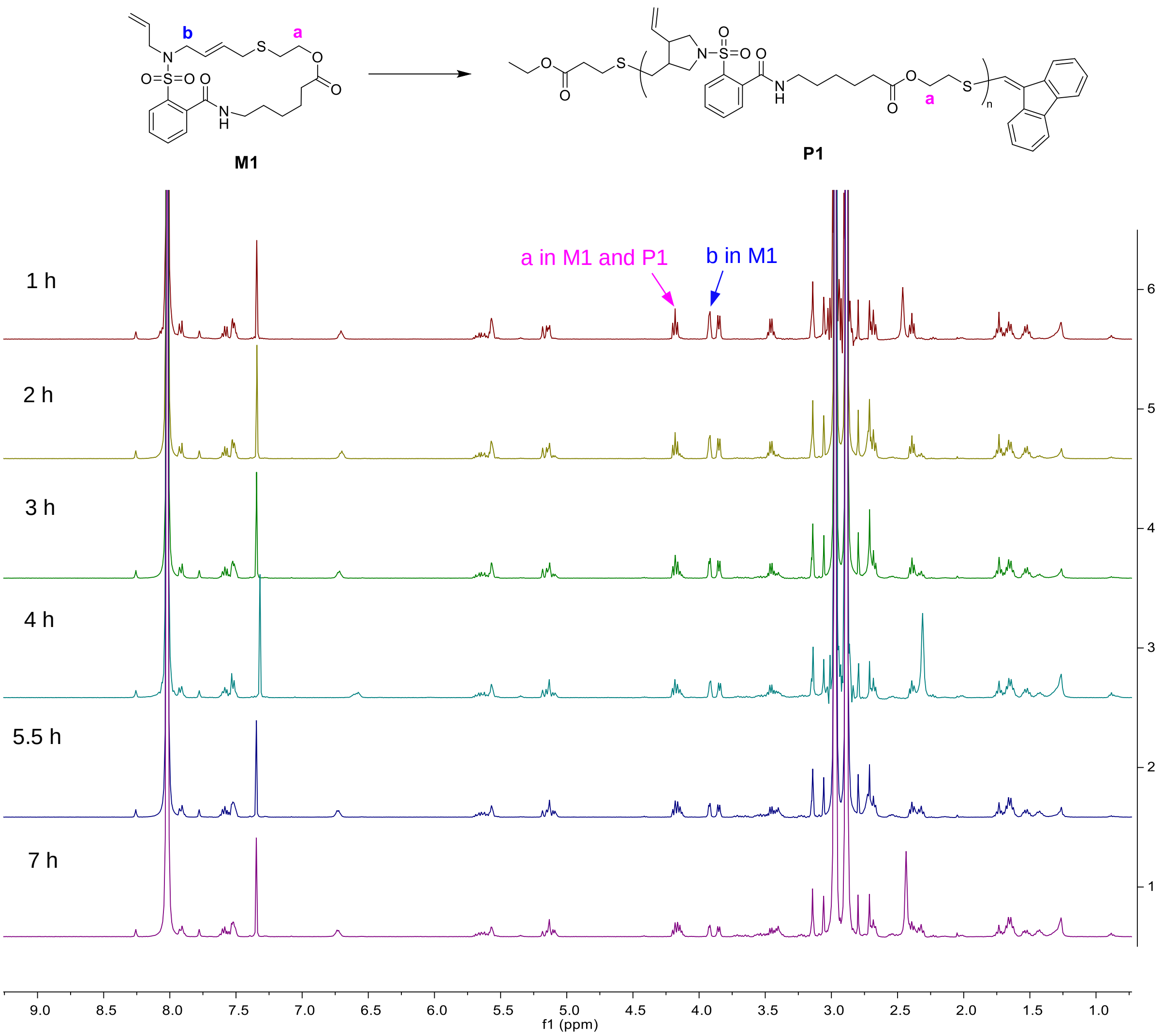

Figure S18. ^{1}H NMR ($CDCl_3$, 25°C) traces for the kinetic study of the SRDP of **M1**.

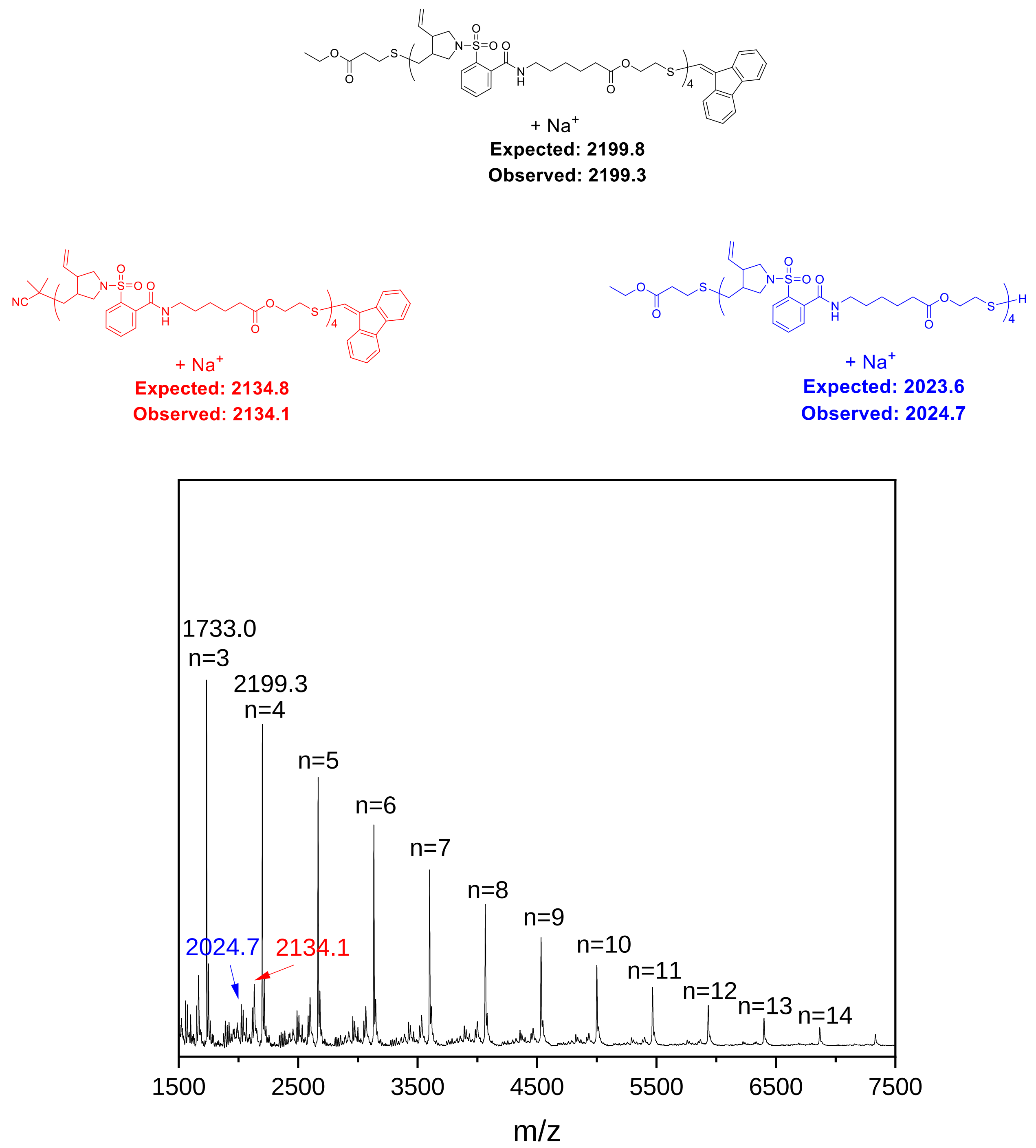

Figure S19. MALDI-TOF analysis of **P1**.

Scheme S6. Synthesis of diblock copolymer **P1-*b*-P2**.

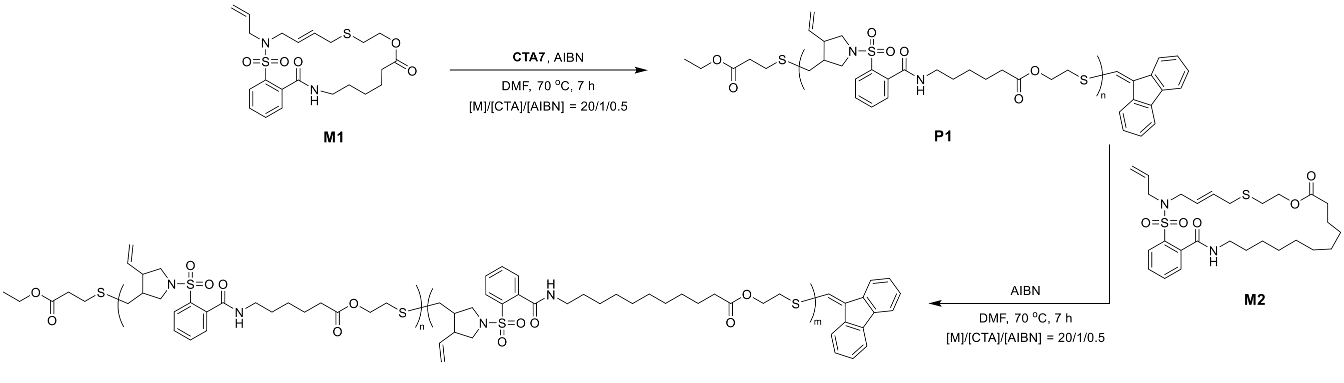

Following the general polymerization procedure, a 10 mL Schlenk vial was charged with monomer **M1** (46.7 mg, 0.1 mmol), followed by the addition of **CTA7** (0.04 M in DMF, 125 μL, 5.0 μmol), AIBN (0.02 M in DMF, 125 μL, 2.5 μmol), and DMF (750 μL). The vial was sealed, and the solution was deoxygenated via three freeze-pump-thaw cycles, backfilled with nitrogen, then heated at 70 °C for 7 h. The vial was cooled and opened to air to stop the polymerization. The monomer conversion was monitored by ^{1}H NMR spectroscopy (45% monomer conversion). The reaction mixture was precipitated twice with hexane, yielding the macroinitiator **P1** ($M_{n,SEC}$ = 6.7 kDa, *Đ* = 1.33).

A 10 mL Schlenk vial was charged with monomer M**2** (43.0 mg, 0.08 mmol), macroinitiator **P1** (4.0 μmol), AIBN (0.02 M in DMF, 100 μL, 2.0 μmol), and DMF (700 μL). The vial was sealed, and the solution was deoxygenated via three freeze-pump-thaw cycles, backfilled with nitrogen, then heated at 70 °C for 7 h. The vial was cooled and opened to air to stop the polymerization. The monomer conversion was monitored by ^{1}H NMR spectroscopy (42% monomer conversion). The reaction mixture was precipitated two times with hexane, yielding the polymer **P1-*b*-P2** ($M_{n,SEC}$ = 12.9 kDa, *Đ* = 1.48).

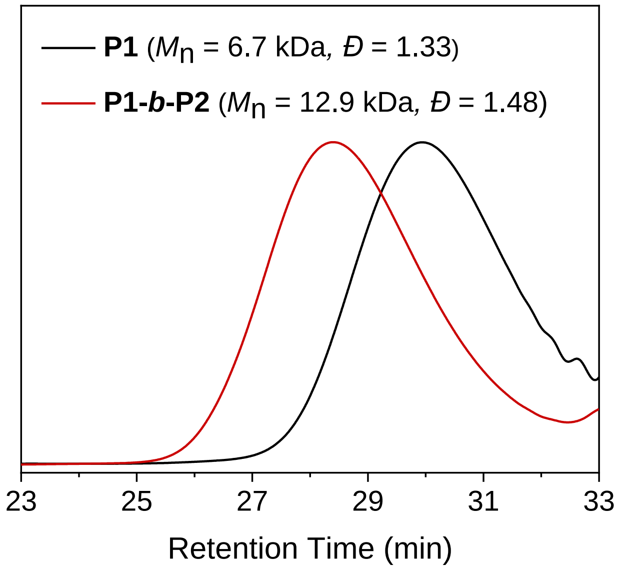

Figure S20. SEC traces for the synthesis of diblock copolymer **P1-*b*-P2**.

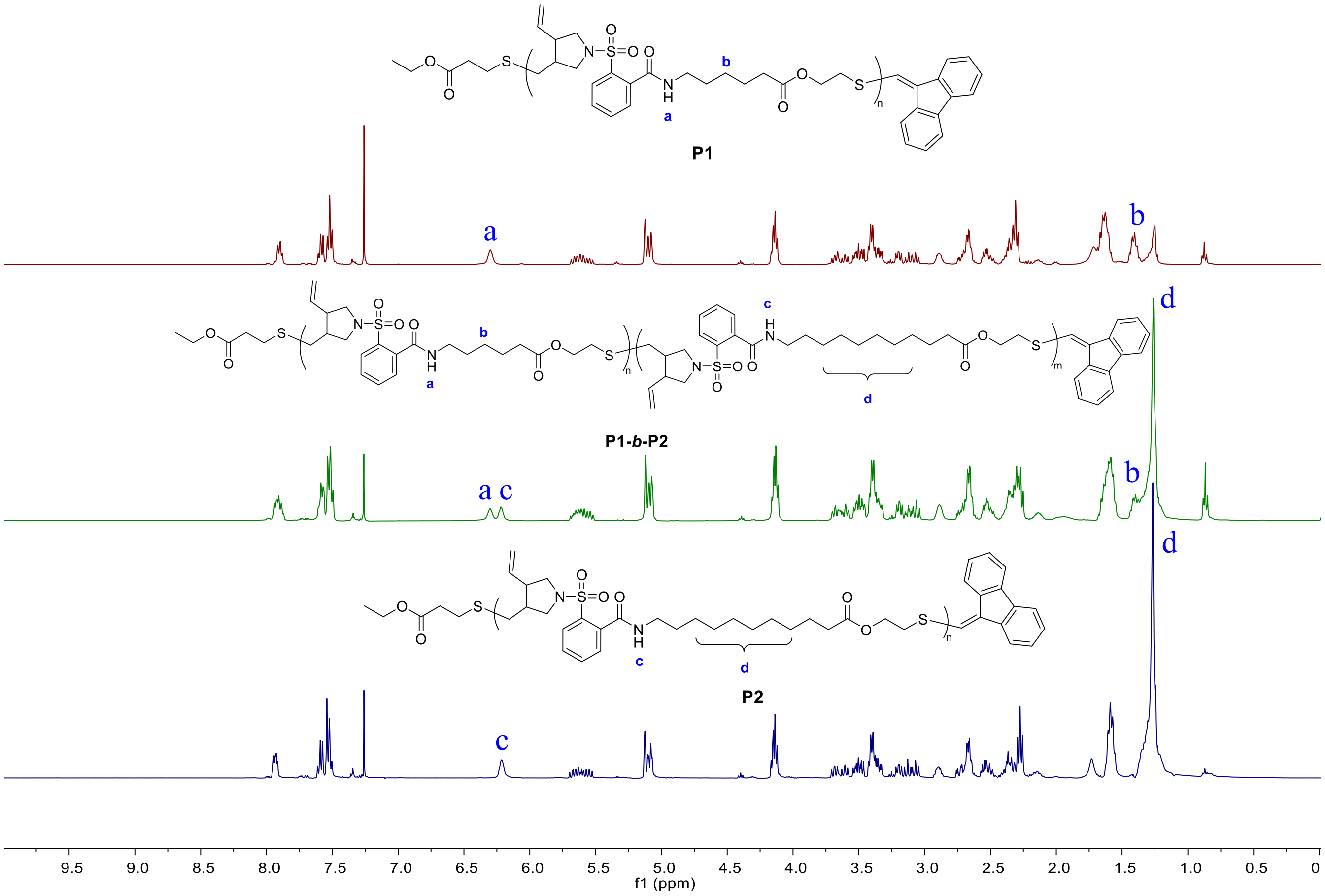

Figure S21. ^{1}H NMR ($CDCl_3$, 25°C) analysis of the diblock copolymer **P1-*b*-P2**.

DFT Calculations

(1) Initiation

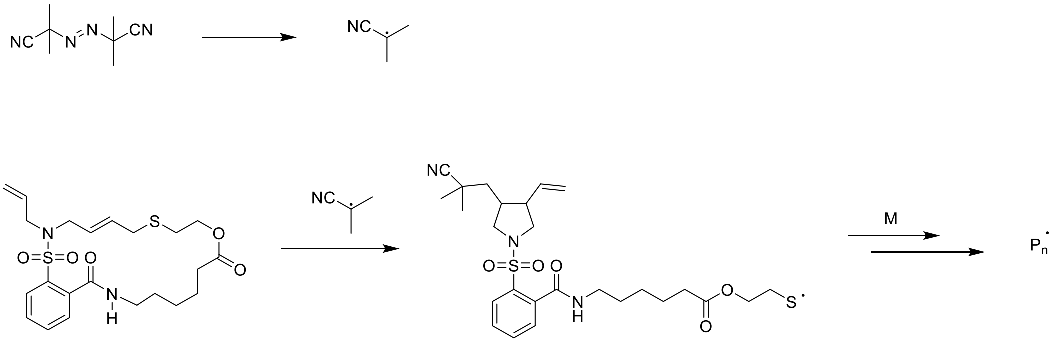

(2) Pre-equilibrium

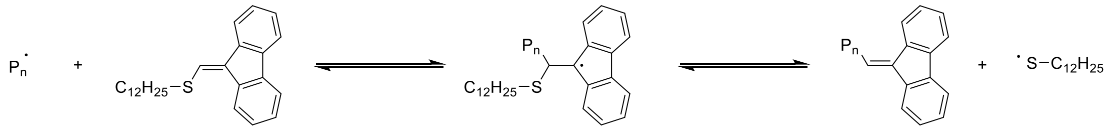

(3) Reinitiation

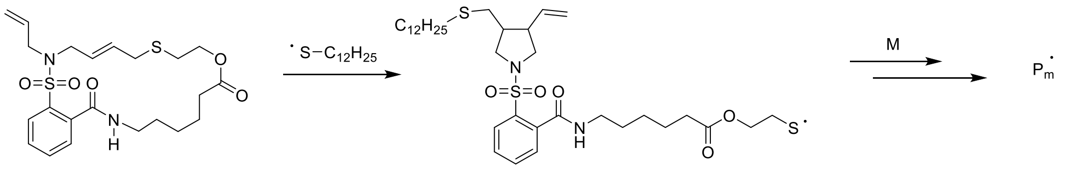

(4) Main equilibrium

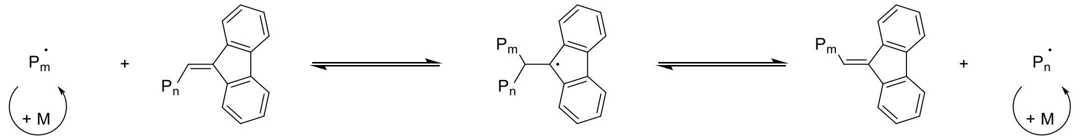

(5) Temination

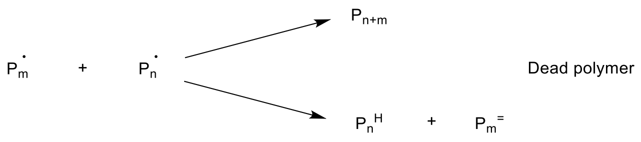

Figure S22. Proposed mechanism of the thiyl radical reversible-deactivation process.

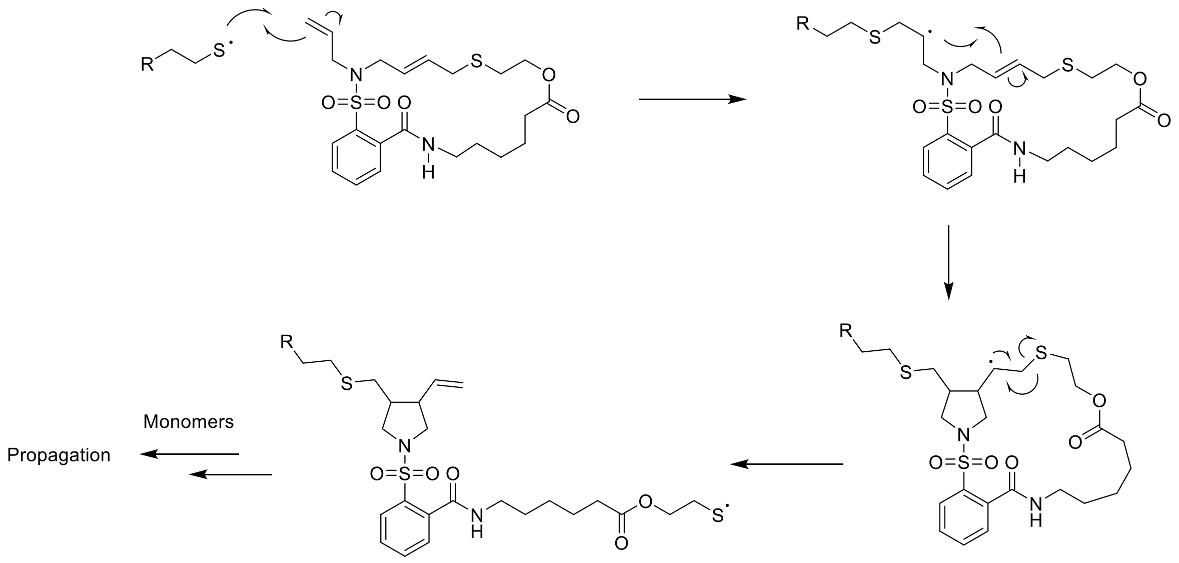

Figure S23. Proposed mechanism for the chain-growth process of the polymerization of **M1/M2**.

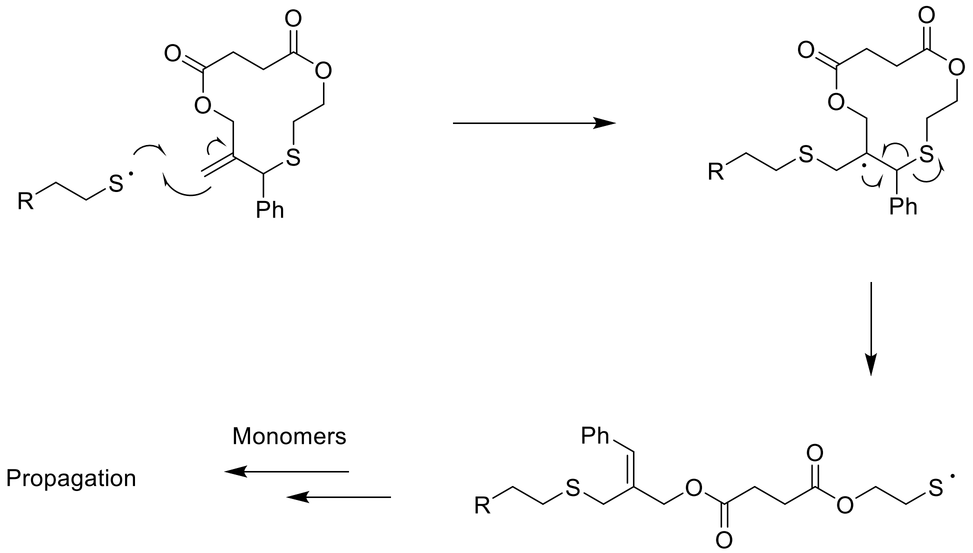

Figure S24. Proposed mechanism for the chain-growth process of the polymerization of **M3**.

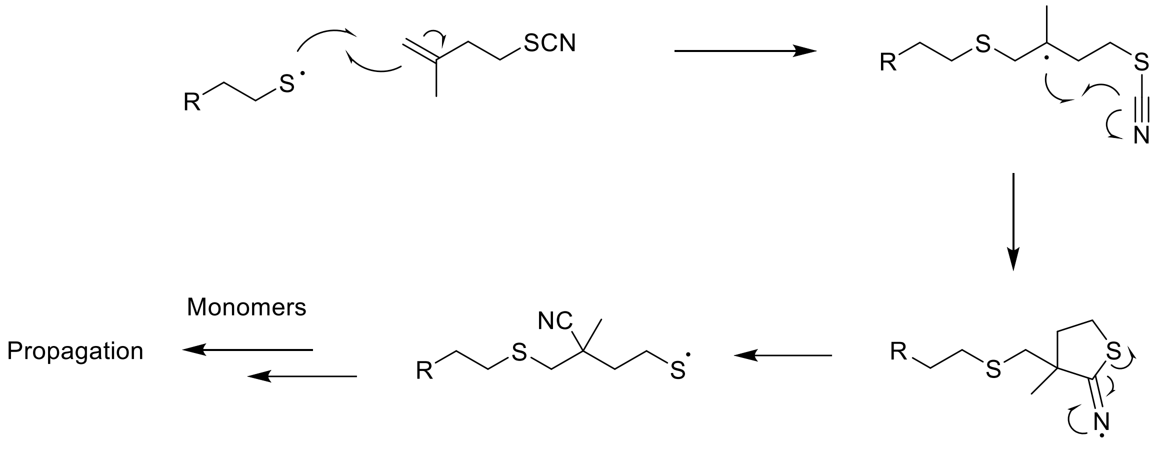

Figure S25. Proposed mechanism for the chain-growth process of the polymerization of **M4**.

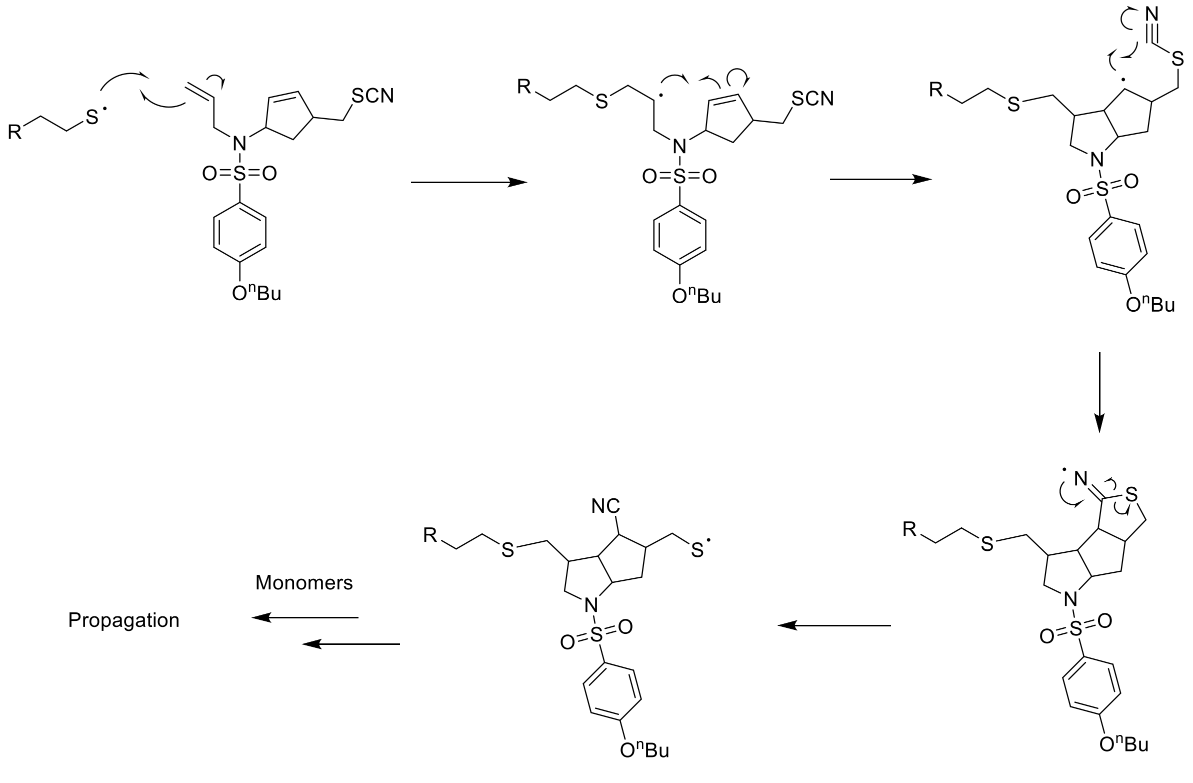

Figure S26. Proposed mechanism for the chain-growth process of the polymerization of **M5**.

Scheme S7. Calculation of rate and equilibrium constants.

The rate constants $k(T)$ were calculated using equation (Eq.1) according to the canonical variational transition state theory (CVT). The equilibrium constants $K(T)$ were calculated using equation (Eq.2). The Eq.1 has been inserted into an Excel file.[10] The rate constants could be calculated conveniently using this Excel.

Eyring's equation:

$$k(T) = \sigma \times \kappa \frac{k_B T}{h} (\frac{RT}{P^0})^{\Delta n} e^{-\frac{\Delta G^{0,\neq}(T)}{k_B T}} \qquad (1)$$

Vant't Hoff isotherm Equation:

$$K(T) = (c^0)^{\Delta n} e^{-\frac{\Delta G(T)}{RT}} \qquad (2)$$

Where h is Planck's constant (6.626×10^{-34} J·s), k_B is Boltzmann's constant (1.381×10^{-23} J/K), P^0 is the standard pressure (1 bar), $G^{0,\ddagger}(T)$ is the standard Gibbs free energy of activation, and Δn=n-1 for unimolecular or bimolecular reactions. σ is the reaction path degeneracy. κ is the tunneling correction factor, which was set to 1 due to the negligible tunneling effect for our reaction. $\Delta G(T)$ is the Gibbs free energy of reaction. c^0 is the standard unit of concentration (mol L^{-1}).

A calculation procedure for the addition rate constant of **TS3** is given below as an example. According to CVT, **TS3** and eight points close to it in the IRC were taken out and saved as Gaussian input files. Their frequencies were calculated at the M06-2X/def2-SVP level, and their single-point energies were calculated at the M06-2X/def2-TZVPP level. The calculations showed that the TS-1 structure has the maximum G. Next, the single-point energy of TS+1 was further calculated at the PWPB95-D3(BJ)/def2-TZVPP level and finally gave the ΔG_{add}^{CVT} of 49.33 KJ/mol.

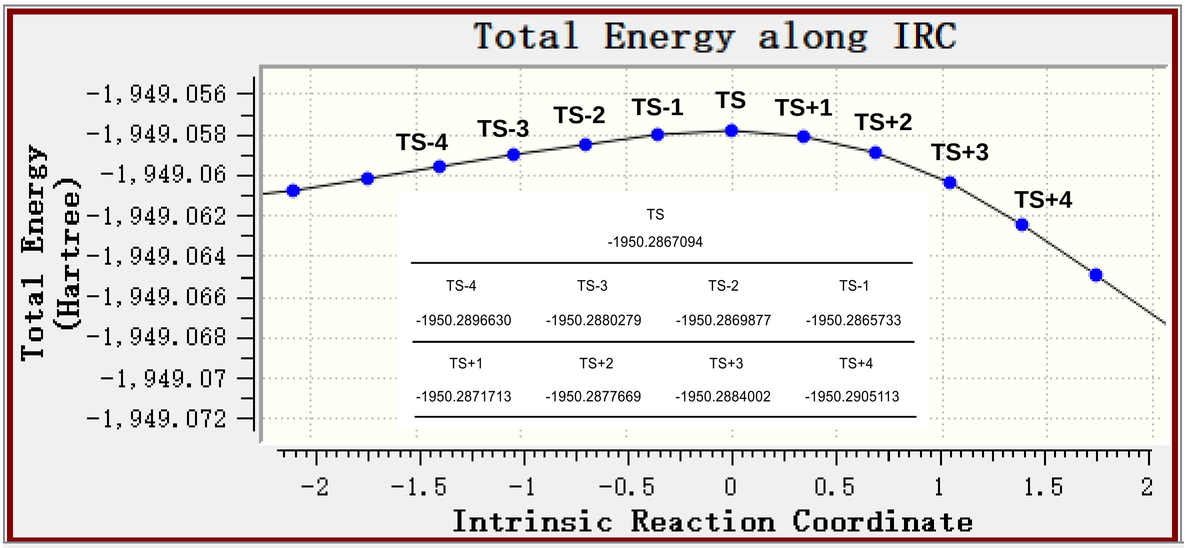

Thus,

$$k_{add} = \sigma \times \kappa \frac{k_B T}{h} \left(\frac{RT}{P^0}\right)^{\Delta n} e^{-\frac{\Delta G_{add}^{CVT}}{k_B T}} = 2.215 \times 10^5 \ \text{L mol}^{-1} \text{s}^{-1}$$

Table S8. Computed results for different CTA-Ps at the main equilibrium.

	Thermal correction to *G* (Hartree)	Single point energy (Hartree)	Imaginary frequency
**Int0**	0.073277	-705.135520212597	
**CTA1-P**	0.153393	-1010.98158004585	
**TS1**	0.247847	-1716.11841250139	347.9697i
**Int1**	0.249011	-1716.13264881997	
**CTA2-P**	0.187822	-1014.11276805460	
**TS2**	0.286402	-1719.25497255712	288.1589i
**Int2**	0.286242	-1719.27212214152	
**CTA3-P**	0.263578	-1245.08242811913	
**TS3**	0.362131	-1950.22622099538	318.6822i
**Int3**	0.362644	-1950.24035275661	
**CTA4-P**	0.244996	-1243.90203163566	
**TS4**	0.343119	-1949.04624286278	273.6293i
**Int4**	0.342733	-1949.06656681688	
**CTA5-P**	0.305370	-1472.89855586387	
**TS5**	0.402408	-2178.04267854449	281.5284i
**Int5**	0.402994	-2178.06272831875	
**M**	0.325079	-1813.27344164294	
**TS6**	0.424070	-2518.41096531706	320.8572i
**Int6**	0.419789	-2518.42701328254	

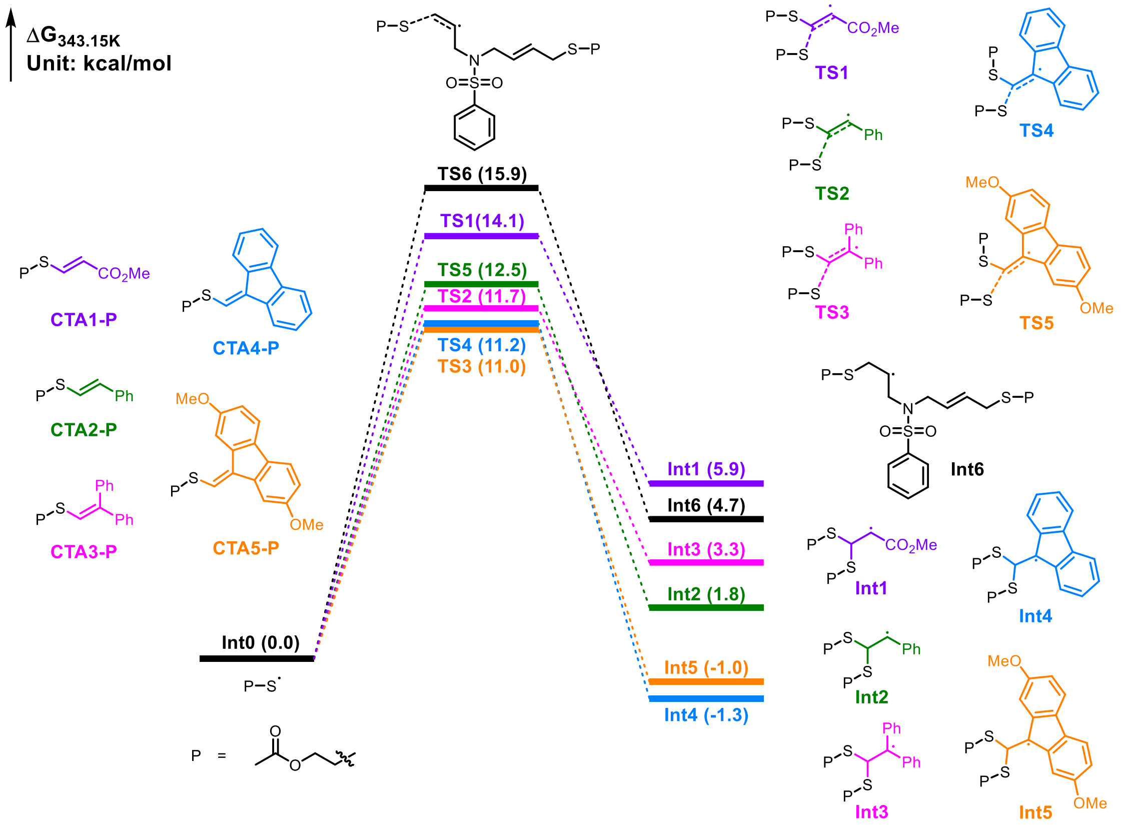

Figure S27. Calculated free energy profiles for different CTA-Ps at the main equilibrium.

Table S9. Calculated addition (k_{add}), elimination (k_{elim}) and equilibrium (K) constants for different CTA-Ps at the main equilibrium.

Entry	CTA-P	ΔG_{add}^{CVT} (KJ mol^{-1})	k_{add} (L mol^{-1} s^{-1})	ΔG_{elim}^{CVT} (KJ mol^{-1})	k_{elim} (s^{-1})	K (L mol^{-1})
1	**CTA1-P**	59.16	7.065×10^3	34.33	4.253×10^7	1.661×10^{-4}
2	**CTA2-P**	52.66	6.895×10^4	44.93	1.036×10^6	6.655×10^{-2}
3	**CTA3-P**	49.33	2.215×10^5	35.41	2.913×10^7	7.604×10^{-3}
4	**CTA4-P**	46.69	5.588×10^5	52.27	7.905×10^4	7.069
5	**CTA5-P**	45.99	7.142×10^5	50.29	1.582×10^5	4.515

Table S10. Computed results for different original CTAs at the pre-equilibrium and reinitiation.

	Thermal correction to *G* (Hartree)	Single point energy (Hartree)	Imaginary frequency
**Int0**	0.073277	-705.135520212597	
**A**	0.261635	-1094.65375612391	
**A-TS1**	0.359245	-1799.79825262757	270.0876i
**A-Int1**	0.359514	-1799.81788220963	
**A-TS2**	0.358408	-1799.79513255896	293.4665i
**A-Int2**	0.089728	-555.884458940861	
**A-TS3**	0.438925	-2369.16093614893	334.1045i
**B**	0.218707	-1204.59994830958	
**B-TS1**	0.317616	-1909.74472900610	262.2628i
**B-Int1**	0.316829	-1909.76505200031	
**B-TS2**	0.318280	-1909.74478491674	260.8236i
**B-Int2**	0.047052	-665.831400365833	
**B-TS3**	0.396086	-2479.11038807666	291.6342i
**C**	0.246134	-1243.89952115212	
**C-TS1**	0.343210	-1949.04289174230	274.0615i
**C-Int1**	0.344092	-1949.06392679866	
**C-TS2**	0.344918	-1949.04022094872	296.8599i
**C-Int2**	0.075310	-705.132822265759	
**C-TS3**	0.423298	-2518.40889647191	320.7717i
**D**	0.244996	-1243.90203163566	
**D-TS1**	0.343119	-1949.04624286278	273.6293i
**D-Int1**	0.342733	-1949.06656681688	
**D-TS2**	0.343115	-1949.046245118290	273.5972i
**D-Int2**	0.073277	-705.135520212597	
**D-TS3**	0.424070	-2518.41096531706	320.8572i
**E**	0.256707	-1207.73547389891	
**E-TS1**	0.353768	-1912.87852280089	301.5061i
**E-Int1**	0.354014	-1912.90121039011	
**E-TS2**	0.353584	-1912.88997440438	311.6577i
**E-Int2**	0.084824	-668.981045418844	
**E-TS3**	0.432837	-2482.25195385572	363.1901i

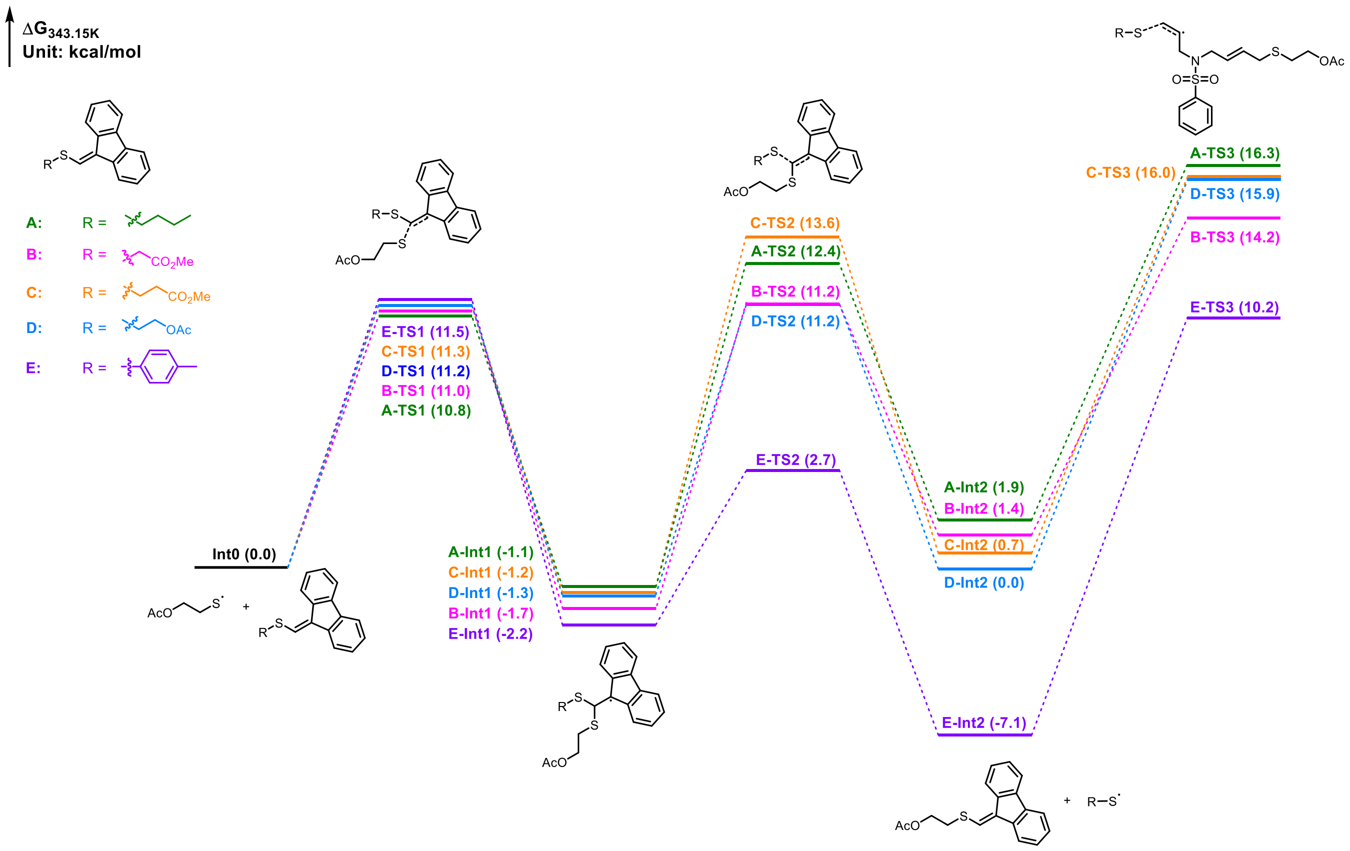

Figure S28. Calculated free energy profiles for different original CTAs at the pre-equilibrium.

NMR analysis of the conversion of monomer M4

Monomer conversion was determined based on the assumption that the integral of peak a at δ = 1.90 ppm corresponds to methylene groups present in polymers. In contrast, the integral of peak b at δ = 4.90 ppm corresponds to the alkenyl group found in unreacted monomers. By normalizing the integral of peak b to 1, the monomer conversion (α) was calculated using the following equation:

$$\alpha = \left(\frac{I_a}{I_a + I_b}\right) \times 100\%$$

For example:

$$\alpha = [62.59 \div (1+62.59)] \times 100\% = 98\%$$

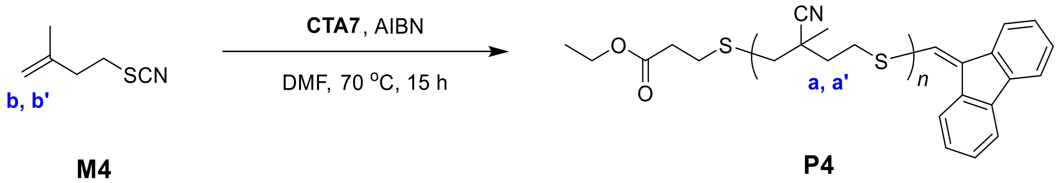

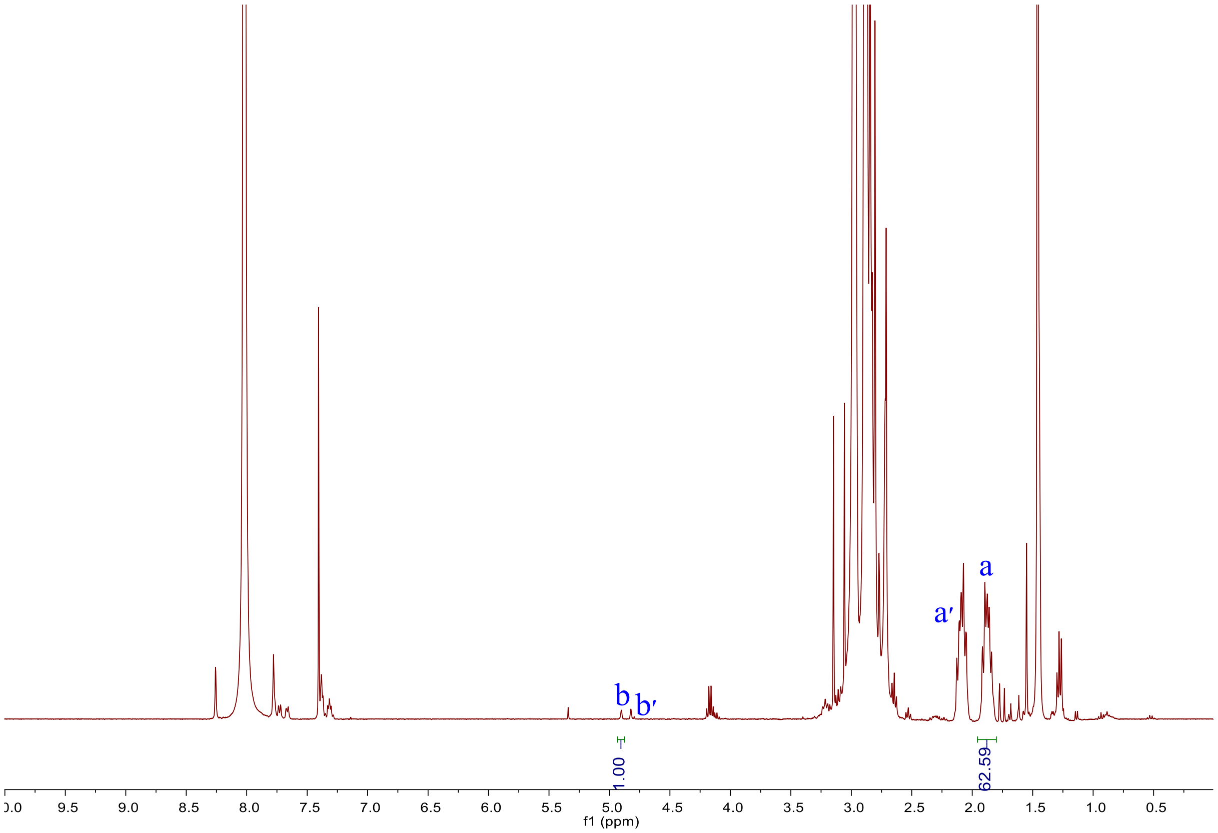

Figure S29. ^{1}H NMR ($CDCl_3$, 25°C) determination of monomer conversion for the polymerization of monomer **M4** (Table S11, entry 2).

NMR analysis of the molecular weight of P4

The determination of $M_{n,NMR}$ for the polymers was based on the assumption that the integral of the peak at δ = 4.12 ppm (Peak a) corresponds to the methylene group of the polymer chain-end, and the integral of the peak at δ = 1.84 ppm (Peak b) corresponds to the methylene group of the polymer repeating unit. By normalizing the integral of Peak a to 2, the $M_{n,NMR}$ was calculated based on the following equation:

$$M_{n,NMR} = \frac{2I_b}{I_a} MW^M + MW^{CTA}$$

For example:

$M_{n,NMR}$ = 25.83×127.21+310.41 = 3600

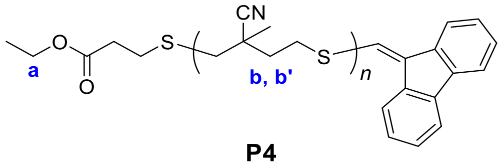

P4

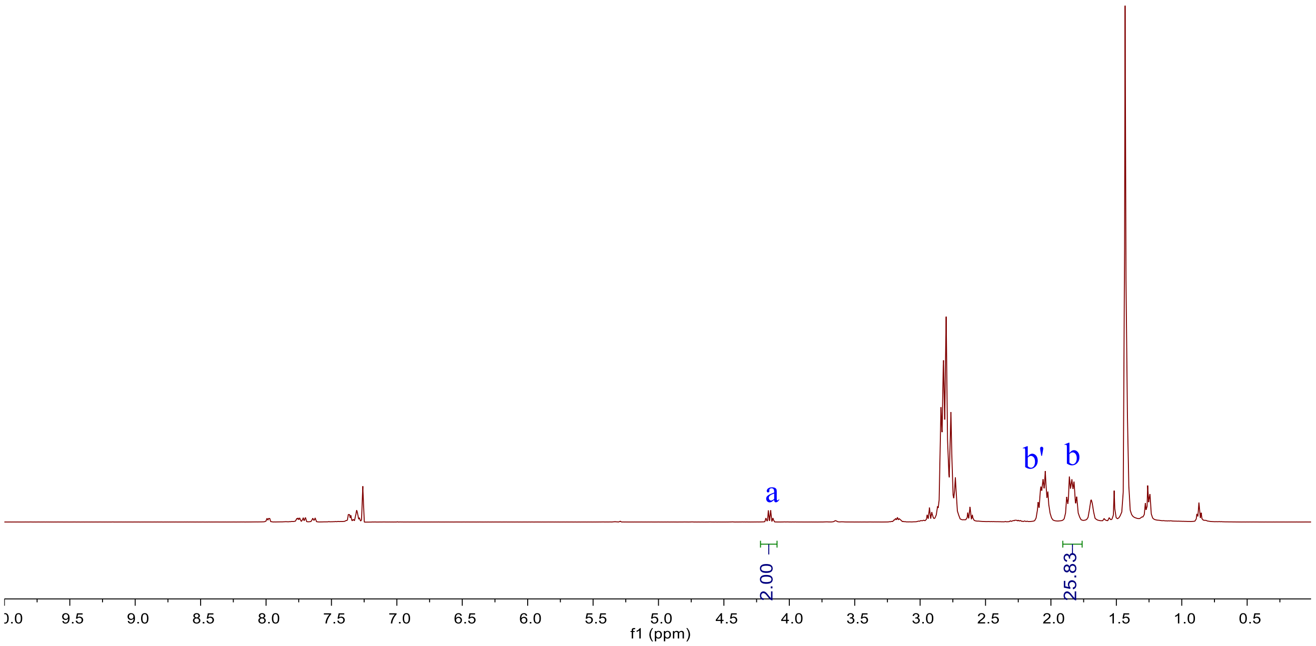

Figure S30. ^{1}H NMR ($CDCl_3$, 25°C) determination of molecular weight of **P4** (Table S11, entry 1).

Table S11. Polymerization of monomer **M4** at different monomer/CTA ratios.

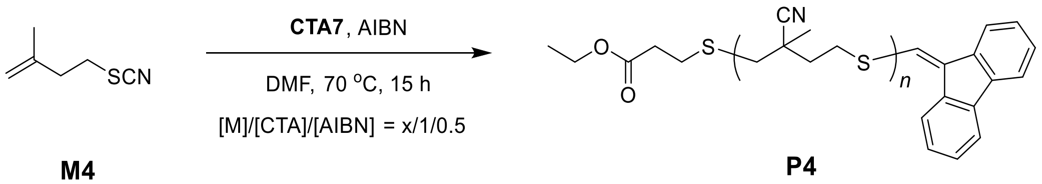

M4 **P4**

Entry[a]	[M]/[CTA]	Conversion[b]	$M_{n,theo}$[c]	$M_{n,NMR}$[d]	$M_{n,SEC}$[e]	$Đ$[e]
1	25/1	99%	3500	3600	5000	1.24
2	50/1	98%	6500	7400	9400	1.26
3	100/1	>99%	13000	16700	16700	1.33

[a] Experimental conditions: [**M**] = 0.8 M, reacted at 70 °C for 15 h under a nitrogen atmosphere, unless otherwise noted. [b] Monomer conversion was determined by ^{1}H NMR analysis of the crude reaction mixture. [c] Theoretical molecular weight was calculated using the following equation: $M_{n\ theo}$= ([M]$_0$/[CTA]$_0$) × MW^M × conversion + MW^{CTA}, where [M]$_0$, [CTA]$_0$, MW^M and MW^{CTA} correspond to initial monomer concentration, initial CTA concentration, molar mass of monomer unit, and molar mass of CTA, respectively. [d] Molecular weight was determined by ^{1}H NMR analysis of the isolated polymers. [e] Molecular weight and polydispersity index ($Đ$) were determined by SEC analysis in THF at 40 °C using polystyrene standards.

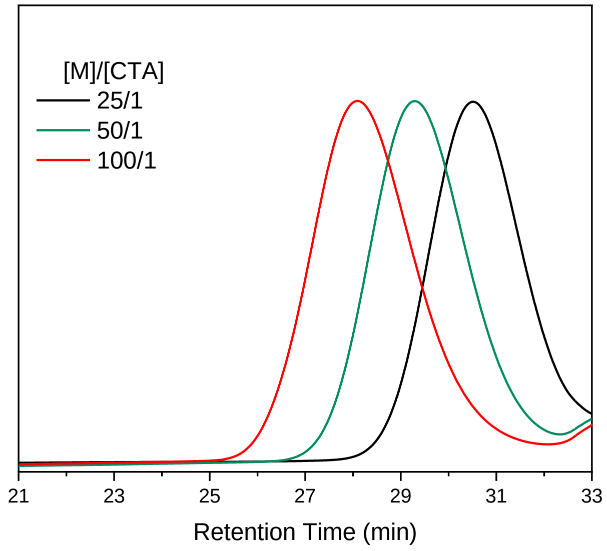

Figure S31. SEC traces for the polymerization of monomer **M4** at different monomer/CTA ratios (Table S11, entries 1–3).

NMR analysis of the conversion of monomer M5

Monomer conversion was determined based on the assumption that the integral of peak a at δ = 6.86 ppm corresponds to phenyl groups present in both monomers and polymers. In contrast, the integral of peak b at δ = 5.39 ppm corresponds to an alkenyl group found in unreacted monomers. By normalizing the integral of peak b to 1, the monomer conversion (α) was calculated using the following equation:

$$\alpha = \left(1 - \frac{2I_b}{I_a}\right) \times 100\%$$

For example:

α = [1-(2×1÷4.70)] ×100% = 57%

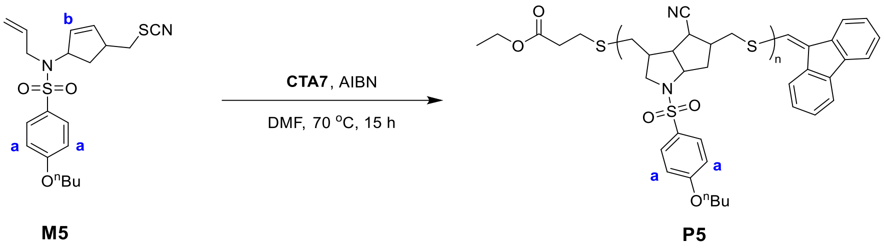

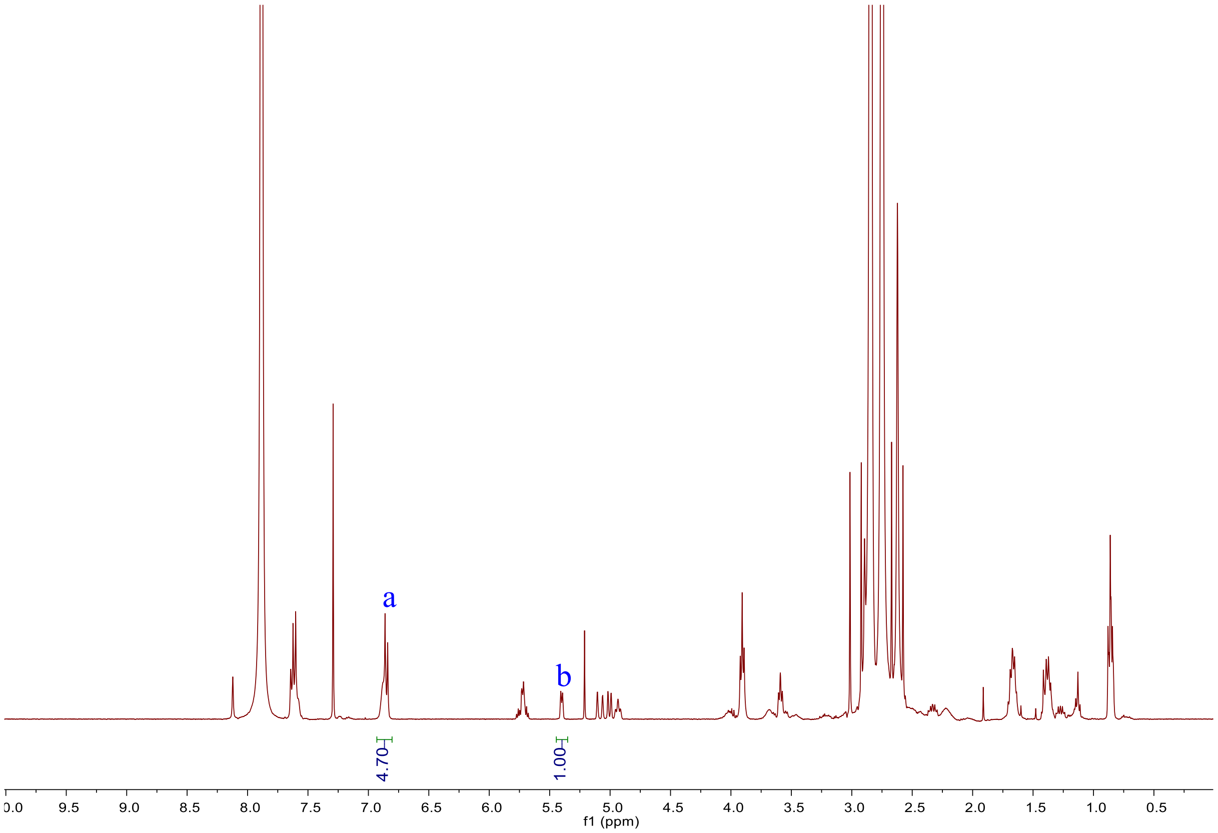

Figure S32. ^{1}H NMR ($CDCl_3$, 25°C) determination of monomer conversion for the polymerization of monomer **M5** (Table S12, entry 1).

NMR analysis of the molecular weight of P5

The determination of $M_{n,NMR}$ for the polymers was based on the assumption that the integral of the peak at δ = 7.62 ppm (Peak a) corresponds to the phenyl group of the polymer chain-end, and the integral of the peak at δ = 6.98 ppm (Peak b) corresponds to the phenyl group of the polymer repeating unit. By normalizing the integral of Peak a to 1, the $M_{n,NMR}$ was calculated based on the following equation:

$$M_{n,NMR} = \frac{I_b}{2I_a} MW^M + MW^{CTA}$$

For example:

$M_{n,NMR}$ = 38.27÷2×406.56+310.41 = 8100

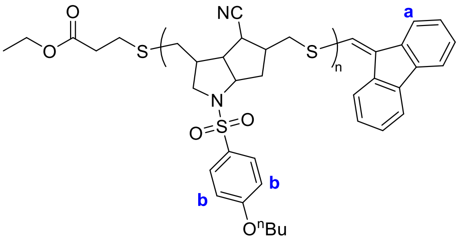

P5

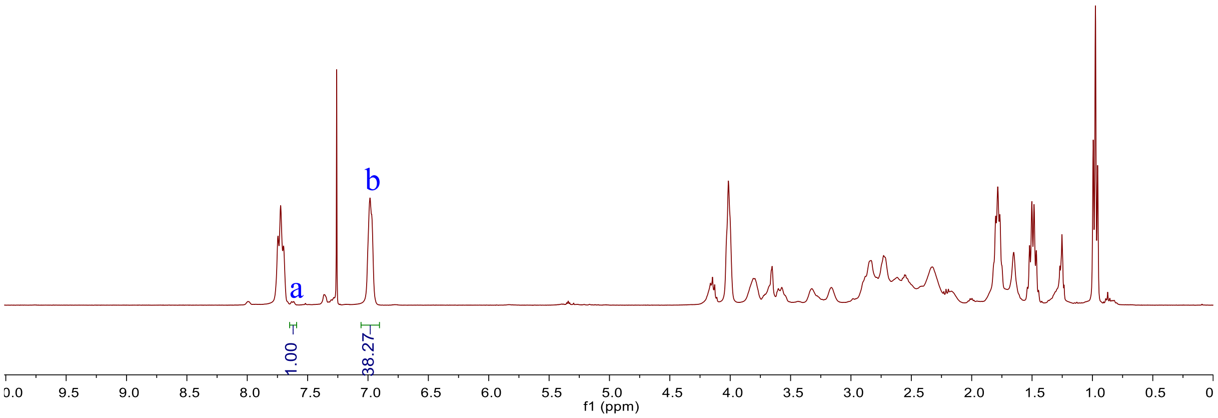

Figure S33. ^{1}H NMR ($CDCl_3$, 25°C) determination of molecular weight of **P5** (Table S12, entry 1).

Table S12. Polymerization of monomer **M5** at different monomer/CTA ratios.

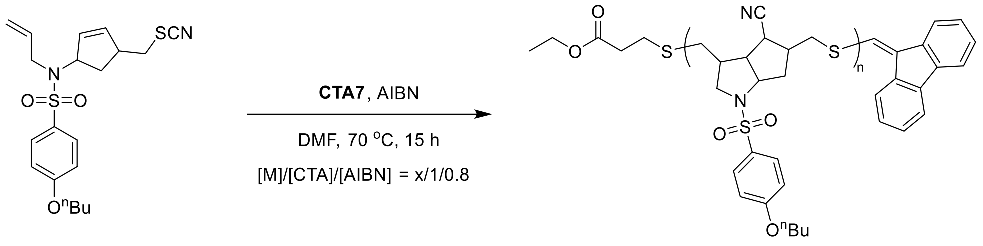

M5 **P5**

Entry[a]	[M]/[CTA]	Conversion[b]	$M_{n,theo}$[c]	$M_{n,NMR}$[d]	$M_{n,SEC}$[e]	$Đ$[e]
1	25/1	57%	6100	8100	5200	1.20
2	50/1	52%	10900	13800	6500	1.26
3	100/1	49%	20200	24200	7900	1.30

[a] Experimental conditions: [**M**] = 0.2 M, reacted at 70 °C for 15 h under a nitrogen atmosphere, unless otherwise noted. [b] Monomer conversion was determined by ^{1}H NMR analysis of the crude reaction mixture. [c] Theoretical molecular weight was calculated using the following equation: $M_{n\ theo}$= ([M]$_0$/[CTA]$_0$) × MW^M × conversion + MW^{CTA}, where [M]$_0$, [CTA]$_0$, MW^M and MW^{CTA} correspond to initial monomer concentration, initial CTA concentration, molar mass of monomer unit, and molar mass of CTA, respectively. [d] Molecular weight was determined by ^{1}H NMR analysis of the isolated polymers. [e] Molecular weight and polydispersity index ($Đ$) were determined by SEC analysis in THF at 40 °C using polystyrene standards.

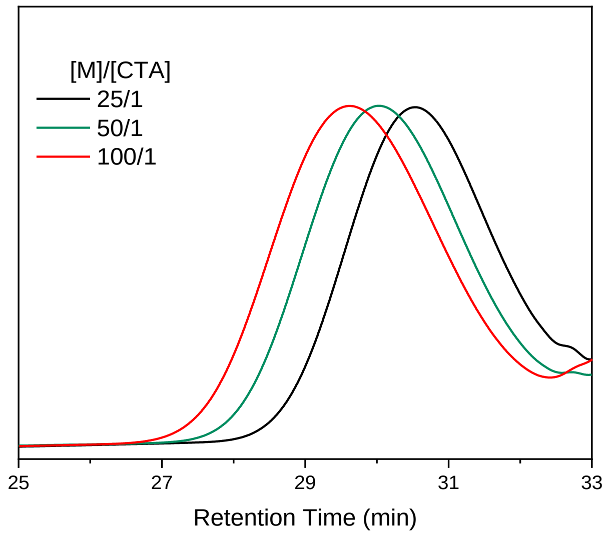

Figure S34. SEC traces for the polymerization of monomer **M5** at different monomer/CTA ratios (Table S12, entries 1–3).

Scheme S8. Procedure for calculating the chain transfer coefficient of the **M4/CTA7** combination.

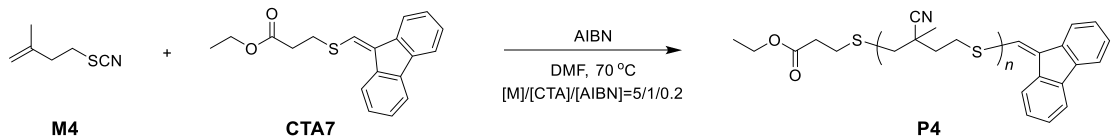

A 10 mL Schlenk vial was charged with **M4** (0.5 mmol), **CTA7** (0.1 mmol), AIBN (0.02 mmol), and DMF (2.5 mL). The vial was sealed, and the solution was deoxygenated by three freeze-pump-thaw cycles, then backfilled with nitrogen. The reaction mixture was stirred at 70°C, and aliquots were taken at various time points to measure the conversion of the monomer and CTA using ^{1}H NMR spectroscopy. The apparent chain transfer coefficient was determined from the slope of a double logarithmic plot of CTA concentration versus monomer concentration (Figures S35 and S36).[11]

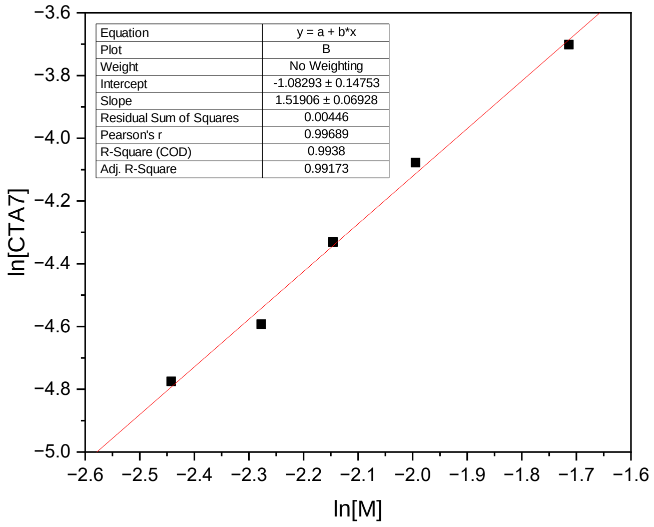

Figure S35. Calculated chain transfer coefficient of the **M4/CTA7** combination.

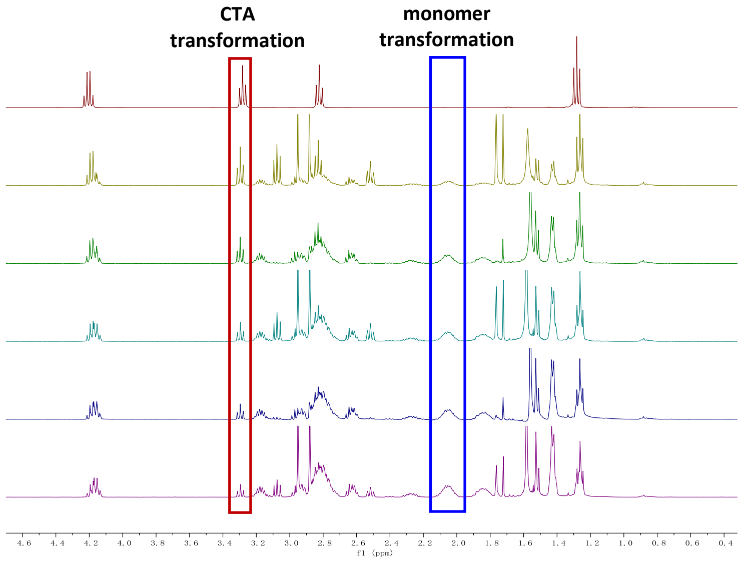

Figure S36. ^{1}H NMR ($CDCl_3$, 25°C) traces for the calculated chain transfer coefficient of the **M4/CTA7** combination.

Scheme S9. Kinetic study for the SRDP of **M4**.

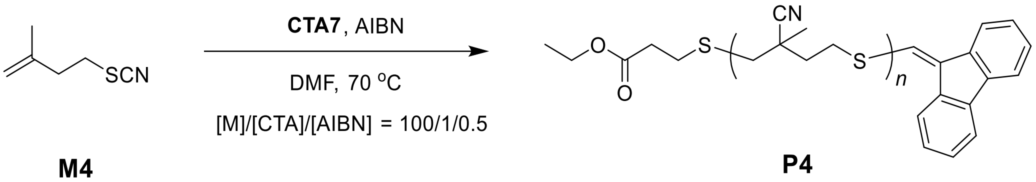

M4 **P4**

A 10 mL Schlenk vial was charged with monomer **M4** (50.9 mg, 0.4 mmol), **CTA7** (0.04 M in DMF, 100 µL, 4.0 µmol), AIBN (0.02 M in DMF, 100 µL, 2.0 µmol) and DMF (800 µL). The vial was sealed, and the solution was deoxygenated by three freeze-pump-thaw cycles, followed by backfilling with nitrogen. The reaction mixture was then stirred at 70 °C, and aliquots were taken at various time intervals (t = 20, 40, 60, 90, 120, 150 min). Monomer conversion was determined by ^{1}H NMR spectroscopy, while molecular weight and dispersity were analyzed by SEC.

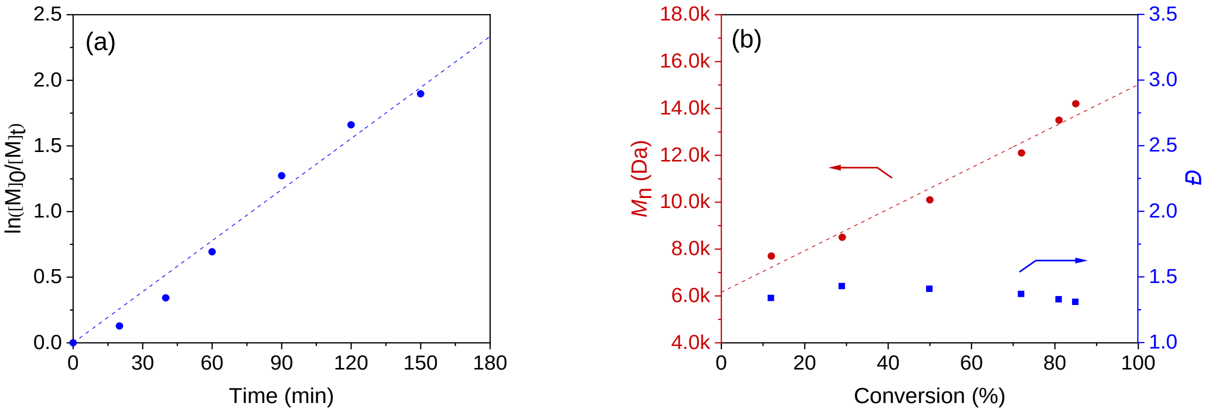

Figure S37. (a) Plot of ln([M]$_0$/[M]$_t$) versus reaction time for the SRDP of **M4**. (b) Plots of M_n and $Đ$ versus monomer conversion for the SRDP of **M4**.

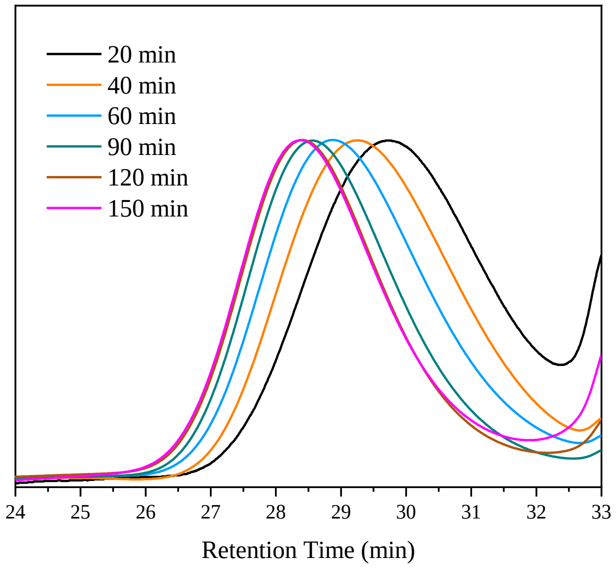

Figure S38. SEC traces for the kinetic study of the SRDP of **M4**.

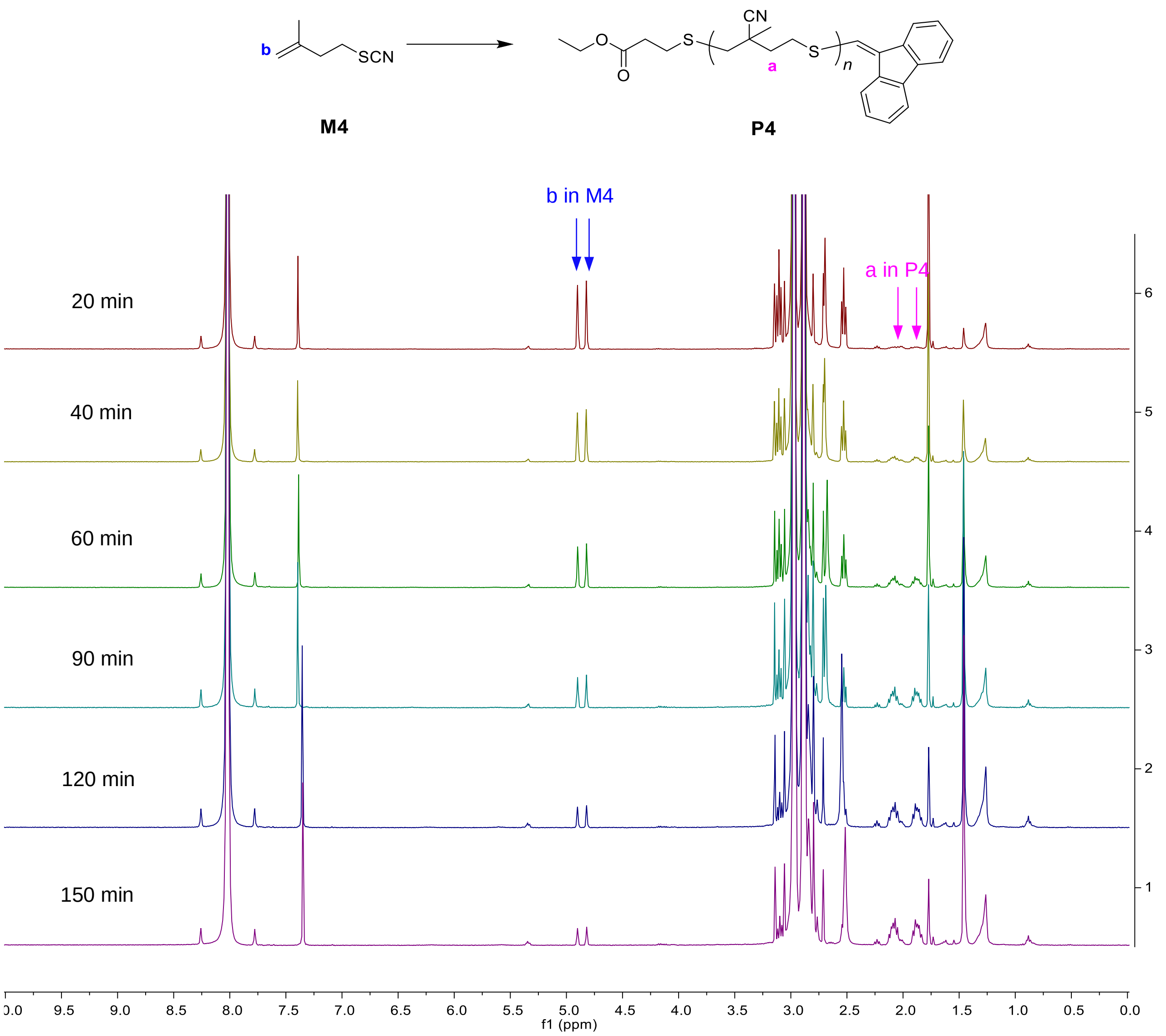

Figure S39. ^{1}H NMR ($CDCl_3$, 25°C) traces for the kinetic study of the SRDP of **M4**.

Scheme S10. Polymer synthesis for MALDI-TOF analysis.

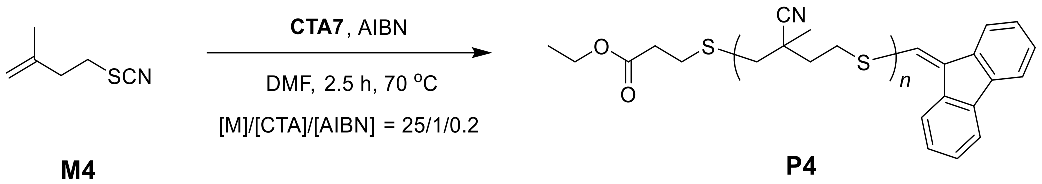

M4 **P4**

Following the general polymerization procedure, a 10 mL Schlenk vial was charged with monomer **M4** (50.9 mg, 0.4 mmol), **CTA7** (0.04 M in DMF, 400 μL, 16.0 μmol), AIBN (0.04 M in DMF, 160 μL, 3.2 μmol) and DMF (440 μL). The vial was sealed, and the solution was deoxygenated via three freeze-pump-thaw cycles, backfilled with nitrogen, then heated at 70 °C for 2.5 h. The vial was cooled and opened to air to stop the polymerization. The monomer conversion was monitored by ^{1}H NMR spectroscopy (54% monomer conversion). The reaction mixture was diluted with a minimum amount of DCM and precipitated with hexane. The obtained solid was re-dissolved with a minimum amount of DCM for further precipitation, yielding the polymer **P4**, which was then characterized using SEC ($M_{n,SEC}$ = 3400, $Đ$ = 1.23) and MALDI-TOF.

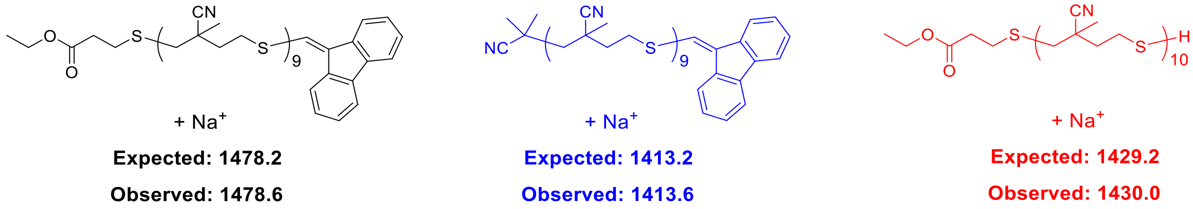

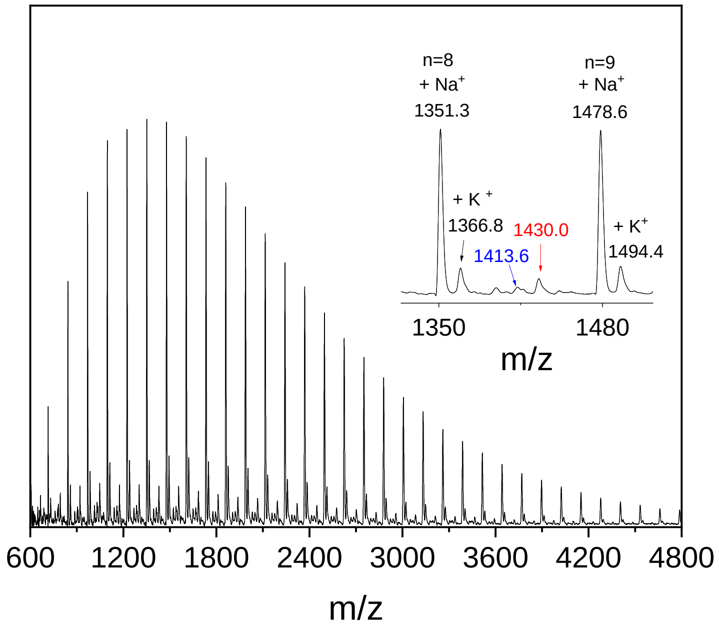

Figure S40. MALDI-TOF analysis of polymer **P4**.

Scheme S11. Synthesis of diblock copolymer **P4-*b*-P1**.

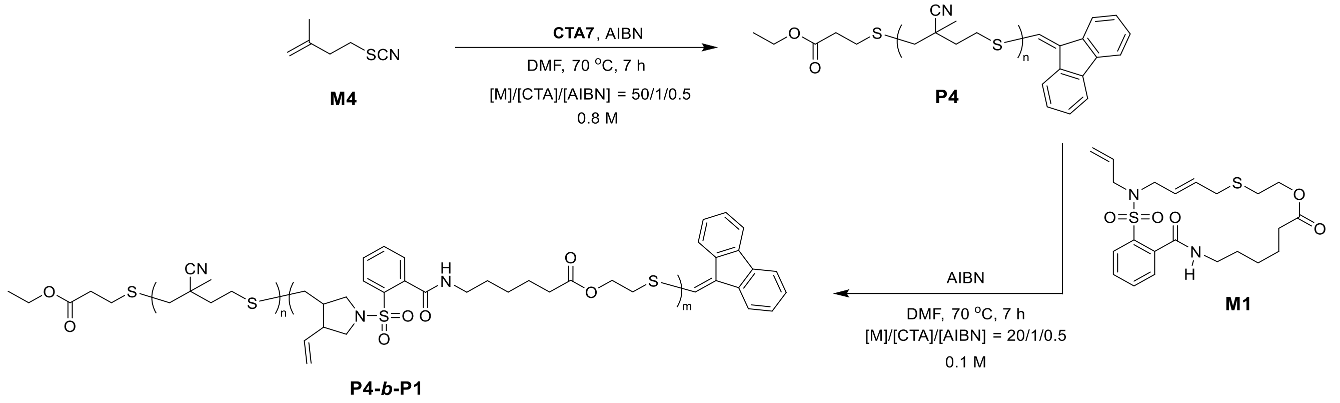

Following the general polymerization procedure, a 10 mL Schlenk vial was charged with monomer **M4** (50.9 mg, 0.4 mmol), followed by the addition of **CTA7** (0.04 M in DMF, 200 μL, 8.0 μmol), AIBN (0.02 M in DMF, 200 μL, 4.0 μmol), and DMF (100 μL). The vial was sealed, and the solution was deoxygenated via three freeze-pump-thaw cycles, backfilled with nitrogen, then heated at 70 °C for 7 h. The vial was cooled and opened to air to stop the polymerization. The monomer conversion was monitored by ^{1}H NMR spectroscopy (98% monomer conversion). The reaction mixture was precipitated twice with hexane, yielding the macroinitiator **P4** ($M_{n,SEC}$ = 9.8 kDa, $Đ$ =1.27).

A 10 mL Schlenk vial was charged with monomer **M1** (46.7 mg, 0.1 mmol), macroinitiator **P4** (5.0 μmol), AIBN (0.02 M in DMF, 125 μL, 2.5 μmol), and DMF (875 μL). The vial was sealed, and the solution was deoxygenated via three freeze-pump-thaw cycles, backfilled with nitrogen, then heated at 70 °C for 7 h. The vial was cooled and opened to air to stop the polymerization. The monomer conversion was monitored by ^{1}H NMR spectroscopy (61% monomer conversion). The reaction mixture was precipitated two times with hexane, yielding the polymer **P4-*b*-P1** ($M_{n,SEC}$ = 16.1 kDa, $Đ$ =1.31).

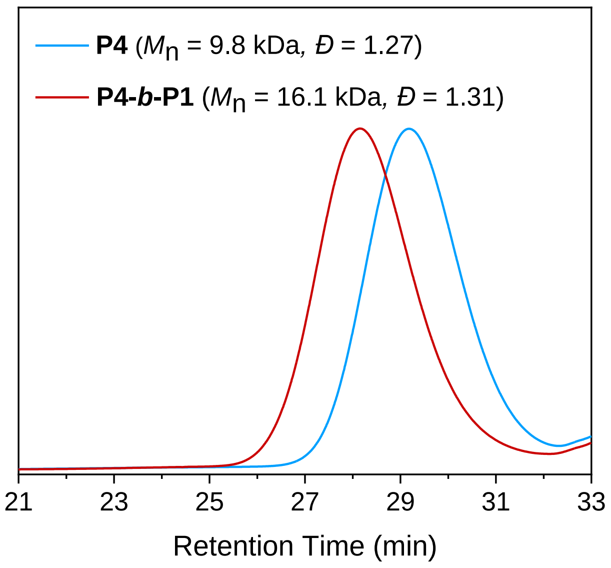

Figure S41. SEC traces for the synthesis of diblock copolymer **P4-*b*-P1**.

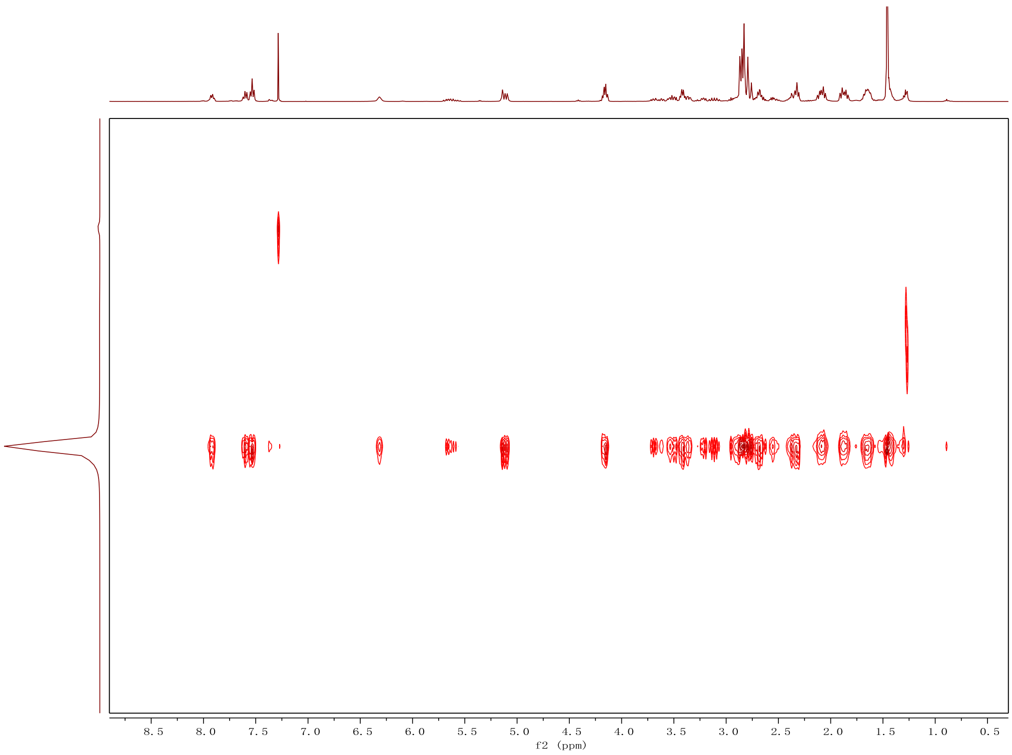

Figure S42. DOSY ($CDCl_3$, 25°C) analysis of diblock copolymer **P4-*b*-P1**.

Scheme S12. Synthesis of diblock copolymer **P1-*b*-P4**.

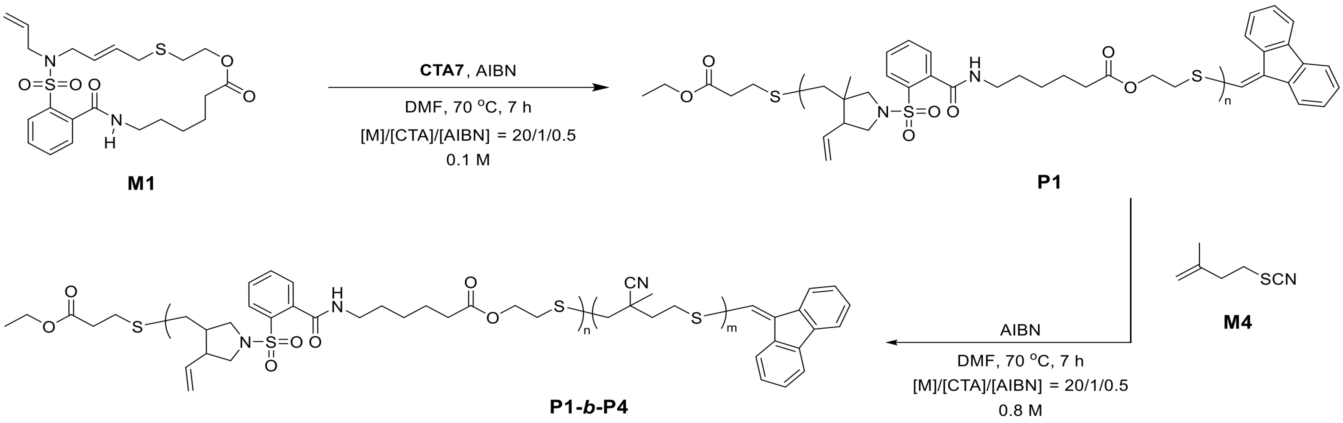

Following the general polymerization procedure, a 10 mL Schlenk vial was charged with monomer **M1** (46.7 mg, 0.2 mmol), followed by the addition of **CTA7** (0.04 M in DMF, 250 μL, 10.0 μmol), AIBN (0.02 M in DMF, 200 μL, 5.0 μmol), and DMF (600 μL). The vial was sealed, and the solution was deoxygenated via three freeze-pump-thaw cycles, backfilled with nitrogen, then heated at 70 °C for 7 h. The vial was cooled and opened to air to stop the polymerization. The monomer conversion was monitored by ^{1}H NMR spectroscopy (48% monomer conversion). The reaction mixture was precipitated twice with hexane, yielding the macroinitiator **P1** ($M_{n,SEC}$ = 6.7 kDa, $Đ$ = 1.33).

A 10 mL Schlenk vial was charged with monomer **M4** (50.9 mg, 0.4 mmol), macroinitiator **P1** (8.0 μmol), AIBN (0.04 M in DMF, 200 L, 4.0 μmol), and DMF (300 L). The vial was sealed, and the solution was deoxygenated via three freeze-pump-thaw cycles, backfilled with nitrogen, then heated at 70 °C for 7 h. The vial was cooled and opened to air to stop the polymerization. The monomer conversion was monitored by ^{1}H NMR spectroscopy (97% monomer conversion). The reaction mixture was precipitated two times with hexane, yielding the polymer **P1-*b*-P4** ($M_{n,SEC}$ = 14.7 kDa, $Đ$ = 1.34).

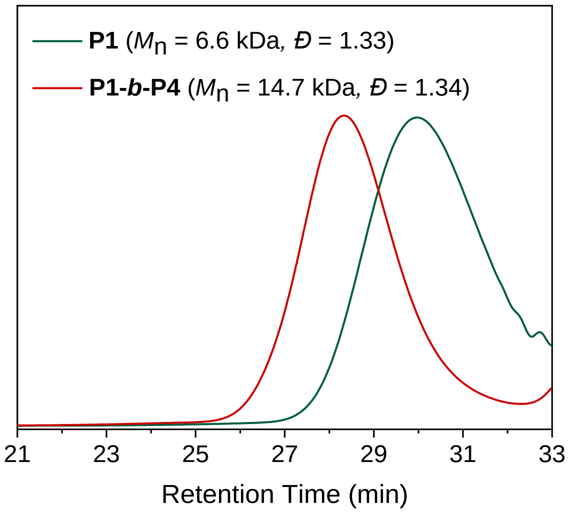

Figure S43. SEC traces for the synthesis of diblock copolymer **P1-*b*-P4**

Scheme S13. Kinetic study for the statistical copolymerization of **M1** and **M4**.

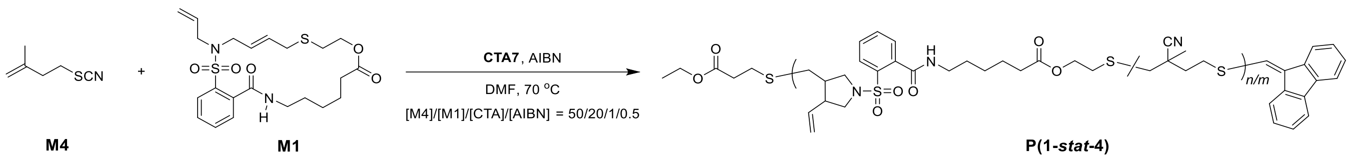

A 10 mL Schlenk vial was charged with monomer **M1** (93.3 mg, 0.2 mmol), **M4** (63.6 mg, 0.5 mmol), **CTA7** (0.04 M in DMF, 250 µL, 10.0 µmol), AIBN (0.02 M in DMF, 250 µL, 5.0 µmol) and DMF (500 µL). The vial was sealed, and the solution was deoxygenated by three freeze-pump-thaw cycles, followed by backfilling with nitrogen. The reaction mixture was then stirred at 70 °C, and aliquots were taken at various time intervals (*t* = 0.5, 1, 2, 3, 4, 5.5, 7 h). Monomer conversion was determined by ^{1}H NMR spectroscopy, while molecular weight and dispersity were analyzed by SEC. The total conversion of the two monomers is defined using the following equation, where $[\mathbf{M1}]_t$ and $[\mathbf{M4}]_t$ are the instantaneous concentrations of **M1** and **M4** at time *t*, respectively, and $[\mathbf{M1}]_0$ and $[\mathbf{M4}]_0$ are the initial concentrations of **M1** and **M4**, respectively.

$$Conversion = \left(1 - \frac{[\mathbf{M1}]_t + [\mathbf{M4}]_t}{[\mathbf{M1}]_0 + [\mathbf{M4}]_0}\right) \times 100\%$$

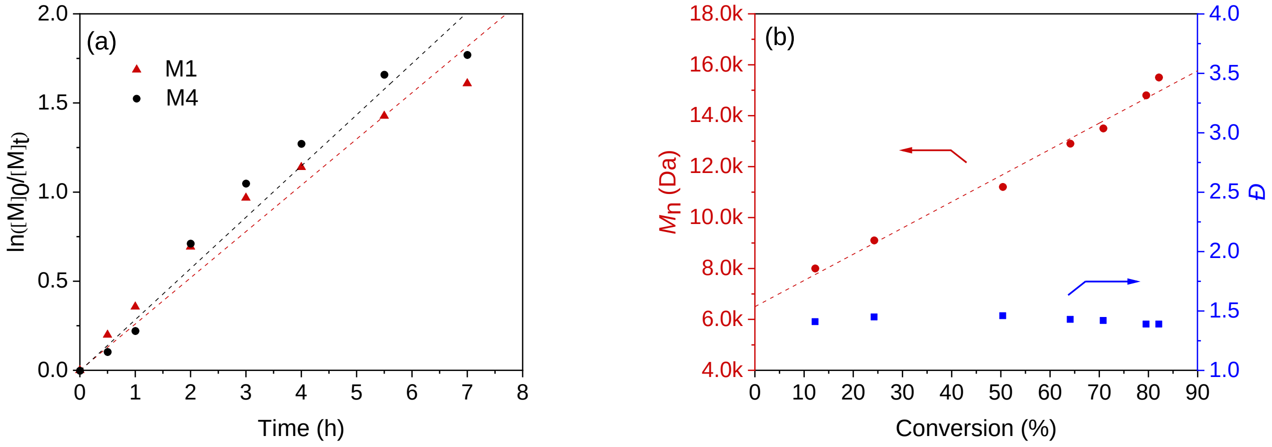

Figure S44. (a) Plot of ln($[M]_0/[M]_t$) versus reaction time for the statistical copolymerization of **M1** and **M4**. (b) Plots of M_n and $Đ$ versus monomer conversion for the statistical copolymerization of **M1** and **M4**.

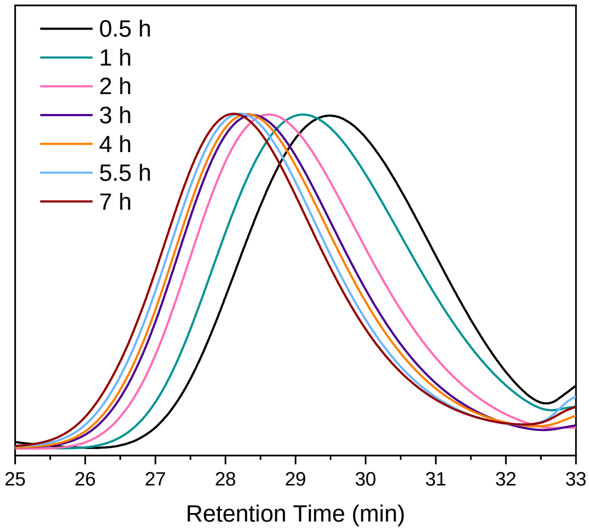

Figure S45. SEC traces for the kinetic study of the statistical copolymerization of **M1** and **M4**.

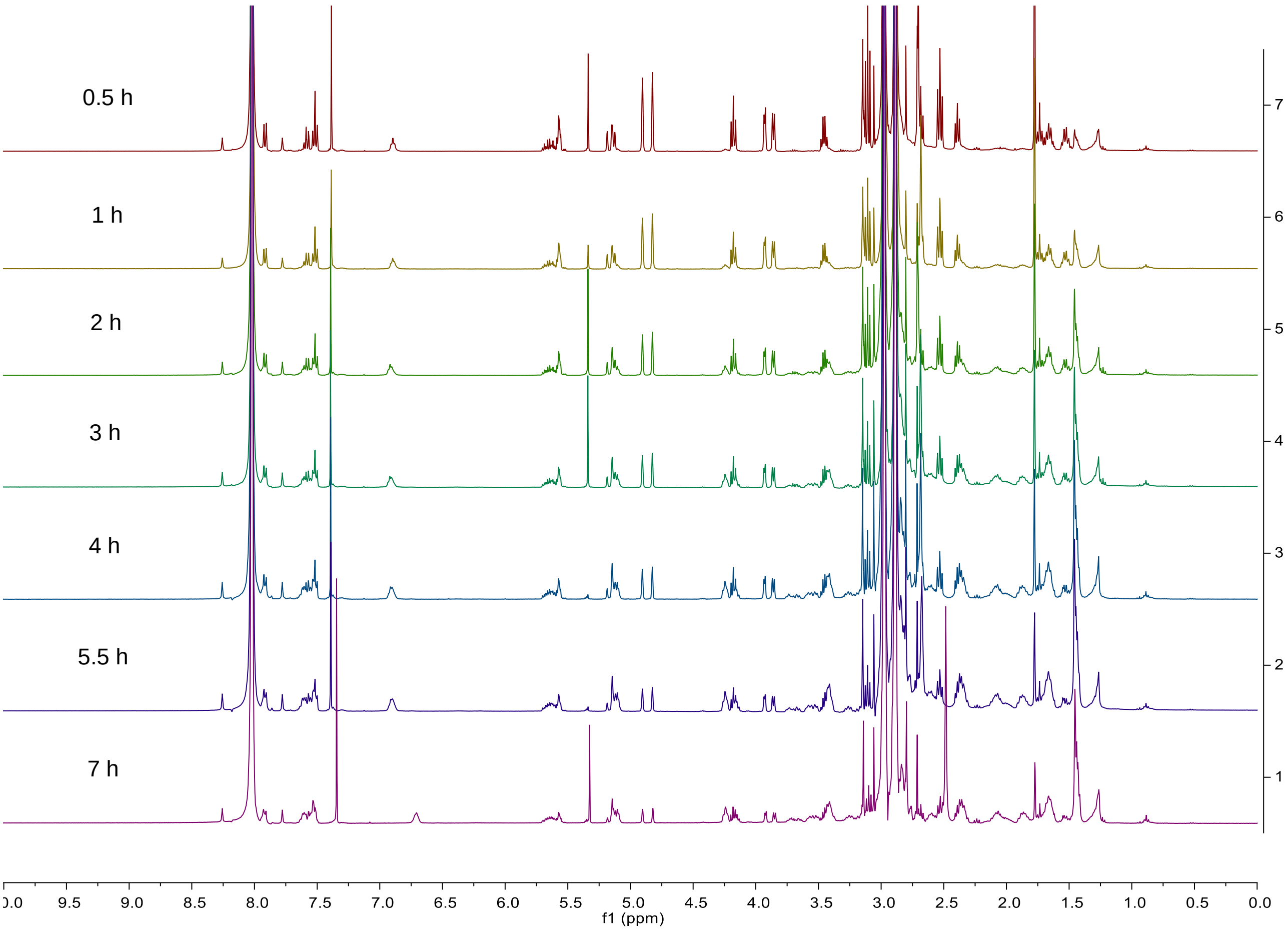

Figure S46. ^{1}H NMR ($CDCl_3$, 25°C) traces for the kinetic study of the statistical copolymerization of **M1** and **M4**.

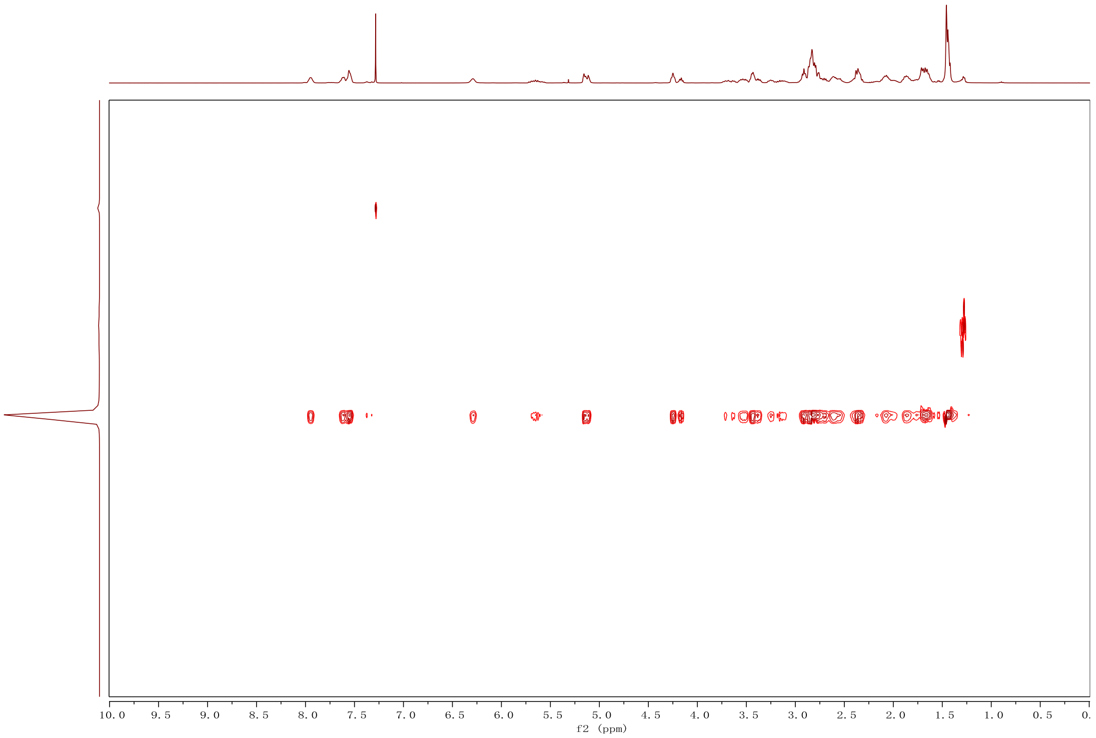

Figure S47. DOSY ($CDCl_3$, 25°C) analysis of the statistical copolymers **P(1-*stat*-4)** ($M_{n,SEC}$ = 15.6 kDa, $Đ$ =1.39).

Scheme S14. Degradation of the statistical copolymer **P(1-*stat*-4)** with sodium methoxide.

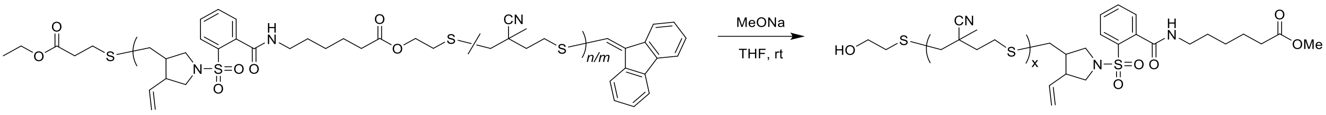

A 3 mL vial equipped with a stir bar was charged with copolymer **P(1-*stat*-4)** ($M_{n,SEC}$ = 15.6 kDa, $Đ$ =1.39, 10 mg) and THF (1.0 mL). Then, sodium methoxide (30 % wt % in methanol, 10 μL) was added and stirred at room temperature. At the given time, a small aliquot was taken, and the reaction was stopped by adding 6 M aqueous hydrochloric acid. The sample was analyzed using SEC.

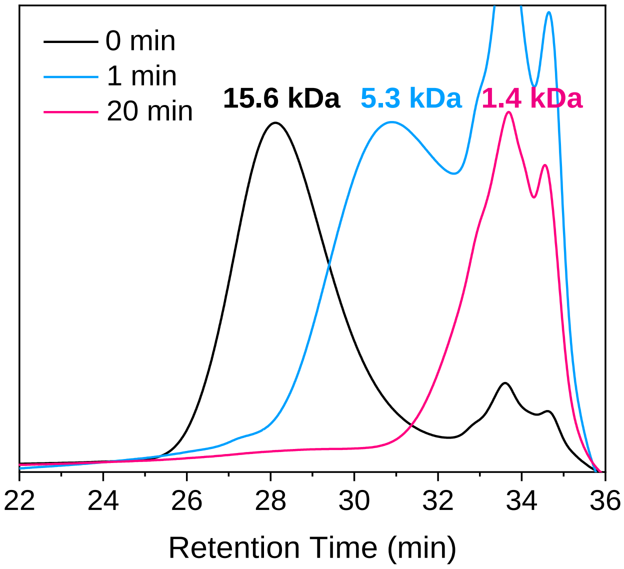

Figure S48. SEC traces for degradation of statistical copolymer **P(1-*stat*-4)**.

References

1 M. J. Frisch, G. W. Trucks, H. B. Schlegel, G. E. Scuseria, M. A. Robb, J. R. Cheeseman, G. Scalmani, V. Barone, B. Mennucci, G. A. Petersson, H. Nakatsuji, M. Caricato, X. Li, H. P. Hratchian, A. F. Izmaylov, J. Bloino, G. Zheng, J. L. Sonnenberg, M. Hada, M. Ehara, K. Toyota, R. Fukuda, J. Hasegawa, M. Ishida, T. Nakajima, Y. Honda, O. Kitao, H. Nakai, T. Vreven, J. A. Montgomery, Jr., J. E. Peralta, F. Ogliaro, M. Bearpark, J. J. Heyd, E. Brothers, K. N. Kudin, V. N. Staroverov, R. Kobayashi, J. Normand, K. Raghavachari, A. Rendell, J. C. Burant, S. S. Iyengar, J. Tomasi, M. Cossi, N. Rega, J. M. Millam, M. Klene, J. E. Knox, J. B. Cross, V. Bakken, C. Adamo, J. Jaramillo, R. Gomperts, R. E. Stratmann, O. Yazyev, A. J. Austin, R. Cammi, C. Pomelli, J. W. Ochterski, R. L. Martin, K. Morokuma, V. G. Zakrzewski, G. A. Voth, P. Salvador, J. J. Dannenberg, S. Dapprich, A. D. Daniels, O. Farkas, J. B. Foresman, J. V. Ortiz, J. Cioslowski and D. J. Fox, Gaussian 16, Gaussian, Inc., Wallingford CT, 2016.

2 a) Y. Zhao and D. G. Truhlar, *Theor. Chem. Acc.*, 2008, **120**, 215–241; b) F. Weigend and R. Ahlrichs, *Phys. Chem. Chem. Phys.*, 2005, **7**, 3297–3305.

3 a) S. Grimme, J. Antony, S. Ehrlich and H. Krieg, *J. Chem. Phys.*, 2010, **132**, 154104; b) L. Goerigk and S. Grimme, *J. Chem. Theory Comput.*, 2011, **7**, 291-309; c) S. Grimme, S. Ehrlich and L. Goerigk, *J. Comput. Chem.*, 2011, **32**, 1456–1465.

4 a) F. Neese, *Wiley Interdiscip. Rev.: Comput. Mol. Sci.*, 2012, **2**, 73–78; b) F. Neese, *Wiley Interdiscip. Rev.: Comput. Mol. Sci.*, 2022, **12**, e1606.

5 T. Lu and Q. Chen, *Comput. Theor. Chem.*, 2021, **1200**, 113249.

6 M. K. Kesharwani, B. Brauer and J. M. Martin, *J. Phys. Chem. A*, 2015, **119**, 1701–1714.

7 S. Grimme, *Chem. Eur. J.*, 2012, **18**, 9955–9964.

8 S. Zhang, C. Cao, S. Jiang and H. Huang, *Macromolecules*, 2022, **55**, 9411–9419.

9 S. Jiang and H. Huang, *Angew. Chem. Int. Ed.*, 2023, **62**, e202217895.

10 T. Lu, TST calculator, http://sobereva.com/310 (accessed July, 2023).

11 G. Moad, E. Rizzardo and S. H. Thang, *Polymer*, 2008, **49**, 1079–1131.

NMR Spectra

^{1}H NMR ($CDCl_3$, 25 °C) and ^{13}C NMR ($CDCl_3$, 25 °C) spectra for **CTA1** (Scheme S1)

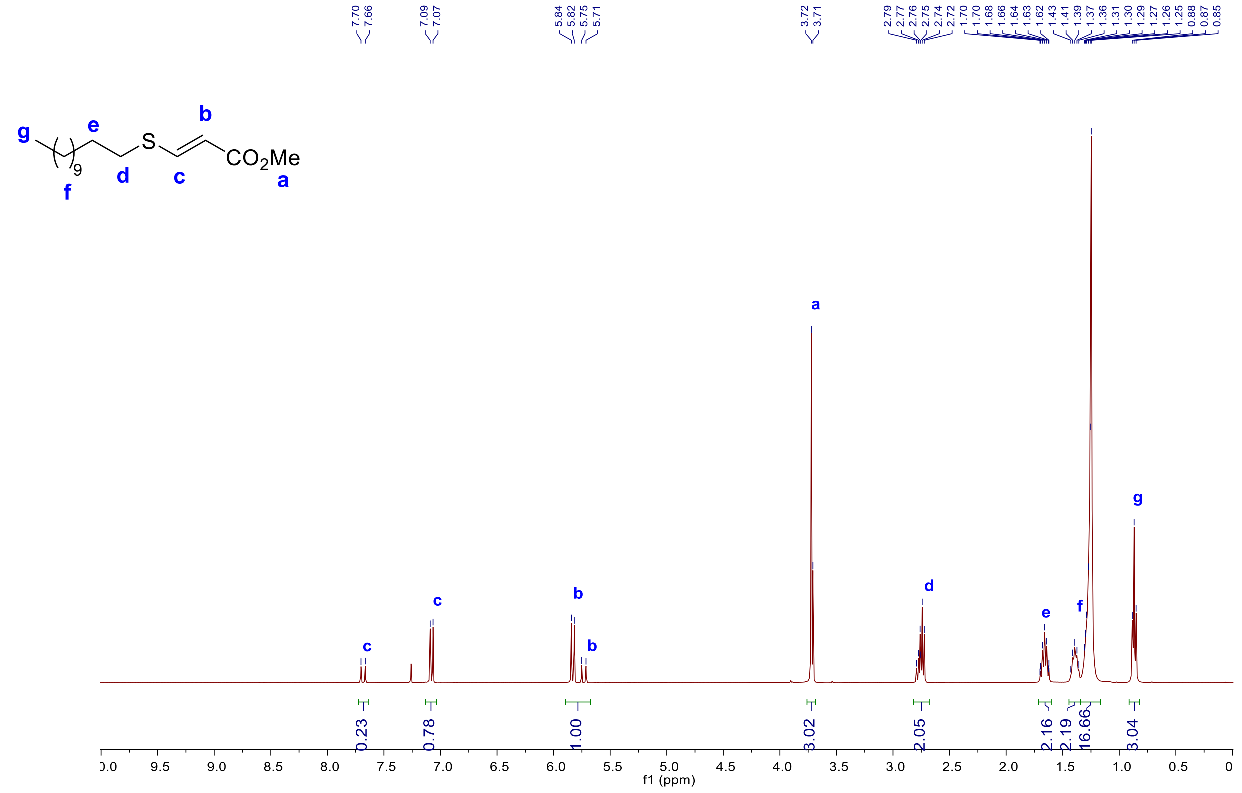

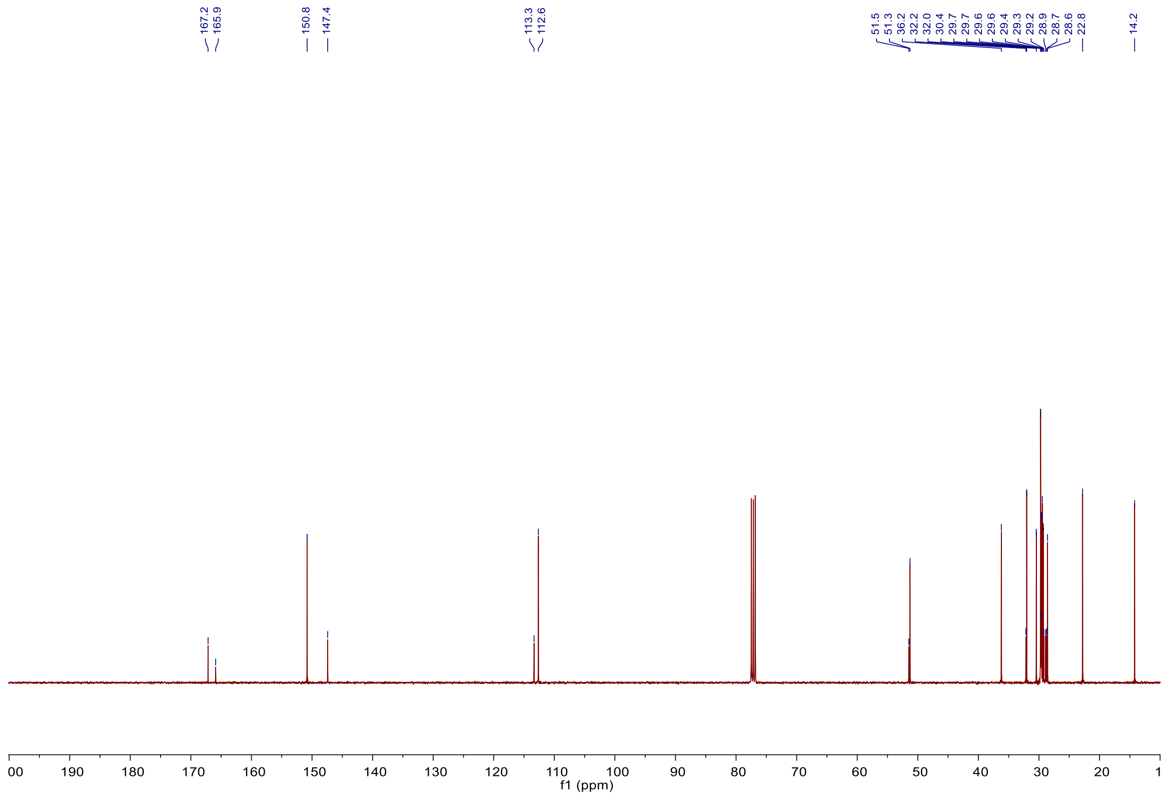

^{1}H NMR ($CDCl_3$, 25 °C) and ^{13}C NMR ($CDCl_3$, 25 °C) spectra for **CTA2** (Scheme S1)

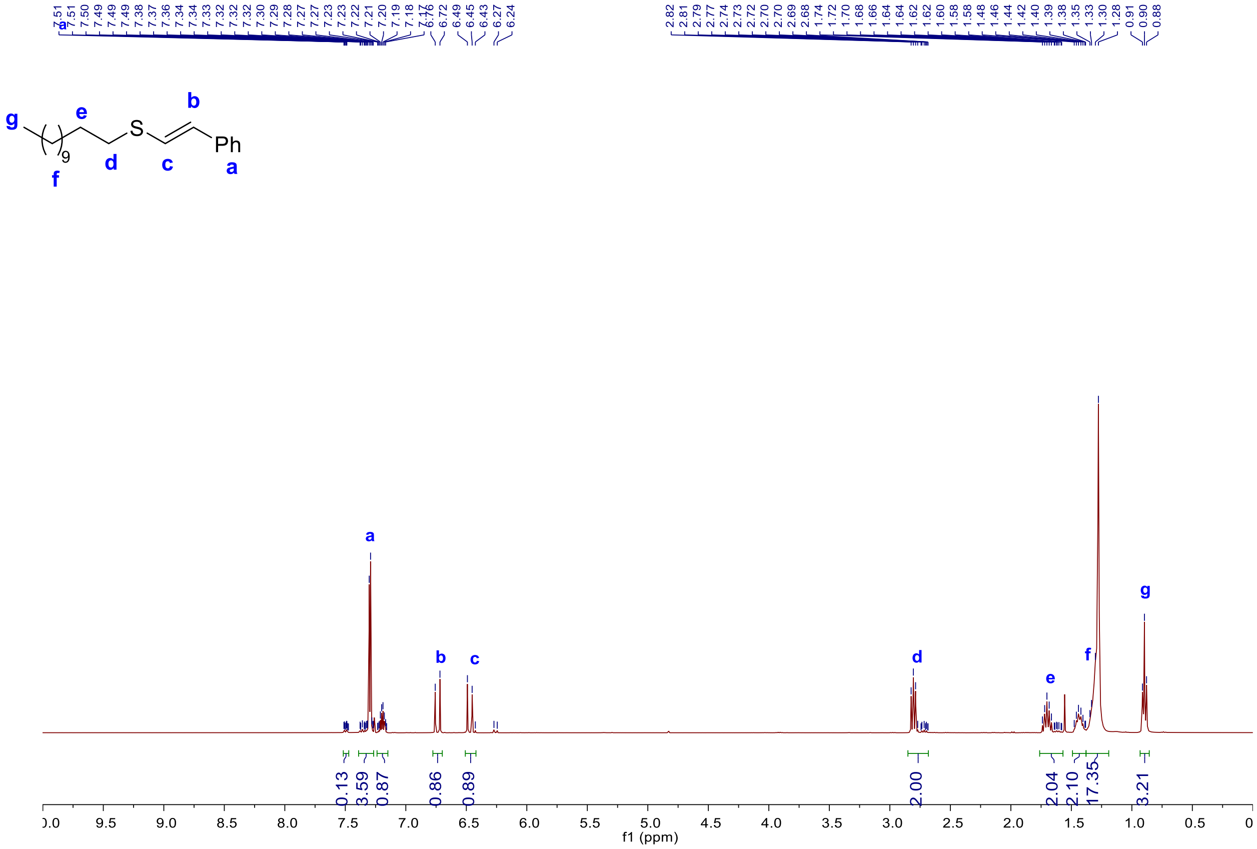

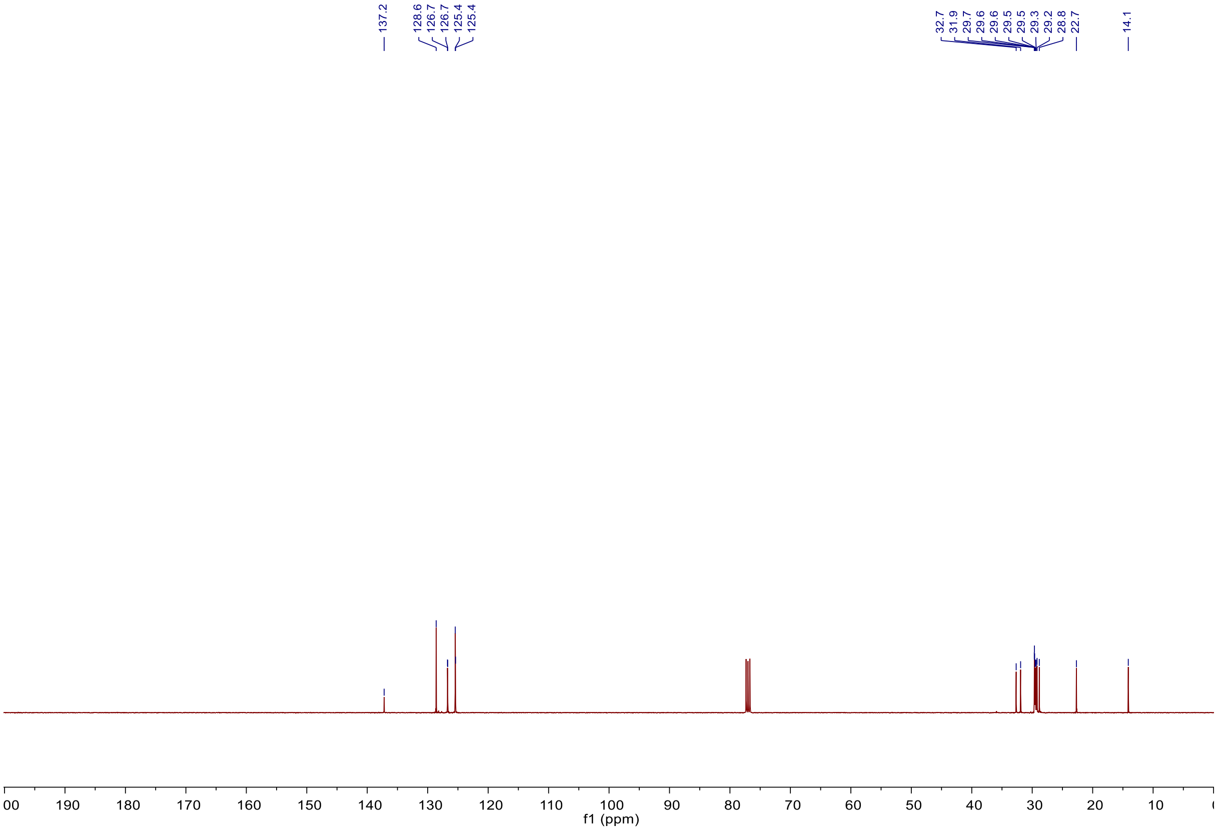

^{1}H NMR ($CDCl_3$, 25 °C) and ^{13}C NMR ($CDCl_3$, 25 °C) spectra for **CTA3** (Scheme S1)

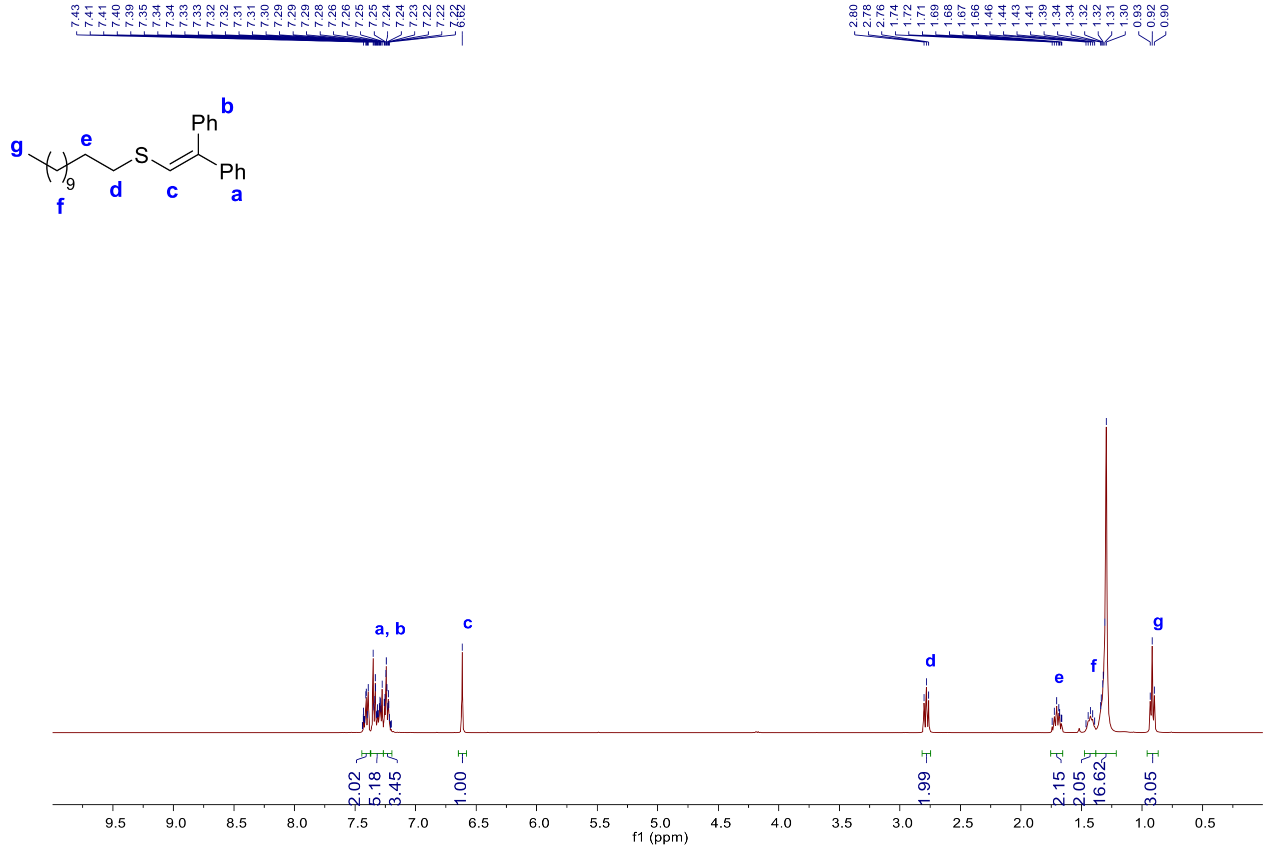

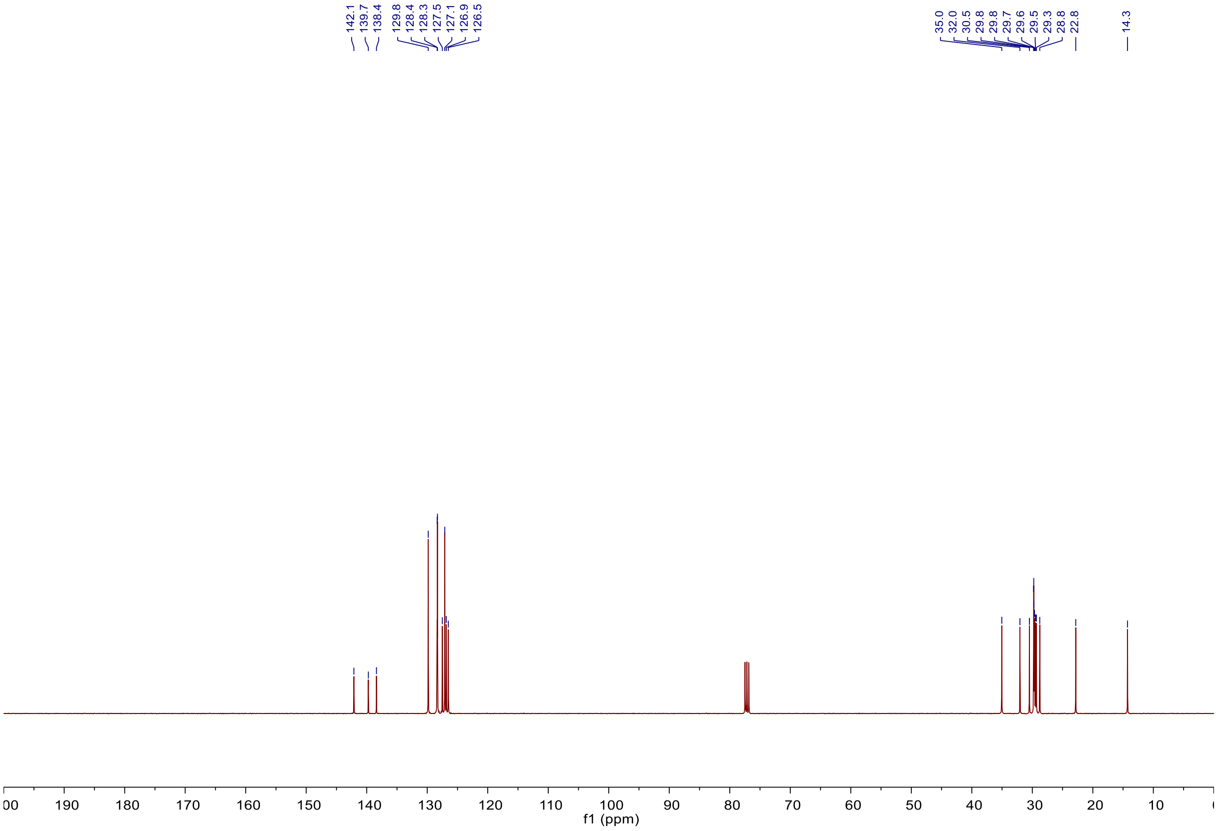

^{1}H NMR ($CDCl_3$, 25 °C) and ^{13}C NMR ($CDCl_3$, 25 °C) spectra for **S1** (Scheme S1)

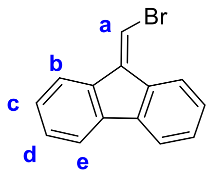

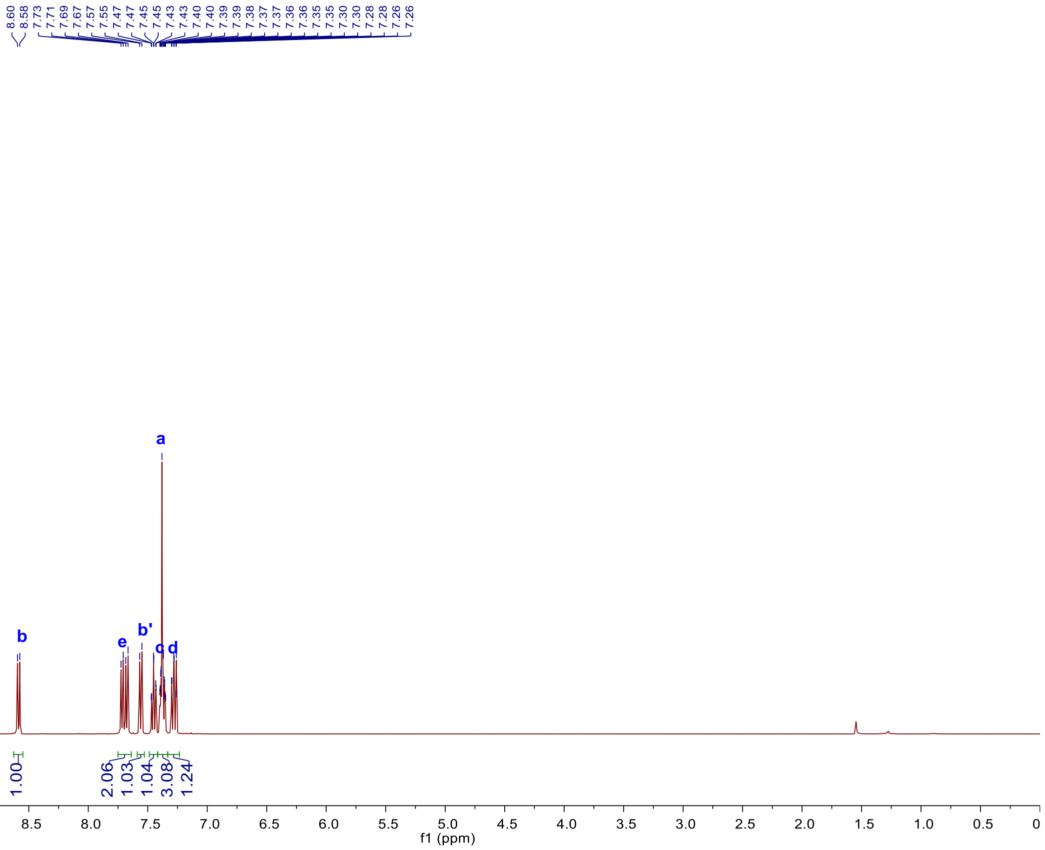

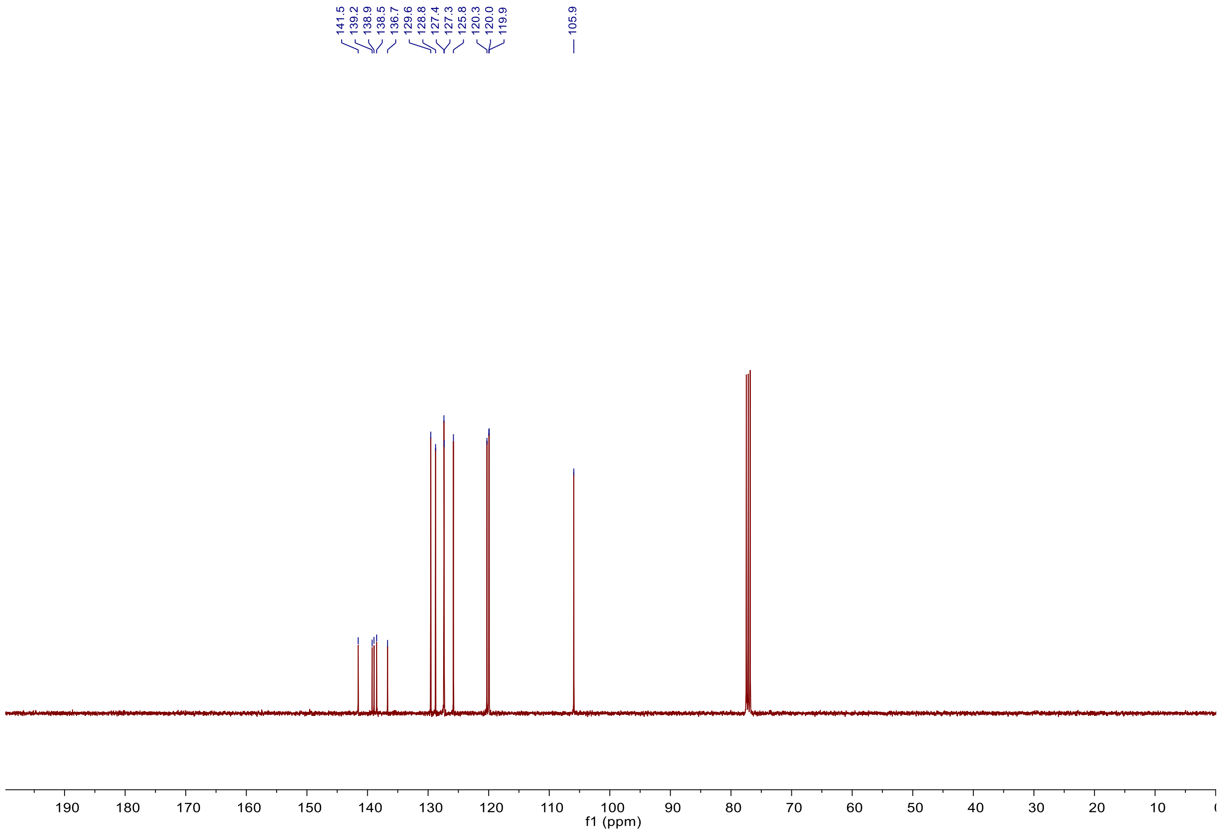

^{1}H NMR ($CDCl_3$, 25 °C) and ^{13}C NMR ($CDCl_3$, 25 °C) spectra for **S2** (Scheme S1)

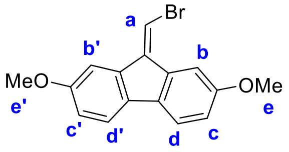

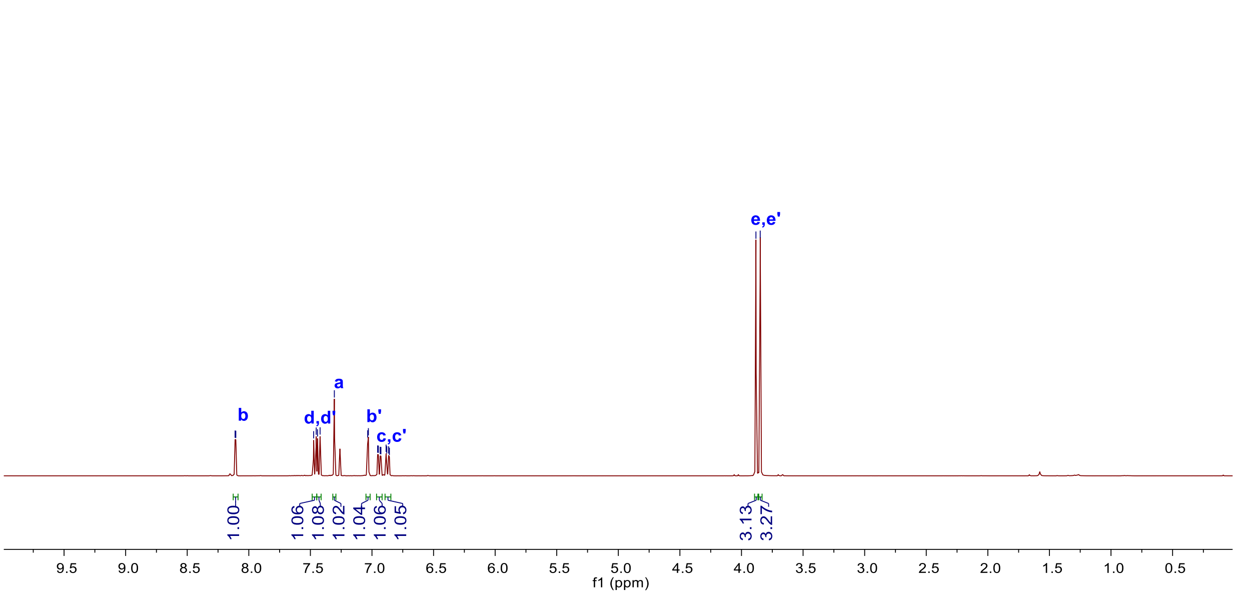

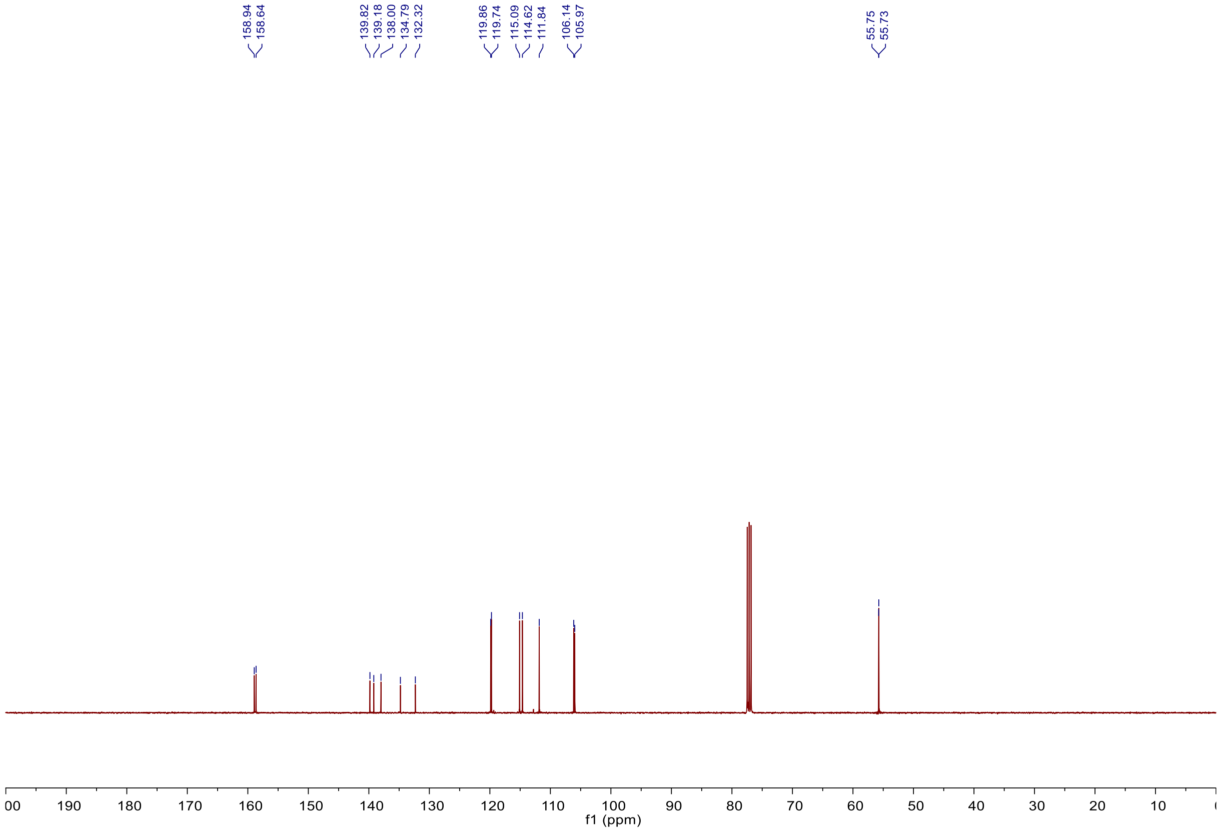

^{1}H NMR (CDCl$_3$, 25 °C) and ^{13}C NMR (CDCl$_3$, 25 °C) spectra for **CTA4** (Scheme S1)

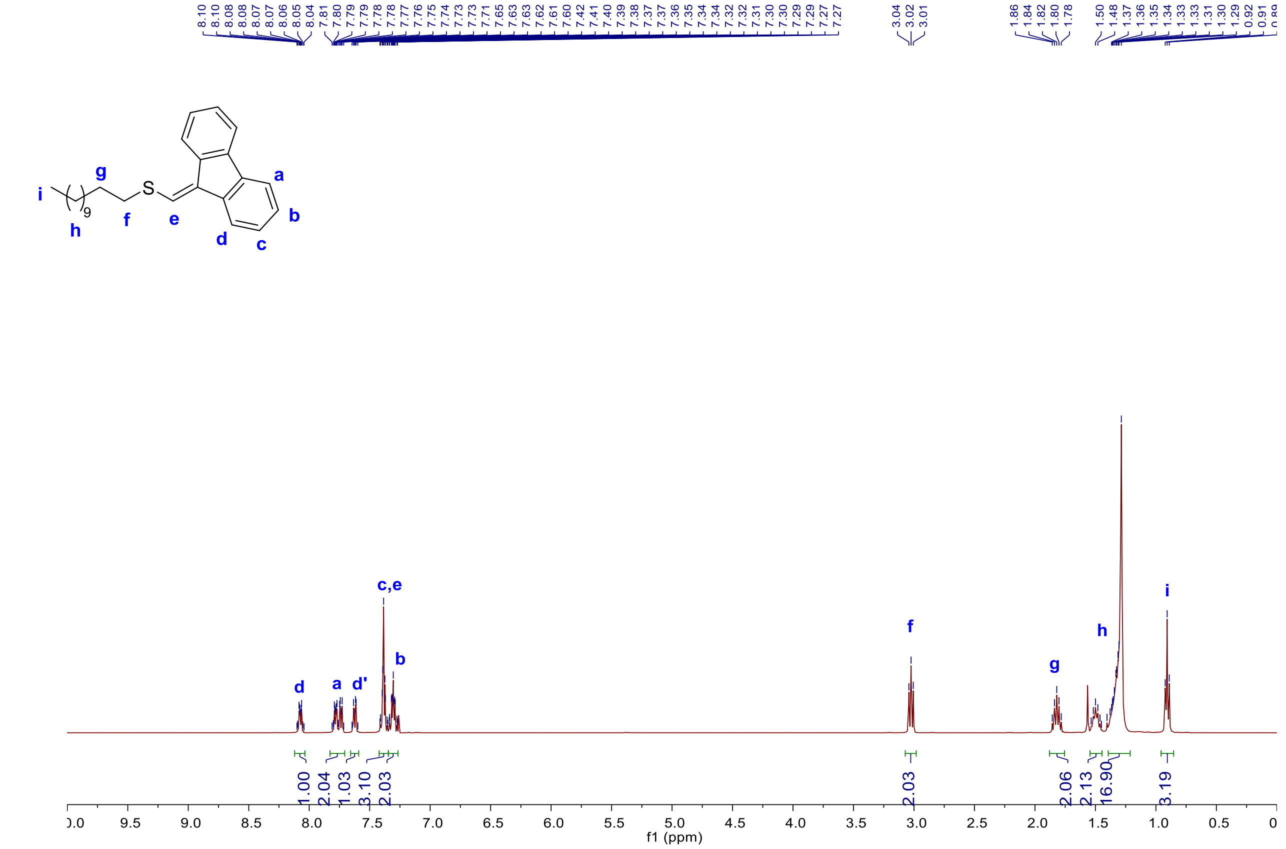

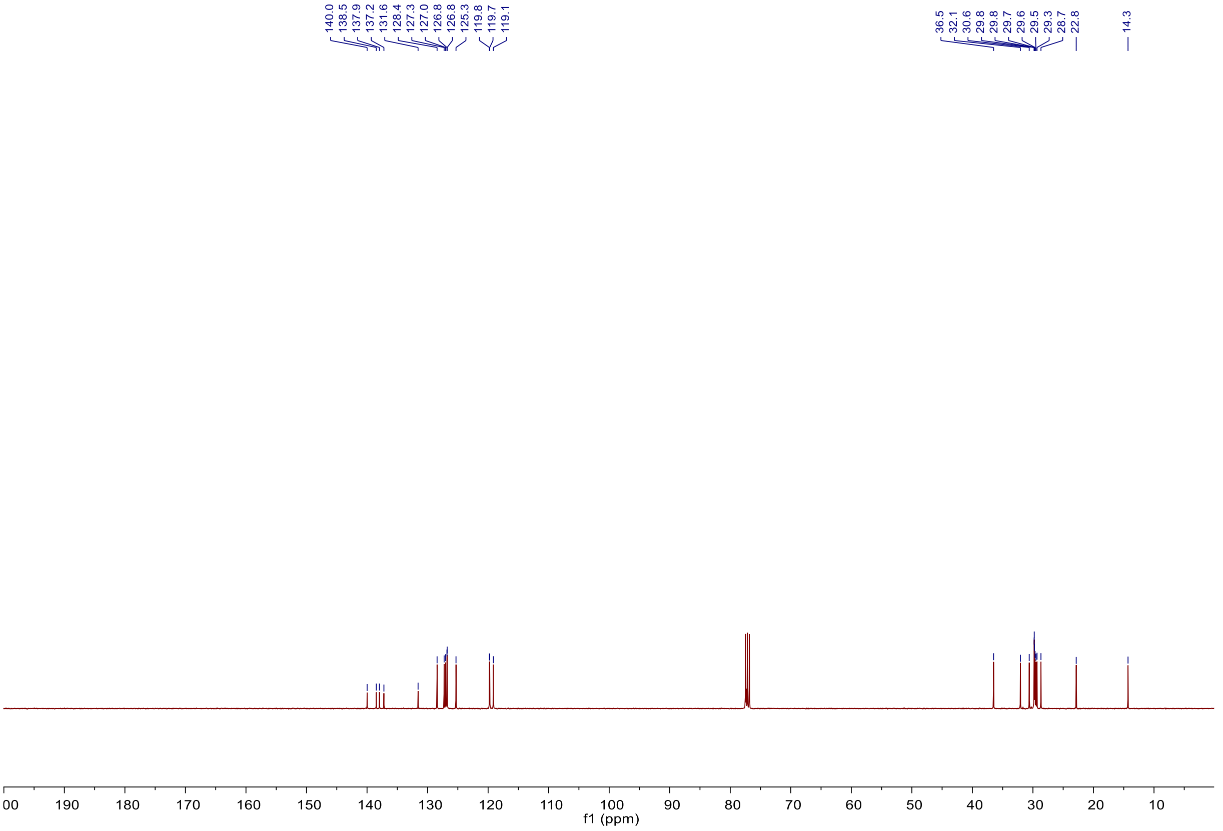

^{1}H NMR ($CDCl_3$, 25 °C) and ^{13}C NMR ($CDCl_3$, 25 °C) spectra for **CTA5** (Scheme S1)

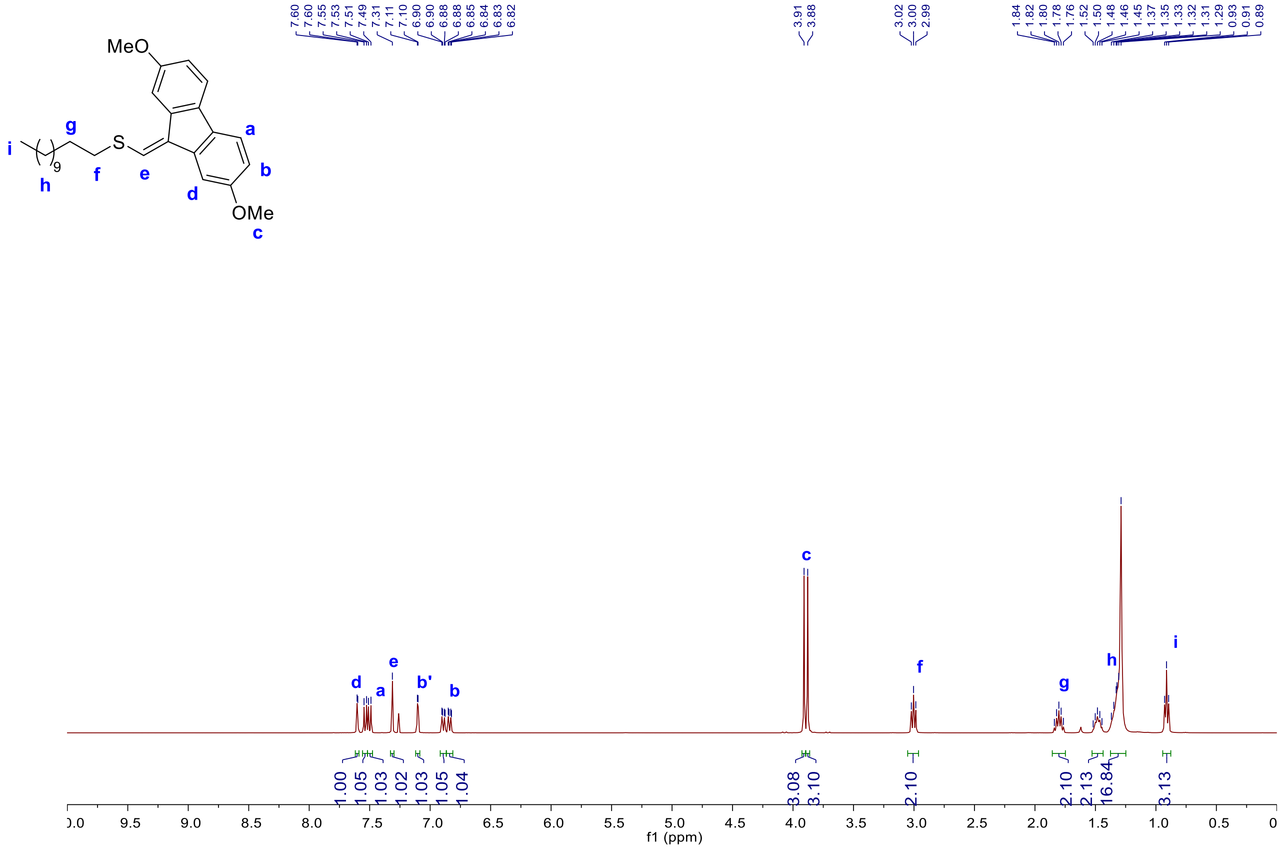

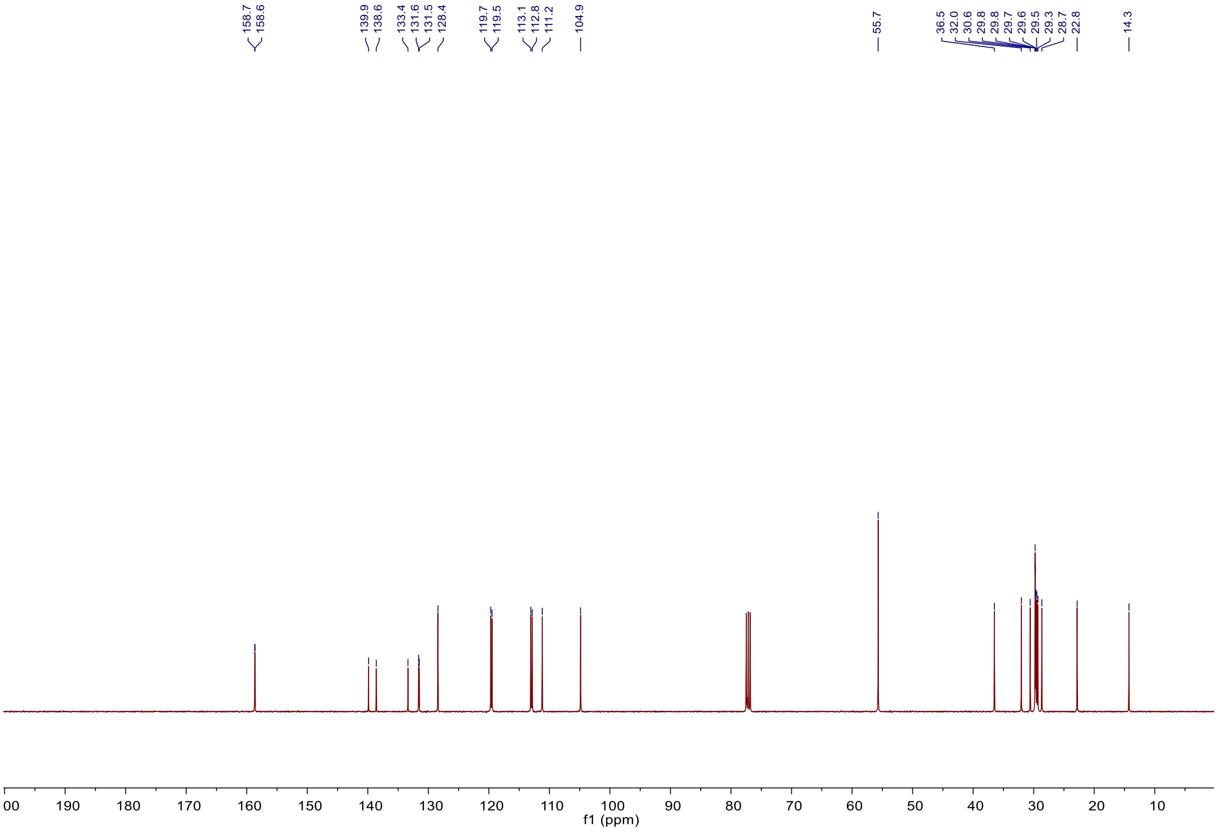

^{1}H NMR ($CDCl_3$, 25 °C) and ^{13}C NMR ($CDCl_3$, 25 °C) spectra for **CTA6** (Scheme S1)

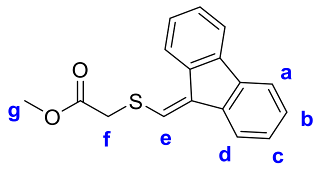

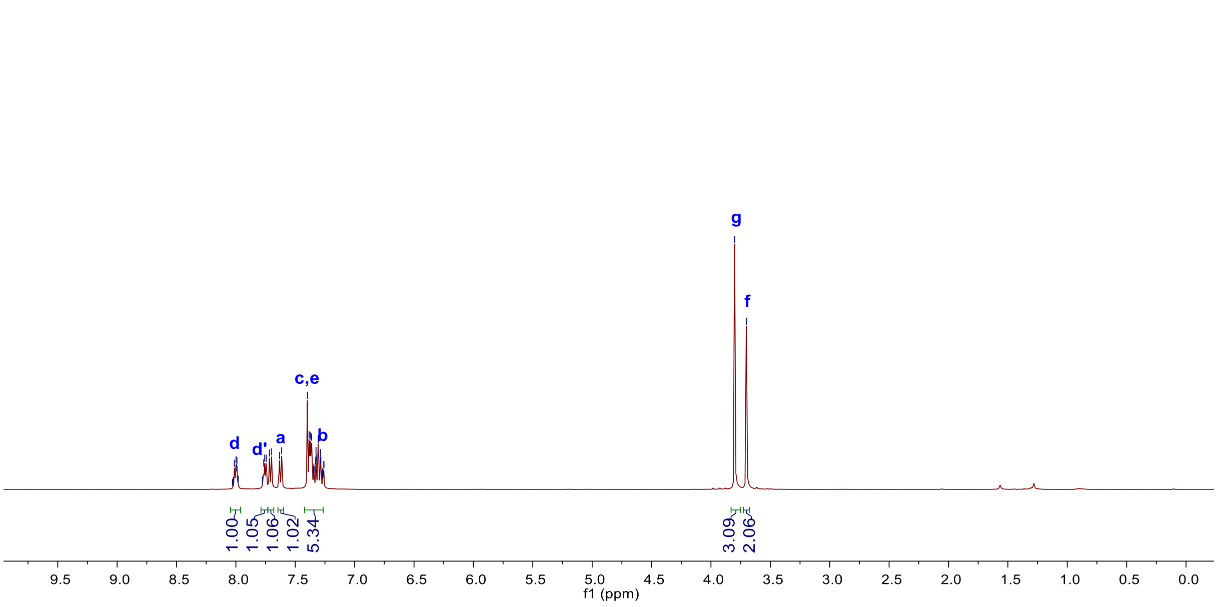

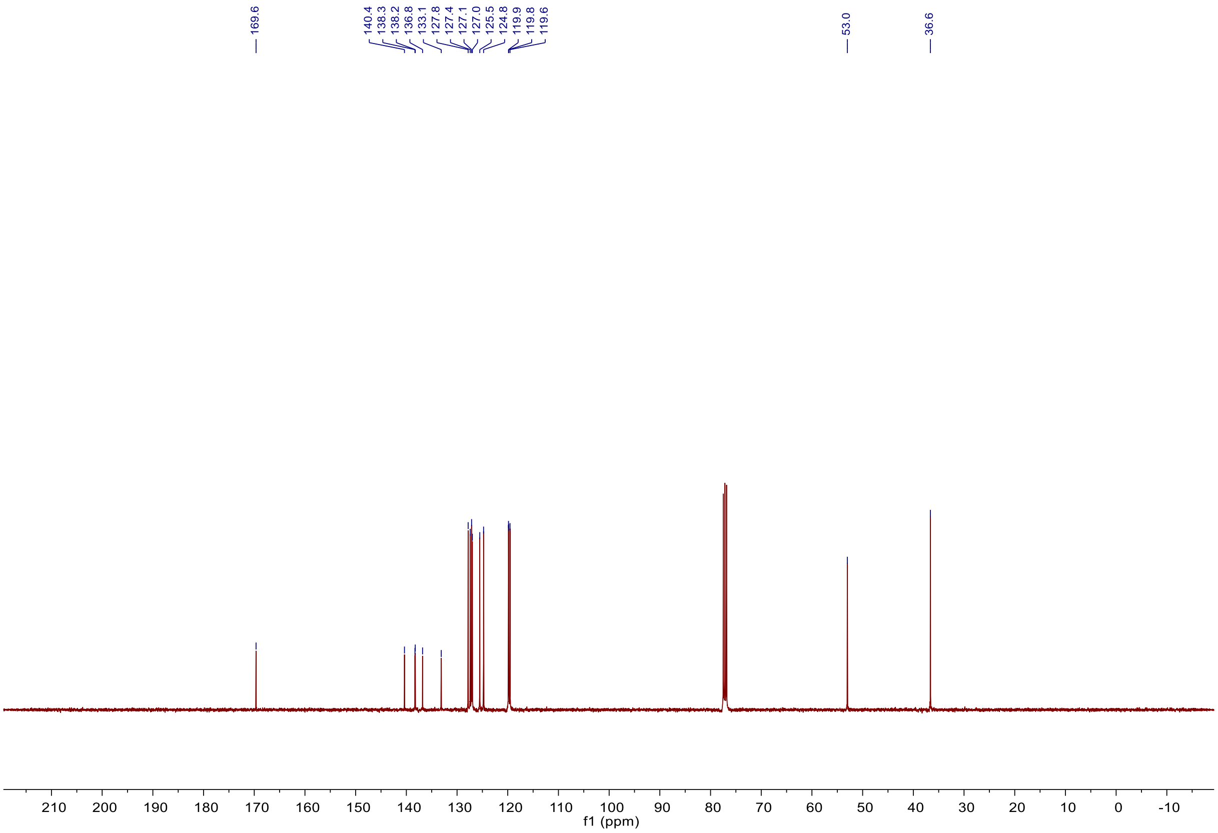

^{1}H NMR ($CDCl_3$, 25 °C) and ^{13}C NMR ($CDCl_3$, 25 °C) spectra for **CTA7** (Scheme S1)

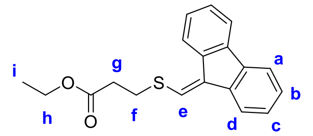

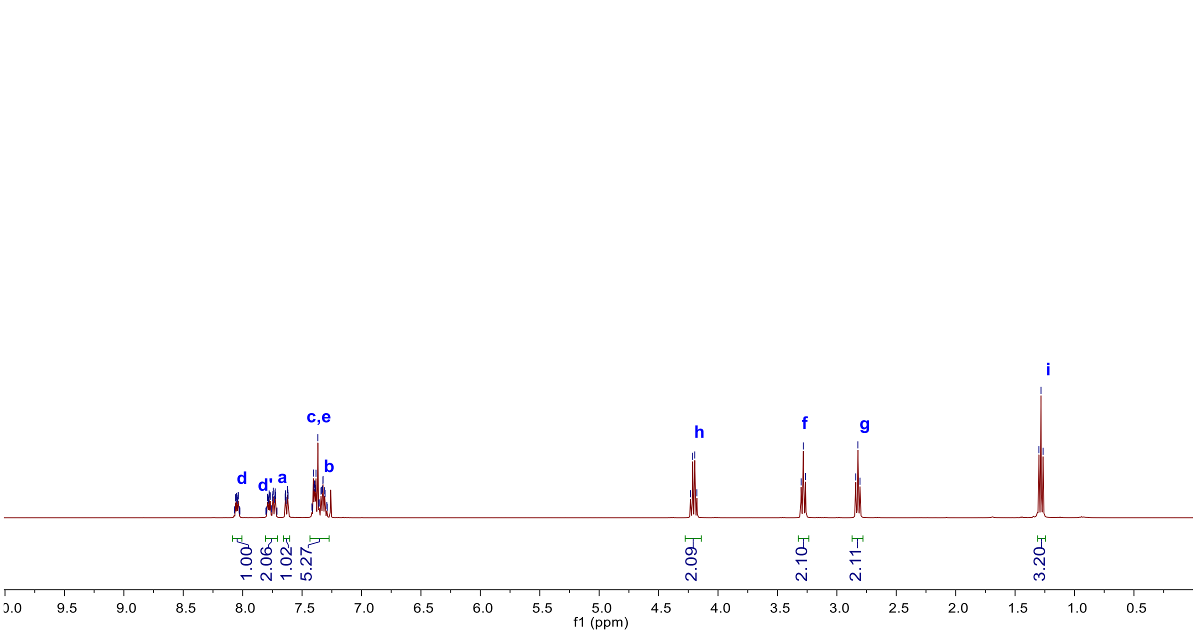

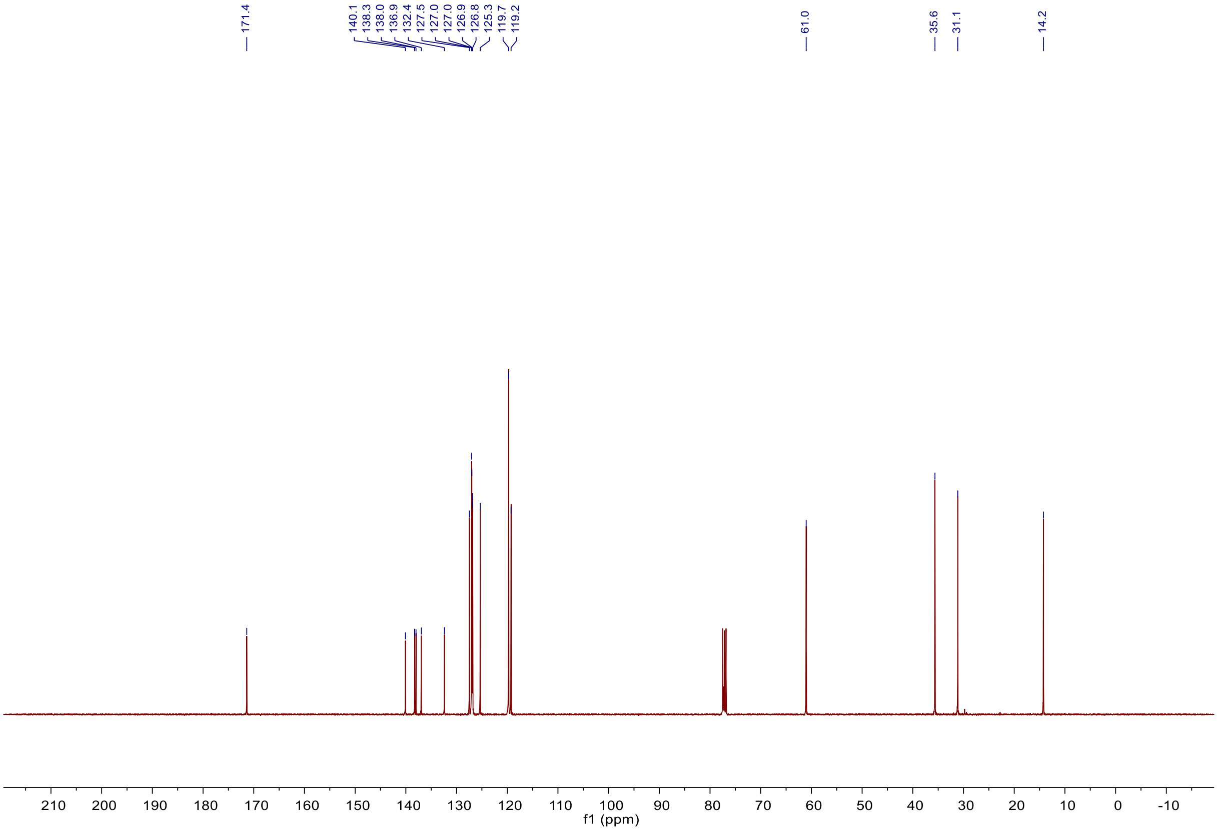

^{1}H NMR ($CDCl_3$, 25 °C) and ^{13}C NMR ($CDCl_3$, 25 °C) spectra for **CTA8** (Scheme S1)

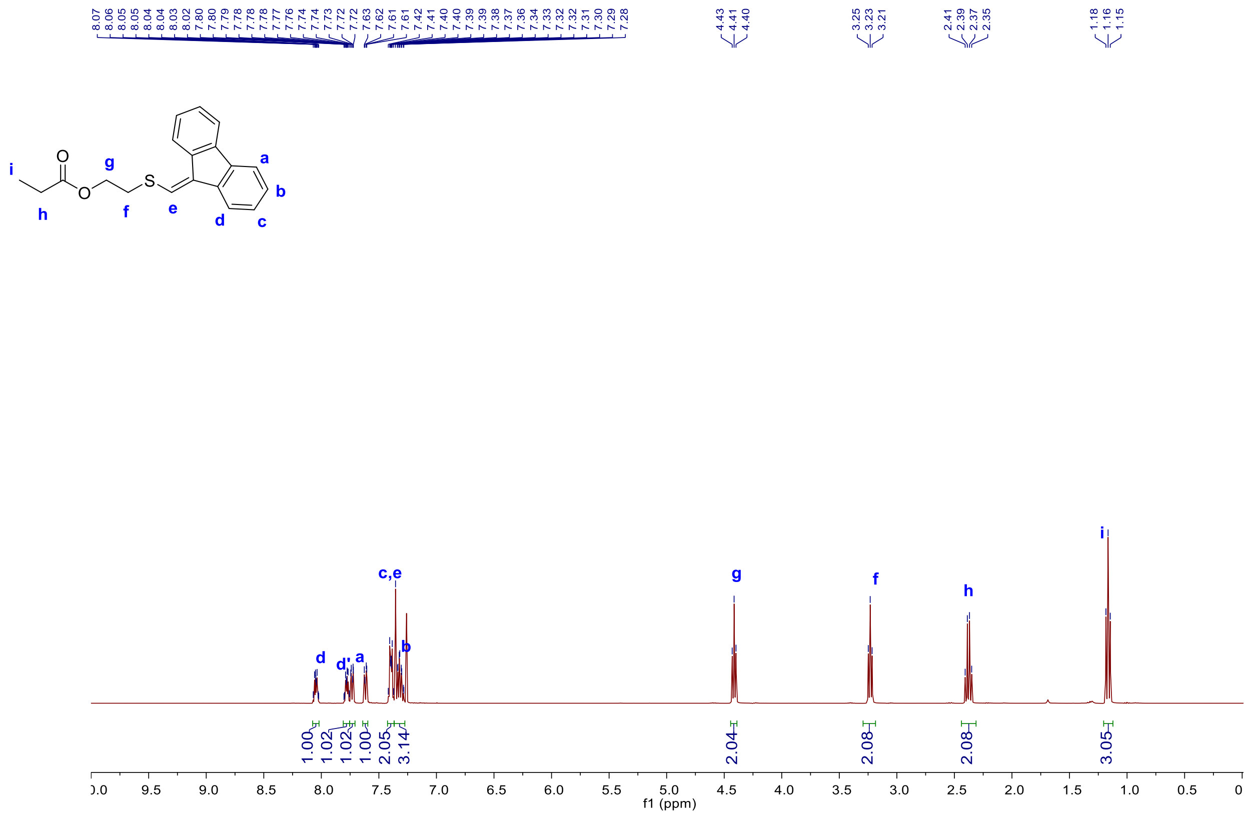

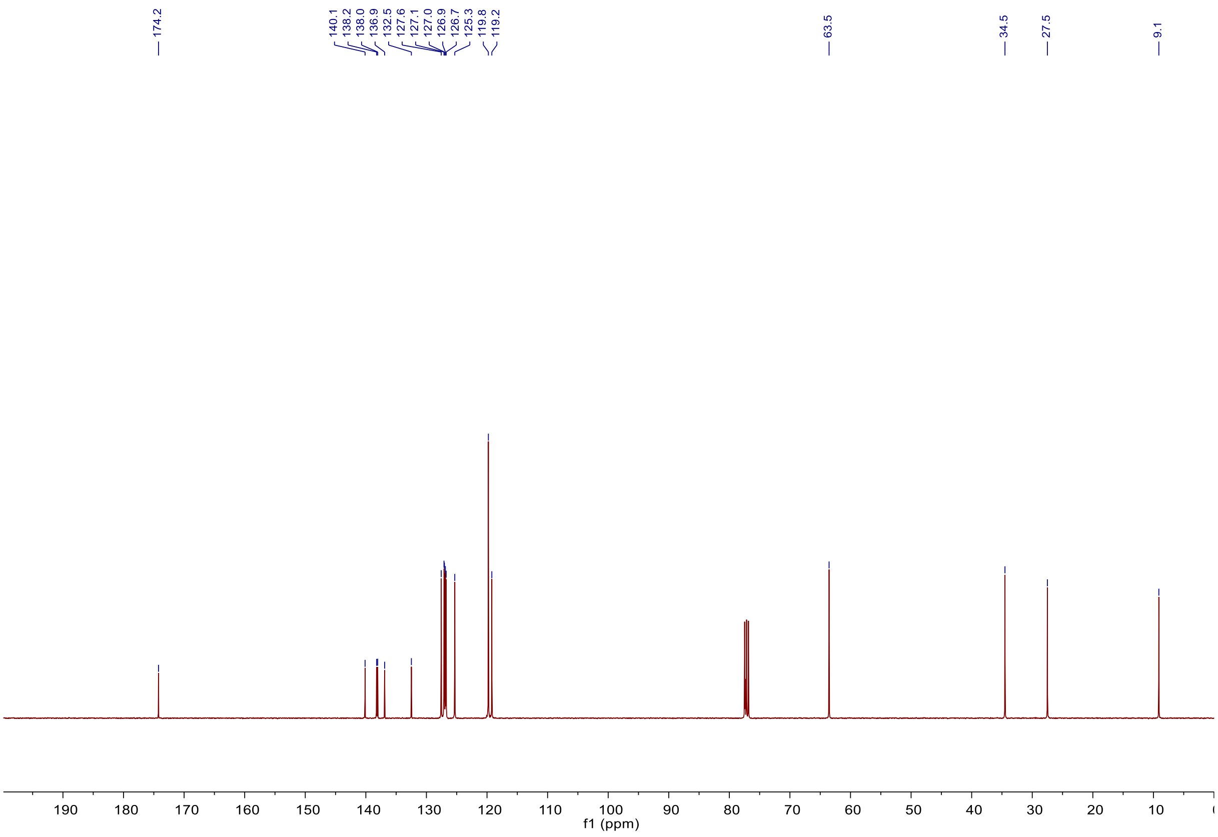

^{1}H NMR ($CDCl_3$, 25 °C) and ^{13}C NMR ($CDCl_3$, 25 °C) spectra for **CTA9** (Scheme S1)

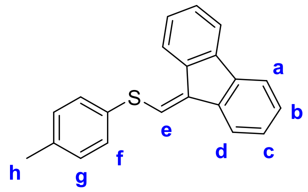

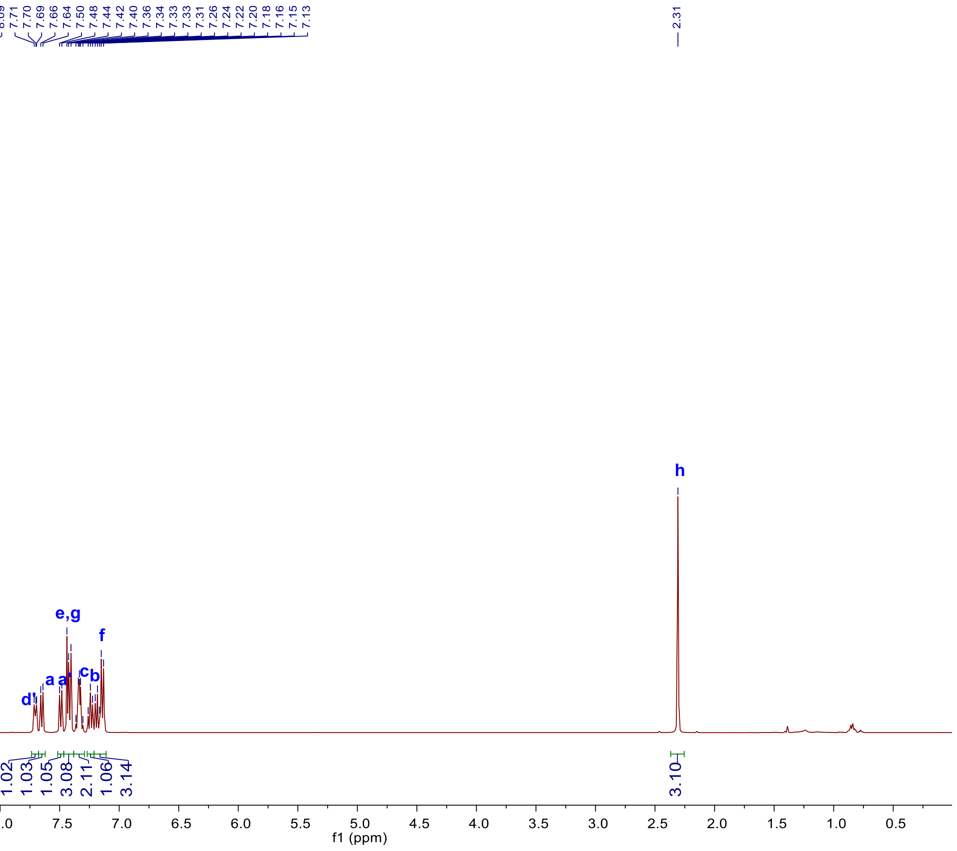

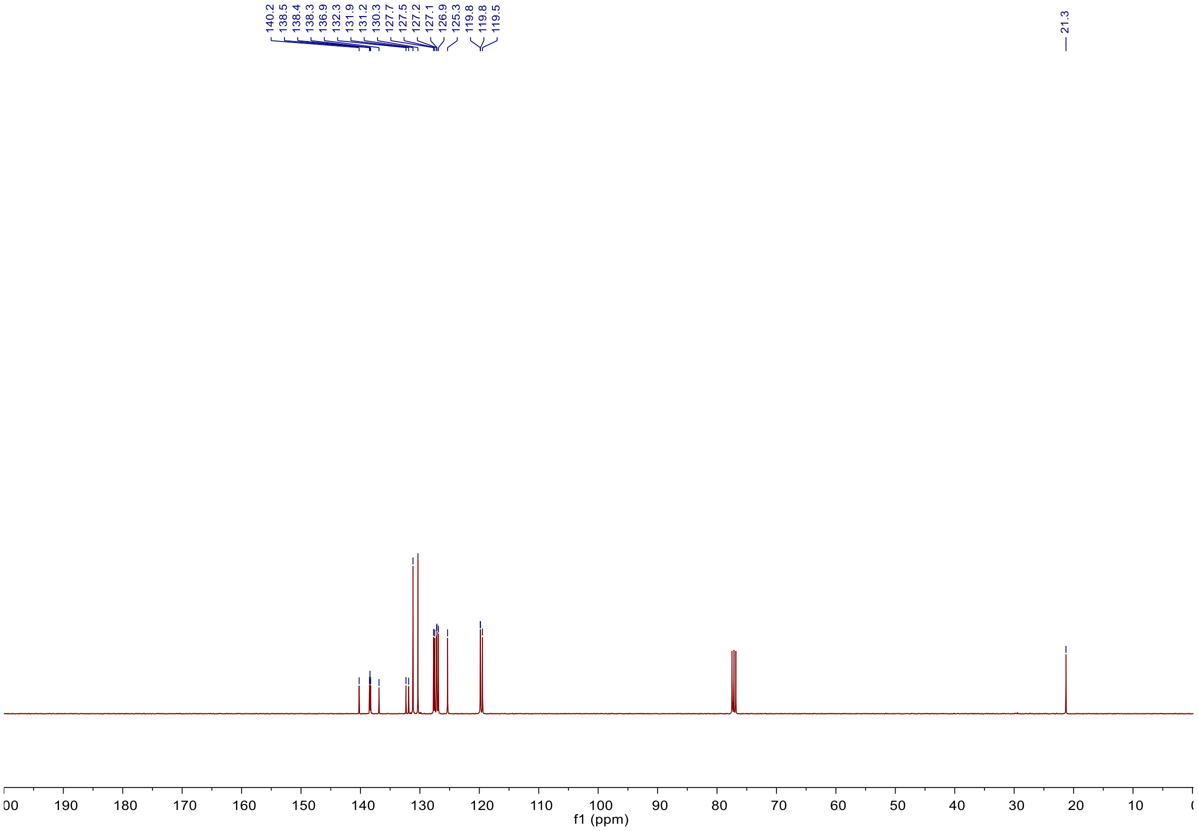

^{1}H NMR ($CDCl_3$, 25 °C) and ^{13}C NMR ($CDCl_3$, 25 °C) spectra for **M1** (Scheme S2)

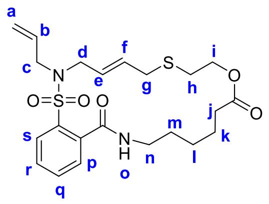

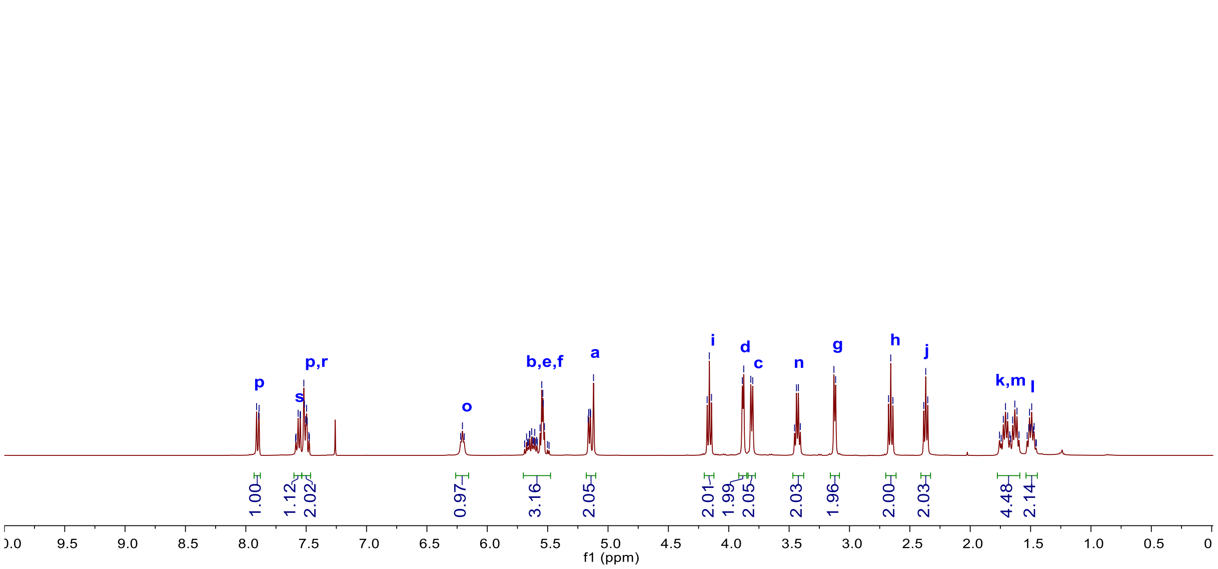

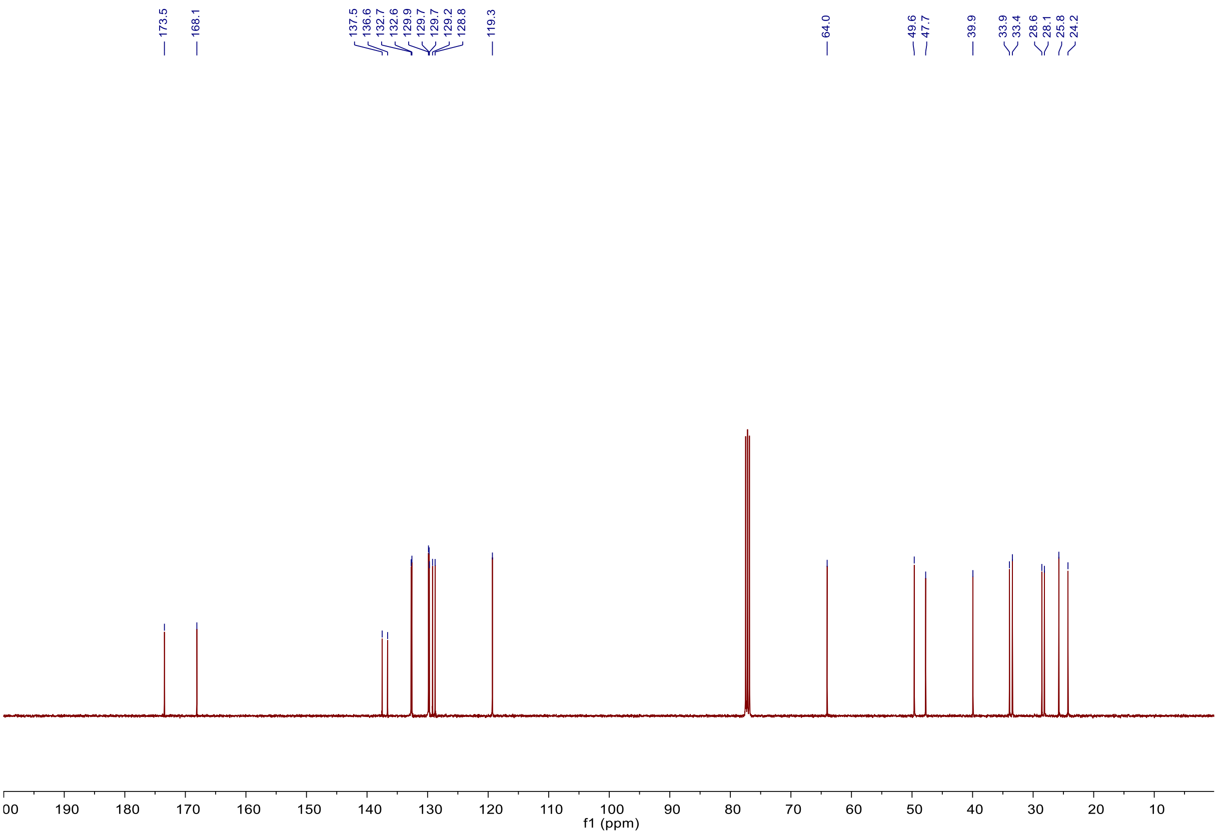

^{1}H NMR ($CDCl_3$, 25 °C) and ^{13}C NMR ($CDCl_3$, 25 °C) spectra for **M2** (Scheme S2)

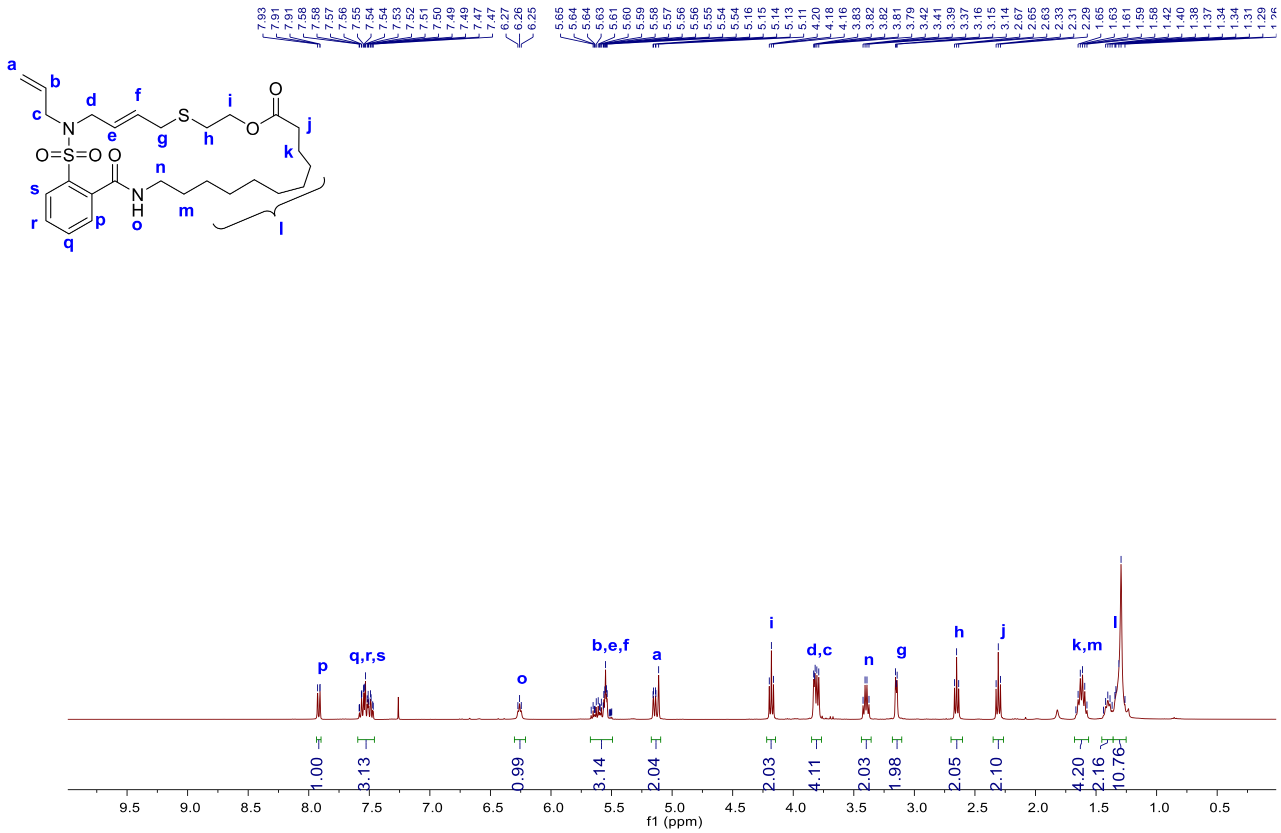

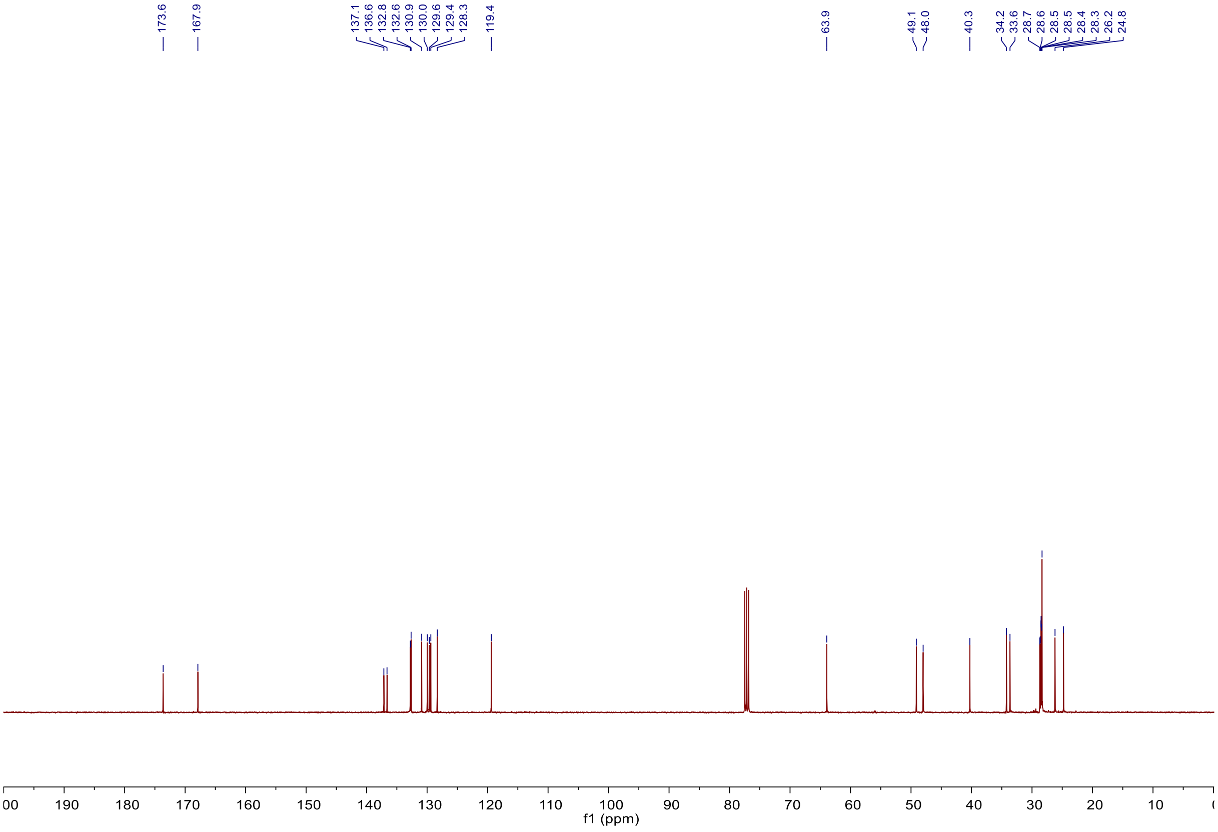

^{1}H NMR ($CDCl_3$, 25 °C) and ^{13}C NMR ($CDCl_3$, 25 °C) spectra for **S3** (Scheme S2)

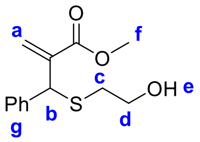

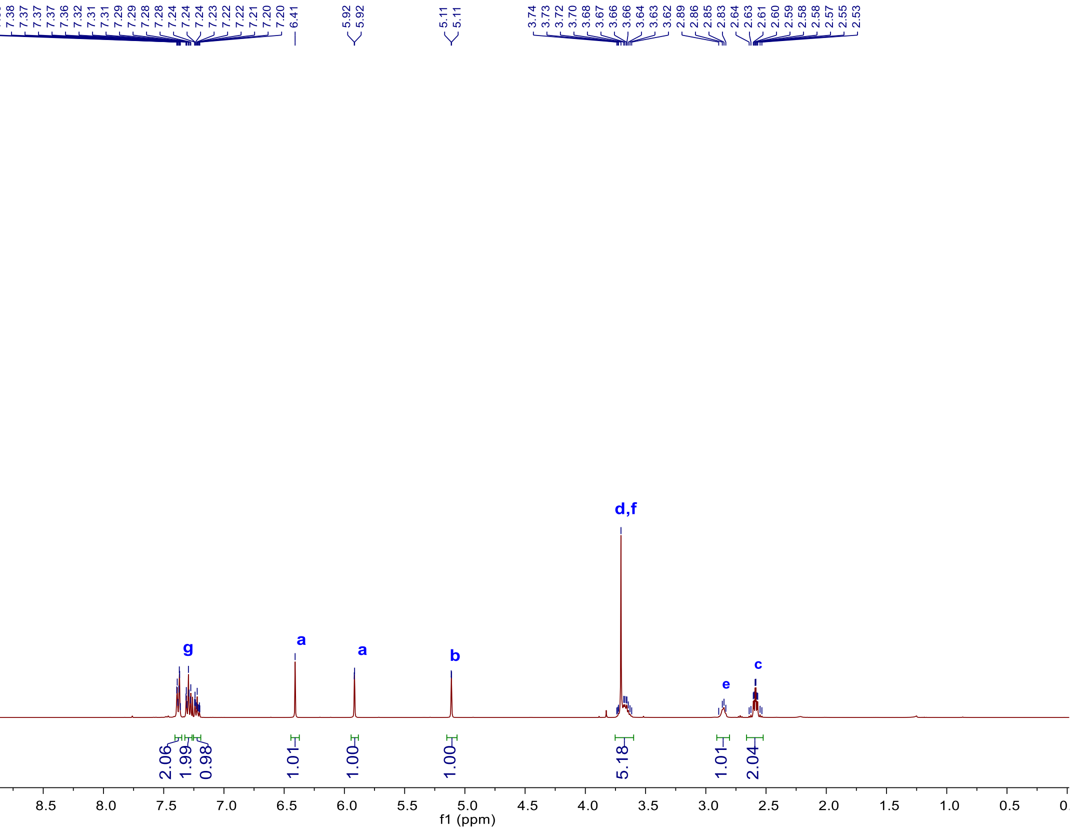

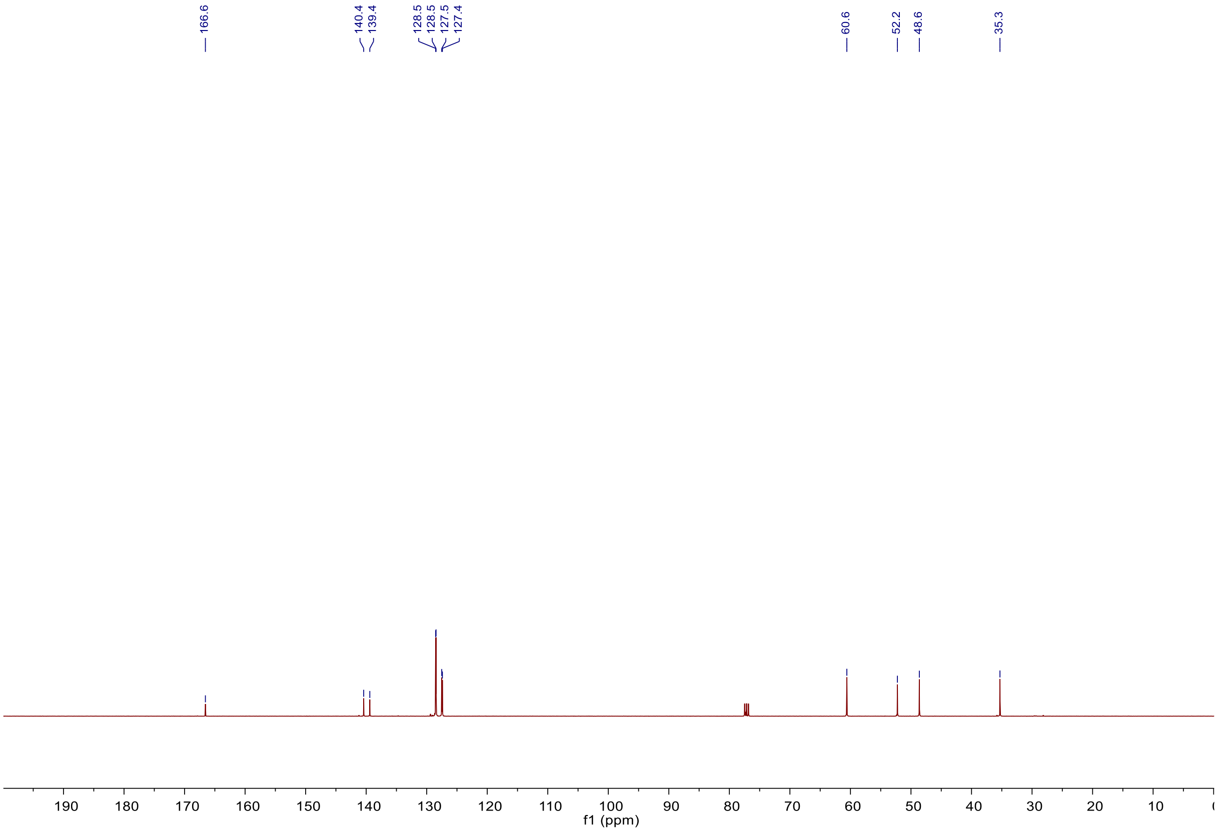

^{1}H NMR ($CDCl_3$, 25 °C) and ^{13}C NMR ($CDCl_3$, 25 °C) spectra for **M3** (Scheme S2)

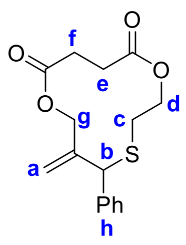

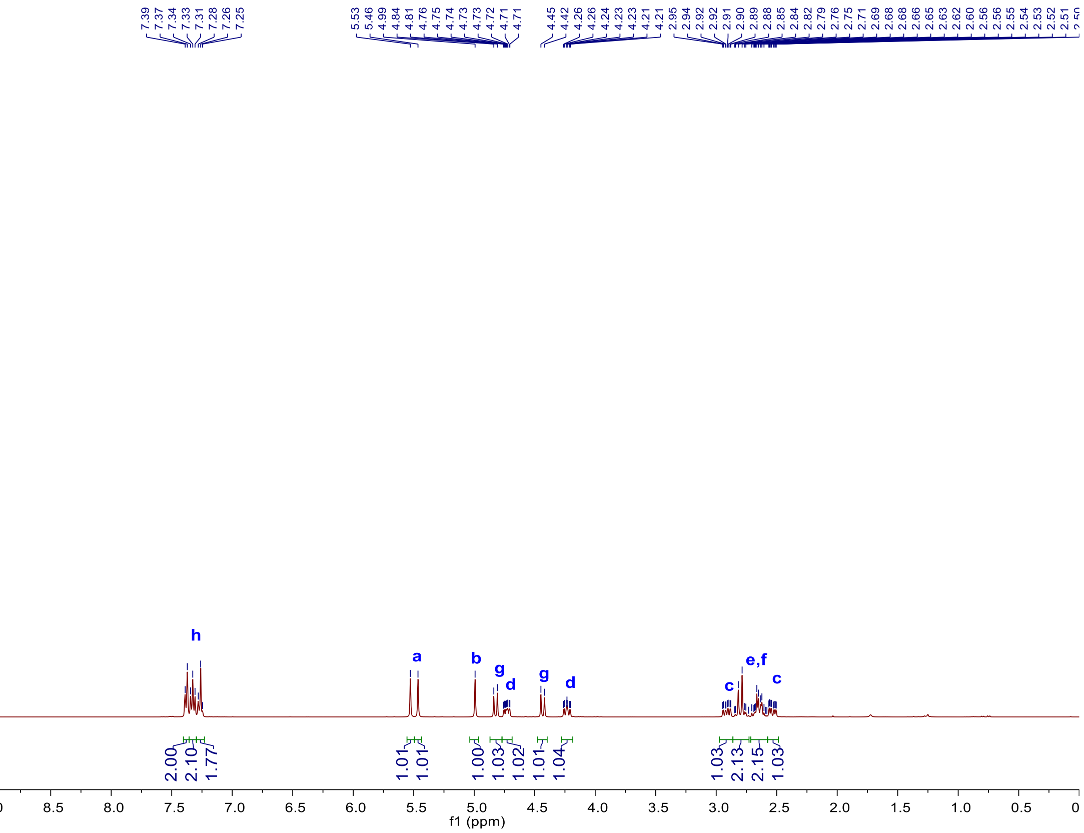

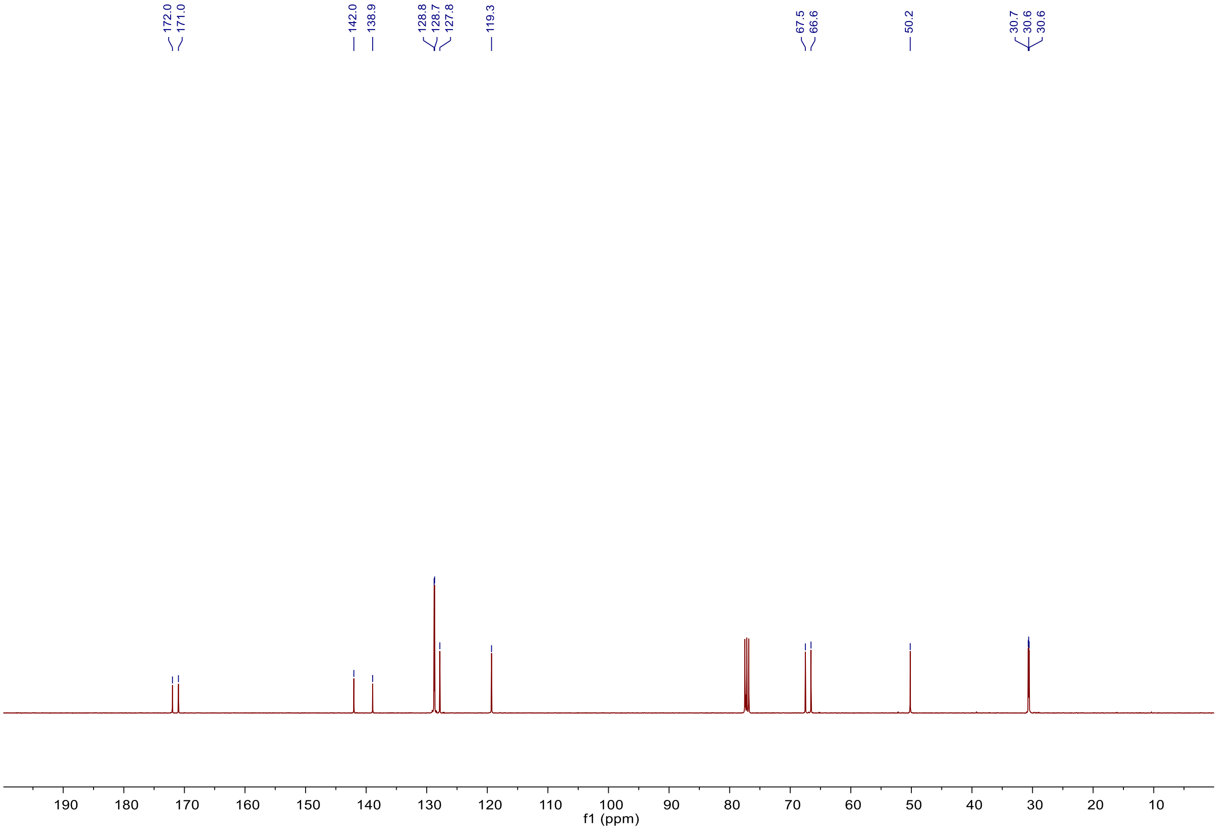

^{1}H NMR ($CDCl_3$, 25 °C) and ^{13}C NMR ($CDCl_3$, 25 °C) spectra for **M4** (Scheme S2)

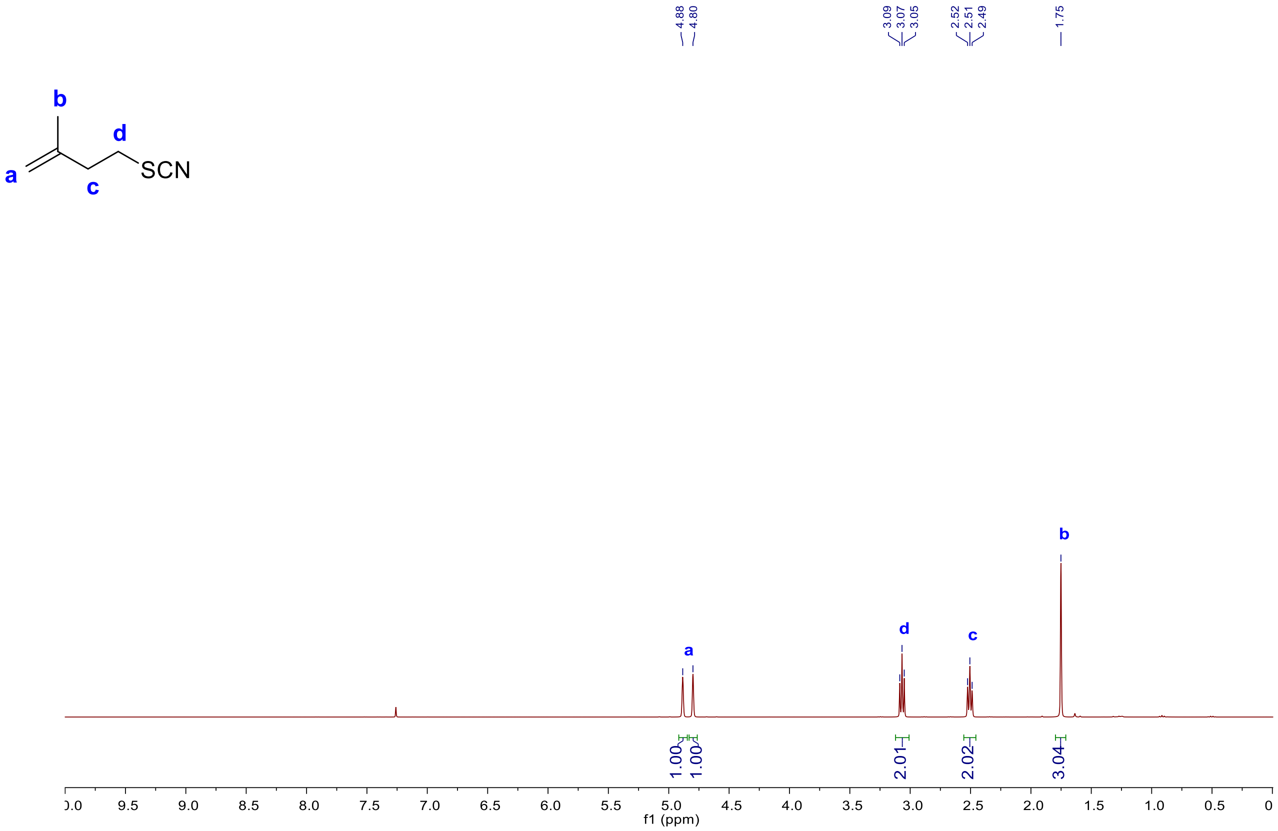

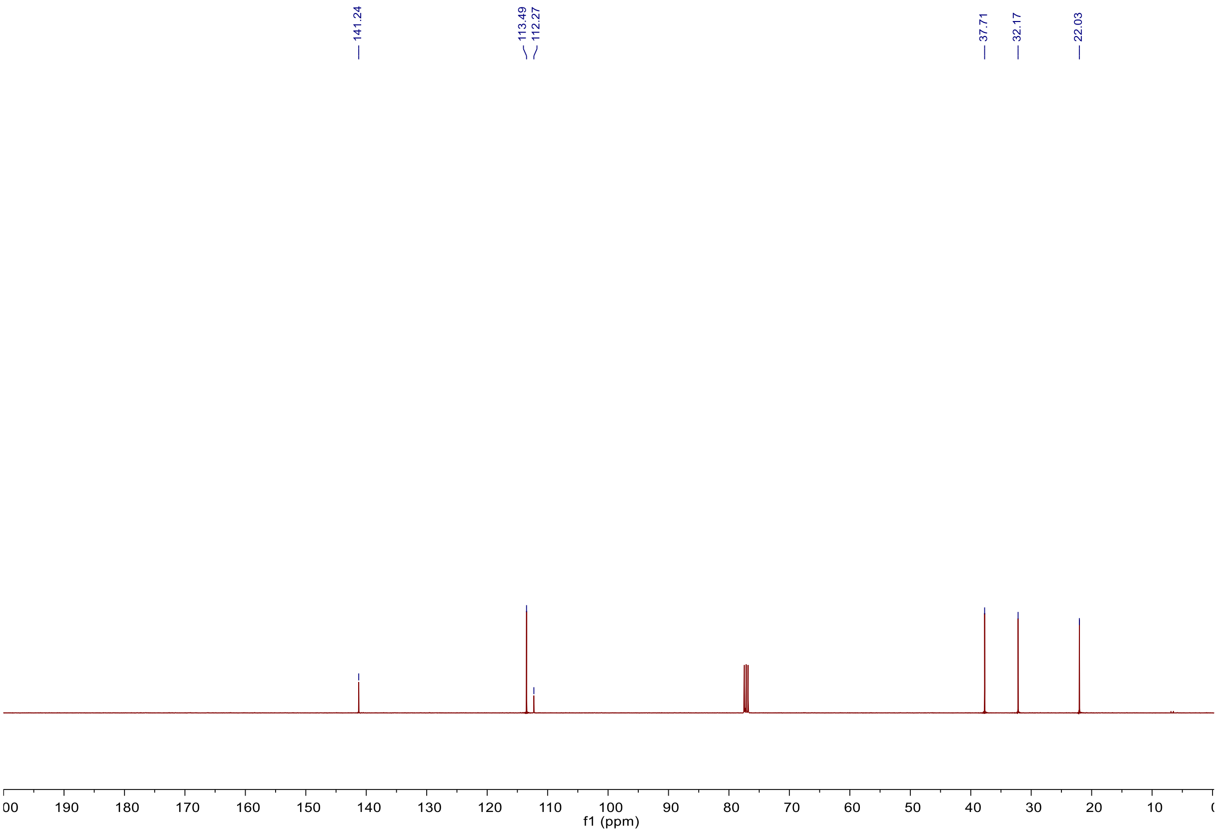

^{1}H NMR ($CDCl_3$, 25 °C) and ^{13}C NMR ($CDCl_3$, 25 °C) spectra for **S4** (Scheme S2)

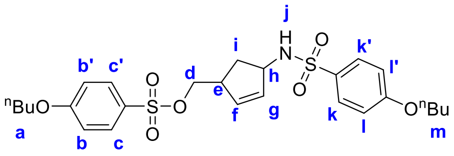

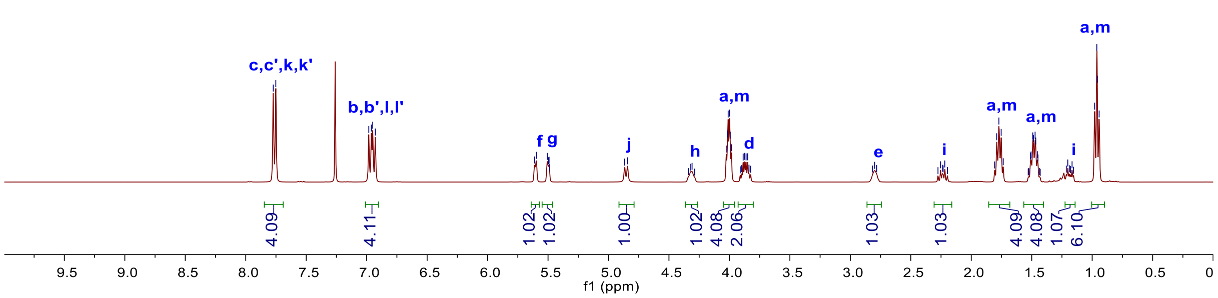

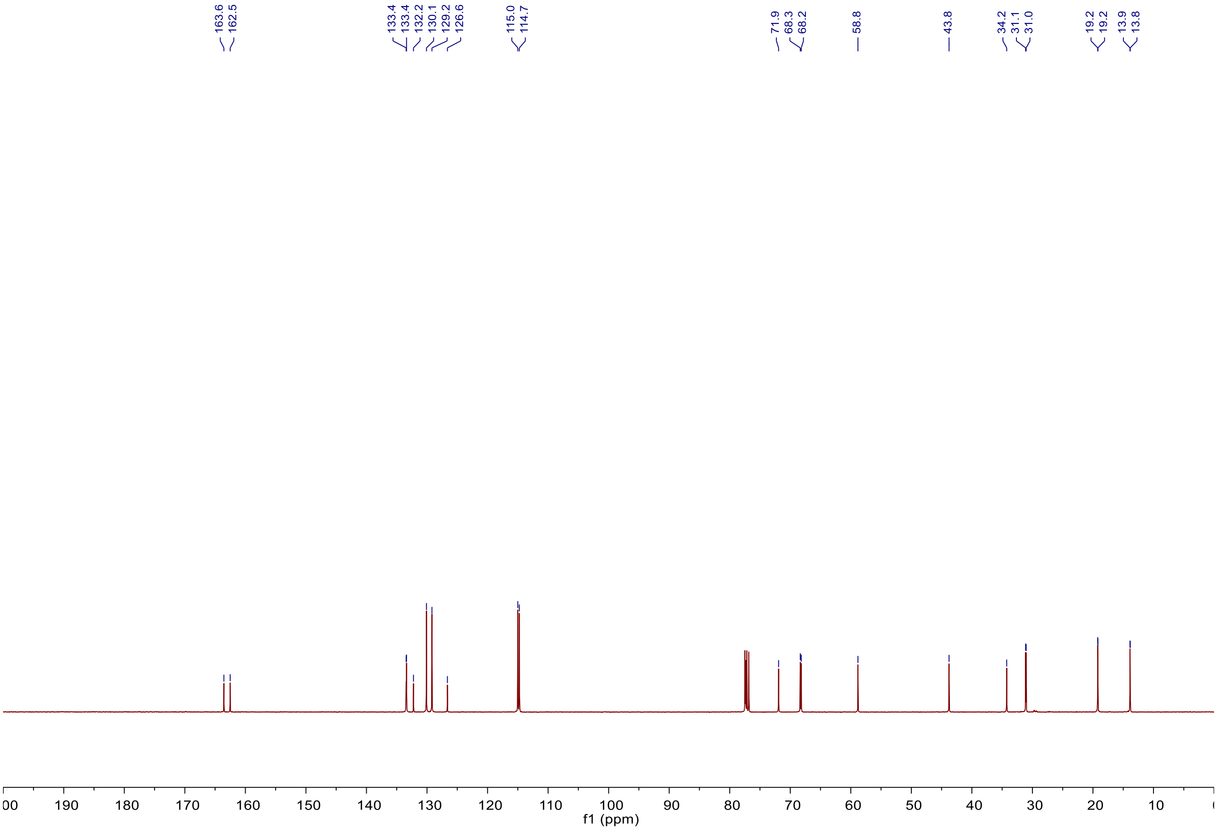

^{1}H NMR ($CDCl_3$, 25 °C) and ^{13}C NMR ($CDCl_3$, 25 °C) spectra for **M5** (Scheme S2)

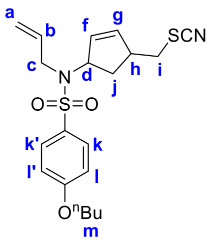
SCN
N
O=S=O
OⁿBu
a
b
c
d
f
g
h
i
j
k
k'
l
l'
m

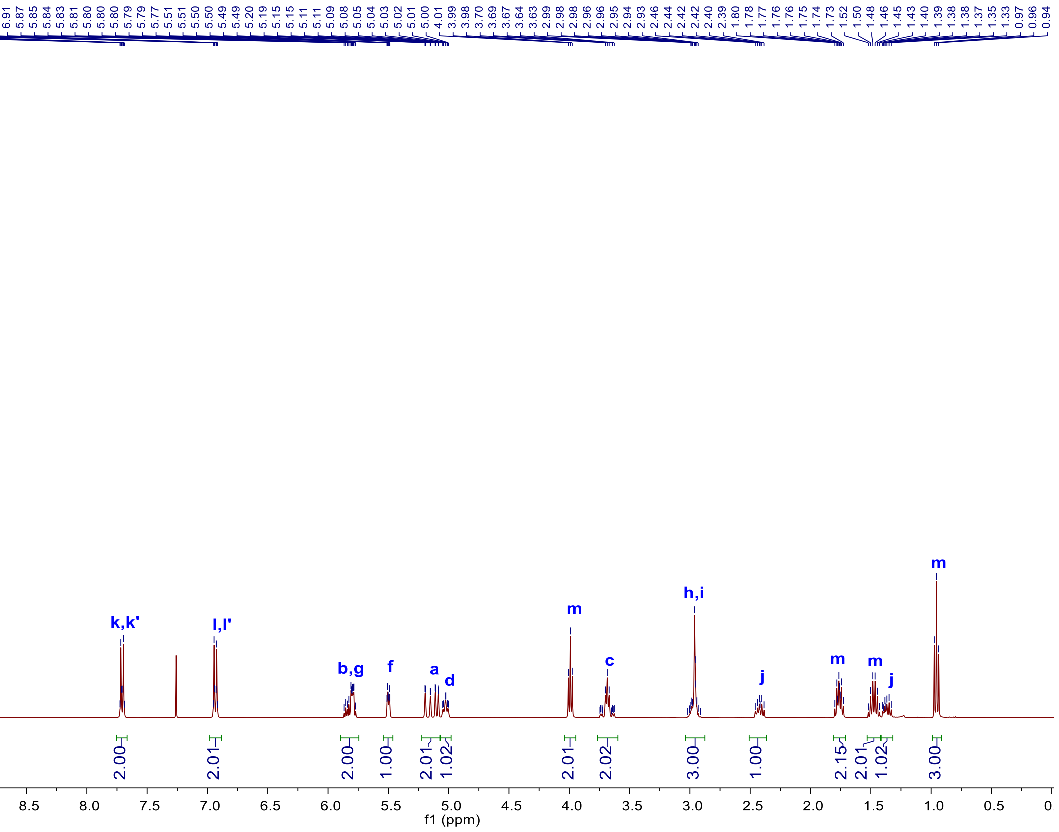
k,k'
l,l'
b,g
f
a
d
m
c
h,i
j
2.00
2.01
1.00
1.02
2.02
3.00
2.15
f1 (ppm)

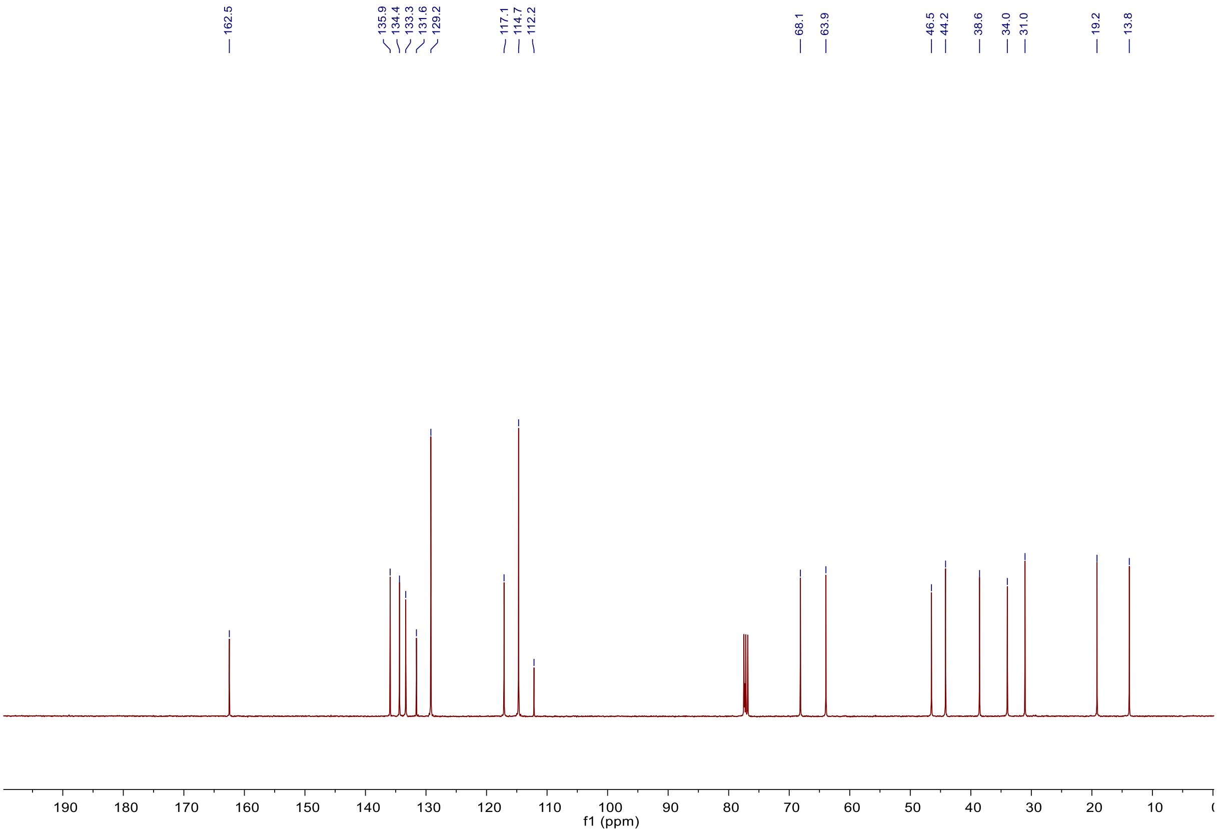
162.5
135.9
134.4
133.3
131.6
129.2
117.1
114.7
112.2
68.1
63.9
46.5
44.2
38.6
34.0
31.0
19.2
13.8
f1 (ppm)

^{1}H NMR ($CDCl_3$, 25 °C) and ^{13}C NMR ($CDCl_3$, 25 °C) spectra for **P1** (Scheme S3)

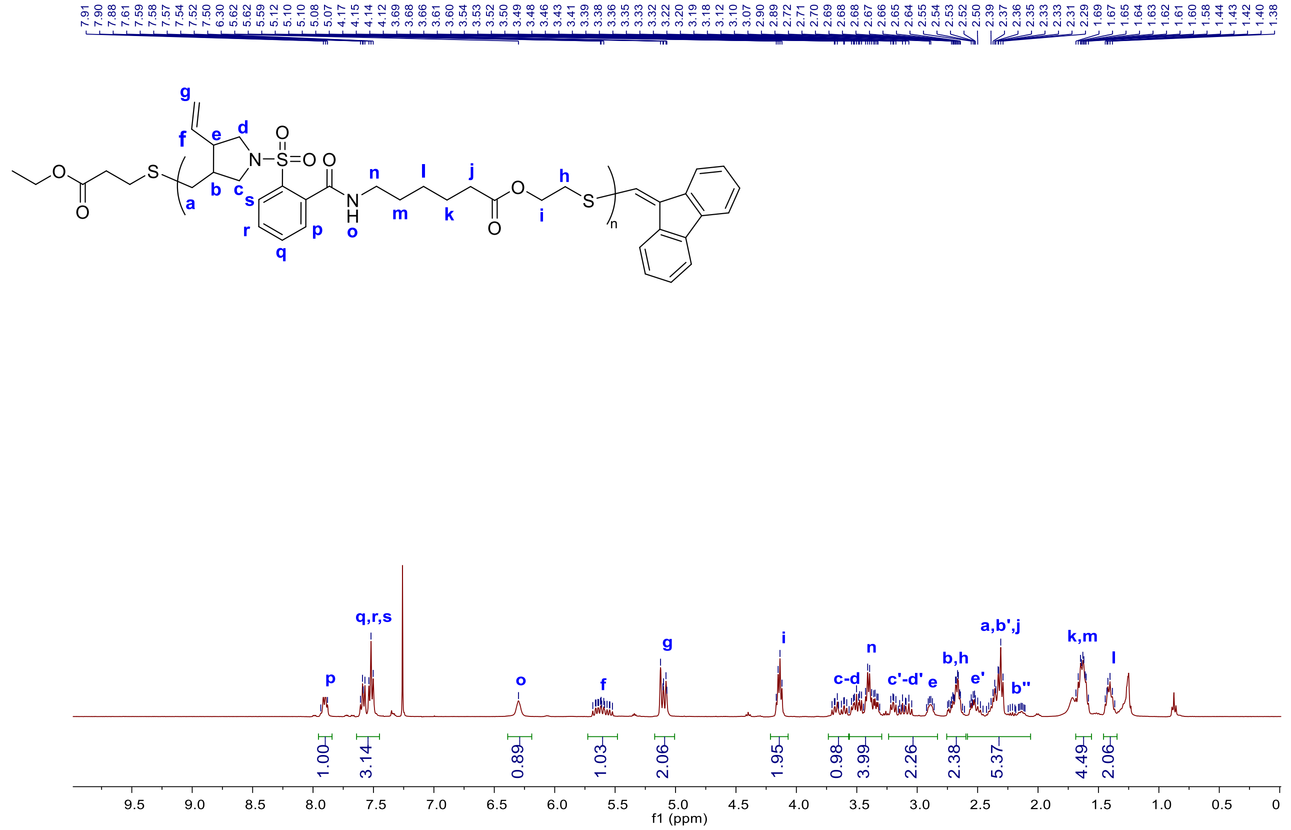

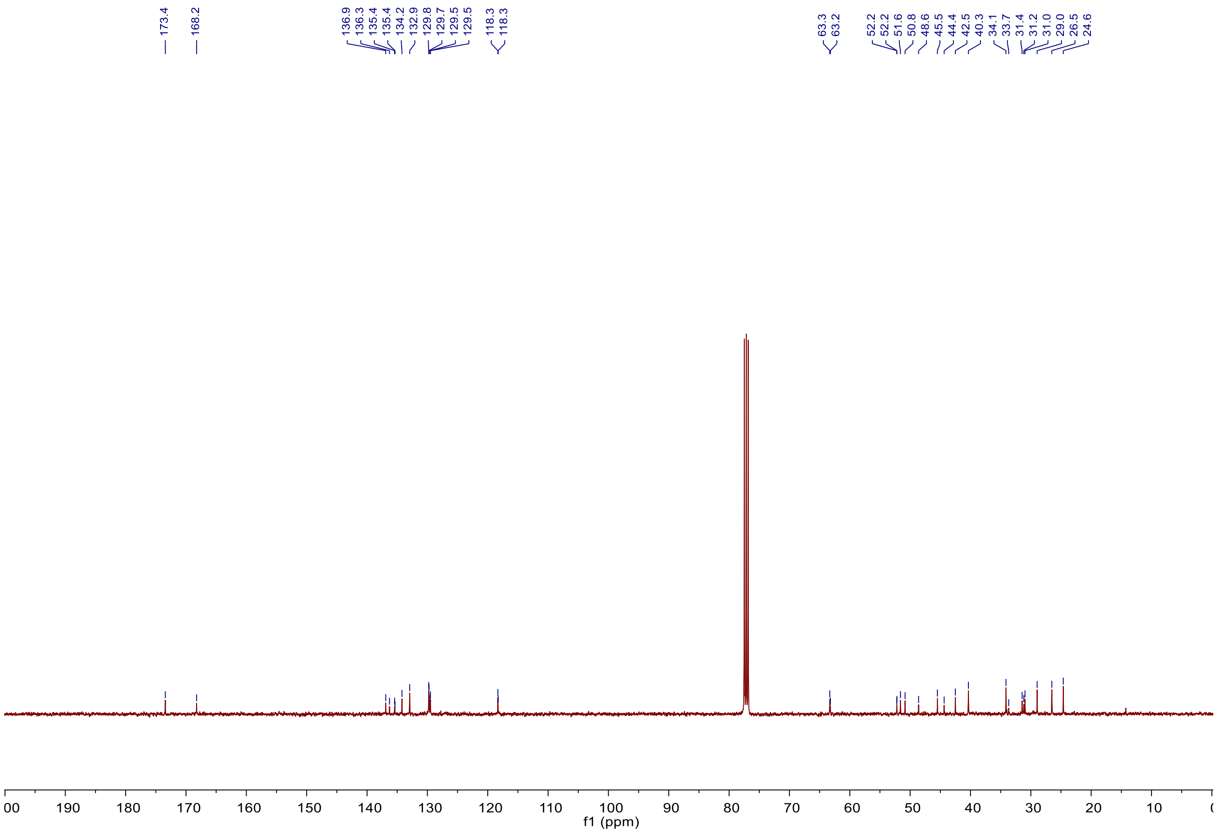

^{1}H NMR ($CDCl_3$, 25 °C) and ^{13}C NMR ($CDCl_3$, 25 °C) spectra for **P2** (Scheme S3)

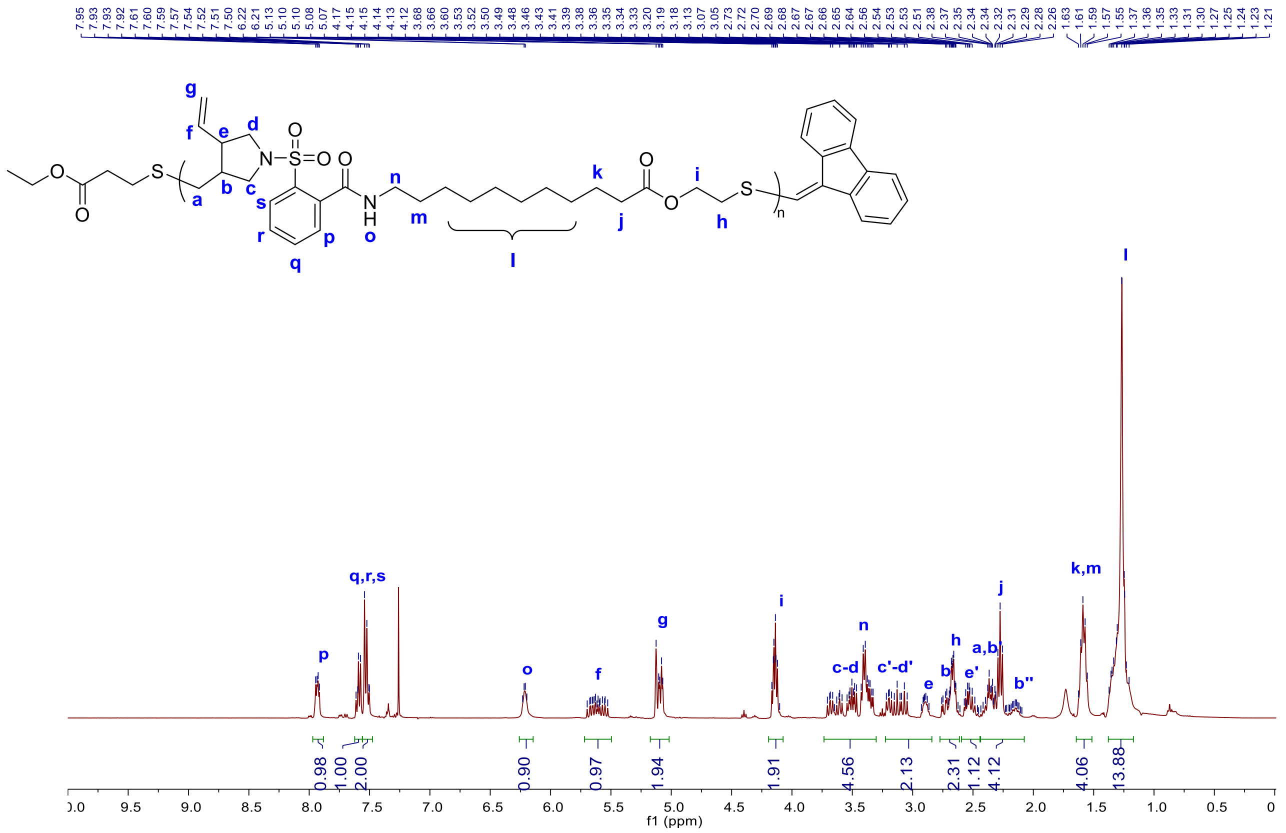

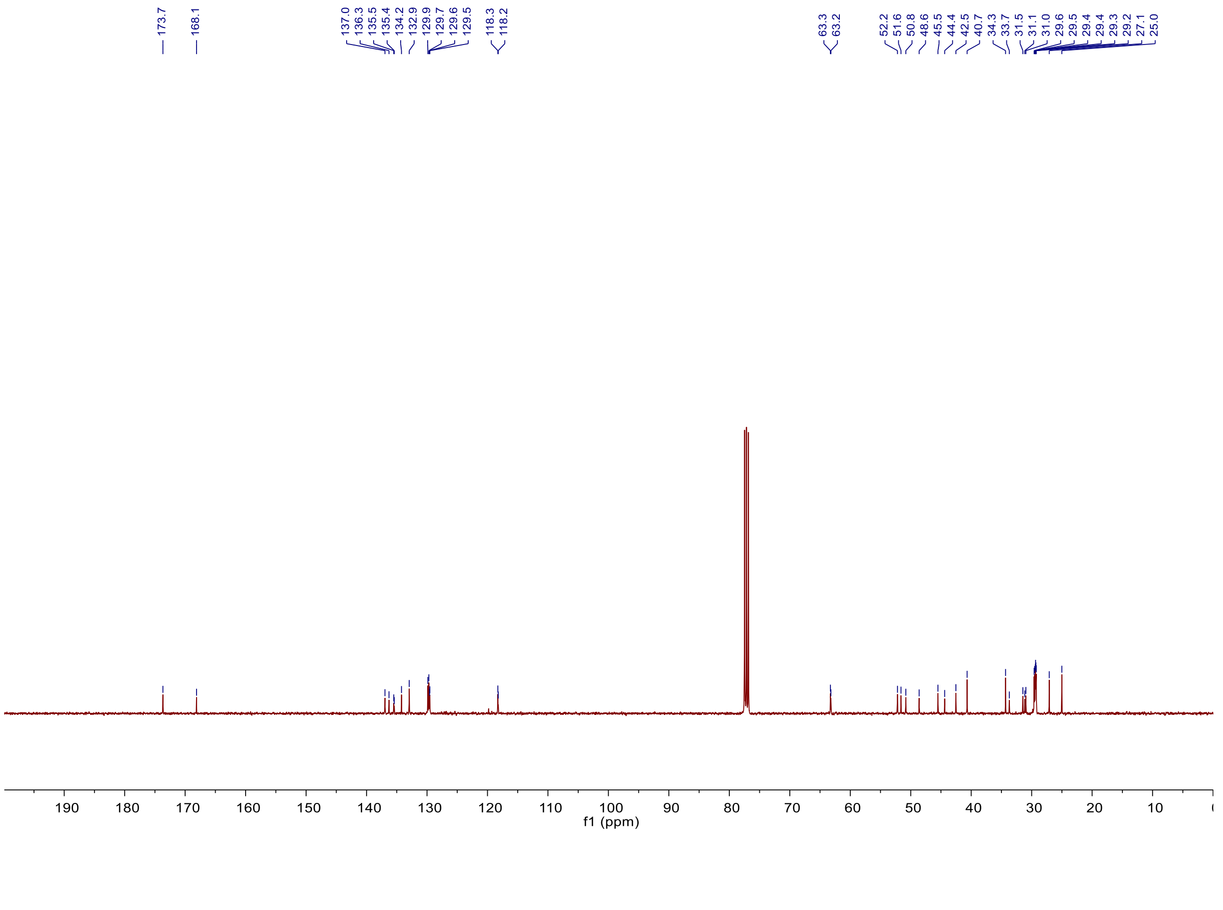

^{1}H NMR ($CDCl_3$, 25 °C) and ^{13}C NMR ($CDCl_3$, 25 °C) spectra for **P3** (Scheme S3)

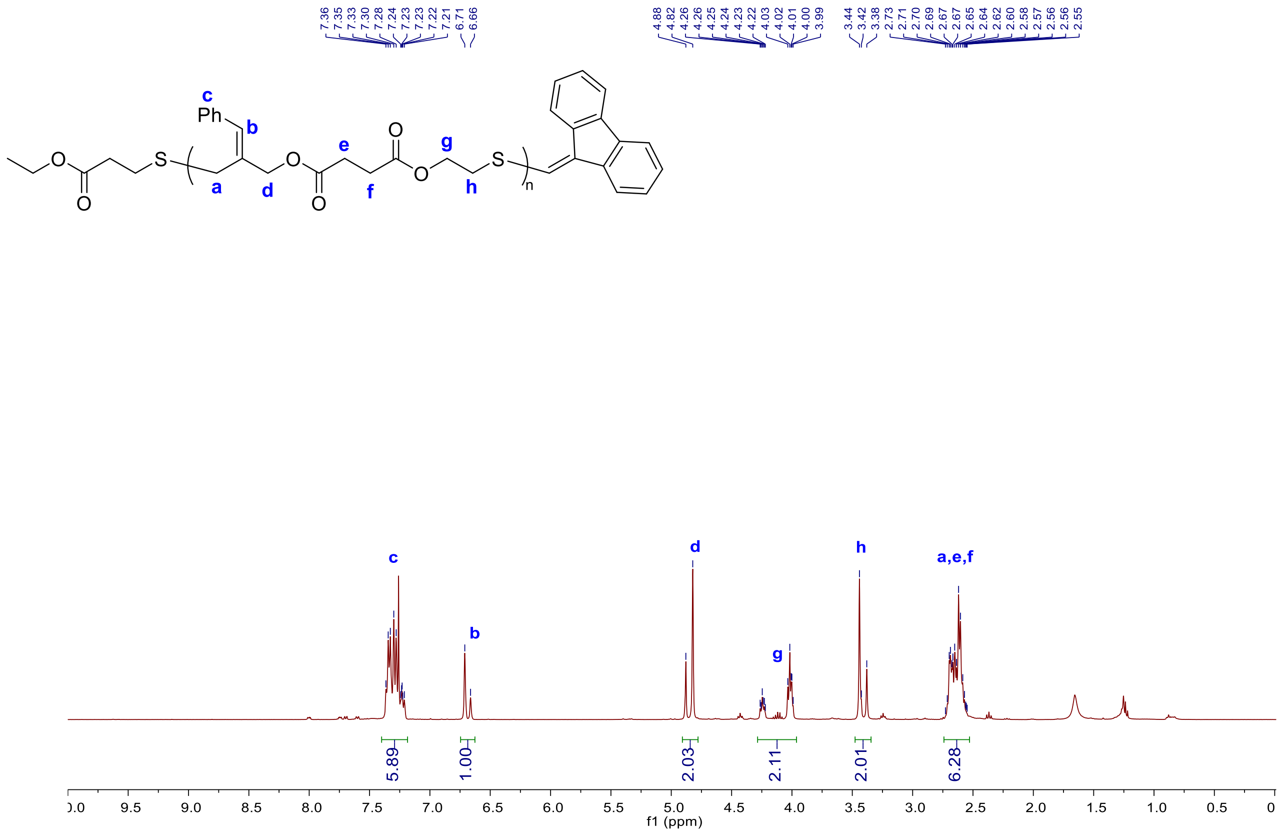

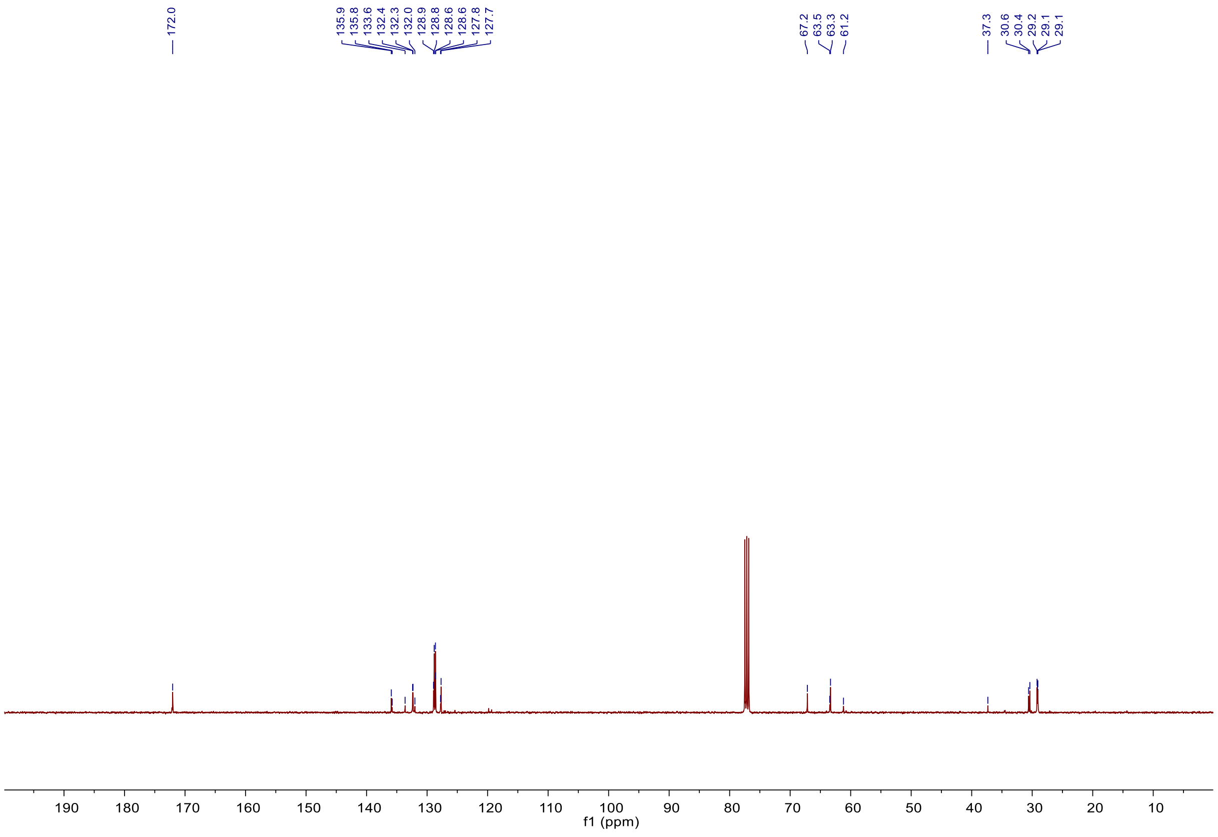

^{1}H NMR ($CDCl_3$, 25 °C) and ^{13}C NMR ($CDCl_3$, 25 °C) spectra for **P4** (Scheme S3)

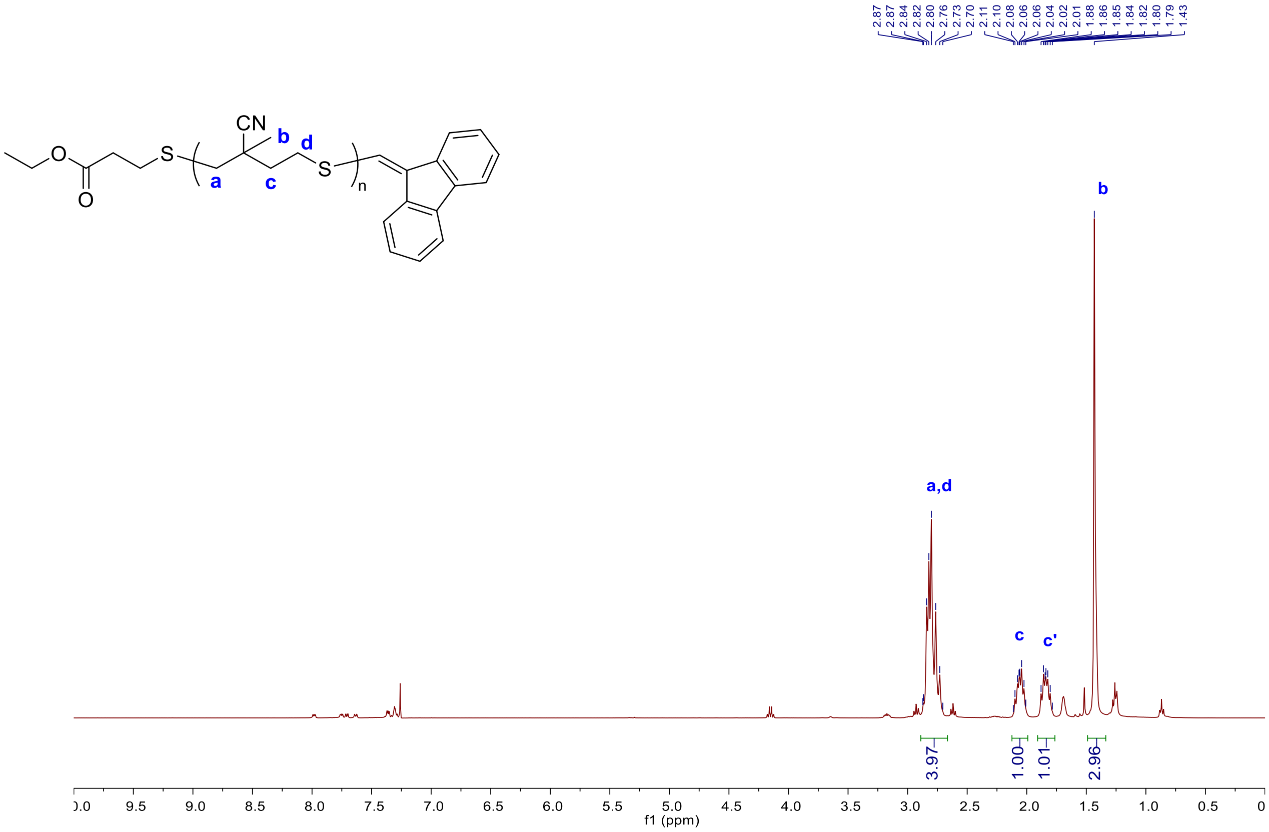

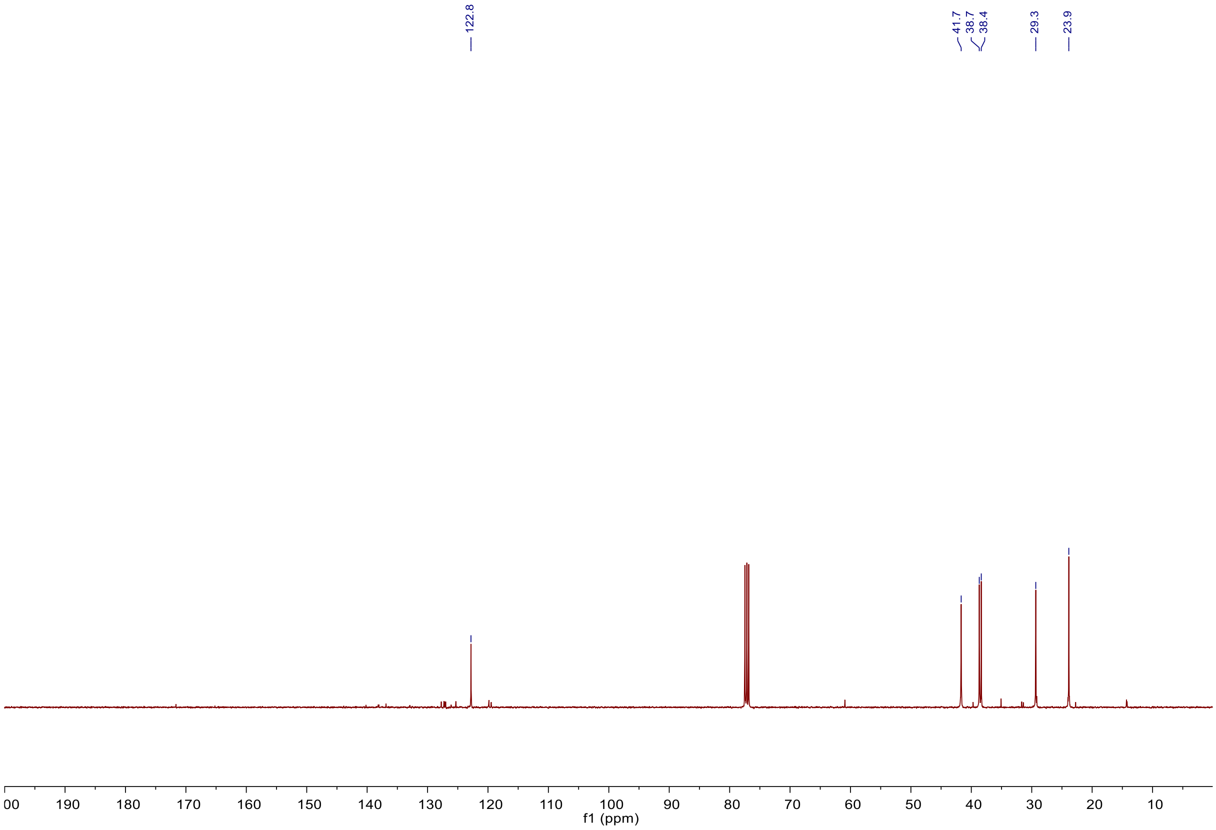

^{1}H NMR ($CDCl_3$, 25 °C) and ^{13}C NMR ($CDCl_3$, 25 °C) spectra for **P5** (Scheme S3)

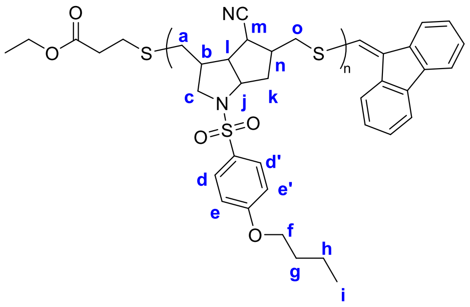

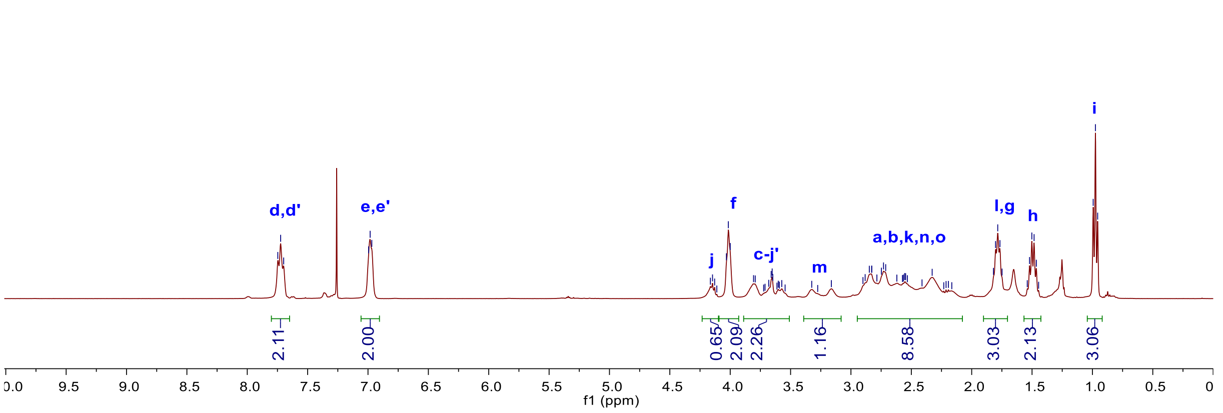

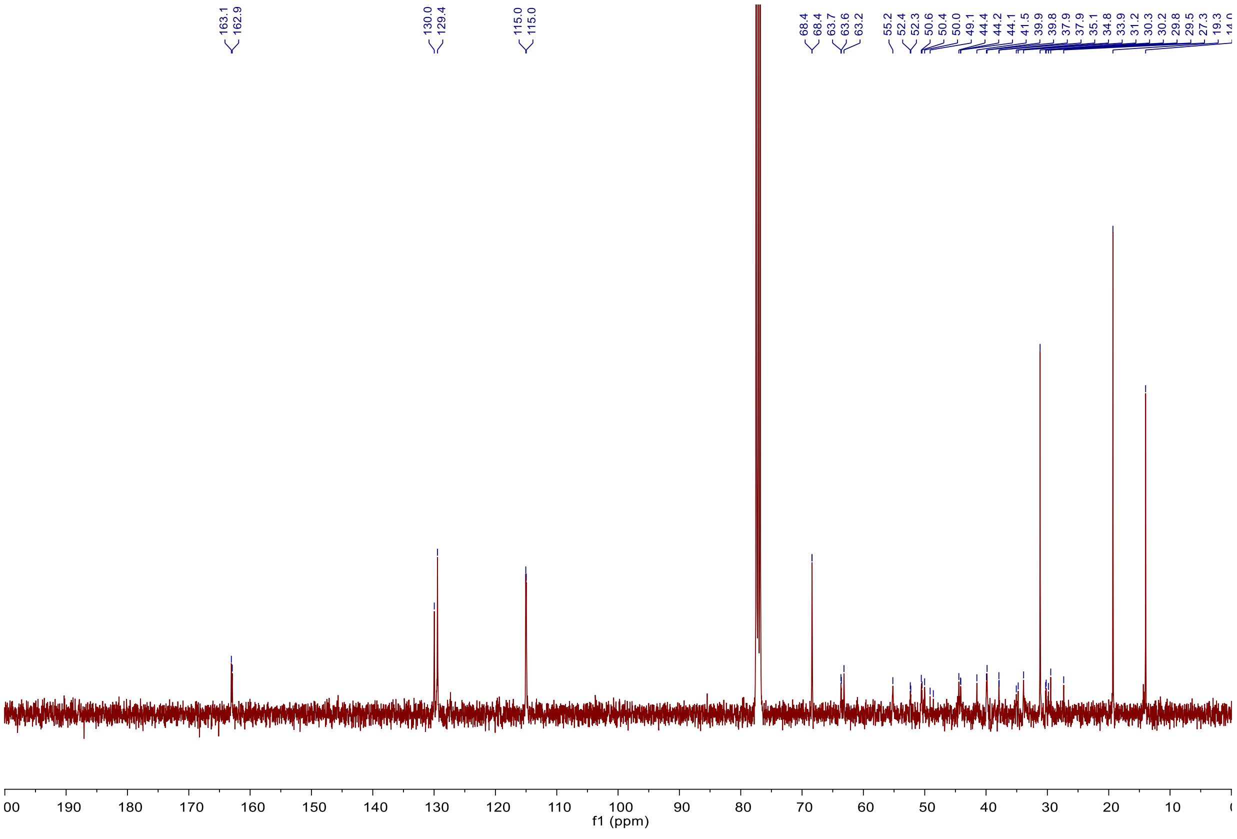

^{1}H NMR ($CDCl_3$, 25 °C) and ^{13}C NMR ($CDCl_3$, 25 °C) spectra for **P1-*b*-P2** (Scheme S6)

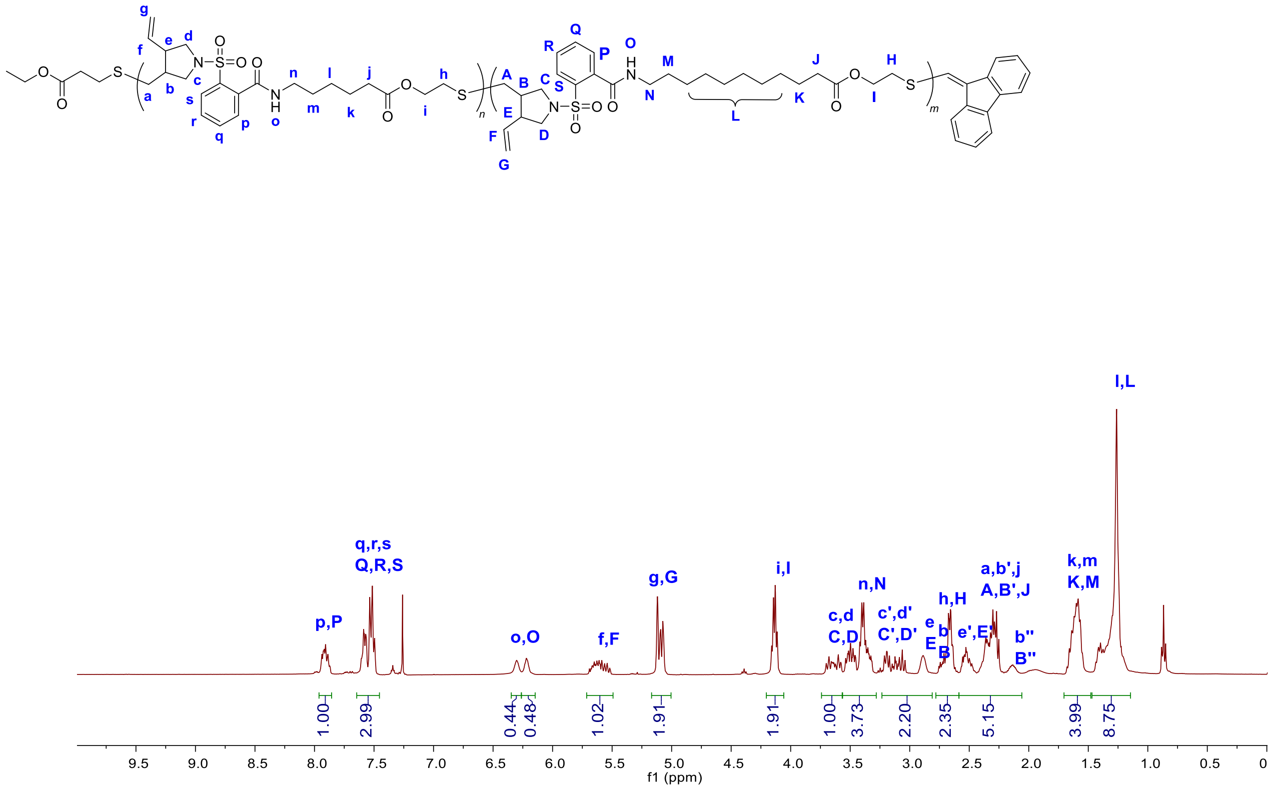

^{1}H NMR ($CDCl_3$, 25 °C) and ^{13}C NMR ($CDCl_3$, 25 °C) spectra for **P4-*b*-P1** (Scheme S11)

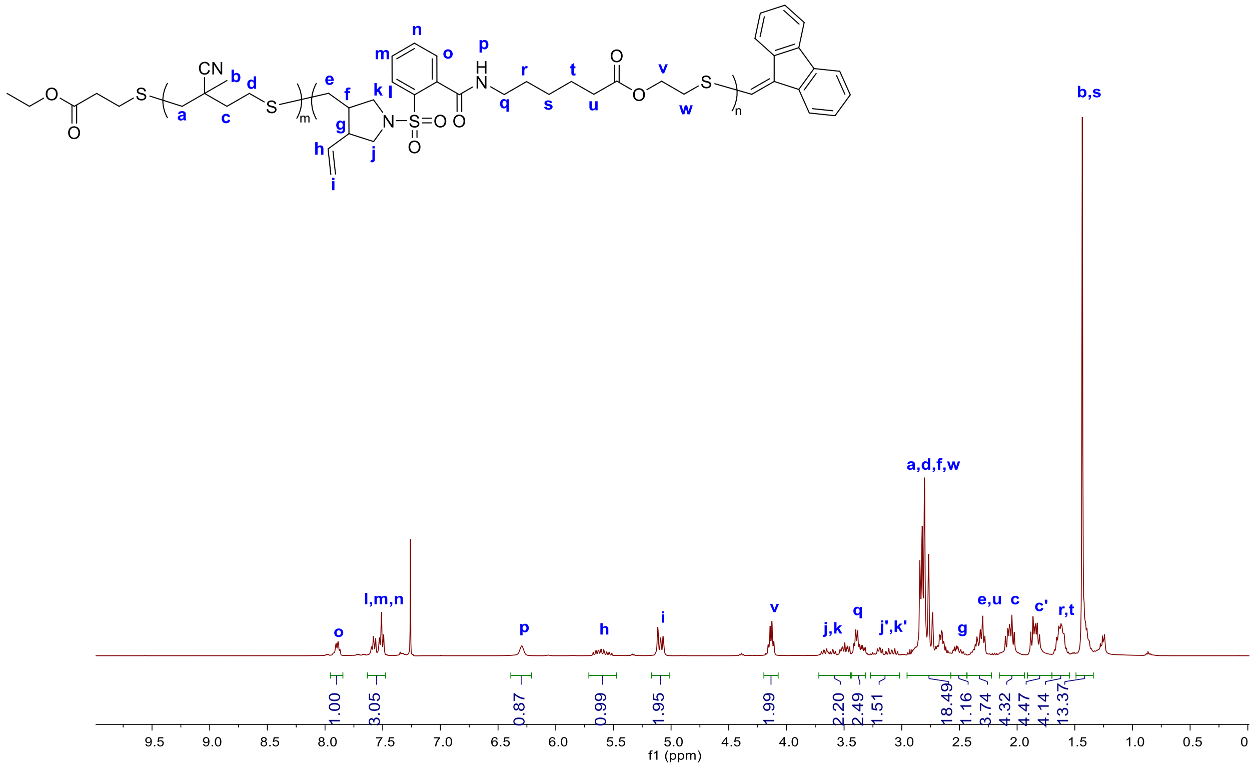

^{1}H NMR ($CDCl_3$, 25 °C) and ^{13}C NMR ($CDCl_3$, 25 °C) spectra for **P(1-*stat*-4)** (Scheme S13)

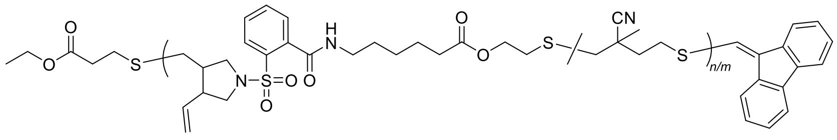

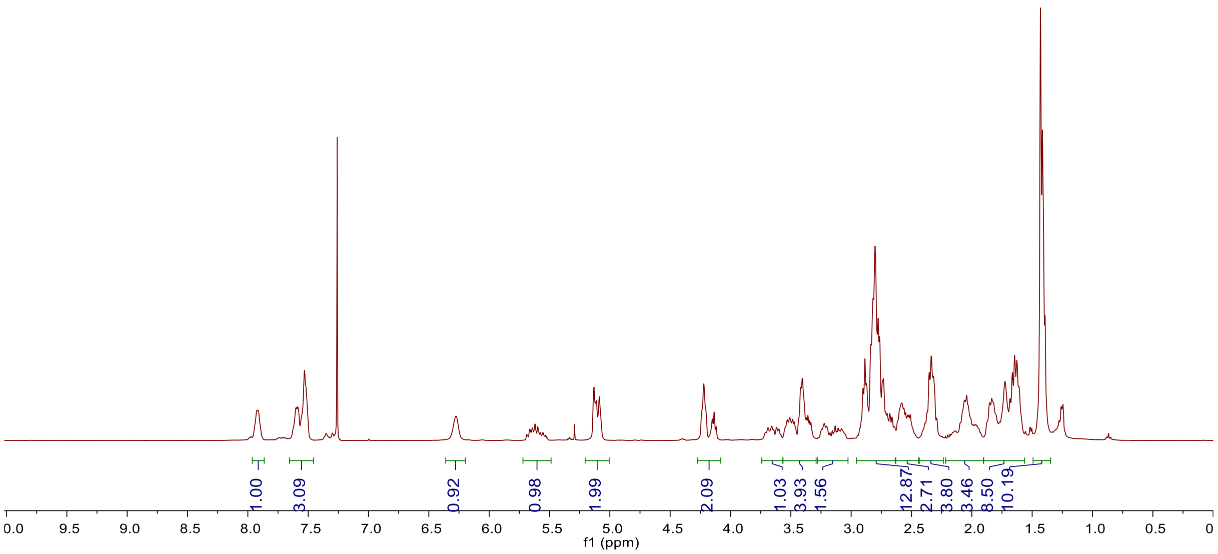

Cartesian Coordinates of All Optimized Structures

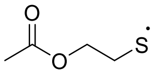

Int0

S	-0.68503100	-0.51128400	-2.53367500
C	-0.44483100	-1.79951200	-1.30696100
H	-1.02821700	-2.67895000	-1.62761300
H	-0.90311100	-1.45414800	-0.36502600
C	1.01120700	-2.16050400	-1.10094800
H	1.46329100	-2.52566800	-2.03516500
H	1.58992500	-1.28828600	-0.76213100
O	1.05672200	-3.18477100	-0.10982800
C	2.27274500	-3.64473000	0.21676000
C	2.19430000	-4.71367100	1.26619300
O	3.28062400	-3.22685200	-0.29011000
H	3.20066700	-5.07143700	1.50529000
H	1.71248600	-4.30880400	2.16693600
H	1.57359000	-5.54330500	0.90063200

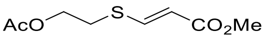

CTA1-P

H	6.22519500	-3.04231400	0.76488200
C	5.15397400	-3.07805900	0.98622300
C	4.40977700	-2.19405600	0.03076500
H	4.96756600	-2.74329600	2.01601100
H	4.78202900	-4.10815800	0.89781800
O	3.08945800	-2.21206400	0.27129900
O	4.89609300	-1.53706800	-0.85130500
C	2.28391800	-1.40477700	-0.57971800
C	0.85237400	-1.58612500	-0.11830700
H	2.59931400	-0.35285500	-0.50444800
H	2.41136700	-1.72264000	-1.62586200
S	-0.23118200	-0.57209000	-1.17580600
H	0.74497000	-1.26025400	0.92504400
H	0.55574800	-2.64013400	-0.20410700
C	-1.77438100	-0.93552500	-0.46734000
C	-2.94056000	-0.42581300	-0.89499100
H	-1.77221800	-1.62759200	0.38236900
C	-4.18816500	-0.81355900	-0.20573000
H	-3.01426000	0.26780800	-1.73470500
O	-4.26775800	-1.56777800	0.73446900
O	-5.25135000	-0.21880400	-0.75500600
C	-6.51026200	-0.52192300	-0.16764800
H	-6.52964100	-0.21962900	0.88851200
H	-7.25921200	0.04230700	-0.73286700
H	-6.72128900	-1.59818500	-0.23370100

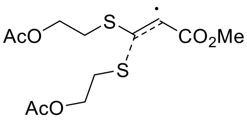

TS1

H	6.22323400	-2.93028300	0.81685600
C	5.15836700	-2.94809800	1.06887800
C	4.38271600	-2.17768600	0.04284900
H	4.99663800	-2.50033000	2.05931800
H	4.79335000	-3.98355900	1.10688300
O	3.06620200	-2.19814400	0.30382200
O	4.84349400	-1.59942000	-0.90555500
C	2.23522300	-1.48375900	-0.60440000
C	0.81307200	-1.65927000	-0.11127300
H	2.53154400	-0.42311100	-0.62052400
H	2.36234100	-1.88741400	-1.62069800
S	-0.33136300	-0.84758800	-1.26976300
H	0.68142900	-1.19436600	0.87748900
H	0.56058300	-2.72664000	-0.04642200
C	-1.80828400	-0.99593900	-0.34804600
C	-2.98401300	-0.42378500	-0.79971400
H	-1.87785000	-1.85685000	0.32400300

C	-4.25012200	-0.82143600	-0.16437100
H	-3.00131500	0.34637900	-1.57340400
O	-4.35495700	-1.65087100	0.70846000
O	-5.28625800	-0.14720200	-0.66822900
C	-6.56031000	-0.46081400	-0.11903900
H	-6.58351900	-0.24044300	0.95706900
H	-7.28688100	0.16594500	-0.64597800
H	-6.79807500	-1.52268000	-0.27075100
H	1.00764300	1.03899200	0.42872400
C	0.52840000	1.95126200	0.80878600
C	-0.97317400	1.89020500	0.62224100
H	0.79663000	2.06021000	1.87134100
O	1.00341500	3.08118900	0.07851400
S	-1.69905400	0.48742500	1.50451500
H	-1.44868500	2.81549700	0.97827400
H	-1.21148900	1.78817800	-0.44901000
C	2.32996600	3.18097400	-0.07672900
C	2.70315600	4.41092100	-0.85003300
O	3.10007600	2.36764900	0.36518800
H	3.78848200	4.44717400	-0.98641800
H	2.19658600	4.40174300	-1.82474400
H	2.36094200	5.30156500	-0.30470100

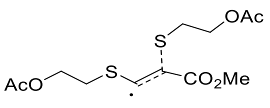

TS1'

H	6.51073500	0.16516100	0.27473100
C	5.50114900	-0.04434100	0.64518400
C	4.50457900	0.75496800	-0.13836100
H	5.42821600	0.16468800	1.71887900
H	5.27810000	-1.10849100	0.47753300
O	3.37741300	0.98138300	0.55910000
O	4.65528700	1.15043300	-1.26328100
C	2.31850600	1.58213700	-0.17508100
C	1.11410600	1.61455200	0.74654400
H	2.60896000	2.59459500	-0.49515000
H	2.11973800	0.98151900	-1.07549100
S	-0.33111300	2.23647300	-0.18417800
H	1.29960300	2.26017900	1.61528000
H	0.87515500	0.60114000	1.10014000
C	-1.56912600	1.21289300	0.42547700
C	-2.78327500	1.03193900	-0.20020100
H	-1.35420900	0.65519600	1.34294800
C	-3.84287200	0.32985000	0.57663400
H	-3.10697500	1.68719600	-1.01133600
H	3.72359100	-2.82677400	1.27507000
C	2.79593100	-2.28529900	1.49497500
C	2.12730500	-1.89039800	0.21194900
H	3.05294100	-1.36038400	2.03417600
H	2.13316300	-2.89106900	2.12230200
O	0.79286300	-1.93818400	0.30421300
O	2.69856100	-1.53579200	-0.78921300
C	0.04465600	-1.49013700	-0.82889100
C	-1.42371000	-1.75688600	-0.55575700
H	0.22513100	-0.41507600	-0.98143000
H	0.38611200	-2.01253900	-1.73433600
S	-2.50265100	-0.83398000	-1.67678000
H	-1.68492400	-1.48608300	0.47948000
H	-1.65451500	-2.82690400	-0.67013000
C	-6.12472200	-0.15229200	0.69027100
H	-7.03530400	0.15100300	0.16396900
H	-5.97763400	-1.23711400	0.59466400
H	-6.19828000	0.10949400	1.75427300
O	-5.05159900	0.54884200	0.07080300
O	-3.63326400	-0.35678300	1.54785500

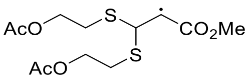

Int1

H	6.24344000	-3.04167000	0.60389900

C	5.20068100	-3.03650800	0.93623500
C	4.35646100	-2.29659900	-0.05815100
H	5.11890000	-2.55092300	1.91857800
H	4.83019300	-4.06552600	1.04067300
O	3.06575400	-2.28105800	0.30649300
O	4.74996000	-1.76944300	-1.06526000
C	2.17289400	-1.59666900	-0.56757100
C	0.79779100	-1.68159900	0.06300100
H	2.49847300	-0.55153400	-0.68478300
H	2.19480700	-2.07103400	-1.56093800
S	-0.42223500	-0.90415300	-1.03822200
H	0.78808100	-1.14463300	1.02351600
H	0.52246300	-2.73145200	0.23755300
C	-1.78975600	-0.69547500	0.14080400
C	-2.99613900	-0.23524100	-0.57477800
H	-1.99776800	-1.65128300	0.64263500
C	-4.29745900	-0.76458300	-0.18467400
H	-2.94401900	0.55716400	-1.32361800
O	-4.46680100	-1.61849800	0.65906200
O	-5.29581000	-0.19929400	-0.87110700
C	-6.60406000	-0.66406700	-0.56349900
H	-6.84564900	-0.47600700	0.49186900
H	-7.28967500	-0.10678800	-1.20995400
H	-6.69097300	-1.74122800	-0.76277500
H	1.10812200	1.14166200	0.20964000
C	0.66335700	2.00197100	0.72907400
C	-0.84938900	1.93192000	0.69334800
H	1.03727000	2.01060800	1.76461600
O	1.04448500	3.20872800	0.07112500
S	-1.46110800	0.45274600	1.55701900
H	-1.28712500	2.80609300	1.19477900
H	-1.21082800	1.91997800	-0.34501700
C	2.35791600	3.39258100	-0.12146700
C	2.63688300	4.69284700	-0.81521300
O	3.18414900	2.59299700	0.23423100
H	3.71371100	4.80141300	-0.97810200
H	2.10208800	4.72106200	-1.77439200
H	2.26208800	5.52271100	-0.20000800

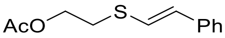

CTA2-P

H	6.36613200	-3.26257700	0.88115400
C	5.30916300	-3.12987700	1.13227300
C	4.60479400	-2.43404500	0.00590400
H	5.20916600	-2.53493100	2.05056300
H	4.83705800	-4.10535300	1.31359400
O	3.30371500	-2.25204900	0.27725200
O	5.10733400	-2.07003100	-1.02443500
C	2.53585600	-1.59614500	-0.72666900
C	1.12091000	-1.49340000	-0.19686200
H	2.96333100	-0.60205800	-0.92898700
H	2.57000400	-2.17581300	-1.66154200
S	0.09134800	-0.63131700	-1.42702900
H	1.11044900	-0.93658300	0.75021800
H	0.70645200	-2.49729700	-0.02747400
C	-1.49534800	-0.90701100	-0.73198300
C	-2.56107000	-0.14570100	-1.02696400
H	-1.56831200	-1.77600900	-0.06967300
C	-3.93557000	-0.37414100	-0.55166400
C	-4.31757600	-1.51668100	0.17430900
C	-4.91437300	0.59395600	-0.83019700
C	-5.62842400	-1.67276200	0.61507800
H	-3.58566100	-2.29711200	0.39116600
C	-6.22795800	0.43528400	-0.39078100
C	-6.59083700	-0.69831900	0.33598000
H	-5.90451100	-2.56698500	1.17716200
H	-6.97133400	1.20160000	-0.61851000
H	-7.61847500	-0.82644300	0.68040400
H	-2.42602600	0.73083500	-1.66963900
H	-4.63356000	1.48386900	-1.39842600

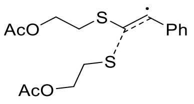

TS2

H	6.38509200	-3.32495500	0.77746400
C	5.31366400	-3.29052100	0.99812600
C	4.62079200	-2.40567300	0.00537100
H	5.14813100	-2.90228900	2.01267100
H	4.88392900	-4.30067900	0.95297500
O	3.30062000	-2.34627100	0.23694200
O	5.14610900	-1.80844700	-0.89682900
C	2.54206100	-1.52880900	-0.64876700
C	1.10090500	-1.62482700	-0.19123100
H	2.91549500	-0.49371000	-0.60662900
H	2.66248300	-1.89108900	-1.68139000
S	0.03483500	-0.70135400	-1.33899800
H	0.97774800	-1.19097900	0.81249300
H	0.78189700	-2.67635800	-0.17174700
C	-1.48381500	-0.81602100	-0.46394700
C	-2.59510300	-0.15229000	-0.93777500
H	-1.58197400	-1.69610300	0.17641700
C	-3.96035200	-0.35427000	-0.47852000
C	-4.29820200	-1.25258800	0.55397500
C	-4.99159200	0.38841500	-1.08427300
C	-5.62060300	-1.39931200	0.95697300
H	-3.52199000	-1.83926800	1.04895300
C	-6.31523800	0.23504500	-0.68090600
C	-6.63537400	-0.65910600	0.34175600
H	-5.86518700	-2.09731100	1.75961800
H	-7.10076700	0.81776300	-1.16525300
H	-7.67181500	-0.77961500	0.66163600
S	-1.22449600	0.57304500	1.56514700
C	-0.44165600	1.97419700	0.73044700
H	-0.85540900	2.90908100	1.13698700
H	-0.70752800	1.93565900	-0.33874000
C	1.06443900	1.94775400	0.87926300
H	1.36338300	1.99449000	1.93821400
H	1.48166600	1.02817000	0.44705900
O	1.58802000	3.08141500	0.18584600
C	2.91426100	3.11918200	0.01161600
C	3.33915100	4.35566600	-0.72443700
O	3.64952900	2.25257500	0.41030100
H	4.42401300	4.34610600	-0.86905800
H	2.82620800	4.40105300	-1.69489800
H	3.04225400	5.24338300	-0.14877800
H	-2.45624700	0.61614100	-1.70555800
H	-4.74006400	1.09079900	-1.88216200

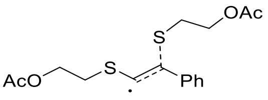

TS2’

H	9.17909500	-1.23889400	1.01993200
C	8.28419400	-0.62481900	1.16080200
C	7.19458300	-1.10262200	0.24857500
H	8.50927900	0.42808300	0.94107800
H	7.94332200	-0.68260700	2.20357600
O	6.07016200	-0.38661500	0.40758500
O	7.27722300	-2.00649900	-0.54016600
C	4.96970700	-0.75505200	-0.41550100
C	3.83488400	0.18433100	-0.06124700
H	5.25045300	-0.66385700	-1.47600700
H	4.69662100	-1.80436500	-0.22628400
S	2.39140400	-0.22019900	-1.09477600
H	4.12813600	1.22564400	-0.25052700
H	3.55818000	0.06943300	0.99550600
C	1.24508300	0.91247300	-0.48854900
C	-0.04278300	0.97801600	-0.98851000
H	1.55151400	1.51028800	0.37517500
C	-0.98979800	2.02295400	-0.54427900
C	-0.99884600	2.50000200	0.77602000

C	-1.92829300	2.52975100	-1.45329200
C	-1.91254400	3.47458900	1.16711100
H	-0.30340200	2.08341400	1.50804000
C	-2.84354600	3.50645900	-1.05975900
C	-2.83769600	3.98320300	0.25056100
H	-1.91136000	3.83400900	2.19781600
H	-3.56585600	3.89364400	-1.78075000
H	-3.55607500	4.74353700	0.56197300
H	-0.23518800	0.55574300	-1.97896000
H	-1.93672500	2.15323700	-2.47879100
H	-8.04252300	-2.41446900	0.27898100
C	-7.29535900	-1.64906700	0.04682700
C	-5.91816200	-2.19926100	0.27540900
H	-7.44719700	-0.76703700	0.68441700
H	-7.39453600	-1.32572600	-0.99838200
O	-4.97298800	-1.29962200	-0.02528100
O	-5.66024800	-3.30326400	0.67938100
C	-3.61881900	-1.71349700	0.16125800
C	-2.73235900	-0.54348800	-0.22043500
H	-3.46531700	-2.00451600	1.21140000
H	-3.41253600	-2.59280400	-0.46715400
S	-0.99208400	-1.00990600	0.01339000
H	-2.98561300	0.32506300	0.40641400
H	-2.91701500	-0.27739700	-1.27238100

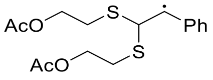

Int2

H	6.52921100	-3.32040000	0.58128300
C	5.47863500	-3.29945700	0.88736500
C	4.68104700	-2.49632600	-0.09650900
H	5.38412300	-2.85274600	1.88683600
H	5.07792200	-4.32116700	0.93919800
O	3.38348000	-2.45343400	0.23944800
O	5.11463500	-1.94515300	-1.07396100
C	2.53281400	-1.70868700	-0.62797000
C	1.13824400	-1.77969600	-0.03987600
H	2.88968100	-0.66918000	-0.69249900
H	2.57004500	-2.14166200	-1.63958600
S	-0.02285700	-0.92113800	-1.14177200
H	1.11520800	-1.28290000	0.94158600
H	0.83066800	-2.82815900	0.08287300
C	-1.42986500	-0.68166700	-0.00838800
C	-2.55119200	-0.10890100	-0.79547600
H	-1.69801200	-1.65673700	0.42515700
C	-3.92648000	-0.25857800	-0.45370600
C	-4.36598800	-0.97774900	0.68792000
C	-4.91466600	0.33792000	-1.28008600
C	-5.72038000	-1.09547100	0.97326500
H	-3.63883100	-1.44029100	1.35726100
C	-6.26456800	0.21238900	-0.98678300
C	-6.67825300	-0.50584400	0.14152500
H	-6.03650500	-1.65270500	1.85715900
H	-7.00575100	0.67741200	-1.63949200
H	-7.74027100	-0.60314600	0.37236300
S	-1.05867100	0.33502700	1.49998800
C	-0.41900800	1.86113000	0.74724900
H	-0.83292100	2.70758900	1.31254100
H	-0.78906500	1.93148100	-0.28610300
C	1.09506800	1.89485200	0.77629100
H	1.47499200	1.82665500	1.80732000
H	1.51569700	1.05972200	0.19865600
O	1.50278900	3.13308500	0.19619200
C	2.82015200	3.30513000	0.02431100
C	3.12612000	4.63815400	-0.59187300
O	3.63045300	2.47255600	0.33898000
H	4.20607300	4.73912900	-0.73818400
H	2.60180700	4.72857300	-1.55303100
H	2.75728800	5.43790200	0.06537000
H	-2.30898000	0.50905200	-1.66443000

H	-4.59380300	0.89983300	-2.16021700

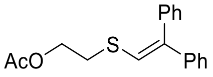

CTA3-P

H	-7.07463600	-3.30340200	0.55382300
C	-6.01539800	-3.43654400	0.31278200
C	-5.22875300	-2.26722400	0.82559700
H	-5.88164500	-3.52044200	-0.77460100
H	-5.63630000	-4.36200600	0.76780500
O	-3.92189600	-2.39535500	0.55109200
O	-5.67588300	-1.31740000	1.41254700
C	-3.07723900	-1.33896300	0.99619600
C	-1.66755200	-1.70431400	0.58031900
H	-3.39787300	-0.38979600	0.54016100
H	-3.15749700	-1.23357300	2.08894900
S	-0.53292900	-0.39092300	1.13459700
H	-1.60794100	-1.80375700	-0.51228700
H	-1.37191100	-2.65484000	1.04567500
C	0.98126100	-1.10896100	0.61023100
C	2.17504600	-0.48378900	0.52987900
H	0.90490200	-2.16579600	0.33785800
C	3.35971800	-1.22782500	0.01709700
C	2.36268700	0.93882200	0.92893400
C	3.23436600	-2.19563000	-0.99292200
C	4.63668200	-0.98095600	0.54795400
C	1.86993500	1.42395900	2.14856300
C	3.04184300	1.82573900	0.07768900
C	4.34499000	-2.90904700	-1.44013400
H	2.25802500	-2.37754200	-1.44688400
C	5.74687900	-1.69465000	0.09968400
C	2.03542700	2.76397900	2.50140900
H	1.36741500	0.74217100	2.83810500
C	3.20184300	3.16437600	0.42748100
C	5.60600600	-2.66375600	-0.89462800
H	4.22553400	-3.65300900	-2.23005400
H	6.72847400	-1.49306700	0.53276100
C	2.69748400	3.63860800	1.64078500
H	1.64987200	3.12247600	3.45757900
H	3.72319900	3.84233600	-0.25079400
H	6.47601000	-3.21966900	-1.24856700
H	2.82640000	4.68694700	1.91589100
H	3.44148000	1.45959500	-0.87079800
H	4.75812000	-0.22915400	1.33071100

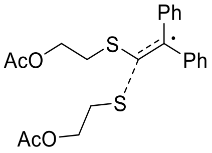

TS3

H	-7.08856100	-3.19906400	0.56324700
C	-6.03944200	-3.33077300	0.28082400
C	-5.21624800	-2.21820800	0.85787500
H	-5.93868600	-3.32991100	-0.81334200
H	-5.66473400	-4.29545800	0.64961000
O	-3.91875200	-2.35301600	0.54369700
O	-5.62883900	-1.30371600	1.52119800
C	-3.04366700	-1.34257400	1.03444000
C	-1.65424900	-1.70922400	0.55453700
H	-3.36151300	-0.36069200	0.64982100
H	-3.09510000	-1.30865400	2.13367600
S	-0.45355400	-0.50392700	1.19974000
H	-1.60080300	-1.68912400	-0.54446400
H	-1.38387200	-2.71305100	0.91100800
C	0.96414200	-1.08792600	0.33708600
C	2.22047200	-0.49604100	0.44115000
H	0.90386500	-2.14895500	0.08567900
C	3.41526400	-1.19680200	-0.06237700
C	2.36999000	0.88678300	0.96072400
C	3.32468400	-2.29883800	-0.93714800
C	4.69829000	-0.78328900	0.34591900

C	1.95552300	1.23554400	2.25356100
C	2.92151000	1.87930000	0.13205300
C	4.46803000	-2.95530000	-1.37969900
H	2.35240200	-2.64154900	-1.29437100
C	5.84091400	-1.44691100	-0.09543600
C	2.08149100	2.54972400	2.70583500
H	1.54914400	0.46986400	2.91737000
C	3.03633500	3.19165900	0.58209000
C	5.73314000	-2.53447800	-0.96174600
H	4.37093700	-3.80055900	-2.06333400
H	6.82241400	-1.11090500	0.24369900
C	2.61591600	3.53014600	1.87087600
H	1.76263700	2.80558300	3.71775200
H	3.45479200	3.95531600	-0.07590600
H	6.62814100	-3.05200300	-1.31115800
H	2.70940000	4.55859800	2.22412300
S	0.62275900	-0.43369200	-1.98861400
C	-0.05649200	1.20683000	-1.64798500
H	0.39211100	1.93094800	-2.34493900
H	0.24081900	1.50524800	-0.62898300
C	-1.56626500	1.22551400	-1.75277300
H	-1.90320500	0.95370800	-2.76544900
H	-2.01486800	0.51605800	-1.04411000
O	-2.00406700	2.54942200	-1.44396300
C	-3.31956900	2.72189700	-1.26860400
C	-3.65076800	4.14992000	-0.94870500
O	-4.11381000	1.82152000	-1.36360600
H	-4.72952100	4.25480000	-0.79629500
H	-3.10737100	4.46018500	-0.04566000
H	-3.32078800	4.79547400	-1.77448200
H	3.24341600	1.61246400	-0.87762400
H	4.80138900	0.05817300	1.03253700

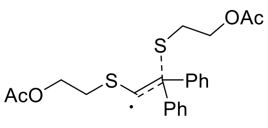

TS3’

H	-8.22118400	-3.41547000	-0.41704900
C	-7.13226500	-3.40539400	-0.52626700
C	-6.60730800	-2.03107100	-0.23678500
H	-6.85111500	-3.69761700	-1.54746100
H	-6.67454300	-4.12622200	0.16510100
O	-5.27086200	-1.98094100	-0.35180500
O	-7.26554300	-1.07042300	0.06305900
C	-4.66452400	-0.71914500	-0.09681900
C	-3.17335900	-0.91407400	-0.28069600
H	-5.05985100	0.03322400	-0.79623700
H	-4.90421000	-0.39059000	0.92603100
S	-2.32820300	0.66675800	0.04030400
H	-2.95414900	-1.23357100	-1.30862100
H	-2.79649800	-1.66848100	0.42319200
C	-0.70976200	0.20053300	-0.32438200
C	0.40311600	1.03630900	-0.14359600
H	-0.58805700	-0.83754000	-0.63940700
C	1.68400700	0.69278800	-0.85251900
C	0.10534300	2.50467600	0.05037500
C	1.81793400	-0.44917100	-1.65272800
C	2.81306000	1.51206100	-0.67441000
C	-0.47876800	3.00451900	1.22130600
C	0.31930600	3.38514500	-1.02073900
C	3.05003200	-0.78586600	-2.22019000
H	0.96341400	-1.09754600	-1.84807700
C	4.03240200	1.19116500	-1.25998700
C	-0.82021800	4.35332900	1.32622700
H	-0.67568200	2.33369200	2.05910400
C	-0.02362300	4.73195900	-0.91606100
C	4.16189300	0.02937000	-2.02741800
H	3.13009100	-1.69003800	-2.82679800
H	4.89427700	1.84185400	-1.10119800
C	-0.59134400	5.22245300	0.26051400
H	-1.26806500	4.72392800	2.24999400

H	0.15343500	5.40052700	-1.76048000
H	5.12329200	-0.23364100	-2.47174100
H	-0.85646700	6.27784800	0.34467400
H	0.75873300	3.01096300	-1.94765000
H	2.73830400	2.39727800	-0.04059700
H	5.82399500	-4.48141600	-0.35978200
C	4.76522100	-4.27114200	-0.17795300
C	4.61735700	-2.92284300	0.46520700
H	4.32308300	-5.04599000	0.46192000
H	4.21748200	-4.27206600	-1.13168800
O	3.35782000	-2.70102900	0.85744500
O	5.50013100	-2.11729800	0.61241300
C	3.07617300	-1.40271800	1.38552400
C	1.57688800	-1.30265200	1.58189900
H	3.61227400	-1.26588600	2.33764600
H	3.43982200	-0.64293400	0.68123300
S	1.04457900	0.37576200	2.00849300
H	1.24382500	-1.98716400	2.37633200
H	1.06403400	-1.61729300	0.65889800

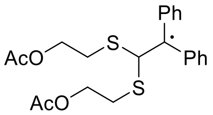

Int3

H	-7.01662400	-3.45643400	0.55334000
C	-5.99096200	-3.52708500	0.17806800
C	-5.14169000	-2.47238800	0.82255000
H	-5.97708500	-3.39071000	-0.91222000
H	-5.57361600	-4.51983200	0.39542000
O	-3.86864100	-2.54477600	0.40695600
O	-5.51794400	-1.64813500	1.61377300
C	-2.97101400	-1.58271800	0.95358400
C	-1.61890500	-1.84196300	0.32171200
H	-3.33391600	-0.56798700	0.72850700
H	-2.93501800	-1.69322500	2.04852400
S	-0.38843100	-0.71039800	1.03876400
H	-1.66612200	-1.66157700	-0.76286800
H	-1.31098200	-2.88258000	0.49810400
C	0.92310400	-0.94637300	-0.20531200
C	2.26100100	-0.45690500	0.25103000
H	0.96006400	-2.02403000	-0.40430100
C	3.45894800	-1.17152700	-0.12522800
C	2.37144600	0.89794800	0.84865100
C	3.44556600	-2.27602100	-1.01533800
C	4.71731300	-0.78099900	0.40298600
C	1.90747400	1.18646600	2.14073200
C	2.93758600	1.93631100	0.08814400
C	4.61769500	-2.94536700	-1.34697800
H	2.51426200	-2.60999400	-1.47341500
C	5.88201800	-1.45972400	0.06983200
C	1.98959800	2.48317900	2.65052400
H	1.49501000	0.38635200	2.75790500
C	3.01419300	3.23066300	0.59687600
C	5.84389600	-2.54698900	-0.80840300
H	4.57393200	-3.78700500	-2.04077800
H	6.83180800	-1.13951600	0.50224300
C	2.53583600	3.50850000	1.87926200
H	1.62851900	2.68954400	3.65968900
H	3.44753500	4.02704000	-0.01099500
H	6.76042700	-3.07755400	-1.07116200
H	2.59584300	4.52247700	2.27868300
S	0.51627600	-0.22906100	-1.87860100
C	-0.14604800	1.41059100	-1.44877700
H	0.28744800	2.13814700	-2.14933800
H	0.17845400	1.67626200	-0.43326600
C	-1.65824400	1.42149700	-1.53091400
H	-2.00853200	1.15695200	-2.54076400
H	-2.09208000	0.70527400	-0.81888200
O	-2.08895100	2.74189300	-1.20452000
C	-3.40948500	2.92323500	-1.06950200
C	-3.73785400	4.34572000	-0.72456800

O	-4.20585300	2.03261100	-1.21580500
H	-4.81958800	4.45609500	-0.59971300
H	-3.21715800	4.63015800	0.20007200
H	-3.38025700	5.00755500	-1.52563600
H	3.30616900	1.71938400	-0.91753100
H	4.76827300	0.05748100	1.09895600

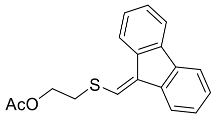

CTA4-P

H	7.95553300	1.00474000	-0.57794500
C	6.96648900	1.36000900	-0.88341700
C	5.91175400	0.70285000	-0.04460100
H	6.79039200	1.12748300	-1.94272100
H	6.90211100	2.45052600	-0.76581800
O	4.68163800	1.11427200	-0.38989800
O	6.10219900	-0.09547800	0.83432100
C	3.60126200	0.55129000	0.34541200
C	2.33276400	1.15980000	-0.21707600
H	3.60474700	-0.54374400	0.23378400
H	3.71735000	0.78307900	1.41515600
S	0.90900700	0.47390400	0.68796400
H	2.23220400	0.91475200	-1.28292400
H	2.34526200	2.25089200	-0.09240100
C	-0.38360700	1.30609000	-0.12201100
C	-1.70197800	1.17822000	0.14701200
H	-0.06469100	1.98788900	-0.91701900
C	-2.77070400	1.90908800	-0.57110500
C	-2.38765600	0.34069700	1.15153000
C	-2.70479800	2.83342900	-1.61407000
C	-4.02077100	1.53419800	-0.03187800
C	-1.89867800	-0.55334600	2.10793200
C	-3.78384500	0.55741300	1.04113900
C	-3.89325900	3.37480300	-2.10835200
H	-1.74552000	3.13314700	-2.04134000
C	-5.20554100	2.07721000	-0.52837900
C	-2.80527500	-1.21770000	2.93926600
H	-0.83194900	-0.74619800	2.22297600
C	-4.68173300	-0.10769300	1.87248300
C	-5.13220200	3.00146500	-1.57195100
H	-3.85568400	4.09900200	-2.92413700
H	-6.17096500	1.78572300	-0.10978000
C	-4.18153800	-0.99906500	2.82476900
H	-2.42998300	-1.91716100	3.68820900
H	-5.75612100	0.06447000	1.78147300
H	-6.04790400	3.43796600	-1.97453100
H	-4.86996400	-1.52942300	3.48506800

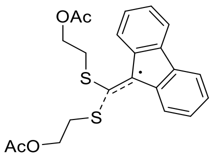

TS4

H	7.43436500	-2.80155500	-0.55558200
C	6.39504400	-3.00518000	-0.27971500
C	5.50298000	-1.94296800	-0.84863500
H	6.28944900	-3.02506900	0.81372800
H	6.08604500	-3.98749500	-0.66281800
O	4.21730600	-2.15727600	-0.52786700
O	5.85460500	-1.00392400	-1.51245200
C	3.28039500	-1.20252400	-1.01441700
C	1.91786900	-1.66365900	-0.53771500
H	3.53176300	-0.20435200	-0.62248900
H	3.33017600	-1.15912100	-2.11331000
S	0.63746600	-0.54829200	-1.18996500
H	1.85685100	-1.63830000	0.56070800
H	1.71605000	-2.68452500	-0.89022100
C	-0.73215900	-1.18671000	-0.31871500
C	-2.00940800	-0.66409300	-0.40513400
H	-0.61902800	-2.22307700	0.01126300
C	-3.19809000	-1.34688700	0.12902400

C	-2.47499500	0.61246700	-0.95889000
C	-3.32590900	-2.57685800	0.77454300
C	-4.33032000	-0.53199800	-0.09263400
C	-1.79854000	1.66496000	-1.58777500
C	-3.88109800	0.69298400	-0.77195300
C	-4.59592400	-2.98399200	1.18985600
H	-2.45628100	-3.21278500	0.95494900
C	-5.59486700	-0.94314700	0.32024600
C	-2.52772000	2.77845200	-2.01436900
H	-0.72059800	1.64122200	-1.75026300
C	-4.59939600	1.80210000	-1.20311700
C	-5.71831100	-2.17799800	0.96396300
H	-4.71488900	-3.94322400	1.69651700
H	-6.47146500	-0.31556300	0.14780700
C	-3.91112100	2.84791800	-1.82708900
H	-2.00625100	3.60284100	-2.50315100
H	-5.67983700	1.85685000	-1.05645100
H	-6.70089400	-2.51779600	1.29586100
H	-4.45941400	3.72642100	-2.17144600
S	-0.43746600	-0.50025300	2.07649900
C	0.07234400	1.19219900	1.71039000
H	-0.39231200	1.87345600	2.43880900
H	-0.32540400	1.46209900	0.71719800
C	1.57852000	1.34566700	1.71281200
H	2.00192700	1.12844400	2.70601700
H	2.04169900	0.66131500	0.98908100
O	1.87283000	2.69520100	1.35209700
C	3.15227300	2.97996800	1.08022300
C	3.32967400	4.42276900	0.70952300
O	4.02903100	2.15612600	1.13418800
H	4.38501100	4.62402600	0.50066800
H	2.71810800	4.65145300	-0.17427100
H	2.97730600	5.05961000	1.53260200

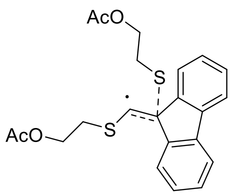

TS4'

H	8.89356500	-2.34896000	0.60022000
C	7.81369300	-2.50022000	0.69381900
C	7.09119900	-1.25338000	0.27997800
H	7.55294600	-2.75004300	1.73135600
H	7.49256000	-3.33695200	0.05785400
O	5.76148400	-1.38983600	0.40694700
O	7.59977200	-0.24070400	-0.12203200
C	4.97617800	-0.26409800	0.03315300
C	3.53091000	-0.65392500	0.26840100
H	5.25941900	0.60859800	0.64099400
H	5.15981700	-0.01591700	-1.02346000
S	2.46031000	0.74109300	-0.20300300
H	3.36313300	-0.88201500	1.32953400
H	3.26376400	-1.52793300	-0.34092300
C	0.93740300	0.13138900	0.30163600
C	-0.28872800	0.78731900	0.10615200
H	0.93918000	-0.86918000	0.74407700
C	-1.47833000	0.35195400	0.89818400
C	-0.47890400	2.25999400	-0.08877200
C	-1.78709200	-0.88950700	1.45006300
C	-2.34686100	1.44846000	1.06859700
C	0.31629800	3.20393500	-0.73487000
C	-1.71933700	2.63604800	0.47114200
C	-3.00849900	-1.04158100	2.11237600
H	-1.10288100	-1.73607400	1.36362500
C	-3.56582200	1.29184100	1.72691100
C	-0.12873500	4.52892500	-0.79422300
H	1.26052500	2.93126700	-1.20818600
C	-2.16055300	3.95579000	0.40381400
C	-3.89565700	0.03330800	2.23591400

H	-3.27320700	-2.01164000	2.53769000
H	-4.24596200	2.13708500	1.84971200
C	-1.35190600	4.90213500	-0.23016900
H	0.48735100	5.27854400	-1.29370700
H	-3.11908700	4.24454500	0.83955600
H	-4.84855800	-0.10986900	2.74826300
H	-1.67960300	5.94140900	-0.28969000
H	-5.65403700	-4.44722700	0.56467000
C	-4.63756600	-4.33810700	0.17351100
C	-4.47320000	-2.97960100	-0.44380800
H	-4.43757800	-5.11154100	-0.58090900
H	-3.90421900	-4.46497700	0.98204000
O	-3.24242800	-2.81427800	-0.94281700
O	-5.32248300	-2.12849600	-0.50157000
C	-2.94071700	-1.52909200	-1.48790500
C	-1.44372000	-1.46719400	-1.72620000
H	-3.49828800	-1.38350500	-2.42663900
H	-3.26295800	-0.75158200	-0.78154100
S	-0.88017500	0.22535500	-2.02407000
H	-1.15582600	-2.08893300	-2.58745800
H	-0.90331200	-1.86808900	-0.85434900

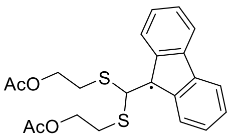

Int4

H	7.41665900	-2.88510200	-0.75923000
C	6.41209400	-3.07836300	-0.37015100
C	5.44437300	-2.09392500	-0.95586500
H	6.40596200	-2.99258000	0.72511100
H	6.09628100	-4.09905700	-0.62669000
O	4.20095200	-2.29140300	-0.49311500
O	5.71050100	-1.22493800	-1.74387100
C	3.19731200	-1.41071800	-0.98995500
C	1.89578100	-1.81653300	-0.32837800
H	3.46405200	-0.37033400	-0.74752400
H	3.14092000	-1.49879100	-2.08598400
S	0.54403300	-0.80090800	-0.99380600
H	1.95383800	-1.64671200	0.75711900
H	1.68766100	-2.87986400	-0.51480900
C	-0.69754600	-1.08462200	0.31044600
C	-2.03813600	-0.56886900	-0.08133500
H	-0.76759600	-2.17263300	0.45988300
C	-3.26375400	-1.22001600	0.32750400
C	-2.40372300	0.60939100	-0.83882800
C	-3.47809300	-2.37595200	1.09076000
C	-4.36859800	-0.48042000	-0.17559900
C	-1.64372300	1.61570000	-1.46342900
C	-3.82982000	0.66831800	-0.91187200
C	-4.79035300	-2.78239100	1.33408800
H	-2.64140600	-2.95097400	1.49344400
C	-5.67204100	-0.89431800	0.07011400
C	-2.30189900	2.64152200	-2.14128000
H	-0.55390900	1.61200500	-1.42153000
C	-4.47312500	1.69411800	-1.59125800
C	-5.87539600	-2.05307400	0.82969000
H	-4.97471700	-3.68013800	1.92646500
H	-6.52195600	-0.32907500	-0.31758400
C	-3.69979200	2.68373700	-2.20941400
H	-1.71466200	3.42362100	-2.62514700
H	-5.56325300	1.72972500	-1.64127300
H	-6.89260200	-2.39191100	1.03330500
H	-4.19073300	3.49685500	-2.74659700
S	-0.24042400	-0.50690400	2.00874900
C	0.16227100	1.24238700	1.73590900
H	-0.19139200	1.79210600	2.61946600
H	-0.40003200	1.61291900	0.86757300
C	1.65145300	1.44740200	1.54433600
H	2.21480800	1.11420800	2.42966400
H	2.01551900	0.88685100	0.67276900

O	1.86218500	2.84300300	1.33744000
C	3.10686900	3.21429000	1.00763000
C	3.20564000	4.69802000	0.81163200
O	4.00998200	2.42715100	0.89142500
H	4.23175400	4.96833200	0.54345100
H	2.51163000	5.00999700	0.01902500
H	2.90769700	5.21093400	1.73650200

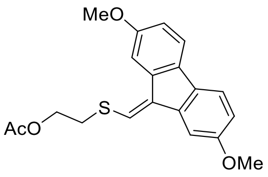

CTA5-P

H	7.46587800	-3.72099200	-1.43701000
C	6.40022100	-3.83391800	-1.21470800
C	5.71675500	-2.50387700	-1.32431400
H	6.26521000	-4.23138700	-0.19917300
H	5.93866900	-4.54356100	-1.91496200
O	4.40133600	-2.60537700	-1.07770500
O	6.24388700	-1.45730000	-1.59434700
C	3.65239300	-1.39755300	-1.15041100
C	2.21422200	-1.76378100	-0.84607600
H	4.04478900	-0.67137200	-0.42217700
H	3.74992100	-0.95715700	-2.15439500
S	1.19914800	-0.25316100	-0.91640300
H	2.13677500	-2.20273500	0.15773200
H	1.83917700	-2.48146900	-1.58807500
C	-0.36447400	-0.94084500	-0.59571100
C	-1.52806900	-0.26063000	-0.50770700
H	-0.36953600	-2.02723000	-0.45988500
C	-2.84112200	-0.89202300	-0.23654100
C	-1.79357000	1.18699600	-0.64429800
C	-3.17343200	-2.22294900	-0.02684100
C	-3.83415500	0.11535300	-0.21072700
C	-0.94106200	2.25485100	-0.90038600
C	-3.18428100	1.40851200	-0.46366400
C	-4.51623300	-2.56023100	0.21313200
H	-2.42947600	-3.02181800	-0.04133200
C	-5.16097600	-0.22501300	0.02736700
C	-1.47341600	3.55504900	-0.97721100
H	0.13276900	2.14153200	-1.04712100
C	-3.70084400	2.69494500	-0.54161100
C	-5.50588600	-1.56385700	0.23998200
O	-4.76864800	-3.87191800	0.40838400
H	-5.93866900	0.54124800	0.05032200
C	-2.84685000	3.77381600	-0.79875700
O	-0.58284400	4.53745300	-1.22813100
H	-4.76895600	2.87560200	-0.40408100
H	-6.54739900	-1.82222100	0.42565700
C	-6.09811300	-4.27068600	0.65313300
H	-3.26175700	4.77906900	-0.85794700
C	-1.05400400	5.86294300	-1.31654000
H	-6.75770600	-4.01953800	-0.19317500
H	-6.08045900	-5.35909400	0.78145100
H	-6.49675600	-3.80740900	1.57009600
H	-0.18122300	6.49320800	-1.52225000
H	-1.78238000	5.97870300	-2.13533600
H	-1.51929600	6.18973700	-0.37256900

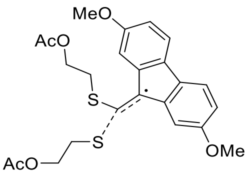

TS5

H	7.44466900	-3.68522400	-1.29689200
C	6.38191700	-3.79100300	-1.05782200
C	5.67750200	-2.48889900	-1.29592900
H	6.25838600	-4.08596400	-0.00649700
H	5.92794300	-4.57459800	-1.67973100
O	4.36380500	-2.58695000	-1.03941700

O	6.18833200	-1.46574200	-1.66782300
C	3.59589100	-1.40254900	-1.22581800
C	2.15828900	-1.77859600	-0.92687500
H	3.96192800	-0.61487200	-0.54803300
H	3.71220200	-1.04335100	-2.25973800
S	1.07926100	-0.34644200	-1.23225400
H	2.03860300	-2.06919400	0.12775400
H	1.84050200	-2.60980000	-1.57138900
C	-0.40998600	-1.00174300	-0.59970100
C	-1.59346800	-0.28777400	-0.53739700
H	-0.46173500	-2.09413600	-0.60701300
C	-2.90097600	-0.90271600	-0.24943600
C	-1.83260900	1.15496400	-0.67720200
C	-3.23812300	-2.22966500	-0.01995700
C	-3.88478000	0.11449100	-0.21805600
C	-0.96754300	2.21337000	-0.94728100
C	-3.22101200	1.39792400	-0.48485000
C	-4.58104100	-2.55295100	0.24195400
H	-2.50162300	-3.03550100	-0.03454300
C	-5.20920700	-0.21209100	0.03937500
C	-1.48356400	3.52046100	-1.02023000
H	0.10373000	2.08679500	-1.10349500
C	-3.71970000	2.68889500	-0.56430700
C	-5.56093500	-1.54782300	0.27012100
O	-4.84044200	-3.85948900	0.45469800
H	-5.98112200	0.55972100	0.06654000
C	-2.85228900	3.75620000	-0.83220600
O	-0.58282400	4.48980000	-1.27981500
H	-4.78393400	2.88479300	-0.41897100
H	-6.60247400	-1.79440700	0.47156700
C	-6.16897700	-4.24543400	0.72570000
H	-3.25444700	4.76654700	-0.89245800
C	-1.03759800	5.82151100	-1.37024200
H	-6.83946500	-4.00159100	-0.11411600
H	-6.15653400	-5.33181500	0.86979200
H	-6.54979400	-3.76530900	1.64153000
H	-0.15775400	6.43877700	-1.58438500
H	-1.76961000	5.94295000	-2.18486800
H	-1.49177400	6.15761000	-0.42418000
H	2.52298200	-0.08218900	1.19503500
C	2.13872700	0.40720600	2.09918400
C	0.62864300	0.50514200	2.05606400
H	2.47693700	-0.17386600	2.97156000
O	2.65792200	1.73452800	2.18190300
S	-0.14693600	-1.11567200	1.88012400
H	0.23945500	0.99155300	2.96303100
H	0.32179300	1.12836500	1.19874400
C	3.96267200	1.88917100	1.92610200
C	4.38366100	3.32312200	2.05529100
O	4.68407800	0.97012100	1.63303200
H	5.44842100	3.41894100	1.82074600
H	3.78626200	3.94494600	1.37458300
H	4.18932600	3.66921200	3.07997500

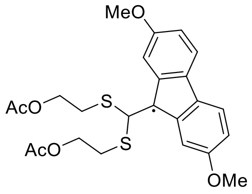

Int5

H	7.38266200	-3.82255300	-1.36102900
C	6.33134700	-3.96178400	-1.09046800
C	5.56762600	-2.69981700	-1.36106400
H	6.24511800	-4.22168900	-0.02639800
H	5.89587100	-4.78628700	-1.67164300
O	4.27110100	-2.83174200	-1.04419600
O	6.02431000	-1.67936900	-1.80507300
C	3.45023900	-1.68699800	-1.25908300
C	2.05405500	-2.06128900	-0.80346000
H	3.84522600	-0.83481000	-0.68466600
H	3.47084000	-1.41501700	-2.32586200

S	0.92246800	-0.68045700	-1.14177400
H	2.04986100	-2.25300500	0.27996200
H	1.70883500	-2.96341800	-1.32849500
C	-0.42738900	-1.11832200	0.00500600
C	-1.62619600	-0.26467800	-0.21478800
H	-0.69860400	-2.16541700	-0.19801000
C	-2.97558500	-0.77223100	-0.07057200
C	-1.72488800	1.14471700	-0.54171300
C	-3.42948800	-2.05022800	0.25388400
C	-3.89446700	0.28614100	-0.31741600
C	-0.75247200	2.12376500	-0.77586600
C	-3.11081200	1.48962400	-0.61656200
C	-4.81380700	-2.27944100	0.32708800
H	-2.75160900	-2.88170500	0.45622300
C	-5.25710200	0.04725400	-0.24406400
C	-1.15409000	3.43391300	-1.08499400
H	0.31687000	1.91794600	-0.72003500
C	-3.49149900	2.78459700	-0.92465200
C	-5.72129800	-1.23704000	0.07865700
O	-5.18662900	-3.53678300	0.64441700
H	-5.97737200	0.84612500	-0.43206900
C	-2.51552100	3.76396900	-1.16181200
O	-0.15531500	4.31734400	-1.29293700
H	-4.54786400	3.05476800	-0.98364400
H	-6.79450400	-1.41368200	0.13481200
C	-6.56324300	-3.82584000	0.73612100
H	-2.82843800	4.77896200	-1.40270800
C	-0.49288800	5.64960300	-1.60594300
H	-7.07658500	-3.65491300	-0.22387700
H	-6.64371000	-4.88632600	1.00095000
H	-7.05139800	-3.22189000	1.51801400
H	0.45172200	6.18994400	-1.73590300
H	-1.07325200	5.70999000	-2.54070400
H	-1.06865800	6.12188000	-0.79361200
H	2.49433700	0.18440800	0.94525400
C	2.20352600	0.47687800	1.96254000
C	0.69633600	0.45598400	2.11969200
H	2.68588600	-0.21178000	2.67359300
O	2.63847300	1.81170600	2.21647400
S	0.01245500	-1.19585800	1.80136600
H	0.41126300	0.72031400	3.14786500
H	0.21972900	1.18253600	1.44716400
C	3.92149800	2.08188600	1.94025000
C	4.25902600	3.51322200	2.23559500
O	4.68180800	1.25443000	1.50855800
H	5.31136000	3.70134000	2.00088900
H	3.61336600	4.17205400	1.63860100
H	4.06287600	3.72527300	3.29566500

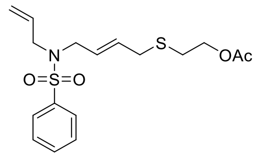

M

C	0.07841200	3.70615000	-2.78683400
C	0.14691900	3.12145100	-1.59145000
H	0.89146600	3.60666500	-3.51351900
H	-0.78528800	4.30867500	-3.07902300
C	1.31465200	2.29453600	-1.13330000
H	-0.67127300	3.19979500	-0.86778900
N	0.88721700	0.94108100	-0.78873600
H	2.09199100	2.25711400	-1.91594400
H	1.75252100	2.74394700	-0.23237300
S	1.24327100	0.27840800	0.66573300
C	0.36251400	0.07516800	-1.84286800
O	1.58186100	1.36325100	1.57771400
O	0.18665500	-0.66629800	1.01340200
C	2.72364100	-0.68175700	0.40343800
C	-1.13384800	-0.05100900	-1.81865800
H	0.83509200	-0.92046700	-1.79862900
H	0.68524900	0.52932700	-2.79403100

C	2.63183100	-2.05905200	0.21206500
C	3.94860700	-0.01285500	0.36173800
C	-1.79001000	-1.19545400	-2.02286200
H	-1.68881600	0.87554700	-1.62991800
C	3.79954400	-2.78190100	-0.03610700
C	5.10574400	-0.74683000	0.11333400
H	3.99454000	1.06442300	0.53218500
C	-3.27801300	-1.30853300	-1.99901500
H	-1.22887200	-2.12667400	-2.17139700
C	5.03035600	-2.12771900	-0.08677900
H	3.74510800	-3.86118100	-0.18626200
H	6.07137300	-0.24006900	0.08112700
H	-3.65860100	-1.82396900	-2.89362200
H	-3.75073800	-0.31714300	-1.95352900
S	-3.86502600	-2.35347900	-0.60898400
H	5.94066600	-2.69784100	-0.27979000
C	-2.99830000	-1.57038700	0.77901600
H	-3.18792500	-2.21629700	1.64880900
H	-1.91567300	-1.55300800	0.58993200
C	-3.48624900	-0.16067300	1.05420400
O	-2.74499300	0.37396900	2.15007800
H	-4.54605500	-0.16458100	1.34515300
H	-3.35699600	0.48557500	0.17653000
C	-1.85576700	1.35039300	1.91974000
C	-1.07664200	1.66341700	3.16217800
O	-1.72772300	1.90123400	0.85592600
H	-0.48407800	2.57022900	3.01178700
H	-1.75998200	1.77185800	4.01474100
H	-0.40062700	0.82073800	3.36220500
H	1.66185700	-2.55497900	0.26730300

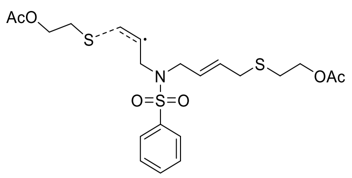

TS6

C	-1.58356700	-2.73985400	-2.32987500
C	-0.90822400	-2.01120800	-1.38888100
H	-2.28269000	-2.24152100	-3.00673200
H	-1.18283400	-3.70098700	-2.65690300
C	-1.24756000	-0.59348700	-1.04225300
H	-0.12873100	-2.47041100	-0.77494500
N	-0.05239300	0.21402300	-0.83586700
H	-1.89043300	-0.14489800	-1.81997600
H	-1.80424400	-0.57804200	-0.09237100
S	0.12611800	1.13602800	0.50689800
C	0.91671800	0.37242600	-1.92049200
O	-0.92679500	0.74816300	1.44221300
O	1.51914700	1.09839500	0.93871000
C	-0.20209400	2.80858900	-0.01800100
C	2.10807300	-0.53299100	-1.79045700
H	1.24008900	1.42435400	-1.99257500
H	0.36928500	0.14945600	-2.85092400
C	0.83237800	3.74023000	-0.04139300
C	-1.50562900	3.14044300	-0.39378000
C	3.37220100	-0.13718300	-1.95521500
H	1.89409600	-1.58233500	-1.55664900
C	0.55179300	5.04375200	-0.45465300
C	-1.76964500	4.44476400	-0.80368700
H	-2.30643400	2.39647000	-0.37223700
C	4.54205000	-1.05500600	-1.82338500
H	3.59246800	0.91848400	-2.15756000
C	-0.74369500	5.39366000	-0.83330000
H	1.35070100	5.78642700	-0.47827100
H	-2.78255900	4.72199700	-1.09979400
H	5.19593000	-1.00348300	-2.70706700
H	4.21406400	-2.09765400	-1.70621000
S	5.64425800	-0.57811100	-0.43611000
H	-0.95835000	6.41426300	-1.15462500
C	4.43686700	-0.41130100	0.90774600

H	5.00246400	-0.01351800	1.76310200
H	3.66286100	0.31931600	0.63516700
C	3.78031900	-1.72734900	1.27935300
O	2.86615800	-1.49116100	2.35020500
H	4.52777700	-2.44961100	1.63714400
H	3.24078300	-2.16050000	0.42731200
C	1.55109300	-1.60663800	2.12629300
C	0.76377800	-1.15640000	3.32037900
O	1.07980100	-2.02890600	1.10035400
H	-0.28071100	-1.46569700	3.21719600
H	1.20690200	-1.56037100	4.23966100
H	0.81197800	-0.05931400	3.36188700
H	1.83766900	3.44730600	0.26323900
S	-3.23444500	-3.98893900	-1.09072200
C	-3.46143600	-2.84213900	0.28739900
C	-4.24810600	-1.61376300	-0.12478200
H	-2.48214300	-2.55764800	0.70247300
H	-3.82166700	-1.15146000	-1.02398300
H	-5.29432700	-1.87607200	-0.34354600
H	-3.98944600	-3.39759900	1.07838700
O	-4.22058900	-0.67757400	0.95715400
C	-4.16653700	0.62733000	0.66837000
O	-4.16542300	1.05170900	-0.46225200
C	-4.06902900	1.47241900	1.90220100
H	-4.63457000	1.02787000	2.72964400
H	-3.00631300	1.51579800	2.18510900
H	-4.42664500	2.48479300	1.68345900

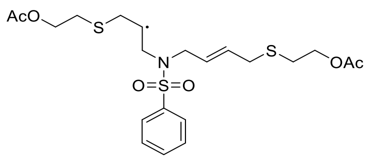

Int6

C	0.36865600	2.05685700	0.35117500
C	-0.04273900	0.97511900	-0.56483800
H	-0.04316500	1.90854900	1.36002700
H	1.46678600	2.11451000	0.42942600
C	-1.22741600	0.10813400	-0.29272000
H	0.41951400	0.90267600	-1.55265100
N	-0.99458100	-1.28139900	-0.70942400
H	-1.49803100	0.14489300	0.78023600
H	-2.10092700	0.47703100	-0.85950300
S	-2.29355800	-2.04200700	-1.41963900
C	-0.21188700	-2.11913400	0.22069500
O	-2.80924700	-1.14437400	-2.44239200
O	-1.85136200	-3.38234800	-1.77414200
C	-3.52890700	-2.18391100	-0.14379100
C	1.02422100	-1.40599000	0.69048000
H	0.06436100	-3.03589000	-0.31287500
H	-0.82989000	-2.39128900	1.09806600
C	-3.55141300	-3.32796700	0.65432000
C	-4.40888100	-1.12076700	0.06956400
C	2.24307800	-1.62764900	0.19164400
H	0.90178300	-0.66191600	1.48599600
C	-4.47373500	-3.40012800	1.69775100
C	-5.32223000	-1.20463100	1.11892200
H	-4.38685900	-0.25045700	-0.58999700
C	3.44730300	-0.85433100	0.62115400
H	2.37810100	-2.35738000	-0.61517300
C	-5.35154400	-2.34048600	1.93099200
H	-4.50745600	-4.28962700	2.32853100
H	-6.01310700	-0.38067400	1.30347700
H	4.33173800	-1.50205100	0.70402600
H	3.27604600	-0.35941200	1.58762400
S	3.80135400	0.42798400	-0.64526900
H	-6.06974200	-2.40178800	2.75032500
C	5.52391500	0.80749700	-0.20971500
H	5.72765100	1.82684700	-0.56684300
H	5.61938700	0.80336800	0.88554400
C	6.49202000	-0.17678100	-0.84527800
O	7.83719500	0.11044900	-0.46171900

H	6.46666300	-0.08538600	-1.93788200
H	6.24278100	-1.20987400	-0.56573900
C	8.23648200	-0.33528400	0.73869000
C	9.65807700	0.05111700	1.01987500
O	7.52180100	-0.96446300	1.47547900
H	9.96709000	-0.35324800	1.98882700
H	10.30898800	-0.33086200	0.22163800
H	9.74300300	1.14680500	1.02311500
H	-2.86418600	-4.15051100	0.45067500
S	-0.08313600	3.75807600	-0.24249100
C	-1.79846100	3.50110700	-0.77351900
C	-2.72521700	3.15559900	0.37220200
H	-1.83569400	2.72308200	-1.55060300
H	-2.39460100	2.25731200	0.91436500
H	-2.77908900	3.98119600	1.09829700
H	-2.10723500	4.44763800	-1.24050300
O	-4.01863700	2.91658200	-0.18509300
C	-4.95268200	2.43691700	0.64691300
O	-4.73448200	2.21444600	1.81007500
C	-6.25845500	2.21156700	-0.05847600
H	-6.50422800	3.07766200	-0.68643800
H	-6.16516500	1.33588000	-0.71833200
H	-7.05024900	2.03438600	0.67678300

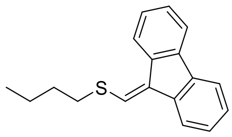

A

C	3.58828600	-1.63442000	-1.22810700
C	2.17356700	-1.97196800	-0.78112800
H	3.95250700	-0.75744700	-0.66714700
H	3.57992800	-1.34977300	-2.29362000
S	1.08332900	-0.53367400	-1.05467900
H	2.14906900	-2.22341900	0.28872400
H	1.77285800	-2.82083300	-1.35321300
C	-0.42534200	-1.16868400	-0.47897600
C	-1.61031500	-0.51684800	-0.44463500
H	-0.37816300	-2.20071400	-0.11507000
C	-2.87022400	-1.10823500	0.05803600
C	-1.94741200	0.85677800	-0.86640100
C	-3.14266100	-2.37347600	0.57984200
C	-3.90147300	-0.14965300	-0.05443300
C	-1.16848400	1.87932400	-1.41563800
C	-3.32796100	1.07509900	-0.63024600
C	-4.44617700	-2.66761700	0.98457300
H	-2.35626100	-3.12545600	0.67326700
C	-5.20241400	-0.44765100	0.35110400
C	-1.77389100	3.10174200	-1.72119100
H	-0.10413200	1.74730700	-1.61081700
C	-3.92484400	2.29570400	-0.93710900
C	-5.46742100	-1.71523500	0.87205100
H	-4.67217300	-3.65374300	1.39439800
H	-5.99774300	0.29532600	0.26301100
C	-3.13654800	3.31013300	-1.48556200
H	-1.17137300	3.90427700	-2.15022400
H	-4.98897100	2.45626100	-0.75230100
H	-6.47883600	-1.96796900	1.19498500
H	-3.58694500	4.27317600	-1.73236300
C	4.54465500	-2.80594300	-1.02050400
H	4.16942400	-3.68076100	-1.57667800
H	4.54121900	-3.08989300	0.04476200
C	5.96525900	-2.48201900	-1.46429000
H	6.63945600	-3.33628000	-1.30740800
H	5.99485300	-2.22168000	-2.53349300
H	6.36906500	-1.62658700	-0.90123500

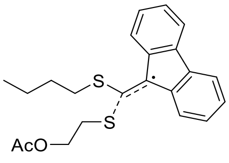

A-TS1

C	3.43308000	-1.67914200	-1.33619400
C	2.02930300	-2.02102800	-0.85821200
H	3.74479000	-0.71018600	-0.91002300
H	3.43393800	-1.56192700	-2.43284400
S	0.86384700	-0.70673300	-1.34516600
H	1.98568200	-2.09757100	0.23964600
H	1.68152500	-2.97041500	-1.29077900
C	-0.54473800	-1.22624100	-0.46280800
C	-1.74371100	-0.53325600	-0.44339500
H	-0.56243500	-2.29176200	-0.21722800
C	-2.99381600	-1.09316000	0.09164900
C	-2.05061300	0.83470300	-0.87471000
C	-3.26492800	-2.34297900	0.64933300
C	-4.01292200	-0.12046000	-0.01618700
C	-1.25959100	1.83676500	-1.45037500
C	-3.42582500	1.08541800	-0.61958600
C	-4.56322500	-2.61086800	1.08926400
H	-2.48330400	-3.10004200	0.74412800
C	-5.30659800	-0.39308400	0.42070000
C	-1.84526500	3.06803700	-1.75768000
H	-0.20125200	1.68206400	-1.66165200
C	-4.00150300	2.31138200	-0.93275100
C	-5.57393400	-1.64846700	0.97450300
H	-4.79280900	-3.58333300	1.52820300
H	-6.09560600	0.35680900	0.33567700
C	-3.19963300	3.30443700	-1.50493000
H	-1.23365100	3.85353300	-2.20436600
H	-5.05913100	2.49644400	-0.73501400
H	-6.58151600	-1.88123100	1.32333100
H	-3.63548300	4.27271800	-1.75688500
S	-0.08783300	-0.78021900	1.96086700
C	0.70599500	0.81998900	1.69806000
H	0.38958600	1.51378900	2.49119300
H	0.34007000	1.23192800	0.74200100
C	2.21447500	0.69891800	1.65422800
H	2.61416300	0.33588700	2.61421600
H	2.52675300	-0.00308700	0.86976900
O	2.74753600	1.99388500	1.37523900
C	4.04632900	2.05226800	1.05743500
C	4.48455600	3.46051700	0.78272800
O	4.74988500	1.07564900	1.00674500
H	5.54904300	3.47423100	0.52856200
H	3.89136300	3.87412800	-0.04452200
H	4.29827100	4.08203400	1.66948500
C	4.44357000	-2.75070300	-0.93470400
H	4.13160200	-3.72050000	-1.35615900
H	4.42479900	-2.86675400	0.16166600
C	5.85677500	-2.41558400	-1.39359700
H	6.57138200	-3.19649400	-1.09617600
H	5.90311900	-2.31605600	-2.48896600
H	6.19683400	-1.46401500	-0.95697100

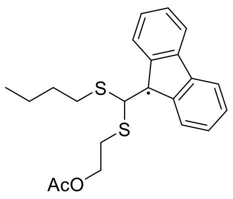

A-Int1

C	3.28689600	-2.12571700	-1.31912200
C	1.99442100	-2.25269400	-0.52380500
H	3.70453700	-1.11387200	-1.18095300
H	3.07543400	-2.23740500	-2.39609300
S	0.77580100	-1.03235900	-1.10313000
H	2.17648900	-2.06627000	0.54600600
H	1.56465800	-3.26061000	-0.62986100
C	-0.45640100	-1.18071800	0.22853900
C	-1.72361000	-0.46951000	-0.09451500
H	-0.67589600	-2.25186600	0.35271100
C	-3.01462100	-0.95030500	0.34830800
C	-1.94426500	0.77015400	-0.80874500
C	-3.36516900	-2.08508800	1.09278200

C	-4.01960300	-0.04635600	-0.09235800
C	-1.06999200	1.67414700	-1.44037100
C	-3.34866100	1.03467900	-0.82293000
C	-4.71244100	-2.30646100	1.37978000
H	-2.60700000	-2.78618000	1.44821300
C	-5.35924100	-0.27696400	0.19619600
C	-1.59734700	2.80255400	-2.06761200
H	0.00829500	1.51044800	-1.44197200
C	-3.86139000	2.16124200	-1.45253900
C	-5.69928600	-1.41577000	0.93652300
H	-5.00176700	-3.18535000	1.95835800
H	-6.13272100	0.41453100	-0.14397800
C	-2.97619600	3.04674600	-2.07850000
H	-0.92136300	3.50578900	-2.55674000
H	-4.93601500	2.35457100	-1.45833300
H	-6.74647400	-1.61164400	1.17320500
H	-3.36388800	3.93658100	-2.57720500
S	0.13100900	-0.72558400	1.92757100
C	0.78150400	0.95162400	1.68038600
H	0.53649300	1.52539100	2.58522900
H	0.25769300	1.42279600	0.83693400
C	2.27935500	0.94272200	1.45045300
H	2.80923200	0.49860500	2.30724000
H	2.53623300	0.36838100	0.54953900
O	2.68727400	2.30011100	1.28508400
C	3.97034900	2.50003900	0.95480500
C	4.27872700	3.95999800	0.80144200
O	4.75131500	1.59614100	0.80729900
H	5.33237100	4.08990900	0.53532900
H	3.63495300	4.39057500	0.02206800
H	4.05790500	4.48272300	1.74228300
C	4.32130900	-3.16500000	-0.89426700
H	3.90020600	-4.17373500	-1.03810300
H	4.51301900	-3.05968400	0.18648700
C	5.62835400	-3.03569100	-1.66548300
H	6.35989500	-3.79227100	-1.34683200
H	5.46289800	-3.16204500	-2.74653900
H	6.08097300	-2.04408900	-1.51091200

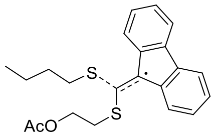

A-TS2

H	-7.78636400	-1.51416200	-0.28423100
C	-6.78183500	-1.94691100	-0.24624000
C	-5.78377200	-0.95497800	-0.76355700
H	-6.52524400	-2.22257400	0.78578900
H	-6.73992400	-2.86003500	-0.85605500
O	-4.53415700	-1.44228200	-0.71281600
O	-6.03139700	0.14627100	-1.17934100
C	-3.50241400	-0.57878300	-1.17577400
C	-2.18791200	-1.24796900	-0.82983900
H	-3.59113700	0.40324300	-0.68671500
H	-3.60569700	-0.42383300	-2.26113500
S	-0.82523200	-0.13433900	-1.28524000
H	-2.11872100	-1.42658600	0.25377500
H	-2.07895100	-2.20123700	-1.36516900
C	0.48920800	-0.97778800	-0.50760400
C	1.78767400	-0.50214400	-0.47815300
H	0.32431300	-2.04969800	-0.37030400
C	2.93738600	-1.31449000	-0.05145200
C	2.31335300	0.83216600	-0.79014800
C	3.00640200	-2.64256800	0.37058700
C	4.10374600	-0.51902800	-0.10578000
C	1.69286300	2.00508900	-1.23795800
C	3.71657000	0.82229500	-0.56727900
C	4.25067200	-3.16592000	0.72921300
H	2.11143700	-3.26635700	0.42193800
C	5.34260400	-1.04573700	0.25064300
C	2.47300800	3.14491500	-1.45068700

H	0.61944100	2.05318000	-1.42227200
C	4.48611900	1.95951800	-0.78571300
C	5.40666800	-2.37786200	0.66871200
H	4.32273200	-4.20310700	1.06104700
H	6.24460800	-0.43208400	0.20770000
C	3.85329700	3.12485000	-1.22981900
H	1.99430500	4.06203100	-1.79759300
H	5.56402300	1.94326500	-0.61299100
H	6.36809400	-2.80927200	0.95266000
H	4.44207100	4.02654200	-1.40668500
S	0.15922800	-0.70246300	1.95485700
C	-0.35334200	1.03163900	1.87767800
H	0.16260000	1.57969500	2.68173600
H	0.03803700	1.43593200	0.92760700
C	-1.86050300	1.23990200	1.95605100
H	-2.22585200	0.89358500	2.93775400
H	-2.35230400	0.60666900	1.19994000
C	-2.26314300	2.69481800	1.73518400
H	-1.74290600	3.33396100	2.46823500
H	-1.90859600	3.01380100	0.73974100
C	-3.76817000	2.90802000	1.83956000
H	-4.13802900	2.62625900	2.83753200
H	-4.04093300	3.95893900	1.66484300
H	-4.30740200	2.29455800	1.10048500

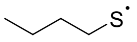

A-Int2

C	0.55250800	-0.29670600	0.35326600
H	0.28498100	-1.31508500	0.01925400
H	0.48743200	-0.32295800	1.45481000
S	2.28744000	-0.10548300	-0.08841100
C	-0.38259300	0.75305800	-0.23478900
C	-2.47331500	-0.72420500	-0.18746800
H	-2.41430300	-0.86273500	-1.27868300
H	-1.98282900	-1.58770700	0.28659000
H	-3.53526600	-0.75430200	0.09684800
H	-0.33311700	0.69728500	-1.33505500
H	-0.01303200	1.75108500	0.04845400
C	-1.83058900	0.59400100	0.23053700
H	-2.41937000	1.43157800	-0.17540800
H	-1.86960100	0.69369000	1.32849600

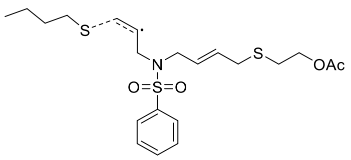

A-TS3

C	-1.79096600	-2.85929800	-2.04208700
C	-1.11375000	-2.04898600	-1.17275900
H	-2.44187000	-2.41890900	-2.80194700
H	-1.42509100	-3.86943700	-2.23489500
C	-1.40695500	-0.58940300	-1.00489600
H	-0.36464500	-2.45419700	-0.48491500
N	-0.19348900	0.18063600	-0.77055200
H	-1.94381800	-0.19191400	-1.88348300
H	-2.05963200	-0.44970700	-0.12967500
S	-0.02121700	1.04719200	0.61061700
C	0.73019600	0.42008500	-1.87882100
O	-0.91027800	0.46605500	1.60947000
O	1.40409000	1.18446100	0.88742600
C	-0.64156500	2.67544200	0.23466700
C	1.98146000	-0.40646400	-1.80579700
H	0.97973000	1.49218100	-1.95198400
H	0.16891500	0.16869200	-2.79328400
C	0.24675600	3.70048900	-0.08380800
C	-2.02422800	2.87141300	0.23297500
C	3.19854100	0.06194200	-2.09054800
H	1.84733800	-1.45428000	-1.51171400
C	-0.26795400	4.95334000	-0.42058400
C	-2.52381400	4.12601600	-0.10692800
H	-2.69809300	2.05541200	0.50197600

C	4.44003600	-0.76325000	-2.02215500
H	3.32742800	1.12131300	-2.34578100
C	-1.64676900	5.16370900	-0.43401700
H	0.41362700	5.76815400	-0.66916000
H	-3.60173500	4.29615100	-0.11012900
H	5.02846100	-0.68542100	-2.94880800
H	4.20401800	-1.82450500	-1.85970500
S	5.58845600	-0.16315000	-0.72247600
H	-2.04327100	6.14586000	-0.69681600
C	4.46805100	-0.10959900	0.70345000
H	5.04057500	0.36700500	1.51261200
H	3.59604600	0.52087900	0.47862200
C	4.00099900	-1.48843800	1.12916000
O	3.11715700	-1.35142400	2.24132800
H	4.85000700	-2.10279400	1.45993400
H	3.48137800	-2.00379700	0.31126100
C	1.81021500	-1.59036200	2.06681400
C	1.03693200	-1.25521800	3.30693400
O	1.34171100	-2.03095100	1.04765000
H	0.01542100	-1.63708800	3.22337700
H	1.54480300	-1.66852100	4.18808600
H	1.00473900	-0.16119800	3.40416900
H	1.32219100	3.51981800	-0.05726500
S	-3.55258900	-3.85737400	-0.74054700
C	-3.85840400	-2.51736100	0.44006100
C	-4.59489200	-1.31451000	-0.13464300
H	-2.89964100	-2.21856600	0.89520000
H	-4.07776700	-0.97123400	-1.04685800
H	-5.60141000	-1.62884800	-0.45692500
H	-4.44514800	-2.97409600	1.25501000
C	-4.69720500	-0.15915600	0.85863700
H	-3.68396900	0.10470700	1.21283100
H	-5.24943700	-0.49495500	1.75219600
C	-5.37122100	1.07042500	0.26227600
H	-5.41797300	1.89843200	0.98521400
H	-4.82453500	1.42742300	-0.62520300
H	-6.40084000	0.84227000	-0.05415500

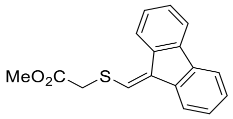

B

C	1.94671500	-1.97172900	-0.63301100
S	0.90896700	-0.52312100	-0.97619600
H	1.94400500	-2.20270900	0.44177200
H	1.57161300	-2.85223500	-1.17386400
C	-0.60503900	-1.13803500	-0.37933000
C	-1.78216900	-0.47394900	-0.37566300
H	-0.56898700	-2.15554800	0.02264000
C	-3.04931100	-1.03657900	0.14357600
C	-2.10661300	0.88654900	-0.84955300
C	-3.33268200	-2.27941500	0.71026500
C	-4.07183900	-0.07484200	-0.00845900
C	-1.31811800	1.88203200	-1.43300700
C	-3.48607300	1.12347400	-0.62693600
C	-4.64011500	-2.54843800	1.12040200
H	-2.55265800	-3.03355800	0.83451500
C	-5.37646900	-0.34736200	0.40244800
C	-1.91328900	3.09693700	-1.78583100
H	-0.25392800	1.73731500	-1.62011000
C	-4.07286100	2.33593400	-0.98069500
C	-5.65310200	-1.59301700	0.96872200
H	-4.87534800	-3.51697900	1.56534500
H	-6.16560600	0.39793600	0.28388700
C	-3.27496000	3.32362900	-1.56318900
H	-1.30308400	3.87843800	-2.24185600
H	-5.13636500	2.51084600	-0.80594800
H	-6.66769400	-1.82572800	1.29666700
H	-3.71727800	4.28017000	-1.84703700
C	5.51302600	-2.61226200	-1.19827000
H	5.99411600	-3.55780900	-0.92958100

H	5.60550800	-2.43416300	-2.27797500
H	5.98040400	-1.78161600	-0.65273000
C	3.36496800	-1.69448000	-1.07136800
O	3.74785500	-0.67043400	-1.57083800
O	4.14107300	-2.74373100	-0.82903600

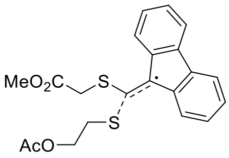

B-TS1

C	1.84798800	-2.09747300	-0.77074600
S	0.72443200	-0.80072100	-1.35212200
H	1.79342300	-2.15103300	0.32886200
H	1.56567900	-3.07847400	-1.17932800
C	-0.68333000	-1.26714500	-0.43206600
C	-1.86282100	-0.54700200	-0.42348400
H	-0.71930800	-2.31873200	-0.13626100
C	-3.11437300	-1.05480700	0.16076100
C	-2.14740700	0.81123100	-0.90270900
C	-3.40332700	-2.27595800	0.76995700
C	-4.11029100	-0.06038000	0.04053200
C	-1.34762000	1.76924500	-1.53740700
C	-3.50860800	1.10623700	-0.62338700
C	-4.69682300	-2.49286200	1.25071800
H	-2.64065100	-3.05123300	0.87215500
C	-5.39910400	-0.28187500	0.51819200
C	-1.91068200	3.00267800	-1.87729600
H	-0.29936200	1.58152900	-1.77107600
C	-4.06221100	2.33343600	-0.96902300
C	-5.68451500	-1.50855900	1.12503000
H	-4.94051400	-3.44248100	1.73013000
H	-6.17028000	0.48522600	0.42392300
C	-3.25137100	3.28312000	-1.59936200
H	-1.29183000	3.75428400	-2.36985600
H	-5.10947400	2.55243800	-0.75201800
H	-6.68868200	-1.70124800	1.50662900
H	-3.66965700	4.25196100	-1.87753300
S	-0.13593100	-0.72229500	1.96275500
C	0.65961400	0.86362200	1.62345000
H	0.35309800	1.58860200	2.39223700
H	0.28315900	1.23857400	0.65629200
C	2.16835200	0.74067600	1.57944600
H	2.56495600	0.36606900	2.53519900
H	2.48474800	0.04963500	0.78504900
O	2.70328400	2.03587200	1.31209900
C	4.03792400	2.11865500	1.22884800
C	4.48593600	3.51670700	0.91985300
O	4.75863500	1.16769300	1.38696100
H	5.57945900	3.55833700	0.89744000
H	4.07702400	3.82387600	-0.05288500
H	4.09382000	4.20696700	1.67918300
C	5.48229400	-2.48502200	-1.07413500
H	6.02459700	-3.34486800	-0.66914000
H	5.63925600	-2.41421900	-2.15863300
H	5.82744800	-1.55865300	-0.59603000
C	3.26220300	-1.76447200	-1.18307100
O	3.59009900	-0.77729300	-1.78651900
O	4.10314600	-2.70819100	-0.78108200

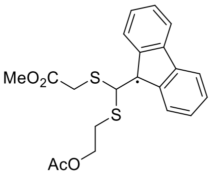

B-Int1

C	1.93604900	-2.16440300	-0.60814500
S	0.74819900	-0.90311700	-1.12680500
H	2.14073700	-2.06140900	0.47008000
H	1.54307400	-3.17757700	-0.77850500
C	-0.48444300	-1.17137900	0.18840200
C	-1.75522700	-0.44543100	-0.08479100

H	-0.69754500	-2.25005500	0.22825600
C	-3.04295700	-0.96756000	0.31891100
C	-1.98379300	0.84036500	-0.70947500
C	-3.38631400	-2.15624700	0.97770000
C	-4.05402700	-0.04236900	-0.05889100
C	-1.11645400	1.79492600	-1.27262000
C	-3.39049600	1.09290300	-0.70943200
C	-4.73253300	-2.40809000	1.24388200
H	-2.62409100	-2.87612000	1.28343600
C	-5.39238100	-0.30314600	0.20854600
C	-1.65284800	2.95904800	-1.82214800
H	-0.03608100	1.64578600	-1.28112600
C	-3.91215300	2.25548400	-1.26083900
C	-5.72517100	-1.49501200	0.86387800
H	-5.01627900	-3.32891600	1.75616100
H	-6.17033800	0.40519600	-0.08331000
C	-3.03376900	3.19037200	-1.82118100
H	-0.98198400	3.70079000	-2.25849200
H	-4.98856300	2.43850400	-1.25654500
H	-6.77131300	-1.71540400	1.08293000
H	-3.42842600	4.10891500	-2.25859000
S	0.10802000	-0.84276900	1.91211700
C	0.72218800	0.86263900	1.79600600
H	0.45377800	1.36155500	2.73774700
H	0.19578200	1.38325500	0.98391100
C	2.22322400	0.91178800	1.59122600
H	2.75110600	0.39720600	2.40853600
H	2.51370700	0.43914800	0.64147000
O	2.59342600	2.28943200	1.57203000
C	3.89436900	2.55341900	1.38709400
C	4.16050200	4.02953600	1.37427300
O	4.71829200	1.68718300	1.25101400
H	5.23039600	4.21145800	1.23249300
H	3.58600200	4.49792200	0.56316100
H	3.82212200	4.47239400	2.32104800

C	5.35235800	-2.93839100	-1.69223100
H	5.90188100	-3.81700100	-1.34028700
H	5.24847200	-2.96992400	-2.78510500
H	5.88318600	-2.02050600	-1.40603900
C	3.23580700	-1.99997800	-1.35781500
O	3.49783600	-1.09878200	-2.10961300
O	4.07185200	-2.99079200	-1.06623700

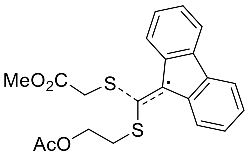

B-TS2

H	-7.91313400	-1.59476700	-0.44109000
C	-6.89510500	-1.96768700	-0.29140900
C	-5.90560200	-0.92033400	-0.70747200
H	-6.73640300	-2.22204500	0.76567700
H	-6.73415000	-2.88017300	-0.88181600
O	-4.64391000	-1.34441900	-0.54537400
O	-6.17108000	0.17161700	-1.13668600
C	-3.61885700	-0.42336700	-0.90597800
C	-2.30384500	-1.10235100	-0.58559300
H	-3.73103200	0.50304700	-0.32452500
H	-3.70225100	-0.17824700	-1.97627000
S	-0.93550100	-0.01143100	-1.07666900
H	-2.21475700	-1.27138100	0.49682800
H	-2.21524300	-2.05393100	-1.12822800
C	0.39625300	-0.96248700	-0.47113700
C	1.70964400	-0.53799500	-0.47501000
H	0.19763800	-2.03607300	-0.41008800
C	2.85452500	-1.43077200	-0.23174700
C	2.26461200	0.80535200	-0.67790000
C	2.90020700	-2.79855200	0.03787300
C	4.04528500	-0.67374900	-0.30235100
C	1.65390700	2.04280700	-0.91419600
C	3.67911300	0.72367300	-0.58161500

C	4.14599500	-3.39937500	0.23374100
H	1.98609300	-3.39350700	0.09678300
C	5.28514300	-1.27766600	-0.11081100
C	2.45997500	3.17528000	-1.06039700
H	0.56963500	2.14661600	-0.96301700
C	4.47349200	1.85428200	-0.73383100
C	5.32545700	-2.64877400	0.15889900
H	4.20036800	-4.46821600	0.44729700
H	6.20630900	-0.69429700	-0.16662900
C	3.85248700	3.08378600	-0.97765900
H	1.99173900	4.14417700	-1.24138100
H	5.56056500	1.78523600	-0.66069700
H	6.28740000	-3.14049800	0.31370300
H	4.46076700	3.98163400	-1.09967200
S	0.19342500	-0.90270900	2.04631300
C	0.01135200	0.88237500	2.13529200
H	0.11794400	1.16317700	3.19807500
H	0.82941400	1.38952100	1.60314300
C	-2.36873500	3.24762900	0.64303500
H	-3.13384300	3.31339800	1.42793500
H	-2.08301900	4.25019200	0.30852900
H	-2.76703000	2.67071900	-0.20473100
C	-1.32578300	1.42161600	1.67041800
O	-2.38968500	0.87531600	1.80479800
O	-1.18355100	2.63530300	1.13769000

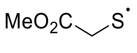

B-Int2

C	0.81373100	-0.76894800	0.06329600
H	0.75954200	-1.51826900	-0.74386100
H	0.79754700	-1.36121200	0.99501100
C	-0.43186800	0.09609700	0.02456700
O	-1.51569100	-0.67837700	-0.02157500
O	-0.46878600	1.29464900	0.04400100
C	-2.77042900	-0.00327100	-0.03502700
H	-2.84826900	0.65015300	-0.91446100
H	-3.53896000	-0.78171200	-0.07574400
H	-2.89390500	0.60116100	0.87388600
S	2.37070400	0.09602700	-0.03945400

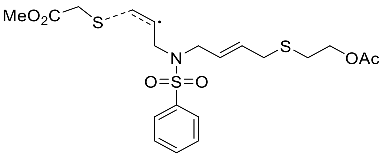

B-TS3

C	-1.81772700	-2.83701300	-1.50302300
C	-1.13577600	-2.06698800	-0.60627400
H	-2.49169600	-2.36942900	-2.22548000
H	-1.47784400	-3.85202100	-1.71880300
C	-1.39763700	-0.60433900	-0.40722000
H	-0.36838000	-2.49541900	0.04582300
N	-0.15141200	0.15107700	-0.44070400
H	-2.08824100	-0.22574600	-1.18104700
H	-1.85932400	-0.44003200	0.57885000
S	0.25195400	1.14980200	0.79617100
C	0.56731400	0.27091000	-1.70932900
O	-0.45296400	0.68825200	1.98477400
O	1.70334400	1.29064700	0.81041900
C	-0.42729500	2.73823800	0.35871900
C	1.80013000	-0.58310600	-1.77530000
H	0.81537700	1.32513300	-1.91791100
H	-0.14685900	-0.04054900	-2.48881700
C	0.38863000	3.70631400	-0.22319300
C	-1.79095300	2.95281500	0.56969100
C	2.96142500	-0.17445200	-2.29110800
H	1.70115800	-1.59860000	-1.37385000
C	-0.18243500	4.91916300	-0.61124000
C	-2.34791300	4.16667000	0.17469700
H	-2.40148400	2.18340400	1.04684100
C	4.18493700	-1.02665200	-2.34988800
H	3.06368800	0.85652400	-2.65242700

C	-1.54480900	5.14607600	-0.41590200
H	0.44248200	5.68962600	-1.06532700
H	-3.41141900	4.35213500	0.33401100
H	4.62245700	-1.03753700	-3.35949900
H	3.96036700	-2.06498800	-2.06729400
S	5.53140400	-0.35962100	-1.29608800
H	-1.98584200	6.09639500	-0.72129100
C	4.65090000	-0.17012700	0.27871100
H	5.34303900	0.36754300	0.94313700
H	3.75381500	0.45017700	0.13997000
C	4.26006900	-1.50087200	0.89353600
O	3.56345500	-1.25681300	2.11521300
H	5.15188300	-2.09278700	1.14152500
H	3.61958000	-2.08132000	0.21696300
C	2.23673300	-1.44349500	2.15453400
C	1.67471500	-0.99595800	3.47067700
O	1.60222700	-1.92067700	1.24798000
H	0.63911700	-1.33396800	3.56785900
H	2.29645000	-1.37470400	4.29232100
H	1.69590300	0.10246400	3.49484300
H	1.45366400	3.51339400	-0.35929900
S	-3.65042600	-3.78874100	-0.23038200
C	-4.14440000	-2.29241300	0.63241400
H	-3.40373400	-1.97883500	1.38197400
H	-5.05610400	-2.54193100	1.20345000
O	-4.60000200	-0.00244700	0.48555700
C	-4.49074100	-1.11084200	-0.25118700
C	-4.95487400	1.18578800	-0.21594200
H	-5.91287500	1.05414900	-0.73711500
H	-5.04493200	1.97453400	0.53811600
H	-4.18172900	1.44861600	-0.95192000
O	-4.65822200	-1.12960100	-1.44096200

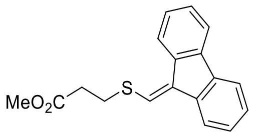

C

C	2.08519700	-1.43449900	-0.59126300
S	0.81862500	-0.16574200	-0.91936000
H	2.05372800	-1.69671600	0.47430200
H	1.84902200	-2.32933900	-1.18173600
C	-0.60163600	-1.02809700	-0.41406300
C	-1.87123500	-0.56378800	-0.43447900
H	-0.41505000	-2.04356100	-0.04902400
C	-3.04659000	-1.34607200	0.00991000
C	-2.39639000	0.74551500	-0.86987000
C	-3.14319400	-2.64225200	0.51716100
C	-4.20664900	-0.55651300	-0.14825100
C	-1.76090100	1.87999000	-1.38207000
C	-3.80306700	0.74658100	-0.69621800
C	-4.40271300	-3.13671600	0.86197800
H	-2.25511700	-3.26496400	0.64520400
C	-5.46295800	-1.05418500	0.19738800
C	-2.53354400	2.99693000	-1.71332900
H	-0.68138200	1.91630700	-1.52954300
C	-4.56708700	1.86268600	-1.02821700
C	-5.55223000	-2.35179700	0.70428900
H	-4.49220000	-4.14913600	1.25992200
H	-6.35873400	-0.44199200	0.07428300
C	-3.92106400	2.99080100	-1.53928700
H	-2.04350100	3.88603500	-2.11376900
H	-5.65038500	1.85590200	-0.89155300
H	-6.52616400	-2.75956000	0.98062700
H	-4.50296700	3.87505000	-1.80521200
C	3.44792200	-0.88477500	-0.96974200
H	3.69278800	0.02201500	-0.39488500
H	3.48991900	-0.60335700	-2.03357400
C	6.83589600	-2.30437600	-0.84714500

H	7.72855100	-1.75688100	-1.16606700
H	6.92280500	-2.58257600	0.21193700
H	6.71780600	-3.21575600	-1.44885100
C	4.53426700	-1.90099000	-0.71642300
O	4.35763400	-3.00598300	-0.27302600
O	5.73314400	-1.42489000	-1.04633700

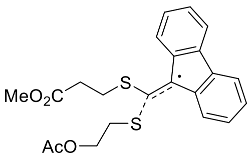

C-TS1

C	2.01844200	-1.49421500	-0.65693000
S	0.69940300	-0.36808400	-1.21061600
H	1.94056900	-1.57832300	0.43713700
H	1.84489400	-2.48501800	-1.09786700
C	-0.65209700	-1.12212200	-0.40740900
C	-1.94482300	-0.62948200	-0.44521300
H	-0.51001700	-2.18152300	-0.17663000
C	-3.11545900	-1.38939700	0.01990200
C	-2.44315100	0.68085100	-0.87741400
C	-3.21295900	-2.67735100	0.54699100
C	-4.26835000	-0.58609800	-0.12655700
C	-1.79379200	1.80452700	-1.40359100
C	-3.85084800	0.70813400	-0.68683100
C	-4.47251500	-3.15349100	0.91843700
H	-2.32846900	-3.30607100	0.67046100
C	-5.52221300	-1.06569000	0.24263900
C	-2.55107700	2.93421500	-1.72579000
H	-0.71555600	1.82226300	-1.56454400
C	-4.59714500	1.83422700	-1.01397900
C	-5.61488700	-2.35828000	0.76686700
H	-4.56713100	-4.15902300	1.33190500
H	-6.41416200	-0.44650200	0.12844100
C	-3.93589700	2.95082000	-1.53618200
H	-2.05090300	3.81367600	-2.13436000
H	-5.67864400	1.84745100	-0.86535000
H	-6.58862800	-2.75224900	1.06297400
H	-4.50645700	3.84334700	-1.79862600
S	-0.37544200	-0.66144900	2.04024000
C	0.08958200	1.07057400	1.83172600
H	-0.37935500	1.66723100	2.62841100
H	-0.33012200	1.42404100	0.87445800
C	1.59298100	1.26011300	1.82232500
H	2.03423300	0.98387000	2.79269300
H	2.05684800	0.64542000	1.04222100
O	1.85972100	2.64076800	1.56885600
C	2.96065500	2.95714700	0.87733600
C	3.07767900	4.44056300	0.68776000
O	3.74496200	2.13904800	0.46806800
H	3.98734100	4.67191000	0.12490500
H	2.19371900	4.81107400	0.15046300
H	3.10208300	4.93416500	1.66907000
C	3.36920300	-0.93947900	-1.07164800
H	3.56316400	0.04467600	-0.61234800
H	3.43673300	-0.80044200	-2.16180200
C	6.80005400	-2.20191700	-0.70291000
H	7.67551200	-1.69264400	-1.11822800
H	6.89041800	-2.27572600	0.38938600
H	6.71719800	-3.21273600	-1.12472900
C	4.48452200	-1.86382700	-0.65225600
O	4.34287300	-2.87960400	-0.02170800
O	5.66943600	-1.41337200	-1.06092600

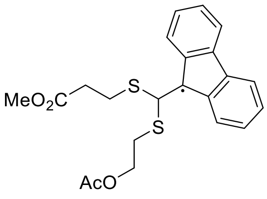

C-Int1

C	1.98619600	-1.68216800	-0.35718900
S	0.59393300	-0.72147600	-1.02012900

H	2.12437500	-1.40808600	0.69878100
H	1.74395000	-2.75323400	-0.40543100
C	-0.62130200	-1.03023900	0.30075700
C	-1.98557700	-0.56941300	-0.07629600
H	-0.65019700	-2.11773000	0.46792700
C	-3.18059000	-1.26284000	0.35266600
C	-2.40488000	0.58681000	-0.83977700
C	-3.34123200	-2.41982400	1.12744900
C	-4.31885700	-0.57090300	-0.14391100
C	-1.69044800	1.61493500	-1.48226900
C	-3.83294900	0.59097800	-0.89645400
C	-4.63385700	-2.87480900	1.38912900
H	-2.47798500	-2.95831700	1.52456900
C	-5.60236700	-1.03321700	0.12005900
C	-2.39463000	2.60883400	-2.16146300
H	-0.60079600	1.65141000	-1.45301600
C	-4.52227800	1.58542200	-1.57762300
C	-5.75201200	-2.19256700	0.89111700
H	-4.77642200	-3.77415800	1.99055200
H	-6.47807400	-0.50520600	-0.26272300
C	-3.79388500	2.59760700	-2.21356000
H	-1.84308600	3.40793400	-2.65937100
H	-5.61349500	1.57947900	-1.61532400
H	-6.75294000	-2.56909400	1.10879400
H	-4.32121100	3.38655600	-2.75235300
S	-0.16038400	-0.41259400	1.98531900
C	0.17237800	1.34686700	1.68509600
H	-0.18376300	1.89253700	2.57027200
H	-0.42133600	1.68721800	0.82531300
C	1.64846100	1.60813600	1.46269000
H	2.24188100	1.29529100	2.33569200
H	2.01648400	1.06421300	0.58201700
O	1.80111800	3.01168300	1.25629000
C	3.02597600	3.43294000	0.91300300
C	3.06320500	4.92021400	0.72146700
O	3.95803000	2.68253900	0.78376400
H	4.07248000	5.23144100	0.43436300
H	2.34141700	5.20858600	-0.05505600
H	2.76472800	5.41723800	1.65488700
C	3.24007100	-1.37633900	-1.15675800
H	3.51466900	-0.31169400	-1.08453500
H	3.10590000	-1.59325000	-2.22810600
C	6.68806100	-2.63869800	-1.00185000
H	7.47912600	-2.31074100	-1.68377500
H	6.96381100	-2.40420800	0.03524900
H	6.53558700	-3.72249900	-1.09603600
C	4.41617900	-2.18470900	-0.66682300
O	4.38959500	-2.96181100	0.25209700
O	5.51177400	-1.93124700	-1.38121200

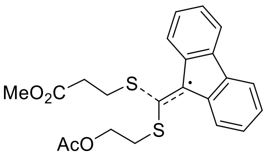

C-TS2

H	-7.65590300	-2.22326600	-0.46987000
C	-6.61876900	-2.56590100	-0.40096200
C	-5.68876500	-1.42219900	-0.67554200
H	-6.41837500	-2.96898400	0.60137600
H	-6.43611400	-3.37015600	-1.12689400
O	-4.40443800	-1.80374700	-0.60394100
O	-6.01403300	-0.29260000	-0.93128700
C	-3.43666500	-0.78779200	-0.84513000
C	-2.07671500	-1.42205100	-0.63621100
H	-3.59707500	0.05253300	-0.15370400
H	-3.54831400	-0.40748100	-1.87284500
S	-0.79196900	-0.18442800	-0.99167500
H	-1.95048500	-1.74700000	0.40754200
H	-1.94330000	-2.28332700	-1.30519100
C	0.59719800	-1.07003700	-0.40911900
C	1.88761900	-0.57628800	-0.43642500

H	0.45723800	-2.15344200	-0.37091000
C	3.08086700	-1.40606100	-0.20516200
C	2.36821000	0.79128900	-0.66169600
C	3.20359600	-2.76642600	0.07940700
C	4.22962800	-0.58920100	-0.30497100
C	1.69089200	1.99223300	-0.90546300
C	3.78704000	0.78355400	-0.59257100
C	4.48237600	-3.29917900	0.25861300
H	2.32374000	-3.40810300	0.16276700
C	5.50257000	-1.12546600	-0.12910300
C	2.43549100	3.16164200	-1.08332800
H	0.60223000	2.04665900	-0.93201300
C	4.51960200	1.95064300	-0.77646600
C	5.61968700	-2.48921100	0.15429900
H	4.59605300	-4.36116700	0.48294100
H	6.39062100	-0.49513000	-0.20707500
C	3.83213900	3.14325400	-1.02531600
H	1.91404600	4.10248300	-1.26720900
H	5.61003700	1.93772600	-0.72419400
H	6.60863600	-2.92812700	0.29726600
H	4.39119100	4.06918400	-1.17094700
S	0.43869100	-1.03901200	2.08712400
C	0.00317400	0.71255500	2.17972000
H	0.45302900	1.11389000	3.09938600
H	0.52322900	1.22187500	1.35209100
C	-1.49584200	1.00828200	2.11783400
H	-1.89087400	1.28270900	3.10941900
H	-2.07095100	0.12709900	1.79955800
C	-3.56263600	3.13222400	-0.05836200
H	-4.62888800	2.94857700	-0.22701300
H	-3.01048800	3.04054700	-1.00422600
H	-3.41180200	4.14072100	0.35020700
O	-3.12972600	2.14241800	0.86858000
C	-1.83083000	2.14200500	1.17974600
O	-1.05608200	2.96017700	0.75409700

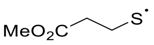

C-Int2

S	-1.52253800	1.44048800	-1.50524800
C	-1.44470700	0.48760400	0.02236400
C	-0.32139400	-0.53076400	0.05702500
H	-2.42010000	-0.02198500	0.09811900
H	-0.35235900	-1.11835500	0.98805900
H	-0.40338700	-1.24615900	-0.77573800
H	-1.38133900	1.17785900	0.87589700
C	1.03195200	0.13102800	-0.02981300
C	3.34479800	-0.23076400	-0.02918900
H	3.50238400	0.28043200	-0.98862800
H	3.52993000	0.47805000	0.78924400
H	4.02136900	-1.08717600	0.05542500
O	2.02246700	-0.75448900	0.04920100
O	1.21219100	1.31597000	-0.15701900

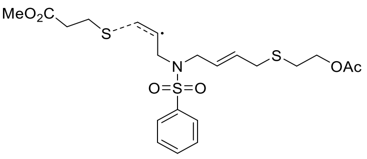

C-TS3

C	-1.27520300	-3.01027300	-1.75192700
C	-0.73129700	-2.20587000	-0.79004600
H	-1.89713000	-2.57471900	-2.53882500
H	-0.82801000	-3.98400500	-1.96171300
C	-1.11041400	-0.76922800	-0.59408800
H	0.00586400	-2.59233100	-0.07921400
N	0.08201300	0.06952100	-0.54177500
H	-1.77596600	-0.42299600	-1.40324900
H	-1.64308700	-0.65308900	0.36319500
S	0.32464800	1.09769000	0.71198400
C	0.87279700	0.24448000	-1.76017400
O	-0.42321700	0.58778400	1.85354800
O	1.75788200	1.34382000	0.82295500

C	-0.43664900	2.63353000	0.22161100
C	2.16557300	-0.51854700	-1.74297300
H	1.05792900	1.31426300	-1.95427600
H	0.23657100	-0.11516200	-2.58537100
C	0.34498400	3.65433200	-0.31580500
C	-1.82217200	2.75582500	0.34973000
C	3.32157500	-0.03297400	-2.20088900
H	2.11610700	-1.53409100	-1.33244100
C	-0.28218900	4.82532800	-0.74373600
C	-2.43468300	3.92963700	-0.08221800
H	-2.40547200	1.94828300	0.79770400
C	4.60455700	-0.79458900	-2.17903100
H	3.36979700	0.99873200	-2.57123700
C	-1.66567400	4.96029000	-0.62962000
H	0.31551000	5.63571500	-1.16367500
H	-3.51530900	4.04430500	0.01604800
H	5.10055900	-0.78082800	-3.16115200
H	4.43946700	-1.84446700	-1.89821100
S	5.83475800	-0.02397700	-1.05571800
H	-2.15085900	5.87876100	-0.96412000
C	4.84781400	0.12238400	0.45946100
H	5.46770500	0.69815900	1.16218500
H	3.92895300	0.69270300	0.26182900
C	4.49044200	-1.22585600	1.05572200
O	3.70993400	-1.01563900	2.23163300
H	5.39582900	-1.77087100	1.35731900
H	3.92151600	-1.83878200	0.34488700
C	2.40300800	-1.31342000	2.20734500
C	1.73829100	-0.88990900	3.48305300
O	1.85946100	-1.86228000	1.28258700
H	0.72414600	-1.29666100	3.52740700
H	2.33717000	-1.21751300	4.34297400
H	1.68517800	0.20758200	3.49306900
H	1.42698700	3.53488700	-0.38623400
S	-3.07414900	-4.18094100	-0.65182700
C	-3.73743100	-2.78714600	0.29067000
C	-4.39521900	-1.72334800	-0.57302000
H	-2.93987500	-2.35684800	0.91337400
H	-3.74951200	-1.42064300	-1.40934400
H	-5.31841600	-2.11368500	-1.03131300
H	-4.46820500	-3.21634700	0.99406700
C	-4.76401200	-0.49038400	0.21310600
C	-5.50213100	1.72747000	0.05381100
H	-5.64001400	2.47164500	-0.73735400
H	-6.45480100	1.55438200	0.57406600
H	-4.76030800	2.07910700	0.78328300
O	-5.06143100	0.53525100	-0.58792600
O	-4.80666900	-0.41979600	1.41492900

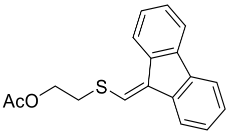

D

H	7.95553300	1.00474000	-0.57794500
C	6.96648900	1.36000900	-0.88341700
C	5.91175400	0.70285000	-0.04460100
H	6.79039200	1.12748300	-1.94272100
H	6.90211100	2.45052600	-0.76581800
O	4.68163800	1.11427200	-0.38989800
O	6.10219900	-0.09547800	0.83432100
C	3.60126200	0.55129000	0.34541200
C	2.33276400	1.15980000	-0.21707600
H	3.60474700	-0.54374400	0.23378400
H	3.71735000	0.78307900	1.41515600
S	0.90900700	0.47390400	0.68796400
H	2.23220400	0.91475200	-1.28292400
H	2.34526200	2.25089200	-0.09240100
C	-0.38360700	1.30609000	-0.12201100
C	-1.70197800	1.17822000	0.14701200
H	-0.06469100	1.98788900	-0.91701900

C	-2.77070400	1.90908800	-0.57110500
C	-2.38765600	0.34069700	1.15153000
C	-2.70479800	2.83342900	-1.61407000
C	-4.02077100	1.53419800	-0.03187800
C	-1.89867800	-0.55334600	2.10793200
C	-3.78384500	0.55741300	1.04113900
C	-3.89325900	3.37480300	-2.10835200
H	-1.74552000	3.13314700	-2.04134000
C	-5.20554100	2.07721000	-0.52837900
C	-2.80527500	-1.21770000	2.93926600
H	-0.83194900	-0.74619800	2.22297600
C	-4.68173300	-0.10769300	1.87248300
C	-5.13220200	3.00146500	-1.57195100
H	-3.85568400	4.09900200	-2.92413700
H	-6.17096500	1.78572300	-0.10978000
C	-4.18153800	-0.99906500	2.82476900
H	-2.42998300	-1.91716100	3.68820900
H	-5.75612100	0.06447000	1.78147300
H	-6.04790400	3.43796600	-1.97453100
H	-4.86996400	-1.52942300	3.48506800

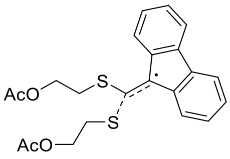

D-TS1

H	7.43436500	-2.80155500	-0.55558200
C	6.39504400	-3.00518000	-0.27971500
C	5.50298000	-1.94296800	-0.84863500
H	6.28944900	-3.02506900	0.81372800
H	6.08604500	-3.98749500	-0.66281800
O	4.21730600	-2.15727600	-0.52786700
O	5.85460500	-1.00392400	-1.51245200
C	3.28039500	-1.20252400	-1.01441700
C	1.91786900	-1.66365900	-0.53771500
H	3.53176300	-0.20435200	-0.62248900
H	3.33017600	-1.15912100	-2.11331000
S	0.63746600	-0.54829200	-1.18996500
H	1.85685100	-1.63830000	0.56070800
H	1.71605000	-2.68452500	-0.89022100
C	-0.73215900	-1.18671000	-0.31871500
C	-2.00940800	-0.66409300	-0.40513400
H	-0.61902800	-2.22307700	0.01126300
C	-3.19809000	-1.34688700	0.12902400
C	-2.47499500	0.61246700	-0.95889000
C	-3.32590900	-2.57685800	0.77454300
C	-4.33032000	-0.53199800	-0.09263400
C	-1.79854000	1.66496000	-1.58777500
C	-3.88109800	0.69298400	-0.77195300
C	-4.59592400	-2.98399200	1.18985600
H	-2.45628100	-3.21278500	0.95494900
C	-5.59486700	-0.94314700	0.32024600
C	-2.52772000	2.77845200	-2.01436900
H	-0.72059800	1.64122200	-1.75026300
C	-4.59939600	1.80210000	-1.20311700
C	-5.71831100	-2.17799800	0.96396300
H	-4.71488900	-3.94322400	1.69651700
H	-6.47146500	-0.31556300	0.14780700
C	-3.91112100	2.84791800	-1.82708900
H	-2.00625100	3.60284100	-2.50315100
H	-5.67983700	1.85685000	-1.05645100
H	-6.70089400	-2.51779600	1.29586100
H	-4.45941400	3.72642100	-2.17144600
S	-0.43746600	-0.50025300	2.07649900
C	0.07234400	1.19219900	1.71039000
H	-0.39231200	1.87345600	2.43880900
H	-0.32540400	1.46209900	0.71719800
C	1.57852000	1.34566700	1.71281200
H	2.00192700	1.12844400	2.70601700
H	2.04169900	0.66131500	0.98908100
O	1.87283000	2.69520100	1.35209700

C	3.15227300	2.97996800	1.08022300
C	3.32967400	4.42276900	0.70952300
O	4.02903100	2.15612600	1.13418800
H	4.38501100	4.62402600	0.50066800
H	2.71810800	4.65145300	-0.17427100
H	2.97730600	5.05961000	1.53260200

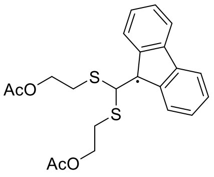

D-Int1

H	7.41665900	-2.88510200	-0.75923000
C	6.41209400	-3.07836300	-0.37015100
C	5.44437300	-2.09392500	-0.95586500
H	6.40596200	-2.99258000	0.72511100
H	6.09628100	-4.09905700	-0.62669000
O	4.20095200	-2.29140300	-0.49311500
O	5.71050100	-1.22493800	-1.74387100
C	3.19731200	-1.41071800	-0.98995500
C	1.89578100	-1.81653300	-0.32837800
H	3.46405200	-0.37033400	-0.74752400
H	3.14092000	-1.49879100	-2.08598400
S	0.54403300	-0.80090800	-0.99380600
H	1.95383800	-1.64671200	0.75711900
H	1.68766100	-2.87986400	-0.51480900
C	-0.69754600	-1.08462200	0.31044600
C	-2.03813600	-0.56886900	-0.08133500
H	-0.76759600	-2.17263300	0.45988300
C	-3.26375400	-1.22001600	0.32750400
C	-2.40372300	0.60939100	-0.83882800
C	-3.47809300	-2.37595200	1.09076000
C	-4.36859800	-0.48042000	-0.17559900
C	-1.64372300	1.61570000	-1.46342900
C	-3.82982000	0.66831800	-0.91187200
C	-4.79035300	-2.78239100	1.33408800
H	-2.64140600	-2.95097400	1.49344400
C	-5.67204100	-0.89431800	0.07011400
C	-2.30189900	2.64152200	-2.14128000
H	-0.55390900	1.61200500	-1.42153000
C	-4.47312500	1.69411800	-1.59125800
C	-5.87539600	-2.05307400	0.82969000
H	-4.97471700	-3.68013800	1.92646500
H	-6.52195600	-0.32907500	-0.31758400
C	-3.69979200	2.68373700	-2.20941400
H	-1.71466200	3.42362100	-2.62514700
H	-5.56325300	1.72972500	-1.64127300
H	-6.89260200	-2.39191100	1.03330500
H	-4.19073300	3.49685500	-2.74659700
S	-0.24042400	-0.50690400	2.00874900
C	0.16227100	1.24238700	1.73590900
H	-0.19139200	1.79210600	2.61946600
H	-0.40003200	1.61291900	0.86757300
C	1.65145300	1.44740200	1.54433600
H	2.21480800	1.11420800	2.42966400
H	2.01551900	0.88685100	0.67276900
O	1.86218500	2.84300300	1.33744000
C	3.10686900	3.21429000	1.00763000
C	3.20564000	4.69802000	0.81163200
O	4.00998200	2.42715100	0.89142500
H	4.23175400	4.96833200	0.54345100
H	2.51163000	5.00999700	0.01902500
H	2.90769700	5.21093400	1.73650200

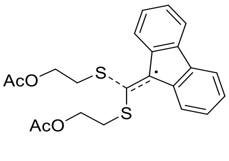

D-TS2

H	-7.43423800	-2.80129100	-0.55588600
C	-6.39496200	-3.00487000	-0.27981400

C	-5.50279400	-1.94281000	-0.84885200
H	-6.28953600	-3.02448600	0.81365200
H	-6.08592100	-3.98728500	-0.66262300
O	-4.21715200	-2.15715400	-0.52797500
O	-5.85431800	-1.00384500	-1.51283400
C	-3.28015300	-1.20255000	-1.01463800
C	-1.91766700	-1.66368400	-0.53781100
H	-3.53146800	-0.20429000	-0.62289800
H	-3.32987800	-1.15932400	-2.11354000
S	-0.63727200	-0.54828300	-1.19001100
H	-1.85673400	-1.63830700	0.56061600
H	-1.71580700	-2.68455300	-0.89028700
C	0.73237000	-1.18661700	-0.31873600
C	2.00960400	-0.66396500	-0.40519400
H	0.61925600	-2.22295600	0.01133000
C	3.19830100	-1.34664100	0.12908500
C	2.47515200	0.61254400	-0.95910100
C	3.32615000	-2.57649600	0.77482000
C	4.33050500	-0.53174200	-0.09266900
C	1.79867100	1.66493400	-1.58813100
C	3.88125100	0.69313000	-0.77216300
C	4.59616900	-2.98351600	1.19022800
H	2.45654000	-3.21241500	0.95533900
C	5.59505800	-0.94277800	0.32030700
C	2.52782100	2.77839400	-2.01486300
H	0.72073400	1.64114200	-1.75063800
C	4.59951900	1.80221100	-1.20346900
C	5.71853400	-2.17752100	0.96422400
H	4.71515500	-3.94265700	1.69705500
H	6.47163500	-0.31518600	0.14779600
C	3.91121800	2.84792800	-1.82758200
H	2.00632800	3.60270300	-2.50375500
H	5.67995700	1.85701400	-1.05680100
H	6.70112100	-2.51723000	1.29620100
H	4.45948600	3.72640300	-2.17204800
S	0.43786600	-0.49990000	2.07642800
C	-0.07246200	1.19235200	1.71011000
H	0.39222500	1.87388600	2.43824900
H	0.32491200	1.46214600	0.71674000
C	-1.57867900	1.34538700	1.71296900
H	-2.00167400	1.12839200	2.70640000
H	-2.04192900	0.66059800	0.98969000
O	-1.87353600	2.69470000	1.35189100
C	-3.15325100	2.97906500	1.08087900
C	-3.33131500	4.42174500	0.71003800
O	-4.02975100	2.15500300	1.13570800
H	-4.38674800	4.62251200	0.50119500
H	-2.71983300	4.65069400	-0.17373700
H	-2.97927200	5.05877500	1.53311700

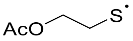

D-Int2

S	-0.68503100	-0.51128400	-2.53367500
C	-0.44483100	-1.79951200	-1.30696100
H	-1.02821700	-2.67895000	-1.62761300
H	-0.90311100	-1.45414800	-0.36502600
C	1.01120700	-2.16050400	-1.10094800
H	1.46329100	-2.52566800	-2.03516500
H	1.58992500	-1.28828600	-0.76213100
O	1.05672200	-3.18477100	-0.10982800
C	2.27274500	-3.64473000	0.21676000
C	2.19430000	-4.71367100	1.26619300
O	3.28062400	-3.22685200	-0.29011000
H	3.20066700	-5.07143700	1.50529000
H	1.71248600	-4.30880400	2.16693600
H	1.57359000	-5.54330500	0.90063200

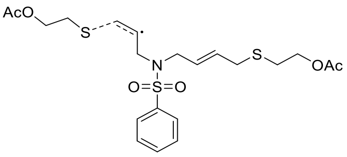

D-TS3

C	-1.58356700	-2.73985400	-2.32987500
C	-0.90822400	-2.01120800	-1.38888100
H	-2.28269000	-2.24152100	-3.00673200
H	-1.18283400	-3.70098700	-2.65690300
C	-1.24756000	-0.59348700	-1.04225300
H	-0.12873100	-2.47041100	-0.77494500
N	-0.05239300	0.21402300	-0.83586700
H	-1.89043300	-0.14489800	-1.81997600
H	-1.80424400	-0.57804200	-0.09237100
S	0.12611800	1.13602800	0.50689800
C	0.91671800	0.37242600	-1.92049200
O	-0.92679500	0.74816300	1.44221300
O	1.51914700	1.09839500	0.93871000
C	-0.20209400	2.80858900	-0.01800100
C	2.10807300	-0.53299100	-1.79045700
H	1.24008900	1.42435400	-1.99257500
H	0.36928500	0.14945600	-2.85092400
C	0.83237800	3.74023000	-0.04139300
C	-1.50562900	3.14044300	-0.39378000
C	3.37220100	-0.13718300	-1.95521500
H	1.89409600	-1.58233500	-1.55664900
C	0.55179300	5.04375200	-0.45465300
C	-1.76964500	4.44476400	-0.80368700
H	-2.30643400	2.39647000	-0.37223700
C	4.54205000	-1.05500600	-1.82338500
H	3.59246800	0.91848400	-2.15756000
C	-0.74369500	5.39366000	-0.83330000
H	1.35070100	5.78642700	-0.47827100
H	-2.78255900	4.72199700	-1.09979400
H	5.19593000	-1.00348300	-2.70706700
H	4.21406400	-2.09765400	-1.70621000
S	5.64425800	-0.57811100	-0.43611000
H	-0.95835000	6.41426300	-1.15462500
C	4.43686700	-0.41130100	0.90774600
H	5.00246400	-0.01351800	1.76310200
H	3.66286100	0.31931600	0.63516700
C	3.78031900	-1.72734900	1.27935300
O	2.86615800	-1.49116100	2.35020500
H	4.52777700	-2.44961100	1.63714400
H	3.24078300	-2.16050000	0.42731200
C	1.55109300	-1.60663800	2.12629300
C	0.76377800	-1.15640000	3.32037900
O	1.07980100	-2.02890600	1.10035400
H	-0.28071100	-1.46569700	3.21719600
H	1.20690200	-1.56037100	4.23966100
H	0.81197800	-0.05931400	3.36188700
H	1.83766900	3.44730600	0.26323900
S	-3.23444500	-3.98893900	-1.09072200
C	-3.46143600	-2.84213900	0.28739900
C	-4.24810600	-1.61376300	-0.12478200
H	-2.48214300	-2.55764800	0.70247300
H	-3.82166700	-1.15146000	-1.02398300
H	-5.29432700	-1.87607200	-0.34354600
H	-3.98944600	-3.39759900	1.07838700
O	-4.22058900	-0.67757400	0.95715400
C	-4.16653700	0.62733000	0.66837000
O	-4.16542300	1.05170900	-0.46225200
C	-4.06902900	1.47241900	1.90220100
H	-4.63457000	1.02787000	2.72964400
H	-3.00631300	1.51579800	2.18510900
H	-4.42664500	2.48479300	1.68345900

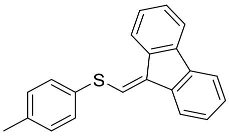

E

S	-0.77511900	-0.26586000	1.60941100
C	0.49946700	-1.08687100	0.75174200
C	1.75723100	-0.62565700	0.57803600

H	0.23208500	-2.07384500	0.36153100
C	2.81966300	-1.38643000	-0.11819400
C	2.37450700	0.65328000	0.98591000
C	2.79833400	-2.65052500	-0.70695600
C	3.99941300	-0.61171500	-0.13543600
C	1.86327000	1.75798200	1.67206900
C	3.72350200	0.65889900	0.55149300
C	3.96292000	-3.12824800	-1.31166900
H	1.89261600	-3.26072300	-0.69878400
C	5.16067800	-1.09262000	-0.73936800
C	2.69931300	2.85262900	1.91114100
H	0.83201300	1.78885800	2.02386900
C	4.55217500	1.75096700	0.79575400
C	5.13296800	-2.35836500	-1.32805500
H	3.96128000	-4.11559200	-1.77690100
H	6.07281000	-0.49253000	-0.75230300
C	4.02936000	2.85092300	1.48006100
H	2.30543600	3.71899800	2.44521400
H	5.59067000	1.74754000	0.45863200
H	6.03145200	-2.75285700	-1.80586500
H	4.66392300	3.71574800	1.68137600
C	-2.19608200	-1.24273900	1.16872100
C	-2.42344200	-1.67289300	-0.14110100
C	-3.13583000	-1.52487500	2.16611700
C	-3.57291000	-2.40395400	-0.43855200
H	-1.71025000	-1.43445400	-0.93302700
C	-4.28884400	-2.23732600	1.84873400
H	-2.96132800	-1.19315600	3.19170100
C	-4.52464400	-2.69649800	0.54535600
H	-3.73899700	-2.74140200	-1.46410000
H	-5.01751200	-2.45227600	2.63409300
C	-5.76201200	-3.48989100	0.22596800
H	-5.71322600	-4.48839700	0.68747900
H	-6.66102900	-2.99397200	0.61954800
H	-5.88159500	-3.62212900	-0.85729300

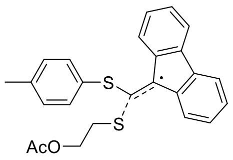

E-TS1

S	-0.69321800	-0.24186800	1.32484100
C	0.52802000	-0.94545100	0.29767400
C	1.85503600	-0.53961500	0.33884100
H	0.30335700	-1.95466200	-0.05758500
C	2.94474700	-1.29842200	-0.29168300
C	2.46383000	0.65820100	0.92665600
C	2.92364300	-2.49445600	-1.00980900
C	4.15981000	-0.60793500	-0.08412100
C	1.92065800	1.73902700	1.63213100
C	3.86211800	0.61458300	0.67700900
C	4.12857100	-2.99441500	-1.50931900
H	1.99003300	-3.03397200	-1.18411600
C	5.35868600	-1.11236600	-0.58016600
C	2.77267100	2.75393300	2.07641000
H	0.85334900	1.81443000	1.84129700
C	4.70299700	1.62611100	1.12594600
C	5.33300200	-2.31359400	-1.29495000
H	4.13061700	-3.92818400	-2.07407800
H	6.29875900	-0.58126700	-0.41836900
C	4.14745600	2.69944000	1.83012600
H	2.35456300	3.59934500	2.62497100
H	5.77635700	1.58465900	0.93095000
H	6.26219600	-2.72476500	-1.69311100
H	4.79297200	3.50259000	2.18962100
S	0.28748400	-0.05487100	-2.00804900
C	-0.30431200	1.57857600	-1.51703700
H	0.12473800	2.32752300	-2.19955800
H	0.08415600	1.80386100	-0.50915600
C	-1.81617500	1.65956100	-1.51935400
H	-2.22091800	1.47362000	-2.52616800

H	-2.25615500	0.91662800	-0.83741400
O	-2.17445100	2.97423200	-1.09391800
C	-3.48469600	3.22969400	-0.98672300
C	-3.73723600	4.63703500	-0.53178800
O	-4.32892400	2.40826000	-1.23372700
H	-4.81452600	4.81076400	-0.44673100
H	-3.24835500	4.80330000	0.43799900
H	-3.29619900	5.33993100	-1.25210000
C	-2.12860000	-1.24360200	1.00396600
C	-2.38392400	-1.86840800	-0.21824100
C	-3.05727900	-1.34661000	2.04762800
C	-3.55731900	-2.60732800	-0.37983100
H	-1.68709800	-1.76570300	-1.05167300
C	-4.22762300	-2.07434600	1.86270400
H	-2.86069300	-0.86321300	3.00724200
C	-4.49705700	-2.72371400	0.64892600
H	-3.74716300	-3.09627100	-1.33805700
H	-4.94500100	-2.14896600	2.68354300
C	-5.75846900	-3.52399700	0.47427400
H	-5.76003300	-4.40013200	1.14080700
H	-6.64405900	-2.92270700	0.72731400
H	-5.86349400	-3.88034400	-0.55875400

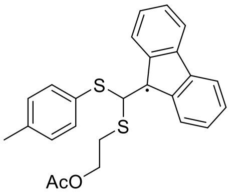

E-Int1

S	-0.76195100	-0.60911000	1.43121200
C	0.23620600	-1.09876900	-0.03938100
C	1.65831600	-0.69571100	0.12820600
H	0.19156700	-2.19612800	-0.09347100
C	2.76206600	-1.53911200	-0.27458600
C	2.21767400	0.53116400	0.65267400
C	2.77759000	-2.81443400	-0.85544400
C	3.98270600	-0.86614100	0.00803300
C	1.62601400	1.70286900	1.15860700
C	3.64189400	0.43566700	0.59367700
C	4.00957300	-3.40353300	-1.14130100
H	1.84916200	-3.34149400	-1.08547300
C	5.20453700	-1.46236600	-0.27786800
C	2.44726700	2.73984700	1.60000600
H	0.54174600	1.81273100	1.20213000
C	4.44875200	1.47455500	1.03835600
C	5.20926800	-2.73817800	-0.85589500
H	4.03937900	-4.39595800	-1.59408000
H	6.14318400	-0.94916200	-0.05936300
C	3.84249700	2.63002500	1.54590200
H	1.99302200	3.65110400	1.99246100
H	5.53656100	1.39413800	0.99049500
H	6.16020300	-3.22061800	-1.08822000
H	4.46322400	3.45521200	1.89895500
S	-0.43755200	-0.64678800	-1.69028200
C	-0.27513300	1.15868700	-1.76869600
H	-0.26460900	1.39782300	-2.84220800
H	0.69489400	1.46762400	-1.35442100
C	-1.42345300	1.87195400	-1.08531400
H	-2.38707000	1.57819800	-1.52968700
H	-1.45630600	1.63673300	-0.01312800
O	-1.22059000	3.27380700	-1.25848300
C	-2.07665900	4.07621300	-0.61219500
C	-1.76099200	5.52355500	-0.84736300
O	-2.97594300	3.65389900	0.06804100
H	-2.48901500	6.15125600	-0.32407300
H	-0.74604000	5.73839000	-0.48478500
H	-1.78146800	5.73496900	-1.92523500
C	-2.34116500	-1.30282200	0.98920300
C	-2.50199100	-2.68359800	0.84202500
C	-3.44360800	-0.45837000	0.81585000
C	-3.74843900	-3.20886100	0.50515900

H	-1.65163800	-3.35278600	0.99276800
C	-4.68892800	-0.99667200	0.49857600
H	-3.32849500	0.62216200	0.92424100
C	-4.86195500	-2.37735900	0.33171500
H	-3.86116300	-4.28877100	0.38490000
H	-5.54407400	-0.32893500	0.36788200
C	-6.21323300	-2.94053700	-0.01408100
H	-6.93094000	-2.75968800	0.80065000
H	-6.61960600	-2.45979700	-0.91619200
H	-6.16102200	-4.02273100	-0.19066500

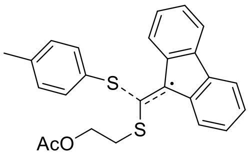

E-TS2

H	-8.64783900	-0.30480600	0.27131900
C	-7.69445500	-0.78500800	0.51275200
C	-6.59310200	-0.15246100	-0.28424700
H	-7.47745100	-0.67989700	1.58475100
H	-7.74190700	-1.85892400	0.28572100
O	-5.40573200	-0.71801800	-0.01738900
O	-6.71679600	0.74851600	-1.07120000
C	-4.28515200	-0.18902700	-0.71781800
C	-3.07589700	-0.97191400	-0.24904300
H	-4.17900300	0.88361100	-0.49409400
H	-4.44109300	-0.29508700	-1.80226600
S	-1.59876400	-0.35342500	-1.10581300
H	-2.91844400	-0.83349400	0.83023100
H	-3.20169900	-2.04160900	-0.46787900
C	-0.38797600	-1.33736400	-0.31877800
C	0.97103200	-1.20642500	-0.58520700
H	-0.74870800	-2.30719700	0.03703600
C	1.98026700	-2.17437200	-0.13631500
C	1.69892400	-0.08978700	-1.18611000
C	1.84011700	-3.40391000	0.50906600
C	3.26512700	-1.66783900	-0.44003200
C	1.26654900	1.11442200	-1.75725500
C	3.09254500	-0.35964500	-1.08928300
C	2.99223400	-4.12161800	0.83848200
H	0.85357800	-3.80264600	0.75488800
C	4.41036800	-2.38682700	-0.10960900
C	2.22018900	2.03365700	-2.20083200
H	0.20865300	1.35961700	-1.84615700
C	4.03459200	0.55922800	-1.53485900
C	4.26327600	-3.62065000	0.53206900
H	2.90009600	-5.08529500	1.34232800
H	5.40295900	-1.99658700	-0.34327300
C	3.58858300	1.76423500	-2.09012100
H	1.88933800	2.97753600	-2.63804500
H	5.10242500	0.34876800	-1.44812600
H	5.14928700	-4.19926500	0.79904900
H	4.31457100	2.49889800	-2.44298000
S	-0.32416400	-0.59882000	2.00536900
C	0.38822600	0.96087300	1.68817700
C	1.78099800	1.14610200	1.80562100
C	-0.40147800	2.04741000	1.27245400
C	2.35822500	2.37286900	1.50603100
H	2.40251800	0.30680000	2.12657500
C	0.18803500	3.27496000	0.97512400
H	-1.48185900	1.91802500	1.18446000
C	1.57292800	3.45739900	1.07898500
H	3.44051500	2.49781800	1.59601800
H	-0.43890700	4.10907400	0.65126200
C	2.21995100	4.76655800	0.72327600
H	2.87371900	5.11772700	1.53527500
H	2.85099900	4.65170700	-0.17258300
H	1.47068000	5.54196300	0.51713500

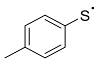

E-Int2

C	1.21503300	-1.04229100	0.00000000
C	1.21717300	0.34449700	0.00000000
C	0.00000000	1.06847200	0.00000000
C	-1.21516400	0.33568100	0.00000000
C	-1.20371100	-1.04748900	0.00000000
C	0.00953500	-1.76320800	0.00000000
H	2.16228800	-1.58549300	0.00000000
H	2.15876100	0.89629900	0.00000000
H	-2.15943300	0.88292300	0.00000000
H	-2.14704000	-1.59843900	0.00000000
S	-0.00540500	2.78544700	0.00000000
C	-0.00190300	-3.26265100	0.00000000
H	-0.53478700	-3.64385900	0.88448300
H	-0.53478700	-3.64385900	-0.88448300
H	1.01570800	-3.67278600	0.00000000

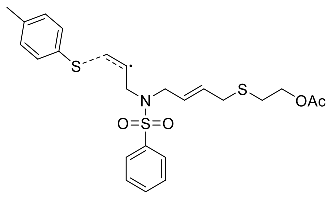

E-TS3

C	-1.52301400	-2.71123700	-2.36999700
C	-0.87551100	-2.00369400	-1.38020700
H	-2.02240500	-2.16193400	-3.17229300
H	-1.18807800	-3.72284500	-2.61000600
C	-1.13582500	-0.55344100	-1.12106300
H	-0.21403400	-2.49914500	-0.66284400
N	0.09694700	0.17135700	-0.84161600
H	-1.67132300	-0.09510900	-1.97178900
H	-1.77942700	-0.46205900	-0.23113300
S	0.20437000	1.12522500	0.48823300
C	1.06415100	0.37178100	-1.92177700
O	-0.73520800	0.61678300	1.48006100
O	1.61551600	1.27163900	0.82567400
C	-0.39048700	2.72523000	-0.02506900
C	2.29442100	-0.47569800	-1.77575300
H	1.33726900	1.43723200	-2.00771900
H	0.53453600	0.10915900	-2.85202400
C	0.50582900	3.77009200	-0.23364600
C	-1.76352200	2.87680900	-0.23393000
C	3.53445100	-0.03009500	-1.98917300
H	2.12454900	-1.51790000	-1.48066600
C	0.01036200	5.00209000	-0.66473300
C	-2.24334900	4.11034400	-0.66569300
H	-2.44505600	2.04077300	-0.05615400
C	4.75508600	-0.87438400	-1.83423900
H	3.69707200	1.02483800	-2.24323900
C	-1.35725000	5.17018800	-0.87979600
H	0.69797500	5.83293900	-0.82967500
H	-3.31322100	4.24697100	-0.83158900
H	5.40416900	-0.81412800	-2.72072100
H	4.49171400	-1.93030300	-1.67914900
S	5.82537500	-0.28058800	-0.46629000
H	-1.73886400	6.13580600	-1.21572300
C	4.61468900	-0.19550100	0.88250400
H	5.13909200	0.28392000	1.72203200
H	3.76816500	0.44402700	0.59403200
C	4.10281400	-1.56338000	1.29314500
O	3.14202500	-1.40278300	2.33647400
H	4.91864600	-2.18075100	1.69418800
H	3.63859300	-2.08623600	0.44697400
C	1.84549800	-1.60522400	2.06187500
C	0.98910200	-1.25761200	3.24262000
O	1.44387500	-2.02529900	1.00636600
H	-0.03280000	-1.61093500	3.07633900
H	1.41637500	-1.69130100	4.15608100
H	0.97661300	-0.16393000	3.34709300
H	1.57088400	3.61940800	-0.05247100
S	-3.51074100	-3.50132900	-1.43407000

C	-3.83953200	-2.14245100	-0.36932300
C	-3.41591100	-2.17625700	0.97178500
C	-4.43889500	-0.97114000	-0.85481200
C	-3.54722600	-1.05513100	1.78217500
H	-2.95293400	-3.08577800	1.35987100
C	-4.56178200	0.15140700	-0.03570700
H	-4.78021600	-0.93481000	-1.89144900
C	-4.09939600	0.13642000	1.28636100
H	-3.18774100	-1.08883600	2.81343500
H	-5.00831300	1.06478700	-0.43698700
C	-4.13866000	1.36546600	2.15024300
H	-4.65837700	2.19389000	1.65008200
H	-4.64159000	1.16388300	3.10787300
H	-3.10984500	1.68573200	2.37963300
